# Total Synthesis of an All-1,2-*cis*-Linked Repeating Unit from the *Acinetobacter baumannii* D78 Capsular Polysaccharide

**DOI:** 10.1021/acs.orglett.2c01034

**Published:** 2022-05-06

**Authors:** Dancan K. Njeri, Justin R. Ragains

**Affiliations:** Department of Chemistry, Louisiana State University, 232 Choppin Hall, Baton Rouge, Louisiana 70806, United States

## Abstract

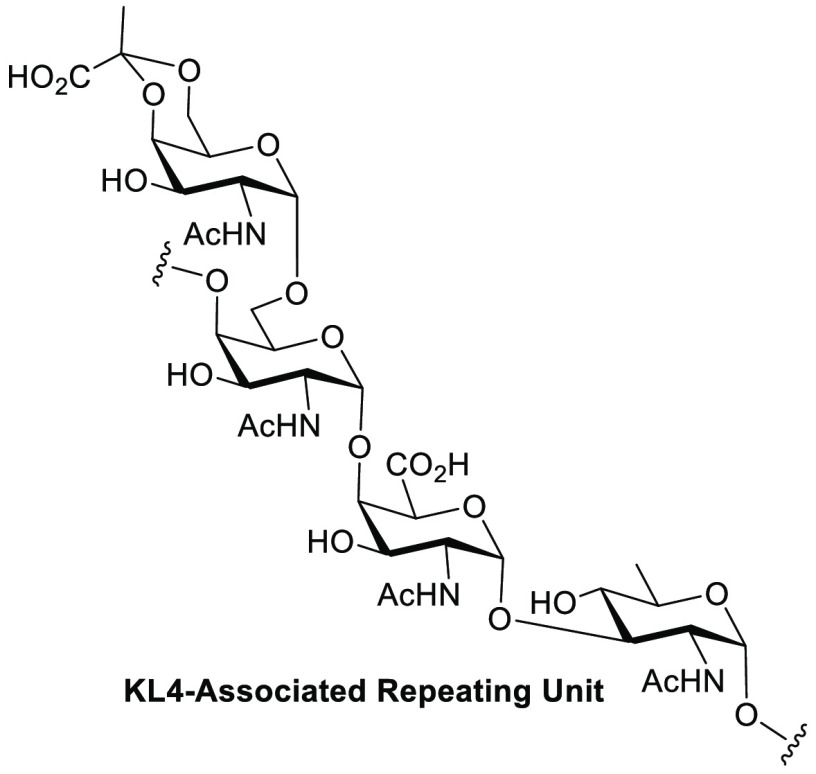

Chemical synthetic efforts have resulted in the preparation of the assigned tetrasaccharide repeating subunit from the *Acinetobacter baumannii* KL4-associated capsular polysaccharide. A convergent synthetic strategy hinging on a 1,2-*cis*-selective [2+2] glycosylation to generate the fully protected tetrasaccharide was key to the success of this synthesis.

*Acinetobacter baumannii* is a Gram-negative, opportunistic bacterial pathogen associated with illness in individuals suffering from traumatic injury as well as the immunocompromised.^1−5^ It is one of the six nosocomial “ESKAPE” pathogens associated with drug resistance and virulence,^6^ and it has been deemed an urgent threat due to the prevalence of clinically relevant strains that are extensively drug-resistant (resistant to at least one agent in all but one or two categories of antimicrobials) and even pandrug-resistant (resistant to all approved antimicrobials).^7,8^ Despite a multitude of efforts, a vaccine remains elusive.^9,10^ Meanwhile, *A. baumannii* is associated with a substantial number of capsular polysaccharide (CPS),^11^ lipooligosaccharide,^12^ and *O*-glycan structures^13^ that might prove to be promising candidates for semisynthetic glycoconjugate vaccine development.^14,15^

As part of a research program aimed at synthesizing glycans associated with the cell surface of *A. baumannii*, we became interested in the KL4 (CPS biosynthetic gene cluster)-associated repeating unit **1** depicted in Scheme 1. Originally isolated by Kenyon et al.^16^ from multidrug-resistant *A. baumannii* strain D78 and assigned using a combination of chemical and spectroscopic analysis, the repeating unit of the KL4 CPS has an intriguing structure. It consists of *N*-acetyl-d-quinovosamine (QuiNAc), *N*-acetyl-d-galactosaminuronic acid (GalNAcA), *N*-acetyl-d-galactosamine (GalNAc), and the 4,6-pyruvate ketal of *N*-acetyl-d-galactosamine (Pyr-GalNAc), a frequently occurring motif in microorganisms.^17^ Particularly striking is the fact that all glycosidic linkages are of the 1,2-*cis*/α configuration that is synthetically more challenging than 1,2-*trans*/β linkages.^18,19^ Establishing the 1 → 4 glycosidic linkage between GalNAc and GalNAcA in reasonable yield appeared to be the greatest challenge. In this work, we recount our efforts that have led to the successful synthesis of tetrasaccharide repeating unit **1**. Particularly noteworthy are two 1,2-*cis*-selective O-glycosylation reactions as well as our resorting to a convergent [2+2] synthetic approach when our initial efforts toward a linear synthesis gave substandard results.

**Scheme 1:**
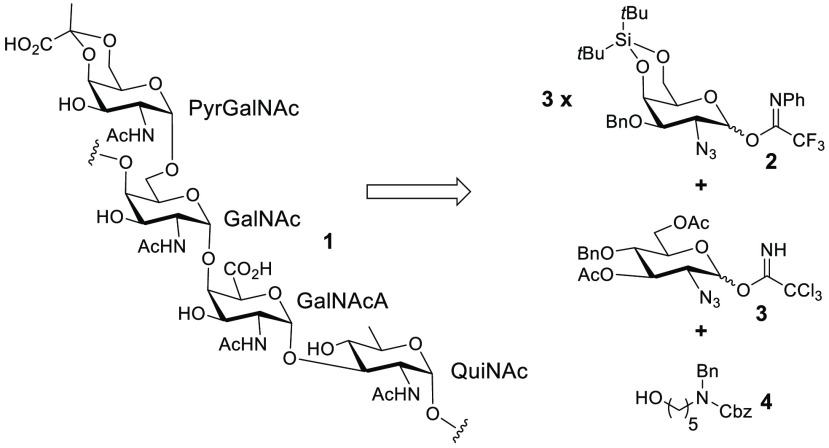
*A. baumannii* KL4-Associated CPS Subunit

Our initial retrosynthesis (Scheme 1) consisted of disconnecting **1** to 2-azido-2-deoxygalactose donor **2** (which we believed to be a dramatically simplifying common intermediate toward GalNAcA, GalNAc, and Pyr-GalNAc) and 2-azido-2-deoxyglucose donor **3**. Steric hindrance due to the bulky di-*tert*-butylsilylene (DTBS) protecting group in **2** could ensure 1,2-*cis* selectivity in the relevant O-glycosylations.^20^ Meanwhile, the 1,2-*cis*-selective O-glycosylation leading to the linkage between **3** and linker molecule **4** (which would enable eventual conjugation to carrier proteins for, e.g., vaccine development^14,15^ or conjugation to a glycan array^21^) could be carried out according to a previously developed synthetic strategy.^22^

Our synthesis of the QuiNAc-linker molecule portion **8** (Scheme 2) commenced with known benzylidene-protected 2-azido-2-deoxythioglucoside **5** (synthesized in five steps from d-glucosamine).^23^ Walking benzylidene to position 4 was effected with BH_3_·THF/TMSOTf followed by acetylation to generate **6**. Subsequent oxidative hydrolysis of thioglycoside (NBS, H_2_O, and acetone) and conversion of intermediate lactol to trichloroacetimidate (CCl_3_CN and K_2_CO_3_)^24^ furnished donor **3**. We then performed multiple attempts at O-glycosylation of linker **4** with **3** according to conditions previously reported by Boons and co-workers (TMSOTf, excess of thiophene, and low temperature).^22^ While yields were reasonable, selectivity was modest [<5:1 in favor of 1,2-*cis* relative to an unwanted byproduct that we attribute to the 1,2-*trans* isomer (data not shown)]. We attribute this to the very high reactivity of **4** resulting in modest selectivity. Coming off our recent success^25^ (and noting the successes of others)^26^ in the development of 1,2-*cis*-selective glucosylation using glucosyl imidates and a combination of either triflic acid or TMSOTf in 1,4-dioxane, we performed glycosylation of **4** with **3** using 1,4-dioxane as the solvent under dilute conditions at room temperature (∼18 °C) (Scheme 2). This furnished target glycoside **7** in 75% yield with only traces of the observable undesired byproduct. Four additional steps of manipulation (Scheme 2; methanolysis, tosylation, Finkelstein iodination, and ionic reduction with NaCNBH_3_ in diethylene glycol diethyl ether) resulted in the formation of alcohol **8**, which was ready for further manipulation.

**Scheme 2:**
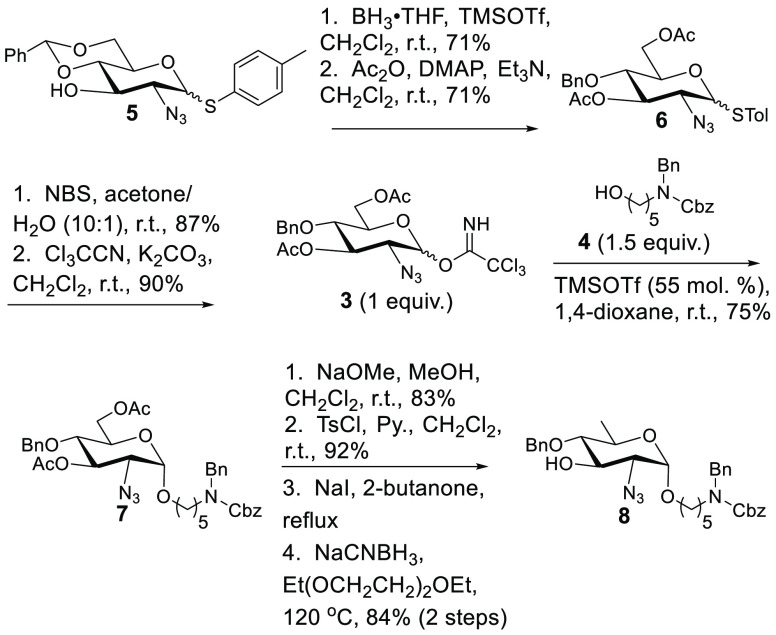
Synthesis of the QuiNAc Portion

Having reached the incipient phase of tetrasaccharide assembly (Scheme 3), we reacted alcohol **8** with DTBS-protected *N*-phenyltrifluoroacetimidate **2** (prepared in six steps from triacetyl d-galactal)^27^ in the presence of triflic acid (HOTf) to provide a high yield of disaccharide **9** as the only observed isomer. This is likely due to the bulk of DTBS that deflects “top-side” attack by the acceptor.^20^ Subsequent manipulation of **9** [DTBS removal with HF·pyridine, two-step oxidation to uronic acid,^28^ and methylation with TMSCHN_2_^29^ (Scheme 3)] resulted in alcohol **10**. While the potential low reactivity of this acceptor (due to the axial disposition of the C-4 alcohol and electron-withdrawing effects from an azido at position 2 and a -CO_2_Me at position 6) was of concern, this potential flaw may have ultimately been to our advantage (Scheme 4, *vide infra*). In any case, subsequent glycosylation of **10** with **2** (HOTf and CH_2_Cl_2_) resulted in a 79% yield of trisaccharide **11** as the only observable isomer, suggesting that the potential low reactivity of **10** was not fatal to the synthesis. Pleased with this result, we removed DTBS (HF·pyridine) and attempted glycosylation of the resulting **12**, once again, with donor **2**. To our great surprise and dismay, these attempts at selective glycosylation at position 6 of the nonreducing-end diol of **12** with **2** resulted in low yields and complex mixtures of products of apparent unselective and even double glycosylation. The cause of such low-yielding reactions with poor regioselectivity is mysterious to us at present.

**Scheme 3:**
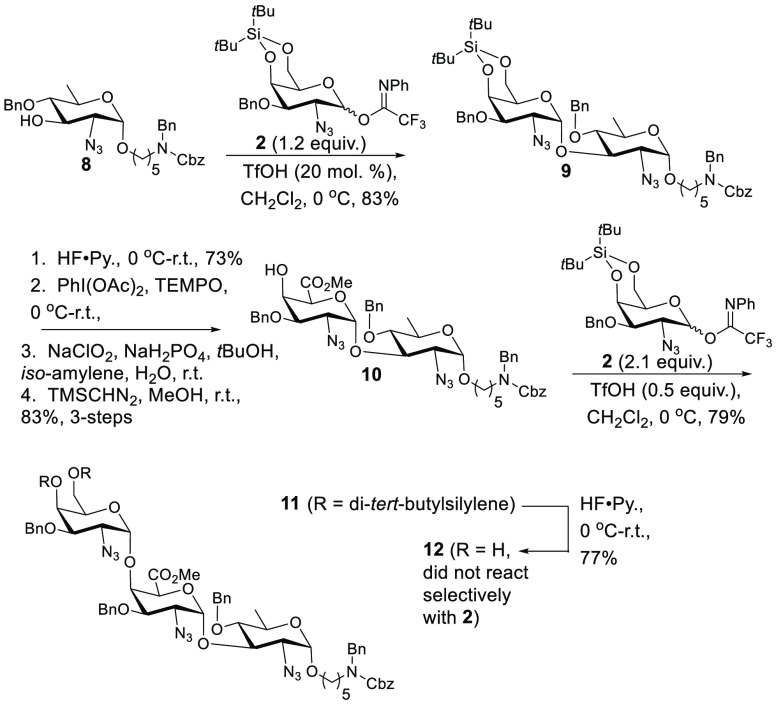
Initial Assembly of a GalNAc/GalNAcA/QuiNAc Trisaccharide

**Scheme 4:**
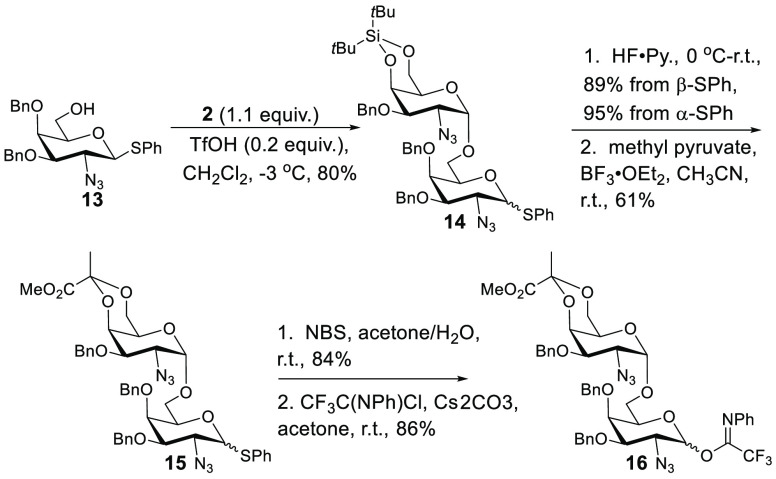
Synthesis of a Pyr-GalNAc/GalNAc Disaccharide Donor

At this stage, we considered a number of alternatives, including benzylidenation and subsequent “walking to the 4” [as with **5** → **6** (Scheme 2)] of diol **12**, but we were dissatisfied with the attendant sacrifice of synthetic efficiency. Therefore, we devised a convergent approach that, while not being without its own risks, would avoid the intermediacy of **12** and streamline the synthesis. Synthesis of a suitable Pyr-GalNAc/GalNAc portion of **1** (Scheme 4) commenced with the glycosylation of **13** (prepared in seven steps from d-galactosamine)^30^ with **2** in the presence of HOTf to generate **14** with 1,2-*cis* as the only observed configuration at the newly forged linkage. Interestingly, this process resulted in epimerization of reducing-end thioglycoside. Regardless of this complication, separation of thioglycoside epimers at this stage was facile. Subsequent DTBS removal (HF·pyridine) preceded pyruvate ketal installation under equilibrating conditions (BF_3_·Et_2_O) to furnish the thermodynamic ketal stereochemistry (**15**), which was confirmed through analysis of NMR chemical shifts.^17^ Oxidative hydrolysis of thioglycoside (NBS, H_2_O, and acetone) and conversion to *N*-phenyltrifluoroacetimidate provided disaccharide donor **16**, which was ready for coupling to acceptor **10**.

Because donor **16** lacked the highly efficacious DTBS group that practically ensures 1,2-*cis* selectivity,^20^ we approached the subsequent glycosylation with some trepidation (Scheme 5). Treatment of a mixture of donor **16** and acceptor **10** in CH_2_Cl_2_ with TMSOTf at 18 °C resulted, to our delight, in a high yield of the desired, fully protected tetrasaccharide **17** with 1,2-*cis* stereochemistry at the newly forged 1 → 4 linkage. In some instances (e.g., running the reaction at 0 °C), we could observe a minor product with a ^13^C signal appearing slightly above 100 ppm, suggesting that some of the undesired 1,2-*trans* isomer might be generated in small quantities. However, we were never able to isolate this byproduct in pure form. The 1,2-*cis* selectivity of this glycosylation may be attributable to the low reactivity of acceptor **10** and equilibration of anomeric triflates derived from **16** with the equatorial triflate (or an ion pair derived from it) being more reactive than the axial triflate as has been suggested by Codée and co-workers.^31^ Blocking of “top-side attack” of **10** by the axial 4-position benzyloxy group in **16** may also be a factor.

**Scheme 5:**
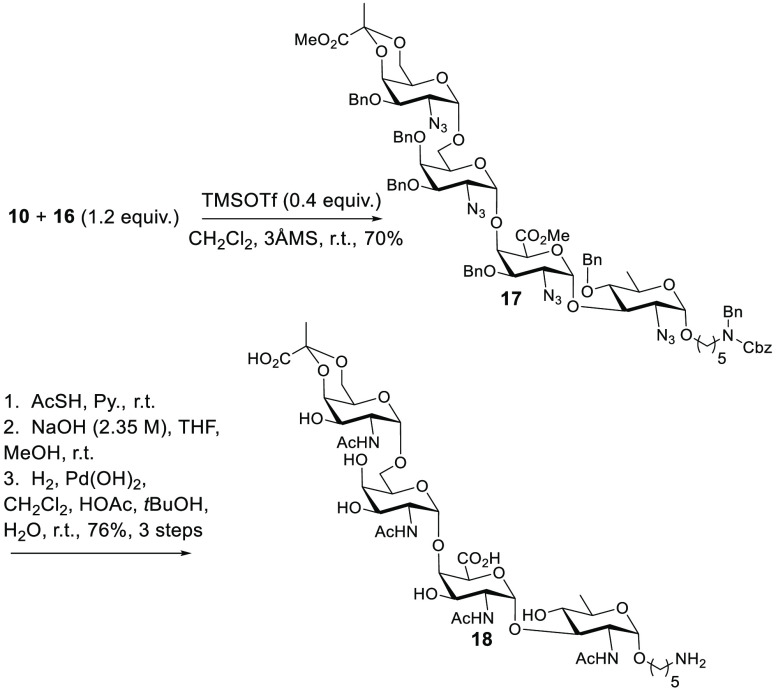
Final Approach to the Target Tetrasaccharide

With fully protected **17** in hand, conversion of azides, hydrolysis of methyl esters, and removal of benzyl protecting groups remained. Thus, treatment with thioacetic acid in pyridine over a period of 80 h resulted in reduction of azides and acylation to the four acetamido groups in the final product. Subsequent hydrolysis of methyl esters (NaOH, MeOH, and THF) and hydrogenolysis of benzyl groups [H_2_ and Pd(OH)_2_] resulted in final product **18**, the linker-attached monomer of **1**. This synthesis proceeded in a total of 35 steps from commercially available starting materials and a longest linear sequence of 23 steps starting from d-glucosamine. NMR of the final product (^1^H, ^13^C, ^13^C-APT, COSY, HSQC, HMBC, and HOHAHA) as well as HRMS helped confirm the structure.

While differences were seen upon comparison of our spectra with those of the CPS originally characterized by Kenyon et al.,^16^ two important points deserve mention. (1) Our product bears a linker, which represents a substantial perturbation of the original structure. (2) While Kenyon et al. do not report on any secondary structure associated with the original CPS, secondary structure would be expected to perturb the appearance of an NMR spectrum relative to a segment with a short chain length. The tetrasaccharide that we have prepared is necessarily devoid of secondary structure due to its short chain length. Due to the regiochemical reliability of procedures such as benzylidene walking [**5** → **6** (Scheme 2) and in the generation of known compound **13** (Scheme 4)], primary alcohol oxidation to carboxylic acid using TEMPO/PhI(OAc)_2_ followed by Pinnick oxidation [**9** → **10** (Scheme 3)],^28^ and the stereochemical verifiability and reliability of pyruvate ketal installation under equilibrating conditions [**14** → **15** (Scheme 4)],^17^ we have high confidence in the structural assignment for **18**. In addition to this, one-dimensional and two-dimensional (2D) NMR analysis assisted us in identifying critical HMBC correlations in **18**, including the following: (1) between linker CH_2_-O ^1^H signals centered at ∼3.71 and ∼3.91 ppm and the QuiNAc anomeric carbon at 96.98 ppm as well as between the QuiNAc anomeric proton at 4.98 ppm and the linker CH_2_-O ^13^C signal at 68.00 ppm, (2) between the GalNAcA anomeric proton at 5.52 ppm and the QuiNAc C3 carbon at 79.20 ppm, and (3) between the GalNAcA C4 proton at 4.58 ppm and the GalNAc anomeric carbon at 99.01 ppm. This accounts for three of four linkages, with the fourth linkage (1 → 6 linkage between PyrGalNAc and GalNAc) being harder to analyze at the stage of product **18** due to substantial signal overlap between these subunits. Nevertheless, this linkage was established with a high degree of confidence from a known set of precursors [**13** and **2** (Scheme 4)] to establish 1,2-*cis* stereochemistry unambiguously as could be ascertained easily with ^13^C spectra of **14**. Also noteworthy is the fact that all anomeric carbons of final product **18** appear at chemical shifts of <100 ppm, affirming that 1,2-*cis* stereochemistry has been established at all four of the glycosidic linkages. Thus, the expected stereochemical and regiochemical outcomes of key transformations in the synthesis of **18** are corroborated by 2D NMR data.

In conclusion, we have synthesized the assigned^16^ KL4-associated tetrasaccharide repeating CPS subunit of *A. baumannii* D78 with a longest linear sequence of 23 steps. Especially noteworthy with this synthesis were the establishment of the glycosidic linkage between the linker and QuiNAc using dilute conditions in 1,4-dioxane and a convergent [2+2] glycosylation to establish the fully protected tetrasaccharide banking on the low reactivity of acceptor **10**. Additional efforts toward the synthesis of *A. baumannii* cell-surface-associated glycans are underway and will be reported in due course.

## Supporting Information Available

Experimental procedures, characterization data, and ^1^H, ^13^C, and 2D NMR spectra (PDF)
