# Supplementary material for: Total Synthesis of an All-1,2-cis-Linked Repeating Unit from the Acinetobacter baumannii D78 Capsular Polysaccharide
Source: Org Lett. 2022 May 6;24(19):3461–5. doi: 10.1021/acs.orglett.2c01034 (PMC9127968; doi:10.1021/acs.orglett.2c01034)
Supplement: Supplementary file 1 — ol2c01034_si_001.pdf [file ol2c01034_si_001.pdf]

Supplemental Information

Total Synthesis of an All-1,2-*cis*-Linked Repeating Unit from the *Acinetobacter baumannii* D78 Capsular Polysaccharide

Dancan K. Njeri and Justin R. Ragains

Department of Chemistry

Louisiana State University

232 Choppin Hall

Baton Rouge, LA 70803

Table of Contents

General Information	S3-4
Synthesis of QuiNAc Precursor Donor 3	S5-10
Synthesis of QuiNAc-Linker Portion 8	S10-15
Synthesis of GalNAc Precursor Acceptor 13	S15-19
Synthesis of GalNAcA/GalNAc/Pyr-GalNAc Donor 2	S19-20
Synthesis of Disaccharide Acceptor 10	S20-24
Failed [3+1] Approach	S25-27
Synthesis of Disaccharide Donor 16	S27-35
[2+2] Glycosylation, Deprotection to Target Tetrasaccharide	S35-41
References	S42
NMR Spectra	S43-191

General information.

Solvents and reagents used were obtained from commercial vendors (Sigma Aldrich, Acros Organics, TCI, etc). 1,4 dioxane used in glycosylation was dried by distilling from sodium benzophenone ketyl. Dichloromethane used in glycosylation was freshly obtained from a solvent purification system (PureSolv 400-5).

Mixtures of glycosyl donor and glycosyl acceptor were co-evaporated with toluene at least two times then placed under high vacuum (if mixture had potential of bumping inside the reaction flask, it was briefly placed under high vacuum). Powdered 3 Å Molecular sieves used in the final 2+2 glycosylation were activated by flame drying under vacuum, allowing to cool while capped under vacuum, and then backfilled with nitrogen gas just before they were poured into the reaction vessel (mass of 3 Å MS used was determined by difference). Room temperature was 18 °C most of the time.

Column chromatography was performed using silica gel (60 Å) from SiliCyle. Thin layer chromatography (TLC) was performed using silica gel (60 Å) with F254 indicator on aluminum sheets purchased from Merck. Prep TLC was performed using "Analtech silica gel GF UV254 20*20 1000 micron" from Miles Scientific. A 3/1 dichloromethane (DCM)/Ethyl acetate (EtOAc) solvent mixture stock solution is referred to as 'DE' throughout this document (especially important in TLC and column chromatography).

A hand-held UV lamp was used to visualize compounds on TLC and Prep TLC. Staining of compounds on TLC was performed by immersing developed TLC in *p*-anisaldehyde stain then drying excess stain on both sides of the TLC plate using a paper towel then heating the aluminum side of the TLC plate using a heat gun until colored spots appear on the side with silica gel.

^{1}H NMR, ^{13}C NMR (DEPT and APT), COSY, HMBC, HSQC, TOCSY and HOHAHA experiments were performed using a Bruker AV-400, Bruker AV-500 NMR or a Bruker AVANCE

Neo 700 MHz spectrometer. Deuterated solvents used for NMR were obtained from Cambridge Isotope Labs ($CDCl_3$), Acros organics (*d*-MeOD), and Sigma Aldrich (D_2O). Structural assignments were made with additional information from gCOSY, gHSQC, and gHMBC experiments.

HRMS was performed using an Agilent 6210 electrospray time-of-flight mass spectrometer. Optical rotation data were obtained using a JASCO P-2000 instrument.

Synthesis of QuiNAc Precursor Donor 3

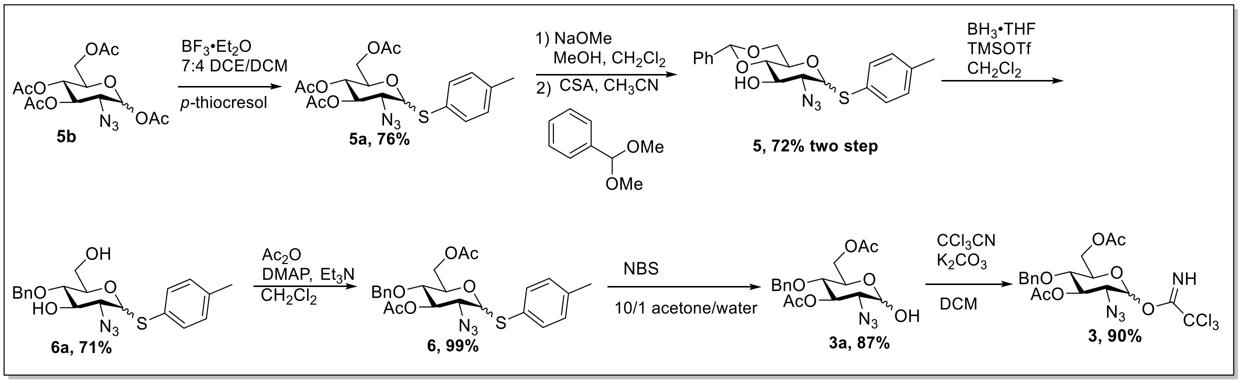

Synthesis of **5a**

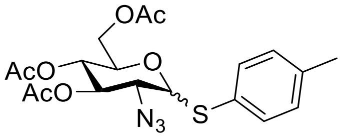

To a RBF charged with a stir bar, 2-azido-2-deoxy-1,3,4,6-tetra-*O*-acetylglucose **5b**[1] (24.08g, 64.50 mmol) and *p*-thiocresol (18.02g, 145.1 mmol) were dissolved in 7/4 dichloroethane/ dichloromethane (275ml). $BF_3 \cdot Et_2O$ (19.0 ml, 154 mmol) was added dropwise, then a reflux condenser was attached. The resulting setup was then warmed to reflux by heating using an oil bath. After 1 hour, TLC indicated complete consumption of starting material **5b** and formation of product **5a**. The reaction mixture was washed using 200 ml water followed by a wash with 5% NaOH (300 ml). The organic phase was separated and dried using Na_2SO_4 then concentrated using a rotary evaporator to give crude material that was purified by flash chromatography (20-40% DE/hexanes(DE= 3/1 DCM/EtOAc)) to give 21.46 g of 4-methylphenyl 3,4,6-tri-*O*-acetyl-2-azido-2-deoxy-1-thio-α/β-D-glucopyranoside **5a** (yellowish foam, 76% yield). Characterization of this compound matched that reported in the literature.[2]

Synthesis of **5**

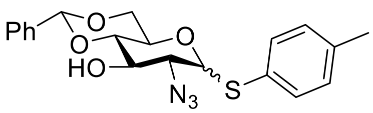

To **5a** (21.46 g, 49.06 mmol) dissolved in (3/1) MeOH/DCM (165 ml), a 5M NaOMe solution in MeOH (16 ml) was added dropwise while stirring. After 20 minutes, TLC indicated complete formation of a more polar compound. The reaction mixture was neutralized by addition of Dowex resin (50WX8 200-400 MESH). The mixture was filtered, then the filtrate was concentrated to give crude tetraol**,** which was then co-evaporated with 5ml toluene two times then placed under high vacuum for 30 minutes. Crude tetraol was then dissolved in CH_3CN (250 ml). Benzaldehyde dimethyl acetal (70ml, 478 mmol) and camphor sulfuric acid (1.98g, 8.52 mmol), were then added into the flask, respectively. The reaction was then heated to reflux with stirring under a nitrogen atmosphere. After 3 hrs, TLC indicated total consumption of tetraol. The reaction was allowed to cool to room temperature, then Et_3N (1.0 ml) was added dropwise to quench the reaction. The mixture was concentrated using a rotary evaporator followed by purification using flash chromatography (15-30% DE/hexanes(DE= 3/1 DCM/EtOAc)) to give 14.19g of **5** (White solid, 72% yield).[3] **^{1}H NMR (500 MHz, $CDCl_3$) δ** 7.56 – 7.33 (m, 17H), 7.16 (m, 5H), 5.57-5.49 (m, 3H), 4.50 (s, 1H), 4.48 (s, 1H), 4.45 – 4.35 (m, 3H), 4.24 (dd, *J* = 10.3, 5.0 Hz, 1H), 4.09 (t, *J* = 9.6 Hz, 1H), 3.92 (dd, *J* = 10.0, 5.6 Hz, 1H), 3.81 – 3.73 (m, 4H), 3.58 (t, *J* = 9.4 Hz, 1H), 3.50 – 3.42 (m, 3H), 3.36-3.30 (m, 2H), 2.70 (s, 1H), 2.67 (s, 1H), 2.37 (s, 3H), 2.34 (s, 3H); **^{13}C NMR (126 MHz, $CDCl_3$) δ** 139.2, 134.3, 133.2, 130.0, 130.0, 129.4, 128.4, 128.4, 126.8, 126.3, 126.2, 102.2, 102.0, 88.1, 86.9, 81.7, 80.2, 74.2, 70.8, 70.3, 68.5, 65.1, 64.0, 63.3, 21.2; **HRMS (ESI) m/z: [M + Cl]-** Calcd for $C_{20}H_{21}N_3O_4SCl$ 434.0947; found 434.0950. **$[\alpha]_D^{25}$ =** +47.8° (*c* 0.18, CH_2Cl_2). **IR (cm^{-1})** 3453, 2923, 2109, 1493, 1381, 1275, 1094, 973, 809, 761, 699.

Synthesis of diol **6a.**

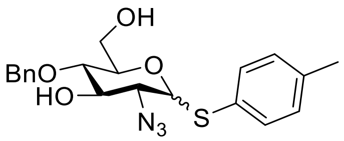

To a stirred solution of **5** (1.16g, 2.90 mmol) in dry dichloromethane (20 mL) under nitrogen atmosphere at 18 °C, was added 1M BH_3·THF (5 ml, 5 mmol, freshly opened) followed by TMSOTf (700.0 µL, 3.857 mmol). After 13 hrs, the reaction was quenched by addition of Et_3N (0.6 ml). The reaction mixture was then transferred to an ice bath and further quenched by dropwise addition of MeOH (10ml) after which gas evolution ceased. . The mixture was concentrated under vacuum then re-dissolved in 100 ml DCM. The organic solution was washed once with water (50 ml), dried using Na_2SO_4, then concentrated to give crude material that was purified by flash chromatography (20-40% EtOAc/hexanes to give 0.83 g of **6a** (White solid, 71% yield).[4]

^{1}H NMR (500 MHz, $CDCl_3$) δ 7.46-7.29 (m, 9H), 7.17 – 7.10 (m, 2H), 5.48 (d, *J* = 5.5 Hz, 1H), 4.79 (s, 2H), 4.73 (d, *J* = 2.4 Hz, 1H), 4.42 (d, *J* = 10.2 Hz, 1H), 4.27-4.22 (m, 1H), 4.00 – 3.89 (m, 2H), 3.85 – 3.70 (m, 4H), 3.61 (t, *J* = 9.1 Hz, 1H), 3.56-3.51 (m, 1H), 3.41 (t, *J* = 9.2 Hz, 1H), 3.36-3.32 (m, 1H), 3.25-3.20 (m, 1H), 2.55 – 2.41 (m, 2H), 2.36 (s, 3H), 2.33 (s, 3H); **^{13}C NMR (126 MHz, $CDCl_3$) δ** 138.4, 137.9, 134.0, 133.1, 130.0, 129.9, 129.3, 128.8, 128.7, 128.3, 128.1, 128.1, 87.4, 86.3, 77.8, 75.0, 73.7, 71.9, 64.0, 61.6, 21.2. **HRMS (ESI) m/z: [M + Na]+** Calcd for $C_{20}H_{23}N_3O_4SNa$ 424.1302; found 424.1304. **$[\alpha]_D^{25}$ =** +24.5° (*c* 0.25, CH_2Cl_2). **IR (cm^{-1})** 3408, 2921, 2108, 1493, 1076, 986, 812, 699, 608.

Synthesis of thioglucoside **6**

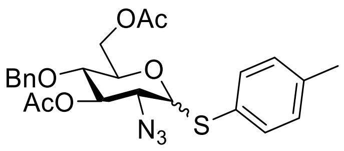

Diol **6a** (1.87g, 4.66 mmol) and 4-dimethylamino pyridine (DMAP, 92.5mg, 0.757 mmol) were dissolved in 25 ml dichloromethane. 3.5 ml of Et_3N was then added followed by addition of acetic anhydride (3.2ml, 34 mmol) under nitrogen atmosphere. The reaction was allowed to stir at room temperature (18 °C). After 3 hrs, TLC showed total consumption of 2d and formation of a new spot and stirring was concluded. The organic phase was then washed with 1M HCl (60 ml) once followed by wash with 50 ml sat. $NaHCO_3$ once. The washed organic phase was dried using $NaSO_4$ then concentrated to give crude material that was purified by flash chromatography (35% DE/hexanes (DE=3/1 DCM/EtOAc)) to give 2.24g of **6** mixture of *alpha* and *beta* (white foam, 99% yield).[5]

^{1}H NMR (500 MHz, $CDCl_3$) δ 7.51 – 7.45 (m, 3H), 7.42 – 7.08 (m, 26H), 5.52 (d, *J* = 5.5 Hz, 1H), 5.45 (dd, *J* = 10.6, 9.0 Hz, 1H), 5.16 (t, *J* = 9.5 Hz, 2H), 4.64 – 4.50 (m, 6H), 4.46 – 4.34 (m, 4H), 4.31 – 4.22 (m, 2H), 4.19 (dd, *J* = 12.0, 5.0 Hz, 2H), 3.93 (dd, *J* = 10.6, 5.5 Hz, 1H), 3.62 – 3.54 (m, 3H), 3.47 (t, *J* = 9.5 Hz, 2H), 3.27 (t, *J* = 10.0 Hz, 2H), 2.36 (s, 5H), 2.33 (s, 3H), 2.08 (s, 7H), 2.03 (s, 4H), 2.02 (s, 2H). **^{13}C NMR (126 MHz, $CDCl_3$)** δ 170.5, 169.7, 139.0, 137.0, 134.4, 133.0, 130.0, 129.8, 128.6, 128.6, 128.3, 128.2, 128.1, 128.0, 126.9, 87.1, 86.0, 77.2, 76.3, 76.0, 75.6, 74.7, 74.6, 73.4, 69.6, 63.2, 62.8, 62.7, 62.3, 21.2, 21.1, 20.9, 20.9. **HRMS (ESI) m/z: [M + H]+** Calcd for $C_{24}H_{28}N_3O_6S$ 486.1693; found 486.1696. **$[\alpha]_D^{25}$ =** +40.3° (*c* 0.007, CH_2Cl_2). **IR (cm^{-1})** 2923, 2107, 1744, 1494, 1454, 1365, 1217, 1089, 1032, 909, 811, 751, 700, 494.

Synthesis of lactol **3a.**

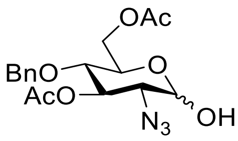

To a stirred solution of **6** (2.24g, 4.61 mmol) in 10/1 acetone /water (44 ml), *N*-bromosuccinamide (1.5g, 14 mmol) was added and the reaction allowed to stir under Nitrogen atmosphere at room temperature. After 1 hour, TLC indicated complete consumption of one of the anomers in **3b**. Additional NBS (1.5g, 8.4 mmol) was added. After 2.5 hrs total, TLC indicated total consumption of both anomers of **6**. The reaction mixture was concentrated under vacuum then redissolved in 50 ml DCM. The organic solution was washed once with water (70 ml), dried using Na_2SO_4, then concentrated to give crude material that was purified by flash chromatography (30-40% EtOAc/hexanes) to give 1.52g of lactol **3a** (white foam, 87% yield).

^{1}H NMR (500 MHz, $CDCl_3$) δ 7.40 – 7.19 (m, 9H), 5.61 (dd, *J* = 10.6, 9.0 Hz, 1H), 5.33 (t, *J* = 3.2 Hz, 1H), 5.09 (dd, *J* = 10.4, 8.9 Hz, 1H), 4.70 (d, *J* = 8.0 Hz, 1H), 4.66 – 4.51 (m, 4H), 4.36 (m, 2H), 4.25 – 4.14 (m, 3H), 4.05 (d, *J* = 3.5 Hz, 1H), 3.63 – 3.56 (m, 2H), 3.36 (dd, *J* = 10.4, 7.9 Hz, 1H), 3.20 (dd, *J* = 10.6, 3.4 Hz, 1H), 2.06 (m, 10H). **^{13}C NMR (126 MHz, $CDCl_3$)** δ 171.0, 170.1, 170.0, 137.2, 137.0, 128.6, 128.6, 128.3, 128.2, 128.1, 128.0, 96.0, 92.2, 76.1, 75.7, 74.7, 74.6, 74.2, 73.0, 72.2, 68.6, 65.2, 62.8, 62.7, 61.9, 20.9, 20.9, 20.9. **HRMS (ESI) m/z: [M + K]+** Calcd for $C_{17}H_{21}N_3O_7K$ 418.1011; found 418.1011. **$[\alpha]_D^{25}$ =** -84.2° (*c* 0.5, CH_2Cl_2). **IR (cm^{-1})** 3433, 2924, 2109, 1741, 1366, 1224, 1073, 1035, 751, 700.

Synthesis of Trichloroacetimidate (TCAI) donor **3**

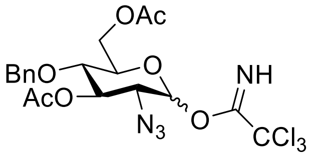

Lactol **3a** (1.52g, 4.01 mmol) dissolved in DCM (25ml) and trichloroacetonitrile (15ml) in a suspension with potassium carbonate (7.08g, 51.2 mmol). The reaction was allowed to stir at room temperature under nitrogen atmosphere. After 16h, the reaction was filtered, concentrated, co-evaporated with dry DCM (8.0 ml) three times and then placed under high vacuum overnight to give 1.9 g of TCAI donor **3** (white foam, 90% yield).

^{1}H NMR (400 MHz, $CDCl_3$) δ 8.80 (s, 1H), 8.77 (s, 1H), 7.42 – 7.22 (m, 20H), 6.45 (d, *J* = 3.6 Hz, 1H), 5.71 (d, *J* = 8.3 Hz, 1H), 5.63 (dd, *J* = 10.6, 9.1 Hz, 1H), 5.19 (dd, *J* = 10.0, 8.6 Hz, 1H), 4.66 – 4.53 (m, 5H), 4.39-4.31 (m, 2H), 4.28-4.21 (m, 2H), 4.18 – 4.10 (m, 1H), 3.77 – 3.65 (m, 5H), 3.59 (dd, *J* = 10.6, 3.6 Hz, 1H), 2.09 (s, 3H), 2.08 (s, 3H), 2.05 (s, 3H), 2.05 (s, 3H). **^{13}C NMR (126 MHz, $CDCl_3$) δ** 170.5, 169.6, 160.8, 160.7, 136.9, 136.9, 128.7, 128.7, 128.4, 128.3, 128.2, 128.1, 96.5, 94.5, 90.6, 90.3, 75.4, 75.1, 74.9, 74.6, 74.1, 73.8, 72.1, 71.4, 63.7, 62.2, 62.2, 61.1, 20.9, 20.8, 20.8. **HRMS (ESI) m/z: [M + Na]+** Calcd for $C_{19}H_{21}Cl_3N_4O_7Na$ 545.0368; found 545.0373. **$[\alpha]_D^{25}$ =** -87.0° (*c* 0.25, CH_2Cl_2). **IR (cm^{-1})** 3338, 2923, 2111, 1742, 1676, 1453, 1365, 1218, 1070, 1029, 796, 751, 700.

Synthesis of QuiNAc-Linker Portion 8

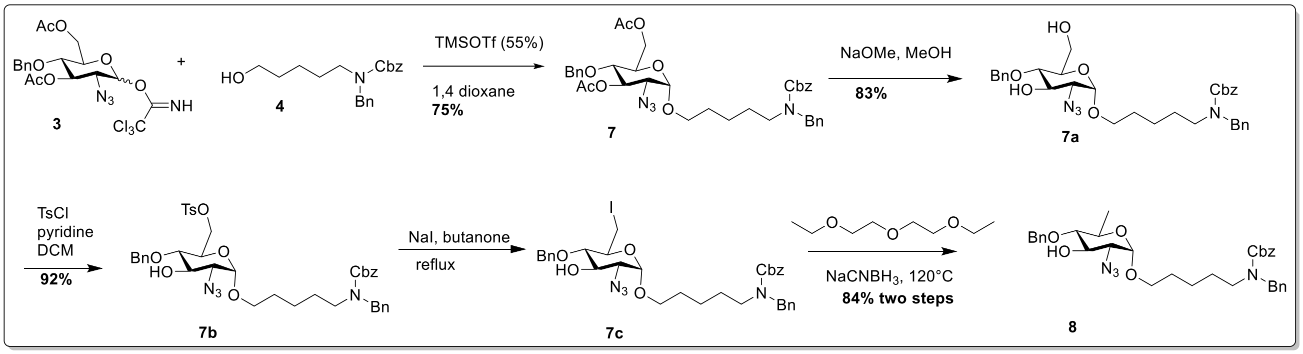

Synthesis of **7**

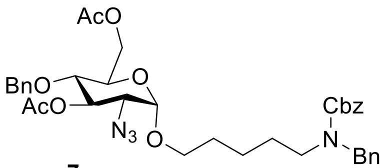

7

Donor **3** (1.90g, 3.63 mmol) and *N*-(benzyl)benzyloxycarbonylaminopentanol acceptor **4**[6] (2.41g, 7.36 mmol) in an R.B.F were co-evaporated thrice with 2 ml toluene. The RBF was charged with an oven-dried magnetic stir bar then capped with a rubber septum. The RBF was then purged and backfilled with nitrogen gas (thrice). Freshly dried 1,4 dioxane (182.0 ml) was then added via a syringe under nitrogen atmosphere followed by dropwise addition of TMSOTf activator (362.0µL, 1.995 mmol). The reaction was allowed to stir under nitrogen atmosphere at room temperature (18 °C). After 42 hours, the reaction was quenched by dropwise addition of Et_3N (0.2ml), then concentrated using a rotary evaporator to give crude material that was diluted with 100 ml DCM. The new organic solution was washed once with 30 ml 1M NaOH (shaken vigorously to get rid of trichloroacetamide side product). The organic phase was dried using Na_2SO_4, then concentrated to give crude material that was purified by flash chromatography (25-30% EtOAc/hexanes) to give 1.8876 g of **7** (slightly yellowish oil, 75% yield). **^{1}H NMR (500 MHz, $CDCl_3$)** δ 7.47 – 7.09 (m, 15H), 5.60 (t, *J* = 9.8 Hz, 1H), 5.17 (m, 2H), 4.89 (d, *J* = 14.0 Hz, 1H), 4.62 (d, *J* = 11.1 Hz, 1H), 4.56 (d, *J* = 11.2 Hz, 1H), 4.53 – 4.45 (m, 2H), 4.36 - 4.22 (m, 2H), 3.91 (m, 1H), 3.72-3.60(m, 1H), 3.57 (t, *J* = 9.5 Hz, 1H), 3.47 – 3.33 (m, 1H), 3.31 – 3.16 (m, 2H), 3.07 (dd, *J* = 10.6, 3.5 Hz, 1H), 2.06 (s, 3H), 2.05 (s, 3H), 1.67 – 1.45 (m, 4H), 1.41 – 1.25 (m, 2H**); ^{13}C NMR (126 MHz, $CDCl_3$)** δ 170.6, 169.8, 156.5, 156.2, 138.0, 137.3, 128.6, 128.6, 128.5, 128.2, 128.1, 128.0, 127.9, 127.9, 127.9, 127.3, 98.0, 76.2, 74.8, 72.2, 68.8, 68.4, 67.2, 67.1, 62.7, 61.2, 50.6, 50.3, 47.1, 46.2, 29.0, 27.9, 27.5, 23.4, 21.0, 20.9. **HRMS (ESI) m/z: [M + H]+** Calcd for $C_{37}H_{45}N_4O_9$ 689.3181; found 689.3180; **$[\alpha]_D^{25}$ =** -23.6° (*c* 1, CH_2Cl_2). **IR (cm^{-1})** 2925, 2106, 1742, 1694, 1453, 1422, 1364, 1220, 1072, 1030, 735, 699, 459.

Synthesis of **7a**

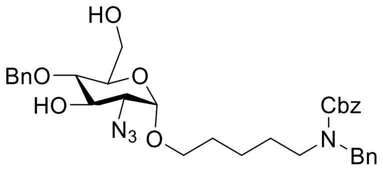

To a stirred solution of **7** (1.8876g, 2.7405 mmol) in 8/1 MeOH/DCM (22.5ml), 5M NaOMe (1.88 ml, 9.4 mmol) was added. The reaction was allowed to stir under nitrogen atmosphere at room temperature. After 21h, the reaction was quenched by portionwise addition of 4.94g Dowex resin (50WX8 200-400 MESH). The mixture was filtered, then the filtrate was concentrated using rotary evaporator to give a crude material that was purified by flash chromatography (35-60% EtOAc/hexanes) to give 1.3777g of **7a** (yellow oil, 83% yield).

^{1}H NMR (500 MHz, $CDCl_3$) δ 7.51 – 7.02 (m, 15H), 5.17 (m, 2H), 4.88 – 4.72 (m, 3H), 4.49 (m, 2H), 4.10 (t, *J* = 10.0 Hz, 1H), 3.92 – 3.15 (m, 8H), 3.11 (dd, *J* = 10.3, 3.6 Hz, 1H), 2.24 – 1.93 (m, 2H), 1.70 – 1.45 (m, 4H), 1.39 – 1.26 (m, 2H); **^{13}C NMR (126 MHz, $CDCl_3$)** δ 156.3, 138.0, 137.9, 128.7, 128.7, 128.6, 128.5, 128.2, 128.2, 128.1, 127.9, 127.9, 127.3, 127.2, 97.8, 78.2, 75.1, 71.8, 71.0, 68.1, 67.2, 63.1, 61.8, 50.5, 50.3, 47.1, 46.1, 29.0, 27.9, 27.3, 23.3. **HRMS (ESI) m/z: [M + Na]+ Calcd for** $C_{33}H_{40}N_4O_7Na$ 627.2789; found 627.2790; **$[\alpha]_D^{25}$ =** -34.3° (*c* 1, CH_2Cl_2). **IR (cm^{-1})** 3416, 2924, 2104, 1676, 1453, 1423, 1361, 1230, 1072, 1028, 734, 698.

Synthesis of **7b**

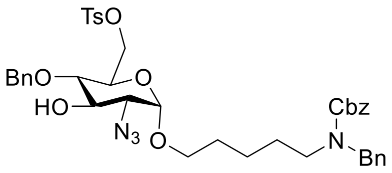

To a stirred solution of **7a** (1.24g, 2.05 mmol) in dichloromethane (20ml), *p*-toluenesulfonyl chloride (1.49g, 7.82 mmol) was added, followed by addition of dry pyridine (20ml). The reaction was allowed to run under nitrogen atmosphere at room temperature (18 °C). After 14hrs, the reaction mixture was concentrated, then diluted with 50ml DCM. The organic phase was washed once with 1M HCl (50ml), followed by one more wash with 50 ml aq. saturated $NaHCO_3$. The organic phase was dried using Na_2SO_4 and then concentrated to give crude material which was purified by flash chromatography (50-60% DE/hexanes (DE= 3/1 DCM/EtOAc)) to give 1.43g of **7b** (sticky colorless gum, 92% yield).

^{1}H NMR (500 MHz, $CDCl_3$) δ 7.78 (d, *J* = 8.0 Hz, 2H), 7.43 – 7.07 (m, 17H), 5.20-5.08 (m, 2H), 4.79-4.71 (m, 2H), 4.57 (d, *J* = 11.1 Hz, 1H), 4.51-4.43 (m, 2H), 4.30-4.16 (m, 2H), 4.05 (t, *J* = 9.9 Hz, 1H), 3.79 – 3.72 (m, 1H), 3.61 – 3.49 (m, 1H), 3.40 (t, *J* = 9.3 Hz, 1H), 3.37 – 3.12 (m, 4H), 3.07 (dd, *J* = 10.4, 3.5 Hz, 1H), 2.46 (s, 1H), 2.41 (s, 3H), 1.59 – 1.41 (m, 3H), 1.34 – 1.19 (m, 2H).**^{13}C NMR (126 MHz, $CDCl_3$) δ** 156.8, 156.4, 145.2, 138.1, 137.8, 137.0, 133.0, 130.1, 128.9, 128.7, 128.7, 128.4, 128.2, 128.2, 128.1, 128.0, 127.5, 97.8, 77.9, 75.3, 72.2, 68.9, 68.7, 68.5, 67.4, 63.0, 50.7, 50.5, 47.2, 46.30, 29.2, 28.0, 27.6, 23.5, 21.8. **HRMS (ESI) m/z: [M + H]+** Calcd for $C_{40}H_{47}N_4O_9S$ 759.3058; found 759.3057; **$[\alpha]_D^{25}$ =** -43.4° (*c* 1, CH_2Cl_2). **IR (cm^{-1})** 3408, 2922, 2105, 1677, 1598, 1473, 1453, 1361, 1175, 1072, 814, 734, 698, 669, 553.

Synthesis of **7c**

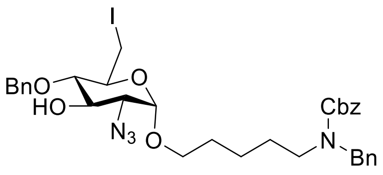

To a R.B.F charged with a magnetic stir bar, **7b** (1.43g, 1.88 mmol) was dissolved in 25ml 2-butanone. NaI (1.69g, 11.3 mmol) was added followed by attachment of a reflux condenser. The reaction mixture was refluxed (by heating using an oil bath). After 6 hrs, TLC indicated total consumption of **7b**. The reaction mixture was concentrated using a rotary evaporater and diluted with 100ml DCM. The organic phase was washed once with 1M aq. sodium thiosulfate (50 ml), dried using Na_2SO_4, and finally concentrated using a rotary evaporator to give crude material (1.57 g) that was used in the next step without further purification (**7c,** colorless oil).

^{1}H NMR (400 MHz, $CDCl_3$) δ 7.41 – 7.12 (m, 15H), 5.16 (m, 2H), 4.91 (d, *J* = 11.2 Hz, 1H), 4.87 – 4.79 (m, 1H), 4.75 (d, *J* = 11.2 Hz, 1H), 4.48 (m, 2H), 4.12 (m, 1H), 3.71 (m, 1H), 3.53 – 3.11 (m, 9H), 1.70 – 1.42 (m, 4H), 1.42 – 1.20 (m, 2H); **^{13}C NMR (101 MHz, $CDCl_3$)** δ 156.9, 137.9, 128.7, 128.7, 128.6, 128.5, 128.2, 128.1, 128.0, 127.9, 127.3, 97.7, 82.3, 75.4, 75.2, 71.8, 69.6, 68.3, 67.3, 63.2, 50.6, 47.2, 46.6, 29.1, 27.9, 23.4. **HRMS (ESI) m/z: [M + H]+** Calcd for $C_{33}H_{40}IN_4O_6$ 715.1987; found 715.1994; **$[\alpha]_D^{25}$ =** -52.8° (*c* 1, CH_2Cl_2).**IR (cm^{-1})** 3405, 2922, 2105, 1676, 1422, 1362, 1225, 1040, 734, 698.

Synthesis of **8**

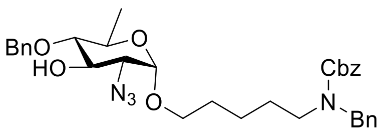

To a stirred solution of **7c** (crude material from above procedure (1.57 g)) in diethylene glycol diethyl ether (11.5ml) in an R.B.F, $NaCNBH_3$(2.0g, 32 mmol) was added. A reflux condenser was

attached, and the reaction was then allowed to proceed at 120 °C using an oil bath under nitrogen atmosphere. After 8 hours, the reaction was allowed to cool to room temperature, then diluted with 50ml EtOAc. The organic solution was washed once with 50ml water, then concentrated using a rotary evaporator to remove EtOAc. **Note**: Due to the high boiling point of diethylene glycol diethyl ether, removal with a rotary evaporator was difficult. The mixture was transferred into a 100ml beaker, and a low stream of pressurized air was blown over it overnight inside the fume hood. On the following morning, most of the visible diethylene glycol diethyl ether had been removed to give a thick oil (product). The oil was then purified by flash chromatography (36-45 % DE/ hexanes (DE= 3/1 DCM/EtOAc)) to give 0.9344 g of **8** (thick, colorless oil, 84% two step yield from **7b**).

^{1}H NMR (500 MHz, $CDCl_3$) δ 7.39 – 7.11 (m, 15H), 5.16 (m, 2H), 4.82-4.70 (m, 3H), 4.48 (m, 2H), 4.05 (t, *J* = 9.7 Hz, 1H), 3.74 (m, 1H), 3.60 (m, 1H), 3.42 – 3.30 (m, 1H), 3.22 (m 2H) 3.12 (dd, *J* = 10.4, 3.6 Hz, 1H), 3.06 (t, *J* = 9.1 Hz, 1H), 2.58 (d, *J* = 3.5 Hz, 1H), 1.64-1.44 (m, 4H), 1.39 – 1.23 (m, 2H), 1.28 (d, *J* = 6.3 Hz, 3H); **^{13}C NMR (126 MHz, $CDCl_3$)** δ 156.8, 156.2, 138.1, 137.9, 136.9, 128.7, 128.6, 128.6, 128.5, 128.1, 128.0, 127.9, 127.9, 127.3, 97.6, 84.6, 75.3, 71.7, 67.9, 67.2, 66.8, 63.3, 50.6, 50.3, 47.1, 46.2, 29.1, 27.9, 27.5, 23.4, 18.0; **HRMS (ESI) m/z: [M + Na]+** Calcd for $C_{33}H_{40}N_4O_6Na$ 611.2840; found 611.2837; **$[\alpha]_D^{25}$ =** +33.1° (*c* 1, CH_2Cl_2). **IR (cm^{-1})** 3434, 3031, 2931, 2104, 1679, 1496, 1474, 1227, 1042, 734, 698.

Synthesis of GalNAc Precursor Acceptor 13

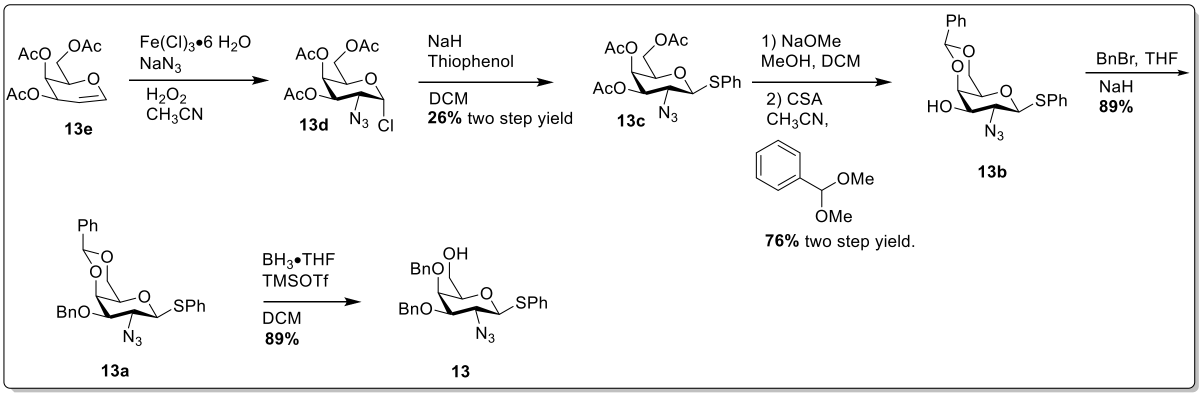

Synthesis of **13c**

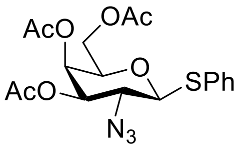

To a stirred solution of triacetyl galactal **13e** (11.83 g, 43.45 mmol) in acetonitrile (125 ml) at -20 °C, $FeCl_3 \cdot 6H_2O$ (14.73g, 54.50 mmol) was added followed by portionwise addition of NaN_3 (3.2851g, 50.532 mmol) over a period of two minutes. The RBF was then capped and allowed to stir under nitrogen atmosphere before dropwise addition of 30% H_2O_2 (5.0ml) over a period of one minute. After 2h, an additional 2ml of 30% H_2O_2 was added dropwise over a period of one minute and the reaction was allowed to warm to 10 °C. Five hours later, the TLC indicated total consumption of starting material. The reaction mixture was diluted with 500ml DCM, washed once with 600 ml water and once with 200 ml sat. $NaHCO_3$(aq). The organic phase was dried using Na_2SO_4 and concentrated to give crude 2-azido-1-chloro-2-deoxy-3,4,6 triacetyl-α-D-galactose **13d** (12.36 g).

Crude **13d** (12.36 g) was then dissolved in 30 ml DCM in an RBF. The flask was placed in an ice bath followed by addition of thiophenol (6.0 ml, 59 mmol). 60% NaH suspended in mineral oil (5g, 125 mmol) was then added portionwise over a period of two minutes and the reaction was allowed to stir under nitrogen atmosphere while warming to room temperature. After 15 hours, the reaction

mixture was transferred to an ice bath and quenched by dropwise addition of water (20 ml) until gas evolution ceased. The mixture was diluted with 200 ml DCM and washed twice with 500 ml water. The organic phase was dried using Na_2SO_4 then concentrated to give a crude material that was purified by flash chromatography (20-25% EtOAc/hexanes) to give 4.84g of **13c** (white solid, 26% two-step yield). Spectroscopic data matched that reported in the literature.[7]

Synthesis of **13b**

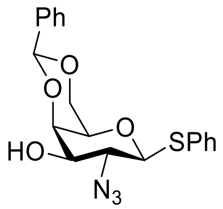

To a stirred solution of **13c** (4.84 g, 11.4 mmol) in 2/1 MeOH/DCM (30ml), a 5M NaOMe solution in MeOH (1 ml) was added dropwise over a period of one minute while stirring. After 30 minutes, TLC indicated total consumption of **13c** to form a single spot. The reaction was quenched by portionwise addition of Dowex resin (50WX8 200-400 MESH) (2.62g). The mixture was then filtered. The filtrate was concentrated and co-evaporated with 2ml toluene twice then placed under high vacuum for 2h to give crude triol.

Triol was dissolved in 50ml acetonitrile by stirring. Benzaldehyde dimethyl acetal (14.0ml, 93.3 mmol) and camphorsulfonic acid (CSA, 0.34g, 1.5 mmol) were then added, respectively. A reflux condenser was attached, and the reaction was heated to 80 °C using an oil bath. After 2h, TLC indicated total consumption of triol. The reaction was allowed to cool to room temperature then quenched by dropwise addition of 0.5 ml Et_3N. The mixture was diluted with 50mL DCM then washed with 50mL water once. The organic phase was dried using Na_2SO_4 then concentrated using a rotary evaporator to give crude material that was purified by flash chromatography (20-

50% EtOAc/hexanes) to provide 3.34g of **13b** (white foam, 76% two-step yield). Spectroscopic data matched that reported in the literature.[8]

Synthesis of **13a**

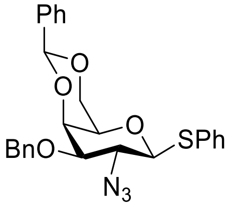

To a stirred solution of **13b** (2.90g, 7.52 mmol) in THF at 0 °C, benzyl bromide (2.24ml, 18.8 mmol) was added followed by portion-wise addition of 60% NaH suspended in mineral oil (0.7524g) over a period of two minutes. The reaction was allowed to stir under nitrogen atmosphere while warming to room temperature. After 16hrs, the reaction was transferred to an ice bath then quenched by dropwise addition of water (12 mL) over a period of 10 minutes until gas evolution stopped. The mixture was diluted with 100ml EtOAc, then washed once with 100ml water. The organic phase was dried using Na_2SO_4 then concentrated to give a crude material that was purified by flash chromatography (15-37% EtOAc/hexanes) to give 3.1811g of **13a** (white foam, 89% yield). Spectroscopic data matched that reported in the literature.[9]

Synthesis of **13**

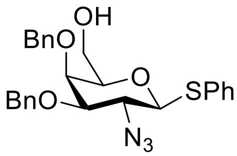

To **13a** (0.496g, 1.04 mmol) in a flame dried round bottom flask charged with a stir bar under nitrogen atmosphere, 4.0 ml of dry DCM (from a solvent purification system) was added.

Immediately thereafter, 1M BH_3·THF (4.0 ml, 4.0 mmol) was added dropwise until effervescence stopped. Finally, TMSOTf (22.5 μL, 0.124 mmol) was added dropwise and the reaction was allowed to stir at room temperature. After three hours, TLC indicated the reaction was complete. The reaction was quenched by dropwise addition of Et_3N (4 drops). The reaction flask was placed in an ice bath then further quenched by dropwise addition of MeOH (10.0 ml) upon which gas evolution ceased. The mixture was concentrated then placed under high vacuum to give a crude material that was further purified by flash chromatography (35-42% EtOAc/hexanes) to give 0.442 g of **13** (white foam, 89% yield). Spectroscopic data matched that reported in the literature[10]

Synthesis of GalNAcA/GalNAc/Pyr-GalNAc Donor 2

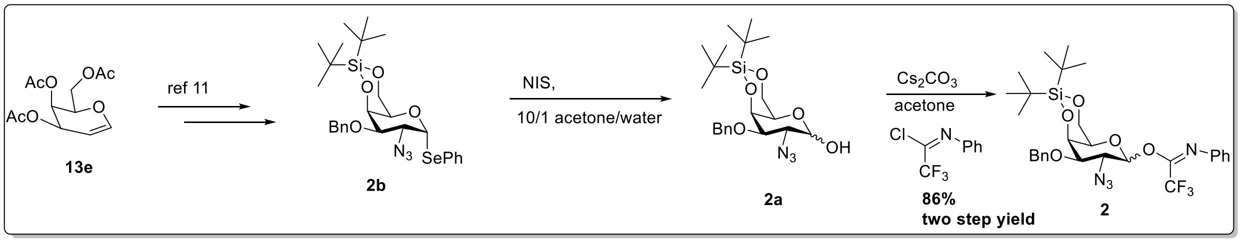

Synthesis of **2**

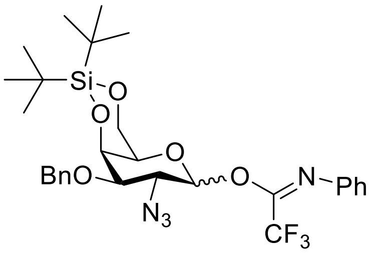

To a stirred solution of **2b**[11] (2.5725g, 4.4766 mmol) in 10/1 acetone/water (30ml), *N*-iodosuccinimide (1.3063g, 5.81 mmol) was added. The reaction was then stirred at room

temperature (18 °C) under nitrogen atmosphere. After 40 minutes, TLC indicated that the reaction was complete. Stirring was discontinued and the reaction was concentrated using a rotary evaporator to give crude material that was dissolved in 20 ml DCM then washed with 10ml 1M sodium thiosulfate. The organic phase was dried using Na_2SO_4 then concentrated to give a crude material that was purified by flash chromatography (15-25% EtOAc/hexanes) to give 1.6780g of lactol **2a.**

To a stirred solution of **2a** (1.6780g, 3.8522) in acetone (25 ml), Cs_2CO_3 (4.03g, 12.4 mmol)) was added followed by dropwise addition of 2,2,2-trifluoro-*N*-phenylacetimidoyl chloride (0.9168 g, 4.417 mmol). The reaction was allowed to run at room temperature (18 °C) under nitrogen atmosphere. Two hours later, TLC indicated that the reaction was complete. Stirring was discontinued and the reaction was concentrated using a rotary evaporator to give material that was dissolved in 100 ml DCM then washed with 50 ml water once. The organic phase was dried using Na_2SO_4 then concentrated to give a crude material that was purified by flash chromatography (10-25% D/Eth/hexanes (D/Eth= 3/1 DCM/Et_2O)) to give 1.4026 g of **2 *alpha*** and 0.9334g of **2 *beta*** (86% two-step yield, both were white solids with yellowish tint). Spectral data matched that reported in the literature.[12]

Synthesis of Disaccharide Acceptor 10

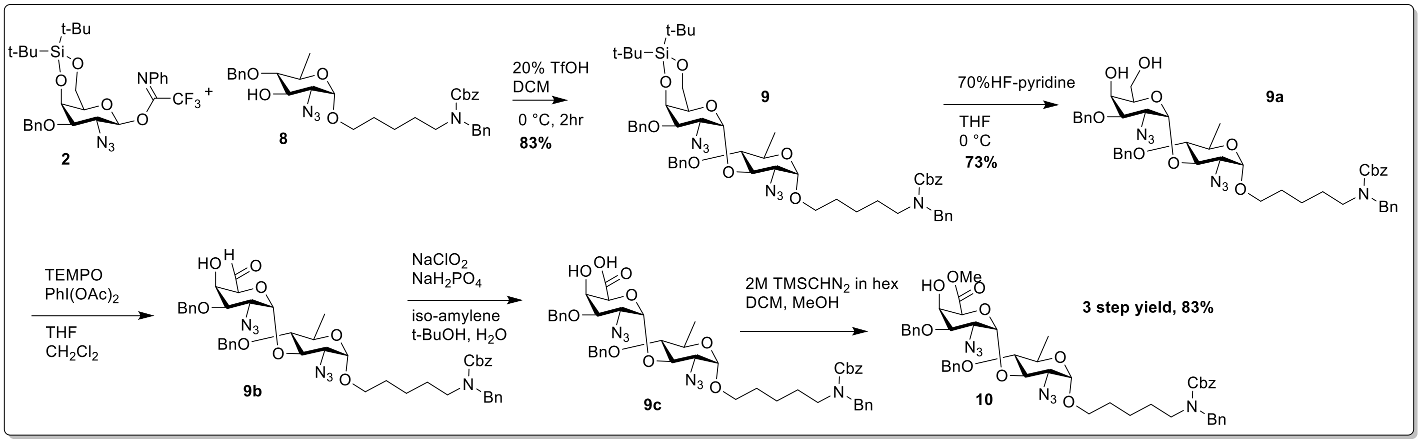

Synthesis of **9**

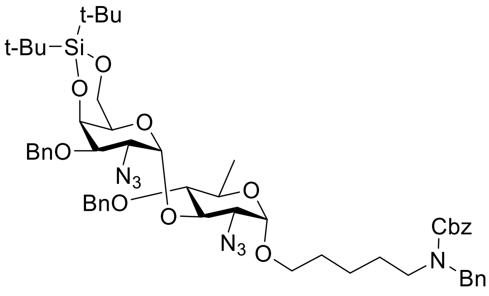

A mixture of donor **2** (1.1556g, 1.9047 mmol) and glycosyl acceptor **8** (0.9230g, 1.5678 mmol) in an oven-dried 20ml scintillation vial was co-evaporated with 2.0 ml toluene twice, then briefly placed under high vacuum. The vial was charged with an oven-dried stir bar then capped with a 14/20 rubber septum. A nitrogen line was then connected to the vial before addition of dry DCM (11.0 ml) (taken directly from a solvent purification system). The setup was flushed with nitrogen gas for approx. 10 sec then stirred at 0°C. TfOH (12.5 µL, 0.142 mmol) was then added dropwise and the reaction allowed to continue stirring at 0°C. After 4 hours, TLC indicated complete consumption of acceptor **8**. The reaction mixture was quenched by dropwise addition Et_3N (10 drops), then washed vigorously with 25 ml 1M NaOH (aq) (to get rid of *N*-phenyltrifluoroacetimide). The organic phase was dried using Na_2SO_4 then concentrated to give a crude material that was purified by flash chromatography (24-36% DE/hexanes (DE= 3/1 DCM/EtOAc)) to give 1.3156g of **9** (white foam, 83% yield).

^{1}H NMR (500 MHz, $CDCl_3$) δ 7.59 – 7.09 (m, 20H), 5.59 (d, *J* = 3.9 Hz, 1H), 5.17 (m 2H), 5.02 (d, *J* = 10.6 Hz, 1H), 4.81 (m, 1H), 4.76 (d, *J* = 11.4 Hz, 1H), 4.73 -4.64 (m, 2H), 4.62 (d, *J* = 2.9 Hz, 1H), 4.49 (m, 2H), 4.31 – 4.22 (m, 2H), 4.19 (t, *J* = 9.5 Hz, 1H), 3.96 (s, 1H), 3.92 (dd, *J* = 10.6, 2.8 Hz, 1H), 3.80 (m, 2H), 3.69 – 3.55 (m, 1H), 3.37 (m, 1H), 3.26 (t, *J* = 9.1 Hz, 2H), 3.19 (m,

1H), 3.01 (dd, J = 10.5, 3.7 Hz, 1H), 1.64 – 1.45 (m, 4H), 1.37-1.25 (m, 2H), 1.32 (d, J = 6.4 Hz, 3H), 1.05 (s, 9H), 1.00 (s, 9H).

^{13}C NMR (126 MHz, CDCl$_3$) δ 156.4, 138.0, 137.9, 137.8, 136.9, 128.6, 128.5, 128.0, 127.9, 127.9, 127.5, 127.3, 98.5, 97.9, 85.9, 75.6, 74.6, 74.2, 70.5, 69.7, 68.1, 68.0, 67.5, 67.2, 67.0, 62.1, 58.1, 50.6, 50.3, 47.1, 46.2, 29.1, 27.9, 27.7, 27.5, 27.3, 23.4, 20.8, 18.0. **HRMS (ESI) m/z: [M + Na]+** Calcd for $C_{54}H_{71}N_7O_{10}SiNa$ 1028.4924; found 1028.4926; **$[\alpha]_D^{25}$ =** +82.1° (*c* 1, CH_2Cl_2). **IR (cm^{-1})** 2932, 2858, 2104, 1698, 1472, 1454, 1047, 733, 696, 443

Synthesis of **9a**

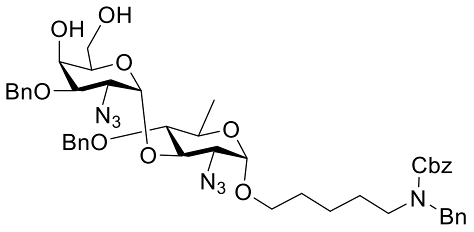

To a stirred solution of **9** (1.3036 g, 1.2955 mmol) in THF (10 ml) at 0 °C, 70% HF-pyridine (1.4 ml) was added. The reaction was allowed to stir and warm to room temperature (18 °C) under nitrogen atmosphere. 40 minutes later, TLC indicated complete consumption of starting material **9**. The reaction was quenched by dropwise addition of saturated $NaHCO_3$ (aq., 10 drops). The mixture was then diluted with EtOAc (20 ml) then washed with 10ml saturated $NaHCO_3$ (aq.) once. The organic phase was dried using Na_2SO_4, then concentrated to give a crude material that was purified by flash chromatography (40-50% EtOAc/hexanes) to give 0.8142 g of **9a** (white foam, 73% yield).

^{1}H NMR (500 MHz, CDCl$_3$) δ 7.51 – 7.09 (m, 20H), 5.58 (d, J = 3.8 Hz, 1H), 5.16 (m, 2H), 5.00 (d, J = 10.5 Hz, 1H), 4.83 (m, 1H), 4.72 (s, 2H), 4.69 (d, J = 10.6 Hz, 1H), 4.54-4.44 (m, 2H), 4.26 (m, 1H), 4.20 – 4.07 (m, 2H), 3.97 (dd, J = 10.5, 3.0 Hz, 1H), 3.91 (m, 2H), 3.83-3.72 (m, 2H), 3.69 – 3.58 (m, 1H), 3.43 – 3.32 (m, 1H), 3.30 – 3.17 (m, 3H), 3.12 (m, 1H), 2.86 (d, J = 38.4 Hz,

1H), 2.58 (s, 1H), 1.65-1.46 (m, 4H), 1.39-1.25 (m, 2H), 1.33 (d, *J* = 6.4 Hz, 3H). **^{13}C NMR (126 MHz, $CDCl_3$)** δ 156.6, 137.9, 137.7, 137.2, 128.7, 128.6, 128.5, 128.3, 128.1, 127.9, 127.9, 127.4, 127.3, 98.4, 97.6, 85.5, 76.0, 74.7, 71.8, 69.6, 68.0, 67.8, 67.2, 67.1, 63.4, 62.3, 58.8, 50.5, 50.2, 47.1, 46.1, 29.1, 27.8, 27.5, 23.4, 18.0. **HRMS (ESI) m/z: [M + Na]+** Calcd for $C_{46}H_{55}N_7O_{10}Na$ 888.3903; found 888.3902; **$[\alpha]_D^{25}$ =** +20.2° (*c* 1, CH_2Cl_2). **IR (cm^{-1})** 3444, 2926, 2105, 1695, 1454, 1423, 1252, 1226, 1132, 1049, 734, 697.

Synthesis of **10**

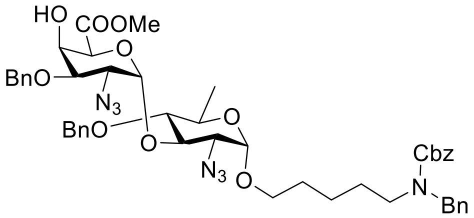

To a stirred solution of diol **9a** (0.8016 g, 0.9256 mmol) in 1/1 DCM/THF (12ml) at 0 °C, 2,2,6,6-tetramethyl-1-piperidinyloxy (TEMPO, 47.0 mg, 0.301 mmol) and (diacetoxyiodo)benzene (PIDA, 459.2 mg, 1.426 mmol) were added. The reaction was allowed to stir at 0 °C under nitrogen atmosphere for five minutes then was allowed to gradually warm to room temperature (18 °C). 1hr into the reaction, more TEMPO (12.0 mg, 0.0768 mmol) was added. More PIDA (51.1 mg) was also added (1hr after addition of more TEMPO). Three hours later (after addition of more PIDA), TLC (40% EtOAc/hexanes) indicated total consumption of **9a** and formation of intermediate aldehyde **9b** (5 hrs into the reaction).

Tert- butanol (2.5ml) and iso-amylene (0.5 ml) were then added into the reaction flask. Immediately thereafter, a solution made by dissolving $NaClO_2$ (201.6 mg, 2.229 mmol) and

NaH_2PO_4 (241.5 mg, 2.013 mmol) in water (1.1 ml) was also added to the reaction. The reaction was allowed to continue while stirring under nitrogen atmosphere. Three hours later, TLC (5/5/0.5 EtOAc/hexanes/AcOH) indicated complete consumption of the intermediate aldehyde **9b** and formation of intermediate carboxylic acid **9c**. The reaction was then quenched by dropwise addition of 1M phosphoric acid (1ml), then diluted with EtOAc (75ml). The mixture was then washed with more 1M phosphoric acid (9ml). The organic phase was separated, dried using Na_2SO_4, and then concentrated to give crude material (carboxylic acid **9c**).

The crude carboxylic acid **9c** was then dissolved in 1/1 DCM/methanol (12ml) in a pear-shaped flask with a stir bar. 4.0 ml of 2M (trimethylsilyl)diazomethane solution (8.0 mmol) in hexanes was added dropwise to the reaction under nitrogen atmosphere over a period of one minute while stirring. After one hour, TLC (5/5/0.5 EtOAc/hexanes/AcOH) indicated total consumption of carboxylic acid **9c** and formation of methyl ester **10**. The reaction was quenched by dropwise addition of AcOH (2.4 ml), then concentrated to give crude material that was purified by flash chromatography (28-40% EtOAc/hexanes) to give 684.3 mg of **10** (white foam, 83% three step yield).

^{1}H NMR (500 MHz, $CDCl_3$) δ 7.48 – 7.12 (m, 20H), 5.66 (d, *J* = 3.8 Hz, 1H), 5.17 (m, 2H), 4.99 (d, *J* = 10.6 Hz, 1H), 4.87 – 4.79 (m, 2H), 4.76 – 4.68 (m, 3H), 4.52 – 4.45 (m, 3H), 4.20 (dd, *J* = 9.6 Hz, 1H), 4.02 (dd, *J* = 10.5, 3.1 Hz, 1H), 3.83 – 3.70 (m, 2H), 3.77 (s, 3H), 3.68 – 3.54 (m, 1H), 3.44 – 3.30 (m, 1H), 3.29-3.15 (m, 3H), 3.00 (dd, *J* = 10.4, 3.7 Hz, 1H), 1.64 – 1.48 (m, 4H), 1.36 – 1.29 (m, 5H). **^{13}C NMR (126 MHz, $CDCl_3$)** δ 169.0, 156.2, 137.9, 137.7, 136.8, 128.8, 128.6, 128.5, 128.5, 128.4, 128.1, 127.9, 127.8, 127.2, 98.4, 97.8, 85.4, 75.6, 75.1, 74.6, 72.0, 70.0, 68.1, 67.2, 67.2, 67.0, 61.8, 58.4, 52.6, 50.4, 47.1, 46.2, 29.1, 27.9, 27.5, 23.4, 18.0; **HRMS (ESI) m/z: [M + Na]+** Calcd for $C_{47}H_{55}N_7O_{11}Na$ 916.3852; found 916.3871; **$[\alpha]_D^{25}$ =** +13.7° (*c* 1, CH_2Cl_2). **IR (cm^{-1})** 3454, 3031, 2924, 2104, 1769, 1734, 1697, 454, 1224, 1026, 698.

Failed [3+1] Approach

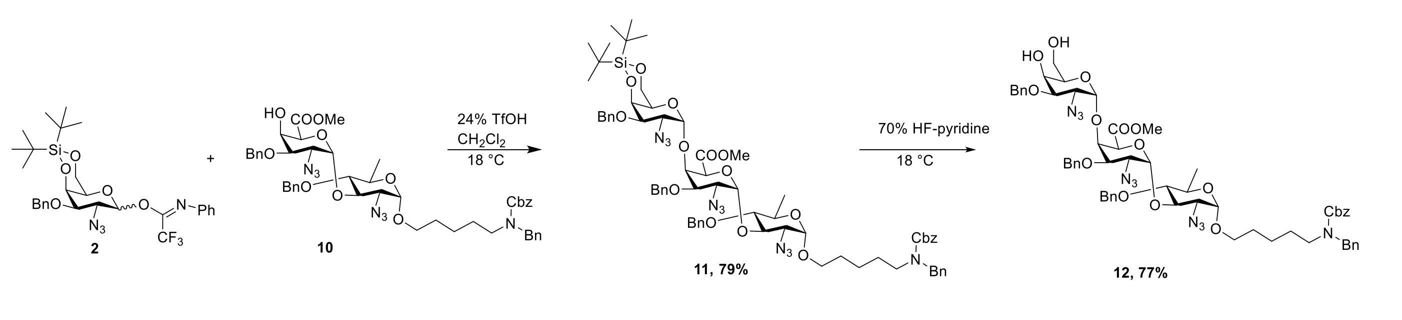

Synthesis of **11**

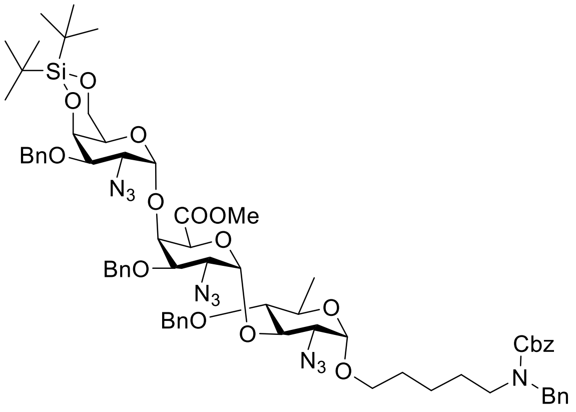

A mixture of donor **2** (42.8 mg, 0.0705 mmol) and glycosyl acceptor **10** (30.5 mg, 0.0341 mmol) in an oven-dried 5ml pear shaped flask was co-evaporated with 0.5 ml toluene twice, then placed under high vacuum. The flask was charged with a stir bar then capped with a 14/20 rubber septum. A nitrogen line was then connected to the flask before addition of dry DCM (1.0 ml taken directly from a solvent purification system). The setup was flushed with nitrogen gas for approx. 5 sec then allowed to stir at 0°C. TfOH (1.5 µL, 0.017 mmol) was then added dropwise using a microsyringe and the reaction was allowed to continue stirring at 0 °C. After 1.5 hours, TLC indicated complete consumption of acceptor **10**. The reaction mixture was quenched by dropwise addition of Et_3N (3 drops), then concentrated to give crude material that was purified by flash

chromatography (36-44% DE/hexanes (DE= 3/1 DCM/EtOAc)) to give 35.1 mg of **11** (white foam, 79% yield).

^{1}H NMR (500 MHz, $CDCl_3$) 7.50 – 7.12 (m, 25H), 5.69 (d, *J* = 3.8 Hz, 1H), 5.21-5.13 (m, 2H), 5.01 (d, *J* = 10.9 Hz, 1H), 4.97 (d, *J* = 3.5 Hz, 1H), 4.88 – 4.80 (m, 3H), 4.73 – 4.59 (m, 5H), 4.52-4.45 (m, 3H), 4.23 (t, *J* = 9.5 Hz, 1H), 3.97 (dd, *J* = 10.9, 2.8 Hz, 1H), 3.92 (s, 1H), 3.83-3.73 (m, 3H), 3.80 (s, 3H), 3.70 – 3.55 (m, 4H), 3.46 – 3.31 (m, 1H), 3.30-3.15 (m, 3H), 2.95 (dd, *J* = 10.4, 3.7 Hz, 1H), 1.67 – 1.47 (m, 4H), 1.35-1.22 (m, 2H), 1.30 (d, *J* = 6.2 Hz, 3H), 1.00 (s, 9H), 0.98 (s, 9H): **^{13}C NMR (126 MHz, $CDCl_3$) δ** ^{13}C NMR (126 MHz, $CDCl_3$) δ 169.3, 156.7, 137.9, 137.8, 137.0, 128.6, 128.5, 128.5, 128.4, 127.9, 127.9, 127.9, 127.8, 127.6, 127.2, 127.0, 127.0, 99.8, 98.5, 97.8, 85.4, 75.3, 75.3, 74.4, 71.9, 70.4, 70.3, 69.5, 68.0, 67.8, 67.2, 67.0, 66.9, 61.6, 59.1, 58.3, 52.7, 50.3, 46.9, 46.2, 28.4, 27.8, 27.6, 27.3, 23.4, 23.3, 17.9. **HRMS (ESI) m/z: [M + Na]+** Calcd for $C_{68}H_{86}N_{10}O_{15}SiNa$ 1333.5936; found 1333.5949; **$[\alpha]_D^{25}$ =** +46.5° (*c* 1, CH_2Cl_2). **IR (cm^{-1})** 2932, 2859, 2107, 1730, 1699, 1473, 1355, 1255, 1135, 1044, 826, 737, 698, 445.

Synthesis of **12**

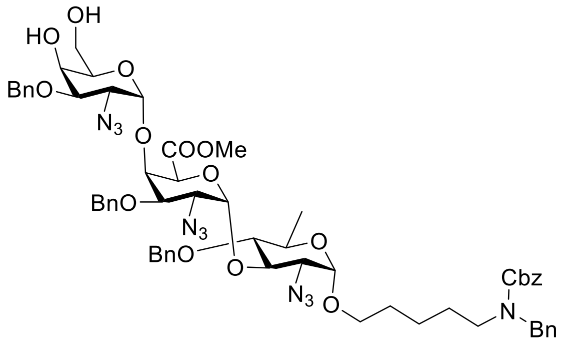

To a stirred solution of **11** (35.1 mg, 0.0268 mmol) in THF (2.0 ml) at 0 °C, 70% HF-pyridine (150 µL) was added. The reaction was then allowed to stir at room temperature under nitrogen

atmosphere. 35 minutes later, TLC indicated complete consumption of starting material **11**. The reaction was quenched by dropwise addition of saturated $NaHCO_3$ (aq., 2 mL). The mixture was then diluted with EtOAc (30 ml) then washed with 10 ml saturated $NaHCO_3$ (aq.) once. The organic phase was dried using Na_2SO_4 then concentrated to give a crude material that was purified by flash chromatography (50-60% EtOAc/hexanes) to give 24.2 mg of **12** (white foam, 77% yield).

^{1}H NMR (500 MHz, $CDCl_3$) δ 7.71 – 7.04 (m, 25H), 5.69 (d, *J* = 4.0 Hz, 1H), 5.17 (m, 2H), 5.06 – 4.95 (m, 2H), 4.92 – 4.80 (m, 3H), 4.74 – 4.60 (m, 6H), 4.54 – 4.43 (m, 2H), 4.22 (t, *J* = 9.5 Hz, 1H), 4.09 (d, *J* = 3.5 Hz, 1H), 4.04 – 3.94 (m, 2H), 3.86 (dd, *J* = 10.4, 3.2 Hz, 1H), 3.81 – 3.56 (m, 3H), 3.78 (s, 3H), 3.50 – 3.32 (m, 3H), 3.29-3.14 (m, 3H), 2.96 (dd, *J* = 10.5, 3.6 Hz, 1H), 2.68 (s, 1H), 1.99 (s, 1H), 1.64 -1.49 (m, 4H), 1.34 -1.28 (m, 2H), 1.31 (d, *J* = 6.3 Hz, 3H).**^{13}C NMR (126 MHz, $CDCl_3$) δ** 168.9, 156.7, 137.9, 137.1, 137.0, 128.7, 128.7, 128.6, 128.5, 128.5, 128.3, 128.2, 128.1, 128.1, 127.9, 127.9, 127.7, 127.4, 127.0, 127.0, 100.0, 98.5, 97.8, 85.4, 75.9, 75.5, 75.4, 75.0, 74.4, 72.2, 71.9, 70.3, 69.6, 68.0, 67.6, 67.2, 67.0, 62.7, 61.6, 60.4, 59.1, 59.1, 52.7, 50.5, 50.3, 47.1, 46.2, 29.1, 27.9, 27.5, 23.4, 18.0. **HRMS (ESI) m/z: [M + Na]+** Calcd for $C_{60}H_{70}N_{10}O_{15}Na$ 1193.4914 found; 1193.4921; **$[\alpha]_D^{25}$ =** -116.8° (*c* 1, CH_2Cl_2). **IR (cm^{-1})** 3443, 3031, 2926, 2107, 1730, 1697, 1454, 1354, 1255, 1133, 1045, 1027, 737, 698.

Synthesis of Disaccharide Donor 16

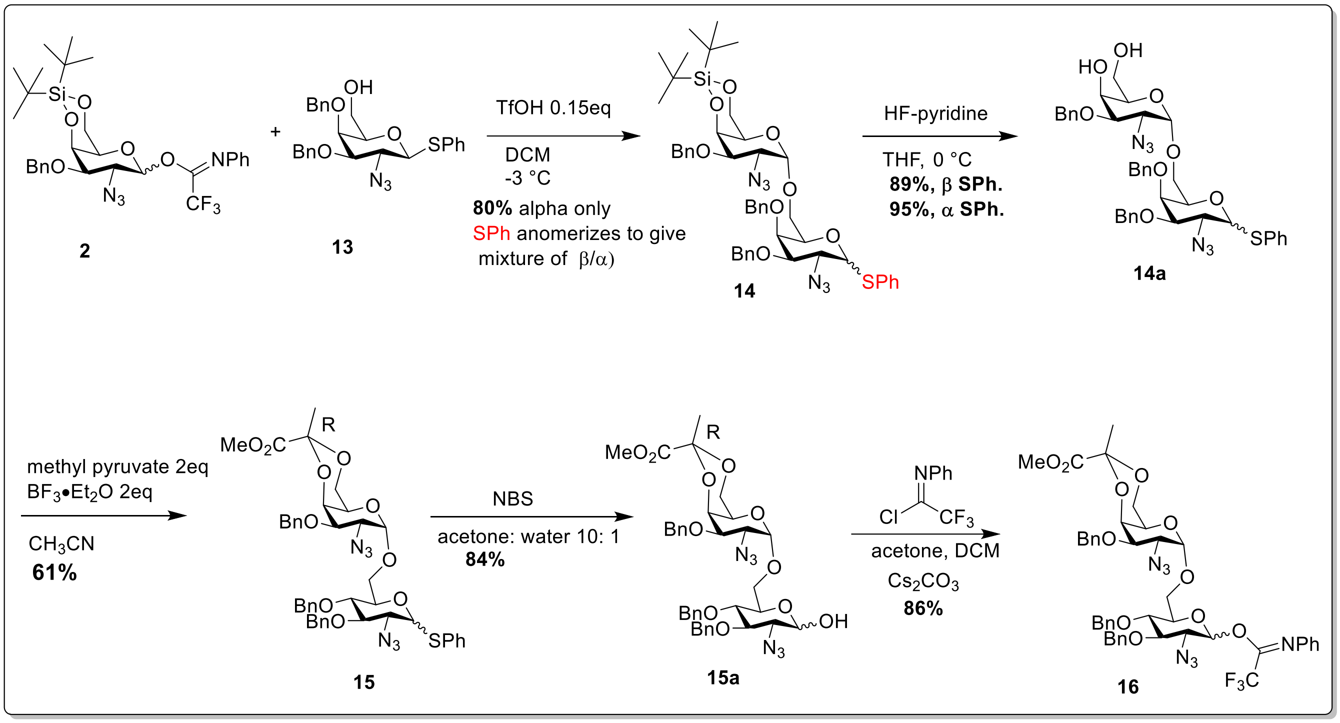

Synthesis of **14**

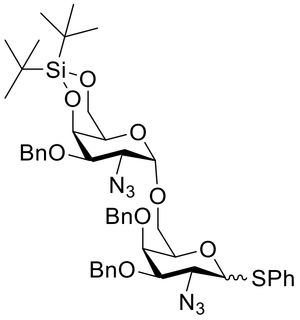

A mixture of donor **2** (267.5 mg, 0.4409 mmol) and acceptor **13** (190.7 mg, 0.3993 mmol) in a pear-shaped flask was co-evaporated with 1 ml toluene twice then placed under high vacuum briefly. The flask was charged with an oven-dried stir bar then was capped with a rubber septum. Dichloromethane (3.0 ml) was added under nitrogen atmosphere, then the flask was flushed with nitrogen gas for approximately 10 seconds. The mixture was allowed to stir at -3 °C before TfOH (7.0 µL, 0.08 mmol) was added dropwise. The reaction was allowed to continue stirring at -3 °C under nitrogen atmosphere. After four hours, TLC showed total consumption of both donor and

acceptor. The reaction was quenched by addition of 3 drops of Et_3N. The reaction mixture was then washed once (vigorously) with 15 ml 1M NaOH. The organic phase was concentrated then purified by flash chromatography to give 285.8 g of a white foam **14**, 80% yield. **Note**: the thiophenyl thioaglycone end (SPh) anomerized to give a mixture of *alpha* and *beta* thioglycosides.

Data for 14 with *beta* SPh; ^{1}H NMR (400 MHz, $CDCl_3$) δ 7.53 – 7.17 (m, 20H), 4.91 (d, *J* = 11.5 Hz, 1H), 4.76 – 4.68 (m, 4H), 4.62 (d, *J* = 11.5 Hz, 1H), 4.56 (d, *J* = 11.6 Hz, 1H), 4.49 (s, 1H), 4.42 (d, *J* = 10.1 Hz, 1H), 4.10 (d, *J* = 1.9 Hz, 2H), 3.90 – 3.80 (m, 3H), 3.76 (m, 2H), 3.63 (m, 1H), 3.58 – 3.52 (m, 1H), 3.47-3.40 (m, 2H), 1.04 (s, 9H), 1.03 (s, 9H). **^{13}C NMR (101 MHz, $CDCl_3$)** δ 138.1, 137.8, 137.3, 132.1, 132.0, 128.9, 128.7, 128.6, 128.4, 128.2, 128.0, 127.9, 127.9, 127.8, 127.8, 127.7, 98.2, 85.7, 82.4, 77.4, 75.5, 74.3, 72.6, 72.2, 70.3, 69.5, 67.4, 67.1, 61.3, 58.3, 27.7, 27.4, 23.4, 20.7; **HRMS (ESI) m/z: [M + Na]+** Calcd for $C_{47}H_{58}N_6O_8SSiNa$ 917.3698; found 917.3704; **$[\alpha]_D^{25}$ =** +75.5° (*c* 1, CH_2Cl_2). **IR (cm^{-1})** 2858, 2109, 1473, 1361, 1260, 1099, 1045, 797, 696, 444.

Data for 14 with *alpha* SPh ^{1}H NMR (500 MHz, $CDCl_3$) δ 7.50 – 7.07 (m, 20H), 5.66 (d, *J* = 5.4 Hz, 1H), 4.96 (d, *J* = 11.3 Hz, 1H), 4.79 (m, 2H), 4.72 (d, *J* = 11.5 Hz, 1H), 4.68 (d, *J* = 3.4 Hz, 1H), 4.61 (d, *J* = 3.2 Hz, 1H), 4.58 (d, *J* = 3.1 Hz, 1H), 4.49 – 4.46 (m, 1H), 4.44 (dd, *J* = 10.6, 5.5 Hz, 1H), 4.36 (td, *J* = 6.0, 1.2 Hz, 1H), 4.08 – 4.01 (m, 3H), 3.80 – 3.72 (m, 2H), 3.71 – 3.66 (m, 2H), 3.48 (dd, *J* = 10.0, 5.8 Hz, 1H), 3.41 (s, 1H), 1.06 (s, 9H), 1.02 (s, 9H); **^{13}C NMR (126 MHz, $CDCl_3$)** δ 138.0, 137.9, 137.3, 133.9, 132.9, 131.6, 129.0, 128.9, 128.7, 128.7, 128.5, 128.2, 128.2, 128.0, 127.9, 127.9, 127.8, 127.4, 98.3, 87.5, 79.1, 75.5, 74.8, 73.4, 72.6, 70.7, 70.3, 69.6, 67.4, 67.2, 67.0, 60.3, 58.4, 27.7, 27.3, 23.4, 20.7; **HRMS (ESI) m/z: [M + Na]+** Calcd for $C_{47}H_{58}N_6O_8SSiNa$ 917.3698; found 917.3710; **$[\alpha]_D^{25}$ =** +131.9° (*c* 1, CH_2Cl_2). **IR (cm^{-1})** 2932, 2858, 2110,1473, 1102, 1067, 740, 697, 651.

Synthesis of **14a *beta* SPh**

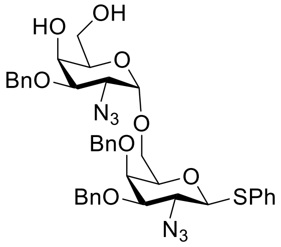

To a stirred solution of **14 beta SPh** (161.8 mg, 0.1807 mmol) in THF (5 ml) at 0 °C in a scintillation vial, 70% HF-pyridine (100 µL) was added dropwise. After 5 minutes, the reaction was allowed to warm to room temperature (18 °C). TLC showed total consumption of **14 beta SPh** after 20 minutes. The reaction was quenched by dropwise addition of saturated aqueous $NaHCO_3$ until gas evolution ceased. The mixture was diluted with 40 mL EtOAc then washed with 5 ml saturated aqueous $NaHCO_3$. The organic phase was dried using Na_2SO_4 and then concentrated to give a crude material that was purified by flash chromatography (50-70% EtOAc/hexanes) to give 121.7 mg of **14a beta SPh** (white foam, 89% yield).

Data for 14a with *beta* SPh; ^{1}H NMR (500 MHz, $CDCl_3$) δ 7.69 – 7.04 (m, 20H), 4.92 (d, *J* = 11.5 Hz, 1H), 4.73 (m, 3H), 4.67 (s, 2H), 4.55 (d, *J* = 11.5 Hz, 1H), 4.42 (d, *J* = 10.1 Hz, 1H), 4.06 (d, *J* = 3.2 Hz, 1H), 3.91 – 3.71 (m, 7H), 3.66 (dd, *J* = 10.4, 3.6 Hz, 1H), 3.56 (t, *J* = 6.1 Hz, 1H), 3.45 (m, 2H), 2.90 (br s, 1H), 2.55 (br s, 1H); **^{13}C NMR (126 MHz, $CDCl_3$)** δ 138.2, 137.4, 137.2, 132.4, 131.8, 129.0, 128.8, 128.7, 128.7, 128.4, 128.4, 128.2, 128.0, 128.0, 127.9, 127.9, 127.8, 127.8, 98.2, 86.0, 82.4, 77.2, 76.0, 74.4, 72.6, 72.3, 71.8, 69.5, 67.3, 67.1, 62.8, 61.4, 59.0. **HRMS (ESI) m/z: [M + Na]+** Calcd for $C_{39}H_{42}N_6O_8SNa$ 777.2677; found 777.2681; **$[\alpha]_D^{23}$ =** -75.2° (*c* 1, CH_2Cl_2). **IR (cm^{-1})** 3447, 3031, 2924, 2108, 1355, 1264, 1046, 1027, 738, 697.

Synthesis of **14a *alpha* SPh**

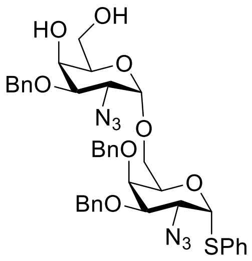

To a stirred solution of **14** alpha SPh (119.4 mg, 0.1334 mmol) in THF (3 ml) at 0 °C in a scintillation vial, 70% HF-pyridine (100 µL) was added dropwise. After 5 minutes, the reaction was allowed to warm to room temperature. TLC showed total consumption of **14 alpha SPh** after 25 minutes. The reaction was quenched by dropwise addition of saturated aqueous $NaHCO_3$ until gas evolution ceased. The mixture was diluted with 40 mL EtOAc then washed with 5 ml saturated aqueous $NaHCO_3$. The organic phase was dried using Na_2SO_4 and then concentrated to give a crude material that was purified by flash chromatography (50-60% EtOAc/hexanes) to give 95.6 mg of **14a alpha SPh** (white foam) 95% yield.

Data for 14a with *alpha* SPh; ^{1}H NMR (500 MHz, $CDCl_3$) δ 7.60-7.15 (m, 20H), 5.67 (d, *J* = 5.4 Hz, 1H), 4.95 (d, *J* = 11.3 Hz, 1H), 4.78 (d, *J* = 2.2 Hz, 2H), 4.70 (d, *J* = 3.5 Hz, 1H), 4.63 (s, 2H), 4.57 (d, *J* = 11.5 Hz, 1H), 4.47-4.36 (m, 2H), 4.04-3.96 (m, 2H), 3.82 – 3.75 (m, 2H), 3.75 – 3.66 (m, 3H), 3.62 (dd, *J* = 10.4, 3.6 Hz, 1H), 3.57 (t, *J* = 5.3 Hz, 1H), 3.47 (dd, *J* = 9.9, 5.9 Hz, 1H), 2.78 (br s, 1H), 2.40 (br s, 1H). **^{13}C NMR (126 MHz, $CDCl_3$)** δ 138.0, 137.3, 137.1, 133.8, 131.6, 129.1, 128.8, 128.7, 128.7, 128.6, 128.5, 128.4, 128.2, 128.0, 128.0, 128.0, 127.9, 127.8, 127.4, 98.1, 87.4, 79.2, 75.9, 74.8, 73.5, 72.7, 71.9, 70.5, 69.4, 67.4, 67.3, 62.9, 60.3, 59.0. **HRMS (ESI) m/z: [M + Na]+** Calcd for $C_{39}H_{42}N_6O_8SNa$ 777.2677; found 777.2675; **$[\alpha]_D^{23}$ =** +6.2° (*c* 1, CH_2Cl_2). **IR (cm^{-1})** 3473, 2917, 2109, 1454, 1262, 1027, 739, 697.

Synthesis of **15**

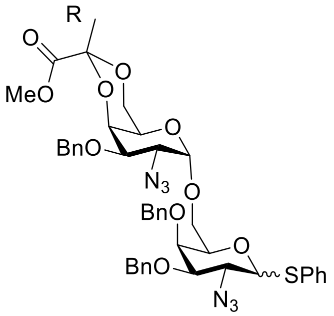

To a stirred solution of **14a** (**mix of alpha and beta SPh**) (219.4 mg, 0.2907 mmol) in CH_3CN (0.5 ml) in a pear shaped flask under nitrogen atmosphere, methyl pyruvate (53 µL, 0.58 mmol) was added followed by $BF_3 \cdot Et_2O$ (54 µL, 0.44 mmol). After 1.5 hrs, TLC showed formation of product but presence of unreacted **14a**. More methyl pyruvate (20 µL, 0.2 mmol) and $BF_3 \cdot Et_2O$ (20 µL, 0.16 mmol) were added and the reaction allowed to stir for an additional 40 minutes. Even though TLC showed some starting material still present, the reaction was quenched by dropwise addition of 0.5 ml saturated aqueous $NaHCO_3$. The mixture was then diluted with DCM (20 ml). The organic phase was separated and dried with Na_2SO_4 then concentrated to give a crude material that was purified by flash chromatography (40-42% DE/hexanes (DE= 3/1 DCM/EtOAc)) to give 147.8 mg of **15** (**mix of alpha and beta SPh**) (white foam, 61% yield).

47.2 mg of **14a** (**mix of alpha and beta SPh**) was recovered. **Note**: The reaction was quenched before all the starting **14a** (**mix of alpha and beta SPh**) was consumed as it was observed in previous runs that allowing the reaction to continue for extended times (>12hrs) or addition of excess amounts of methyl pyruvate and/or $BF_3 \cdot Et_2O$ resulted in substantial decomposition of already-formed product. Spectra for pure **15 beta SPh** and **15 alpha SPh** were obtained from previous reactions using pure **14a beta SPh** and **14a alpha SPh,** respectively, while screening for optimal reaction conditions. Slight anomerization was once again observed while using either pure **14a beta SPh** or **14a alpha SPh** under these conditions.

15 with beta SPh ^{1}H NMR (500 MHz, $CDCl_3$) δ 7.54 – 7.07 (m, 20H), 4.90 (d, *J* = 11.5 Hz, 1H), 4.85 (d, *J* = 11.3 Hz, 1H), 4.79 (d, *J* = 3.2 Hz, 1H), 4.73 (s, 2H), 4.59 (d, *J* = 11.4 Hz, 1H), 4.52 (d, *J* = 11.7 Hz, 1H), 4.45 (d, *J* = 10.1 Hz, 1H), 4.22 (s, 1H), 3.95 (d, *J* = 12.7 Hz, 1H), 3.90 – 3.76 (m, 8H), 3.71 (d, *J* = 11.8 Hz, 1H), 3.56- 3.50 (m, 2H), 3.42 (dd, *J* = 9.8, 2.7 Hz, 1H), 3.35 (dd, *J* = 10.1, 4.2 Hz, 1H), 1.59 (s, 3H). **^{13}C NMR (126 MHz, $CDCl_3$)** δ 170.4, 137.9, 137.5, 137.2, 132.3, 131.4, 129.0, 128.9, 128.7, 128.7, 128.6, 128.6, 128.5, 128.4, 128.3, 128.2, 128.2, 128.1, 128.0, 128.0, 128.0, 128.0, 127.9, 127.5, 98.8, 98.1, 85.5, 82.4, 77.4, 74.3, 73.4, 72.8, 72.3, 69.9, 67.9, 67.5, 65.7, 61.7, 61.4, 58.5, 52.7, 26.0. **HRMS (ESI) m/z: [M + Na]+** Calcd for $C_{43}H_{46}N_6O_{10}SNa$ 861.2888; found 861.2886; **$[\alpha]_D^{25}$ =** -77.6° (*c* 1, CH_2Cl_2). **IR (cm^{-1})** 2924, 2111, 1744, 1584, 1455, 1362, 1274, 1124, 741, 698.

15 with alpha SPh; ^{1}H NMR (500 MHz, $CDCl_3$) δ 7.55 – 7.07 (m, 20H), 5.66 (d, *J* = 5.3 Hz, 1H), 4.95 (d, *J* = 11.4 Hz, 1H), 4.85 – 4.72 (m, 4H), 4.55 (d, *J* = 11.5 Hz, 2H), 4.42 (dd, *J* = 10.6, 5.4 Hz, 1H), 4.36 (t, *J* = 5.9 Hz, 1H), 4.23 (d, *J* = 3.6 Hz, 1H), 3.97 – 3.68 (m, 10H), 3.40 (dd, *J* = 10.3, 5.0 Hz, 1H), 3.27 (s, 1H), 1.60 (s, 3H); **^{13}C NMR (126 MHz, $CDCl_3$)** δ 170.4, 137.9, 137.5, 137.2, 134.5, 133.9, 132.5, 131.5, 129.1, 129.0, 128.7, 128.6, 128.6, 128.5, 128.4, 128.3, 128.2, 128.2, 128.1, 128.1, 128.0, 128.0, 128.0, 128.0, 127.3, 98.8, 98.2, 87.3, 79.1, 74.9, 73.6, 73.4, 72.8, 70.8, 69.9, 67.8, 67.5, 65.6, 61.6, 60.2, 58.4, 52.7, 25.9. **HRMS (ESI) m/z: [M + Na]+** Calcd for $C_{43}H_{46}N_6O_{10}SNa$ 861.2888; found 861.2886; **$[\alpha]_D^{25}$ =** -24.2° (*c* 1, CH_2Cl_2). **IR (cm^{-1})** 2921, 2108, 1743, 1454, 1261, 1122, 1069, 801, 739, 697.

Synthesis of **15a**

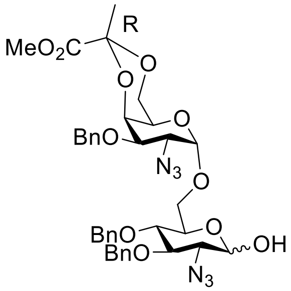

To a stirred solution of **15** (**mix of alpha and beta SPh**, 263.1 mg, 0.3136 mmol) in 11/1 acetone/water (3.2ml) in an R.B.F, NBS (227.6 mg, 1.279 mmol) was added. The reaction mixture was stirred at room temperature under nitrogen atmosphere. After 1 hour, TLC showed total consumption of starting material **15.** The reaction mixture was concentrated then diluted using DCM (30ml). The resultant organic solution was washed once using 20 ml water. The organic phase was dried using Na_2SO_4, then concentrated to give a crude material that was purified by flash chromatography (44-54% EtOAc/ hexanes) to give 196.3 mg of lactol **15a** (white foam, 84% yield).

^{1}H NMR (500 MHz, $CDCl_3$) δ 7.55 – 7.11 (m, 25H), 5.31 – 5.27 (m, 1H), 4.93 (d, *J* = 11.4 Hz, 1H), 4.87 (dd, *J* = 11.5, 3.2 Hz, 1H), 4.81 (d, *J* = 3.5 Hz, 1H), 4.78 (d, *J* = 3.0 Hz, 1H), 4.77 – 4.71 (m, 3H), 4.66 – 4.59 (m, 2H), 4.56 (d, *J* = 11.6 Hz, 1H), 4.45 – 4.37 (m, 2H), 4.09 (t, *J* = 6.1 Hz, 1H), 4.01 – 3.70 (m, 21H), 3.56 – 3.45 (m, 3H), 3.41 – 3.33 (m, 2H), 1.62 (m, 6H); **^{13}C NMR (126 MHz, $CDCl_3$)** δ170.5, 138.0, 137.7, 137.5, 137.4, 137.3, 128.7, 128.6, 128.5, 128.5, 128.3, 128.2, 128.2, 128.1, 128.0, 128.0, 98.8, 98.6, 96.6, 92.5, 92.3, 80.7, 77.3, 74.7, 74.6, 73.8, 73.4, 73.0, 73.0, 72.8, 72.4, 72.1, 69.8, 69.6, 67.6, 65.6, 64.8, 61.9, 61.7, 60.3, 59.0, 58.4, 58.4, 53.5, 52.7 25.9. **HRMS (ESI) m/z: [M + Na]+** Calcd for $C_{37}H_{42}N_6O_{11}Na$ 769.2804; found 769.2800; **$[\alpha]_D^{25}$ =** +24.1° (*c* 1, CH_2Cl_2). **IR (cm^{-1})** 3460, 2923, 2109, 1744, 1712, 1454, 1262, 1124, 1086, 802, 737, 698.

Synthesis of **16**

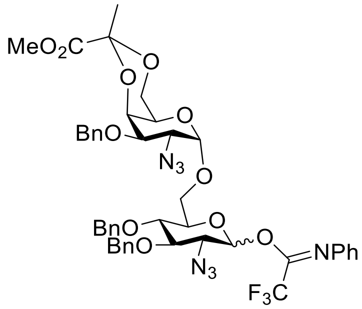

To a stirred solution of lactol **15a** (196.3 mg, 0.2629 mmol) in acetone (2.5 ml), Cs_2CO_3 (494.0 mg, 1.52 mmol) was added. 2,2,2-Trifluoro-*N*-phenylacetimidoyl chloride (108.6 mg, 0.5232 mmol) was then added dropwise. After 1 hour at room temperature (18 °C), TLC showed total consumption of lactol **15a.** The reaction was concentrated then diluted with DCM (50ml). The resultant organic solution was washed once using 10ml water. The organic phase was dried using Na_2SO_4, then concentrated to give a crude material that was purified by flash chromatography (35-42% DE/petroleum ether (DE= 3/1 DCM/EtOAc)) to give 207.7 mg of PTFAI donor **16** (yellow foam, 86% yield). **^{1}H NMR (500 MHz, $CDCl_3$) δ** 7.52 – 7.19 (m, 28H), 7.10 (m, 2H), 6.91 – 6.75 (m, 3H), 4.98-4.93 (m, 1H), 4.90 – 4.72 (m, 5H), 4.69-4.54 (m, 3H), 4.37 – 4.22 (m, 3H), 4.18 – 3.94 (m, 5H), 3.93 – 3.70 (m, 13H), 3.61-3.41 (m, 2H), 3.48-3.38 (m, 2H), 3.32 (dd, *J* = 10.0, 4.9 Hz, 1H), 1.61 (s, 3H). **^{13}C NMR (126 MHz, $CDCl_3$) δ** 173.3, 170.4, 170.3, 143.2, 137.7, 137.7, 137.4, 137.4, 137.1, 137.1, 129.2, 129.1, 129.0, 128.9, 128.7, 128.6, 128.6, 128.6, 128.5, 128.5, 128.3, 128.3, 128.2, 128.2, 128.2, 128.1, 128.1, 128.1, 128.1, 128.0, 127.9, 127.0, 124.7, 119.4, 118.4, 98.8, 98.8, 98.5, 98.1, 80.4, 74.9, 74.7, 74.6, 73.5, 73.4, 73.0, 72.7, 72.3, 71.9, 69.9, 69.8, 67.6, 67.4, 67.1, 65.6, 62.2, 61.9, 59.0, 58.4, 58.3, 52.7, 25.9, 25.9. **HRMS (ESI) m/z: [M + Na]+** Calcd for $C_{45}H_{46}F_3N_7O_{11}Na$ 940.3100; found 940.3093; **$[\alpha]_D^{25}$ =** +18.1° (*c* 1, CH_2Cl_2). **IR (cm^{-1})** 2924, 2112, 1742, 1716, 1489, 1454, 1262, 1207,1159, 1122, 1086, 802, 737, 697.

[2+2] Glycosylation, Deprotection to Target Tetrasaccharide

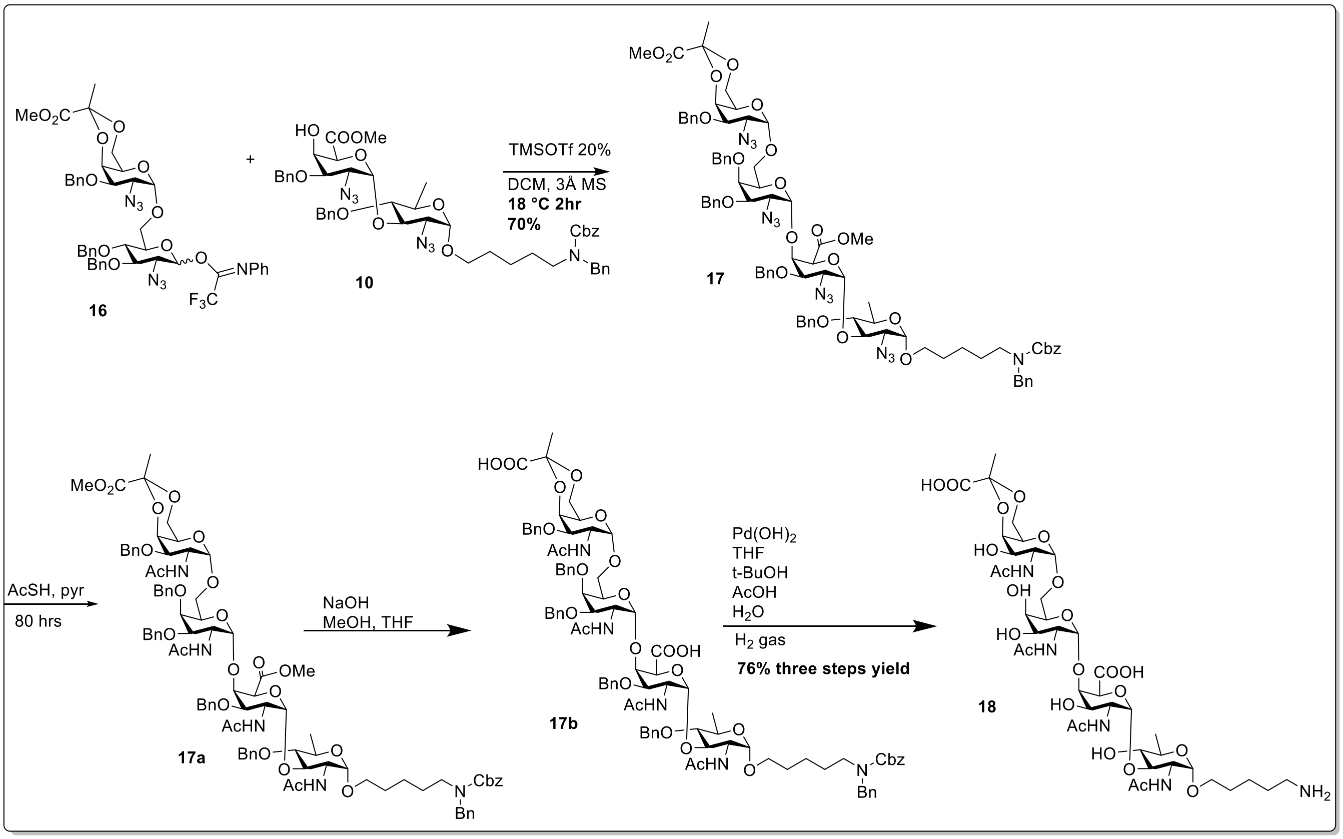

Synthesis of **17**

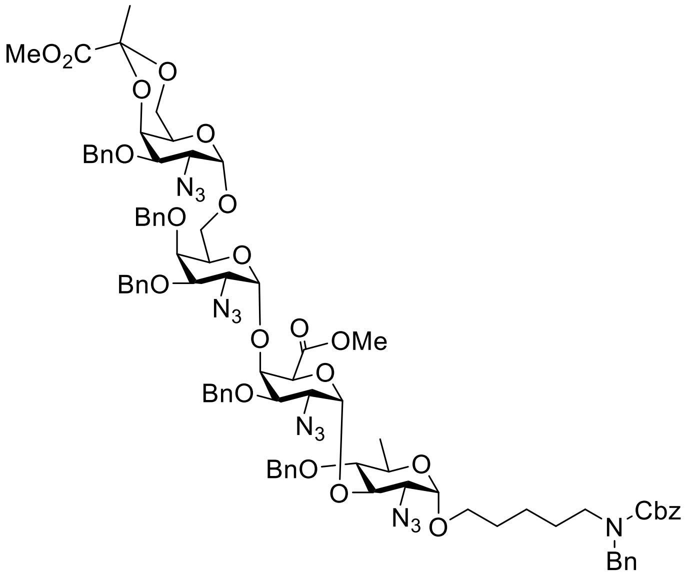

A mixture of disaccharide donor **16** (23.3 mg, 0.0254 mmol) and disaccharide acceptor **10** (19.2 mg, 0.0215 mmol) in a pear-shaped flask was co-evaporated with 0.5 ml toluene two times then

briefly placed under high vacuum. A magnetic stir bar (oven dried) and powdered 3 Å molecular sieves (219 mg, activated by flame drying under vacuum) were added into the flask. The flask was capped, then dichloromethane (0.25 ml) was added under nitrogen atmosphere. The flask was flushed with a positive pressure of nitrogen gas for approx. 5 seconds, then TMSOTf (1.5 µL, 0.0083 mmol) was added dropwise. The reaction was allowed to run at room temperature (18 °C). After 1 h and 40 min, TLC showed that all acceptor **10** was consumed. The reaction was quenched by addition of two drops of Et_3N then concentrated using a rotary evaporator to give crude material. The crude material was purified using preparative TLC (60% DEth/petroleum ether where, DEth= 5/2 DCM/Et_2O) to give 24.3 mg of tetrasaccharide **17** (thick oil, 70% yield).

^{1}H NMR (500 MHz, $CDCl_3$) δ 7.50 – 7.10 (m, 35H), 5.67 (d, *J* = 3.8 Hz, 1H), 5.17 (m, 2H), 5.06 (d, *J* = 11.0 Hz, 1H), 4.95 – 4.88 (m, 2H), 4.86 – 4.79 (m, 4H), 4.75 (d, *J* = 11.1 Hz, 1H), 4.70 (d, *J* = 3.5 Hz, 1H), 4.68 (d, *J* = 3.5 Hz, 1H), 4.65 (d, *J* = 2.7 Hz, 1H), 4.58-4.51 (m, 4H), 4.50 – 4.46 (m, 2H), 4.24 (d, *J* = 2.5 Hz, 1H), 4.20 (t, *J* = 9.5 Hz, 1H), 4.05 (dd, *J* = 8.4, 5.4 Hz, 1H), 3.99 (d, *J* = 2.5 Hz, 1H), 3.96 – 3.91 (m, 2H), 3.88 – 3.84 (m, 1H), 3.86 (s, 3H), 3.81 – 3.72 (m, 4H), 3.76 (s, 3H), 3.70 – 3.55 (m, 3H), 3.44-3.29 (m, 3H), 3.28 – 3.16 (m, 3H), 3.00 (s, 1H), 2.92 (dd, *J* = 10.5, 3.6 Hz, 1H), 1.65 – 1.45 (m, 4H), 1.58 (s, 3H), 1.36-1.24 (m, 2H), 1.29 (d, *J* = 6.4 Hz, 3H); **^{13}C NMR (126 MHz, $CDCl_3$)** δ 170.5, 169.1, 156.7, 138.1, 137.9, 137.5, 137.4, 137.4, 128.6, 128.6, 128.5, 128.5, 128.4, 128.2, 128.1, 128.0, 128.0, 127.9, 127.9, 127.8, 127.7, 127.4, 127.2, 126.9, 99.7, 98.7, 98.6, 98.5, 97.8, 85.2, 77.2, 76.0, 75.6, 74.9, 74.7, 74.4, 73.3, 72.3, 72.1, 71.7, 70.2, 69.8, 69.7, 68.0, 67.3, 67.2, 66.9, 66.0, 65.5, 61.6, 61.5, 59.9, 59.2, 58.5, 52.8, 52.7, 50.6, 50.3, 47.1, 46.2, 29.1, 27.9, 27.5, 25.9, 23.4, 17.9. **HRMS (ESI) m/z: [M + Na]+** Calcd for $C_{84}H_{95}N_{13}O_{21}Na$ 1644.6658; found 1644.6681; **$[\alpha]_D^{25}$ =** +152.8° (*c* 1, CH_2Cl_2). **IR (cm^{-1})** 2924, 2108, 1743, 1698, 1454, 1261, 1125, 1046, 914, 803, 737, 698.

Synthesis of **17a**

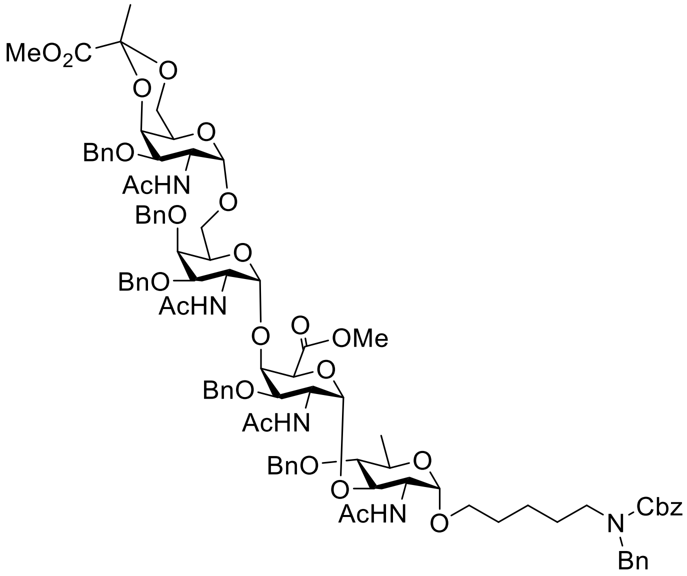

To a stirred solution of tetrasaccharide **17** (43.7 mg, 0.0269 mmol) in pyridine (0.7 ml), thioacetic acid (0.4 ml) was added. The reaction was allowed to stir at room temperature under nitrogen atmosphere. Reaction progress was monitored using FTIR by checking for disappearance of the azido signal (around 2108 cm $^{-1}$). After 32 hrs, more thioacetic acid (0.15 ml) and pyridine (0.25ml) were added. 30 hrs later, more (0.2 ml) pyridine and 0.05 ml thioacetic acid were added again (A white solid was observed in the flask. It dissolved upon addition of the extra AcSH.). After 80 hrs (from start of the reaction), stirring was concluded and the reaction mixture was concentrated then co-evaporated with toluene to give crude material (AcSH was still present due to its high boiling point). A low stream of pressurized air was carefully blown over the mixture for 1hr to get rid of most of the AcSH. The remaining AcSH was removed by loading the crude material to a silica gel column, then running 60 mL of CH_2Cl_2 through it to wash out AcSH (yellow eluent). 2-4% MeOH/DCM was then passed through the folumn to elute **17a** (white solid) which was used in the next step without further purification.

^{1}H NMR (500 MHz, MeOD) δ 7.57 – 7.06 (m, 35H), 5.54 (d, *J* = 3.7 Hz, 1H), 5.15 (m, 2H), 4.96 (d, *J* = 3.5 Hz, 1H), 4.93 (d, *J* = 6.3 Hz, 1H), 4.87 (m, 1H), 4.78 (d, *J* = 11.7 Hz, 1H), 4.75 – 4.71

(m, 2H), 4.66 (m, 2H), 4.63 (d, J = 3.4 Hz, 1H), 4.62 – 4.58 (m, 3H), 4.58 – 4.52 (m, 3H), 4.51 – 4.48 (m, 3H), 4.45 (d, J = 2.7 Hz, 1H), 4.43 – 4.39 (m, 2H), 4.39 – 4.32 (m, 2H), 4.28 (s, 1H), 4.11 (m, 1H), 4.02 (d, J = 3.5 Hz, 1H), 3.98 (dd, J = 11.2, 2.5 Hz, 1H), 3.94-3.86 (m, 1H), 3.88(s, 3H), 3.85 – 3.74 (m, 3H), 3.73 (m, 1H), 3.70(s, 3H), 3.64 – 3.49 (m, 3H), 3.61(dd, J = 11.3, 3.6 Hz, 1H), 3.46 – 3.21 (m, 6H), 2.47 (m, 1H), 1.99 (s, 3H), 1.95-1.89 (m, 6H), 1.84 (s, 3H), 1.58 – 1.46 (m, 4H), 1.48(s, 3H), 1.38-1.29 (m, 2H), 1.32(d, J = 6.4 Hz, 3H). **^{13}C NMR (126 MHz, MeOD) δ** 173.9, 173.6, 173.0, 171.9, 169.7, 158.3, 149.9, 140.4, 139.8, 139.7, 139.5, 138.8, 138.3, 137.9, 129.5, 129.4, 129.4, 129.3, 129.2, 128.8, 128.7, 128.6, 128.6, 128.5, 128.3, 128.2, 127.0, 125.5, 100.8, 100.0, 99.7, 99.3, 98.4, 86.4, 78.7, 78.0, 76.5, 76.3, 75.8, 74.9, 74.4, 73.7, 73.4, 72.8, 71.8, 70.6, 70.5, 68.9, 68.6, 68.3, 68.0, 66.4, 66.3, 62.4, 53.6, 53.0, 52.7, 51.4, 51.2, 50.7, 49.7, 29.9, 28.8, 28.5, 25.9, 24.2, 23.3, 23.1, 22.8, 22.7, 18.2. **HRMS (ESI) m/z: [M + Na]+** Calcd for $C_{92}H_{111}N_5O_{25}Na$ 1708.7460; found 1708.7454; **$[\alpha]_D^{25}$ =** +128.4° (c 1, CH_2Cl_2). **IR (cm^{-1})** 3295, 3032, 2924, 1744, 1659, 1453, 1425, 1369, 1262, 1209, 1122, 1049, 1027, 802, 734, 698.

Synthesis of **17b**

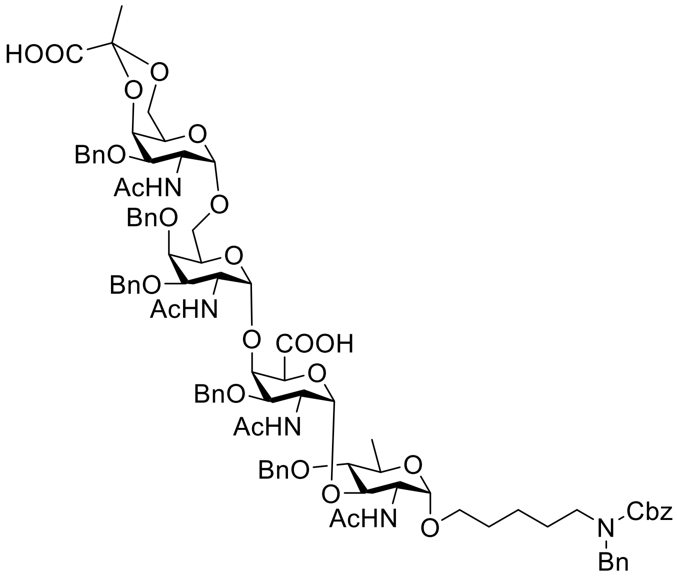

To a stirred solution of **17a** from the previous procedure in 1/1 THF/MeOH (2.0ml), 1.0 ml of 2.35 M NaOH (aq) solution was added. The reaction was allowed to continue stirring under nitrogen atmosphere at room temperature (18 °C). After 14 hrs, the reaction was quenched by dropwise addition of acetic acid (1.0 ml) then concentrated. The concentrate was diluted with EtOAc (15.0 ml). The new organic solution was washed twice using water (5.0 ml). 1ml of 1M HCl was added to the aqueous layer then washed with DCM (5.0 ml). All organic phases were combined, dried using Na_2SO_4, and then concentrated to give **17b** (fluffy white powder) which was used in the next reaction without further purification. **HRMS (ESI) m/z: [M + Na]+** Calcd for $C_{90}H_{107}N_5O_{25}Na$ 1680.7147; found 1680.7113.

Synthesis of **18**

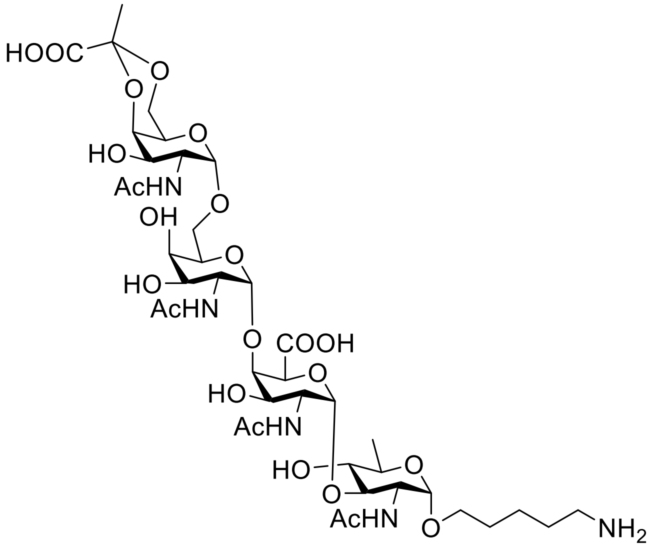

To material **17b** from the previous reaction in a vial with a stir bar, dichloromethane (0.6 ml), AcOH (0.5 ml), *t*-BuOH (0.6 ml), and H_2O (0.6 ml) were added. The mixture was stirred briefly (2 minutes), then $Pd(OH)_2$ (200.2 mg) was added in one batch. The vial was capped with a rubber septum, then hydrogen gas in a balloon was bubbled carefully through the mixture for approx. 25 seconds (this was done using two long, thin reusable syringe needles (Popper 7174 deflected

point septum penetration hypodermic needles 22 * 2’’ (50.8 MM)) where the one attached to balloon was in contact with the reaction mixture on the sharp end while the other needle was impaled through the septum and acting as a hydrogen gas outlet). The reaction was then allowed to continue stirring at room temperature under hydrogen atmosphere (in a double walled balloon (balloon inside similar size balloon)). After 68 hrs, the reaction mixture was filtered by passing through a cotton wool plug (cotton wool was pre-washed with water and methanol to prevent contamination with low molecular weight impurities in the cotton wool) and rinsed using water (10 ml) and MeOH (8.0 ml). A low stream of pressurized air was blown over the filtrate overnight to get rid of the solvents then 10.0 ml of distilled water was added and the solution was then lypholized to give 25.3 mg of crude material that was later purified by passing through sephadex G10 while using deionized water as the mobile phase to give 20.1 mg of **18** (white solid, 76% three step yield).

NMR at 318 K

^{1}H NMR (700 MHz, D_2O) δ 5.52 (d, *J* = 3.8 Hz, 1H), 5.16 (m, 2H), 4.98 (d, *J* = 3.8 Hz, 1H), 4.66 (t, *J* = 6.93 Hz, 1H), 4.58 (m, 2H), 4.51 (dd, *J* = 11.3, 3.5 Hz, 1H), 4.48 – 4.38 (m, 3H), 4.30 – 4.15 (m, 7H), 4.05 – 3.96 (m, 4H), 3.94 – 3.84 (m, 2H), 3.73-3.67 (m, 1H), 3.64 (t, *J* = 9.8 Hz, 1H), 3.24 (t, *J* = 8.3 Hz, 2H), 2.32 – 2.25 (m, 9H), 2.19 (s, 3H), 1.98 – 1.89 (m, 2H), 1.89-1.84 (m, 2H), 1.77 (s, 3H), 1.71-1.64 (m, 2H), 1.51 (d, *J* = 6.7 Hz, 3H).**^{13}C NMR (176 MHz, D_2O) δ** 174.9, 174.5, 174.3, 172.6, 99.9, 99.1, 99.0, 97.7, 97.0, 79.2, 78.2, 76.4, 71.2, 70.7, 69.4, 68.5, 68.0, 67.9, 67.5, 67.0, 66.4, 65.7, 65.5, 62.5, 52.8, 50.0, 49.9, 49.7, 39.7, 28.3, 26.7, 25.3, 22.6, 22.5(m, 1C) 22.3(m, 2C), 22.2(m, 1C), 16.7. **HRMS (ESI) m/z: [M + H]+** Calcd for $C_{40}H_{66}N_5O_{23}$ 984.4143 found 984.4137; **$[\alpha]_D^{25}$ =** +78.9° (*c* 0.8, 2/1 MeOH/H_2O). **IR (cm^{-1})** 3327, 2934, 1610, 1378, 1051, 670, 457.

References.

1) Chen, M.; Huang, C.; He, C.; Zhu, W.; Xu, Y.; Lu, Y. *Chemical Communications* **2012**, *48* (76), 9522.
2) Lourenço, E. C.; Ventura, M. R. *Carbohydrate Research* **2016**, *426*, 33.
3) Chang, C. W.; Lin, M. H.; Chan, C. K.; Su, K. Y.; Wu, C. H.; Lo, W. C.; Lam, S.; Cheng, Y. T.; Liao, P. H.; Wong, C. H.; Wang, C. C. *Angewandte Chemie International Edition* **2021**, *60* (22), 12413.
4) Chao, C.-S.; Yen, Y.-F.; Hung, W.-C.; Mong, K.-K. T. *Advanced Synthesis & Catalysis* **2011**, *353* (6), 879.
5) Ngoje, G.; Li, Z. *Organic & Biomolecular Chemistry* **2013**, *11* (11), 1879.
6) Noti, C.; de Paz, J. L.; Polito, L.; Seeberger, P. H. *Chemistry - A European Journal* **2006**, *12* (34), 8664.
7) Singh, Y. N.; Rodriguez Benavente, M. C.; Al-Huniti, M. H.; Beckwith, D.; Ayyalasomayajula, R.; Patino, E.; Miranda, W. S.; Wade, A.; Cudic, M. *The Journal of Organic Chemistry* **2019**, *85* (3), 1434.
8) Tanaka, H.; Takeuchi, R.; Jimbo, M.; Kuniya, N.; Takahashi, T. *Chemistry - A European Journal* **2013**, *19* (9), 3177.
9) Moumé-Pymbock, M.; Furukawa, T.; Mondal, S.; Crich, D. *Journal of the American Chemical Society* **2013**, *135* (38), 14249.
10) Kanemitsu, T.; Daikoku, S.; Kanie, O. *Journal of Carbohydrate Chemistry* **2006**, *25* (5), 361.
11) Hagen, B.; van Dijk, J. H.; Zhang, Q.; Overkleeft, H. S.; van der Marel, G. A.; Codée, J. D. *Organic Letters* **2017**, *19* (10), 2514.
12) Wang, L.; Zhang, Y.; Overkleeft, H. S.; van der Marel, G. A.; Codée, J. D. *The Journal of Organic Chemistry* **2020**, *85* (24), 15872.

NMR SPECTRA

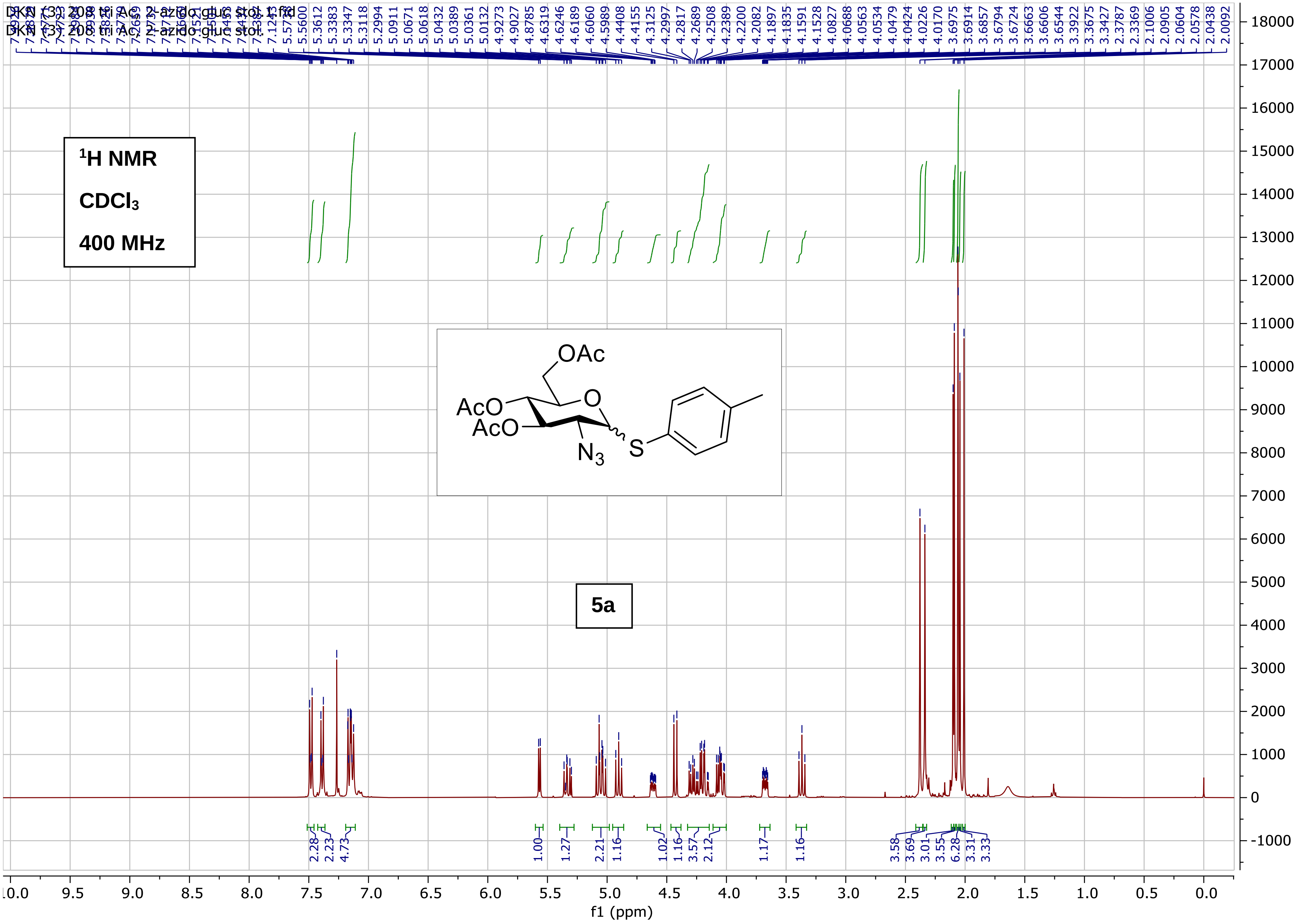

DKN (3) 208 tri Ac 2-azido gluc stol.
1H NMR
CDCl3
400 MHz
OAc
AcO
AcO
O
N3
S
5a
f1 (ppm)

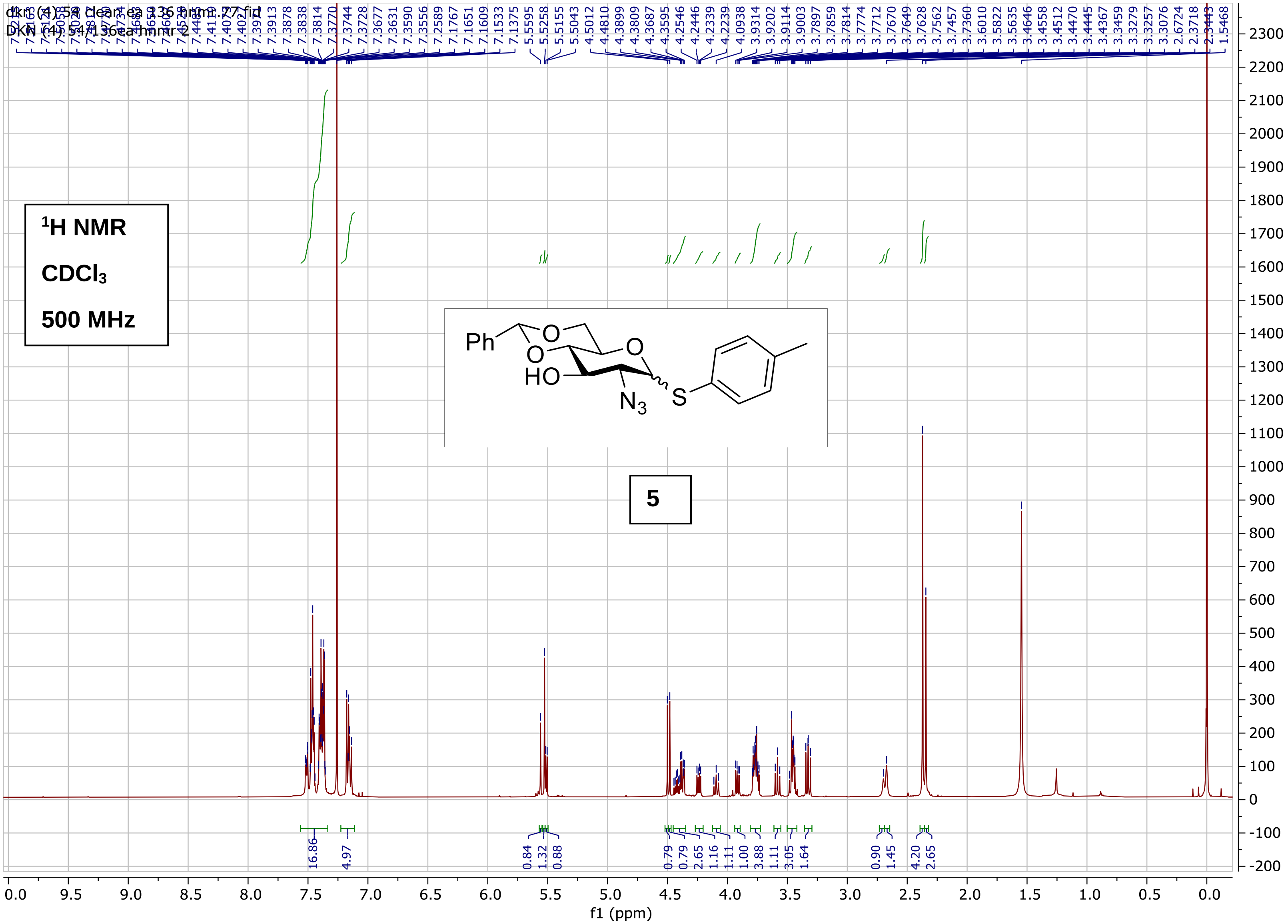

^{1}H NMR
CDCl$_3$
500 MHz
5
f1 (ppm)

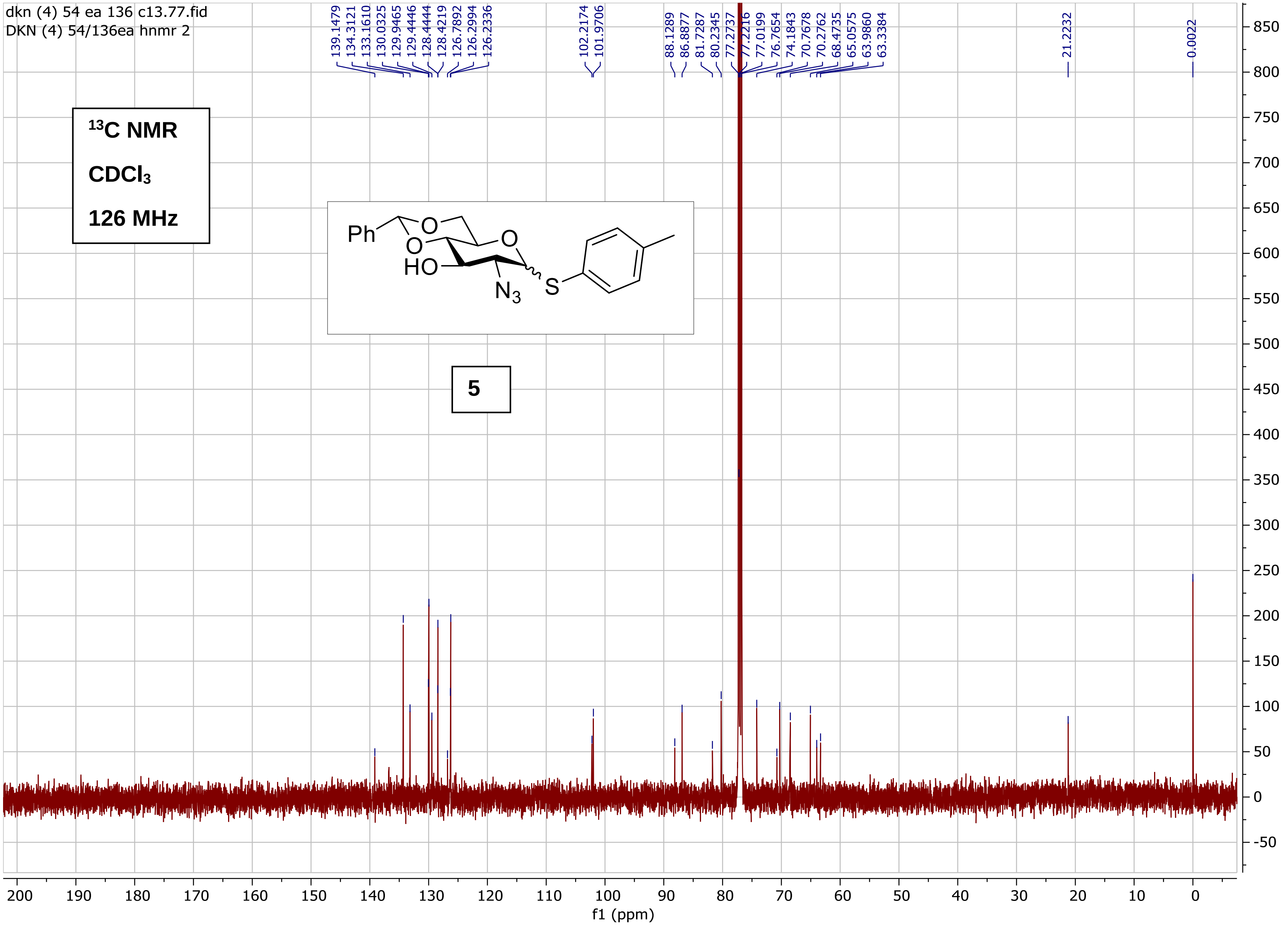
dkn (4) 54 ea 136 c13.77.fid
DKN (4) 54/136ea hnmr 2
139.1479
134.3121
133.1610
130.0325
129.9465
129.4446
128.4444
128.4219
126.7892
126.2994
126.2336
102.2174
101.9706
88.1289
86.8877
81.7287
80.2345
77.2737
77.2216
77.0199
76.7654
74.1843
70.7678
70.2762
68.4735
65.0575
63.9860
63.3384
21.2232
0.0022
13C NMR
CDCl3
126 MHz
Ph
O
O
O
HO
N3
S
5
f1 (ppm)

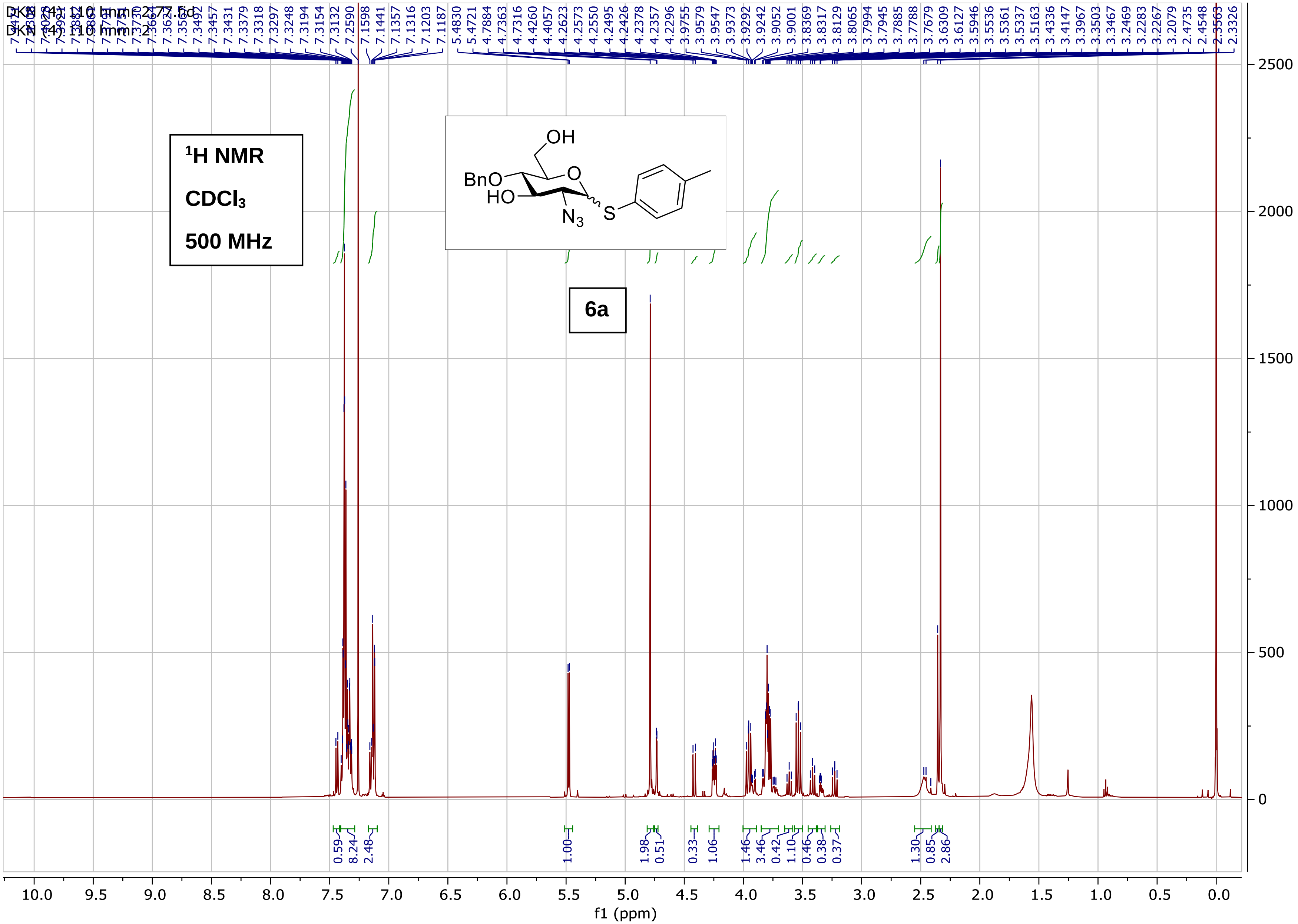
1H NMR
CDCl3
500 MHz
OH
BnO
O
HO
N3
S
6a
f1 (ppm)

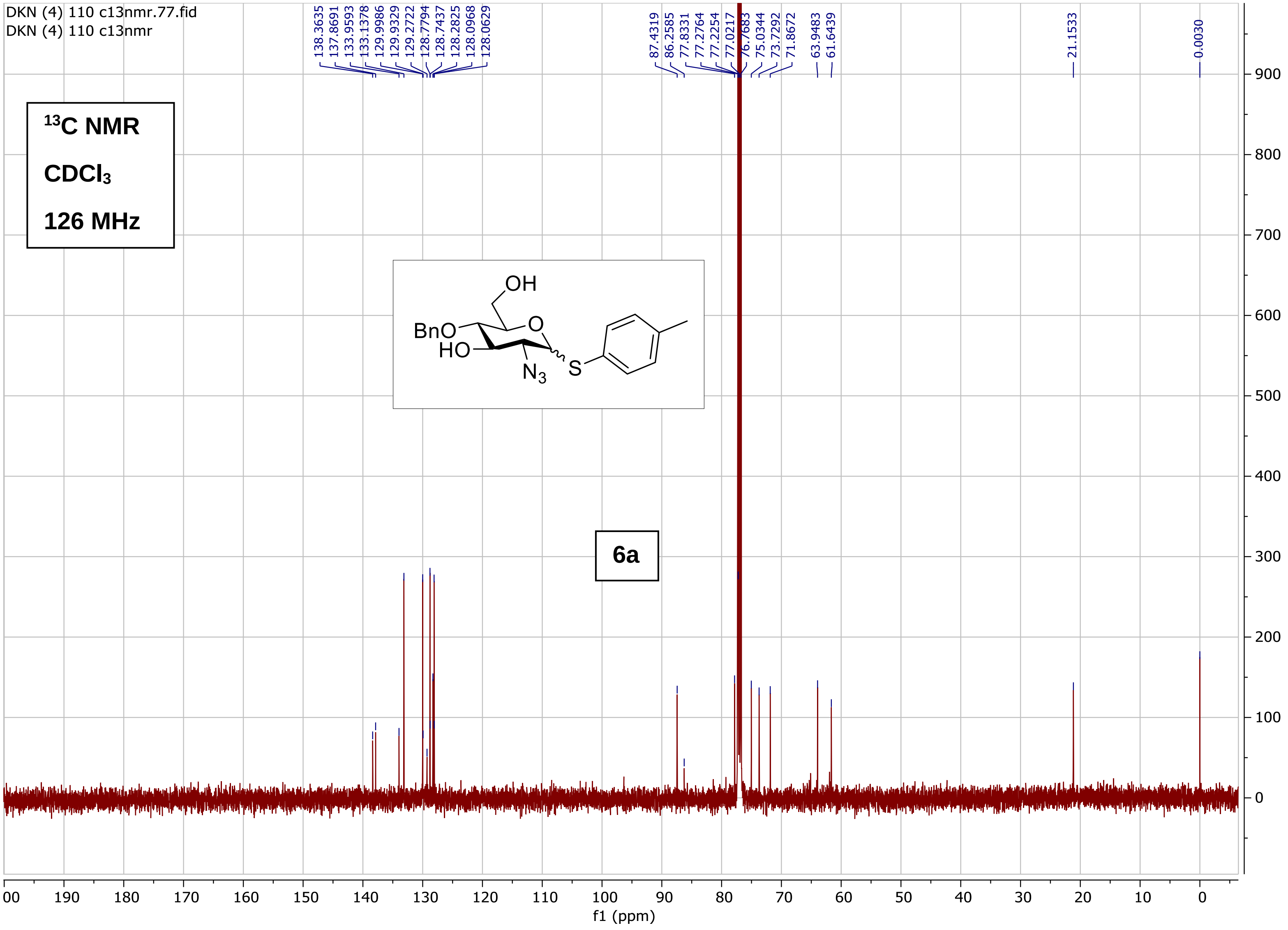
DKN (4) 110 c13nmr.77.fid
DKN (4) 110 c13nmr
138.3635
137.8691
133.9593
133.1378
129.9986
129.9329
129.2722
128.7794
128.7437
128.2825
128.0968
128.0629
87.4319
86.2585
77.8331
77.2764
77.2254
77.0217
76.7683
75.0344
73.7292
71.8672
63.9483
61.6439
21.1533
0.0030
13C NMR
CDCl3
126 MHz
OH
BnO
O
HO
N3
S
6a
f1 (ppm)

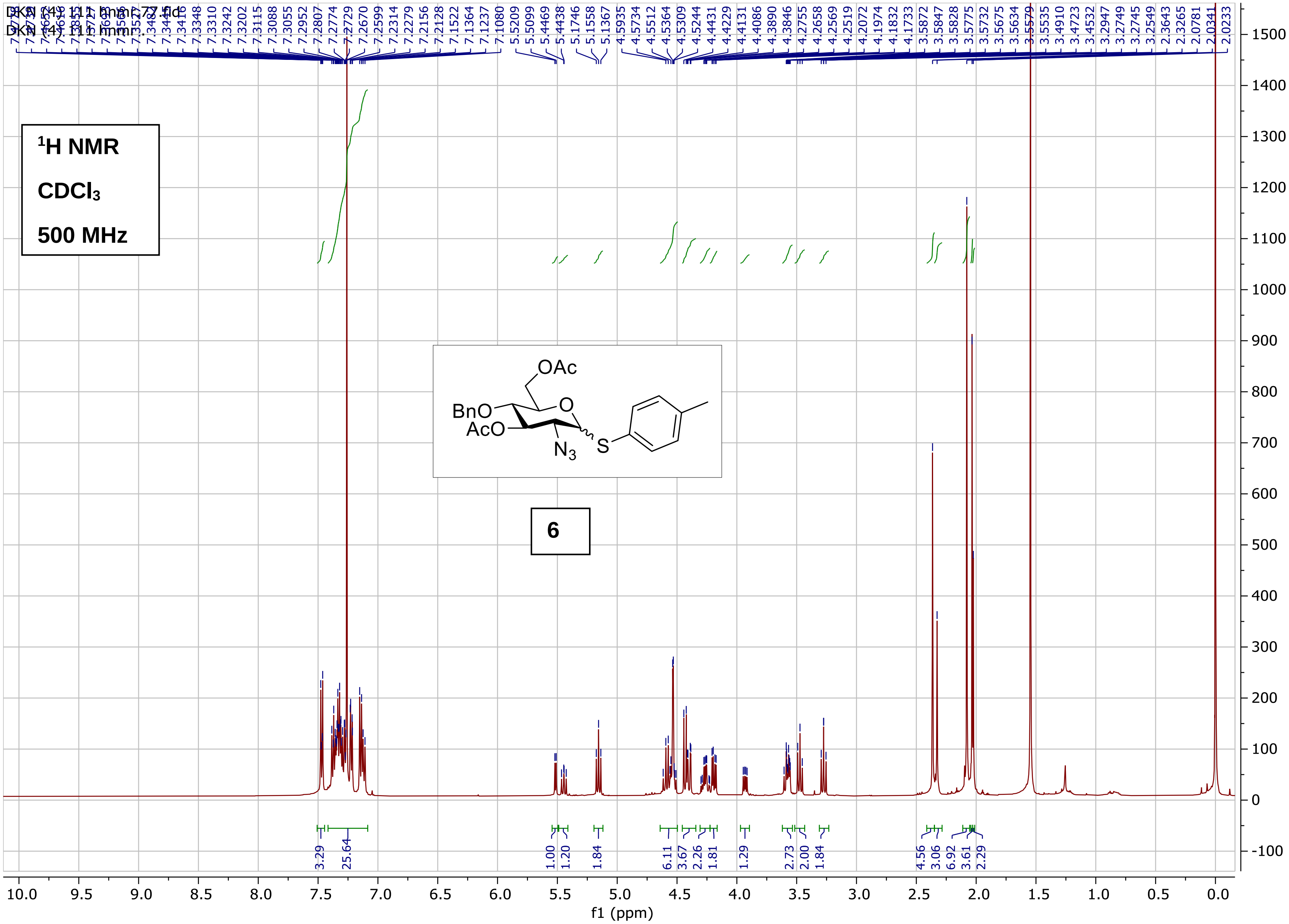

1H NMR
CDCl3
500 MHz
OAc
BnO
AcO
O
N3
S
6
7.3348
7.3310
7.3242
7.3202
7.3115
7.3088
7.3055
7.2952
7.2807
7.2774
7.2729
7.2670
7.2599
7.2314
7.2279
7.2156
7.2128
7.1522
7.1364
7.1237
7.1080
5.5209
5.5099
5.4469
5.4438
5.1746
5.1558
5.1367
4.5935
4.5734
4.5512
4.5364
4.5309
4.5244
4.4431
4.4229
4.4131
4.4086
4.3890
4.3846
4.2755
4.2658
4.2569
4.2519
4.2072
4.1974
4.1832
4.1733
3.5872
3.5847
3.5828
3.5775
3.5732
3.5675
3.5634
3.5579
3.5535
3.4910
3.4723
3.4532
3.2947
3.2749
3.2745
3.2549
2.3643
2.3265
2.0781
2.0341
2.0233
3.29
25.64
1.00
1.20
1.84
6.11
3.67
2.26
1.81
1.29
2.73
2.00
1.84
4.56
3.06
6.92
3.61
2.29
f1 (ppm)

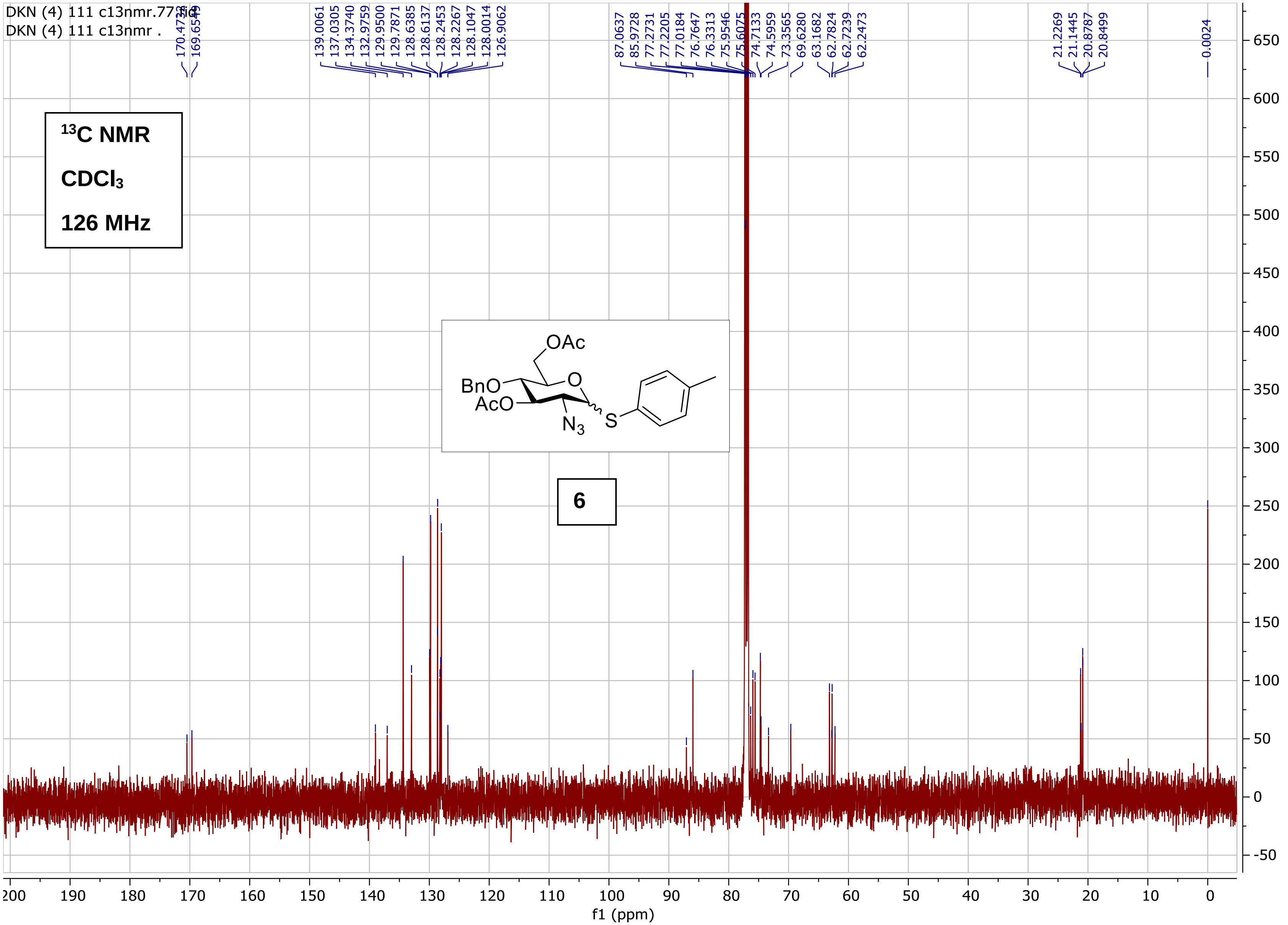

DKN (4) 111 c13nmr.77.fid
DKN (4) 111 c13nmr .
169.6549
139.0061
137.0305
134.3740
132.9759
129.9500
129.7871
128.6385
128.6137
128.2453
128.2267
128.1047
128.0014
126.9062
87.0637
85.9728
77.2731
77.2205
77.0184
76.7647
76.3313
75.9546
75.6075
74.7133
74.5959
73.3565
69.6280
63.1682
62.7824
62.7239
62.2473
21.2269
21.1445
20.8787
20.8499
0.0024
13C NMR
CDCl3
126 MHz
OAc
BnO
AcO
O
N3
S
6
f1 (ppm)

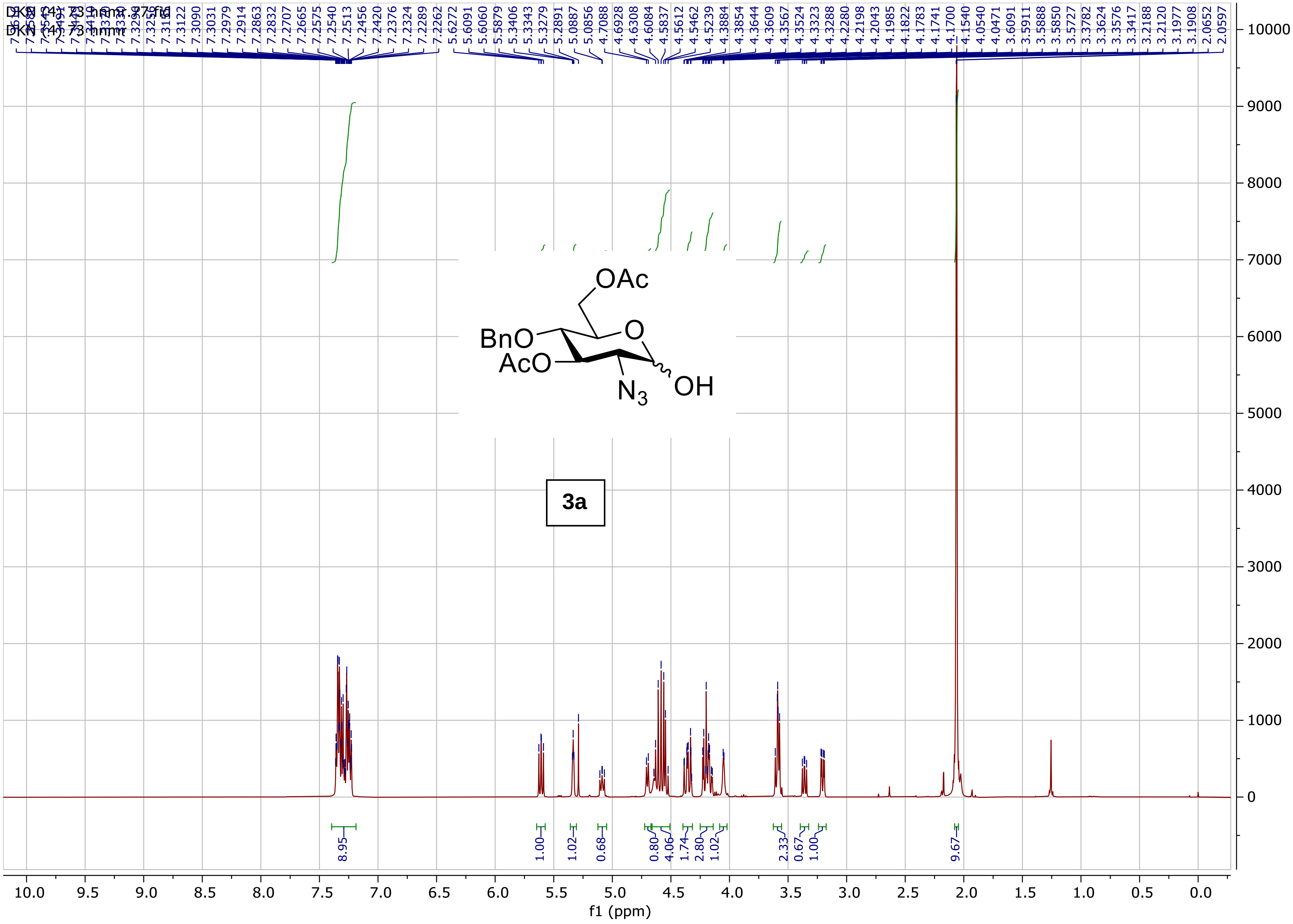
7.3122 7.3090 7.3031 7.2979 7.2914 7.2863 7.2832 7.2707 7.2665 7.2575 7.2540 7.2513 7.2456 7.2420 7.2376 7.2324 7.2289 7.2262
5.6272 5.6091 5.6060 5.5879 5.3406 5.3343 5.3279 5.2891 5.0887 5.0856 4.7088 4.6928 4.6308 4.6084 4.5837 4.5612 4.5462 4.5239 4.3884 4.3854 4.3644 4.3609 4.3567 4.3524 4.3323 4.3288 4.2280 4.2198 4.2043 4.1985 4.1822 4.1783 4.1741 4.1700 4.1540 4.0540 4.0471 3.6091 3.5911 3.5888 3.5850 3.5727 3.3782 3.3624 3.3576 3.3417 3.2188 3.2120 3.1977 3.1908 2.0652 2.0597
OAc
BnO
O
AcO
N3
OH
3a
8.95
1.00
1.02
0.68
0.80
4.06
1.74
2.80
1.02
2.33
0.67
1.00
9.67
f1 (ppm)

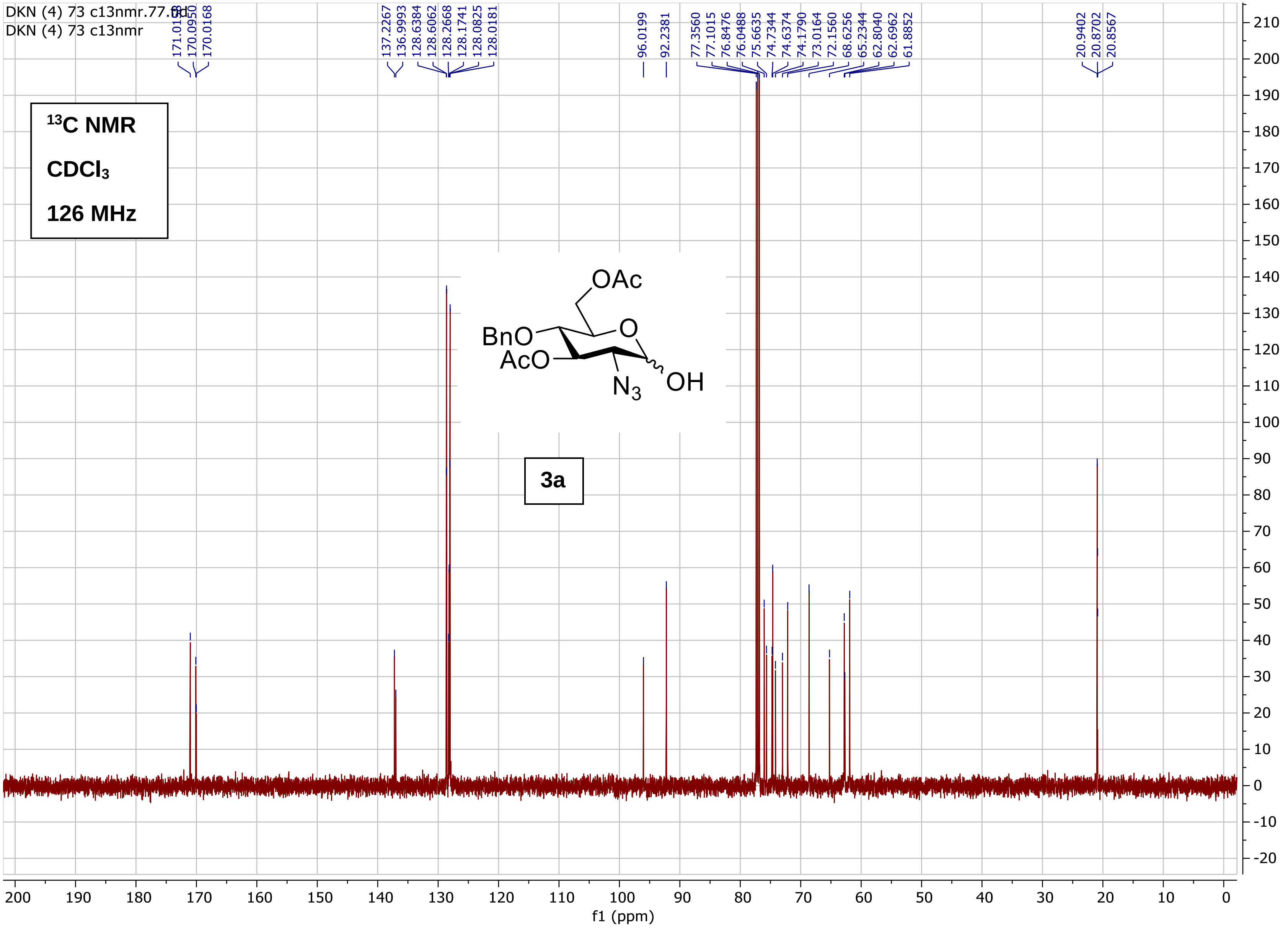

DKN (4) 73 c13nmr.77.fid
DKN (4) 73 c13nmr
171.0158
170.0950
170.0168
137.2267
136.9993
128.6384
128.6062
128.2668
128.1741
128.0825
128.0181
96.0199
92.2381
77.3560
77.1015
76.8476
76.0488
75.6635
74.7344
74.6374
74.1790
73.0164
72.1560
68.6256
65.2344
62.8040
62.6962
61.8852
20.9402
20.8702
20.8567
13C NMR
CDCl3
126 MHz
OAc
BnO
AcO
O
N3
OH
3a
f1 (ppm)

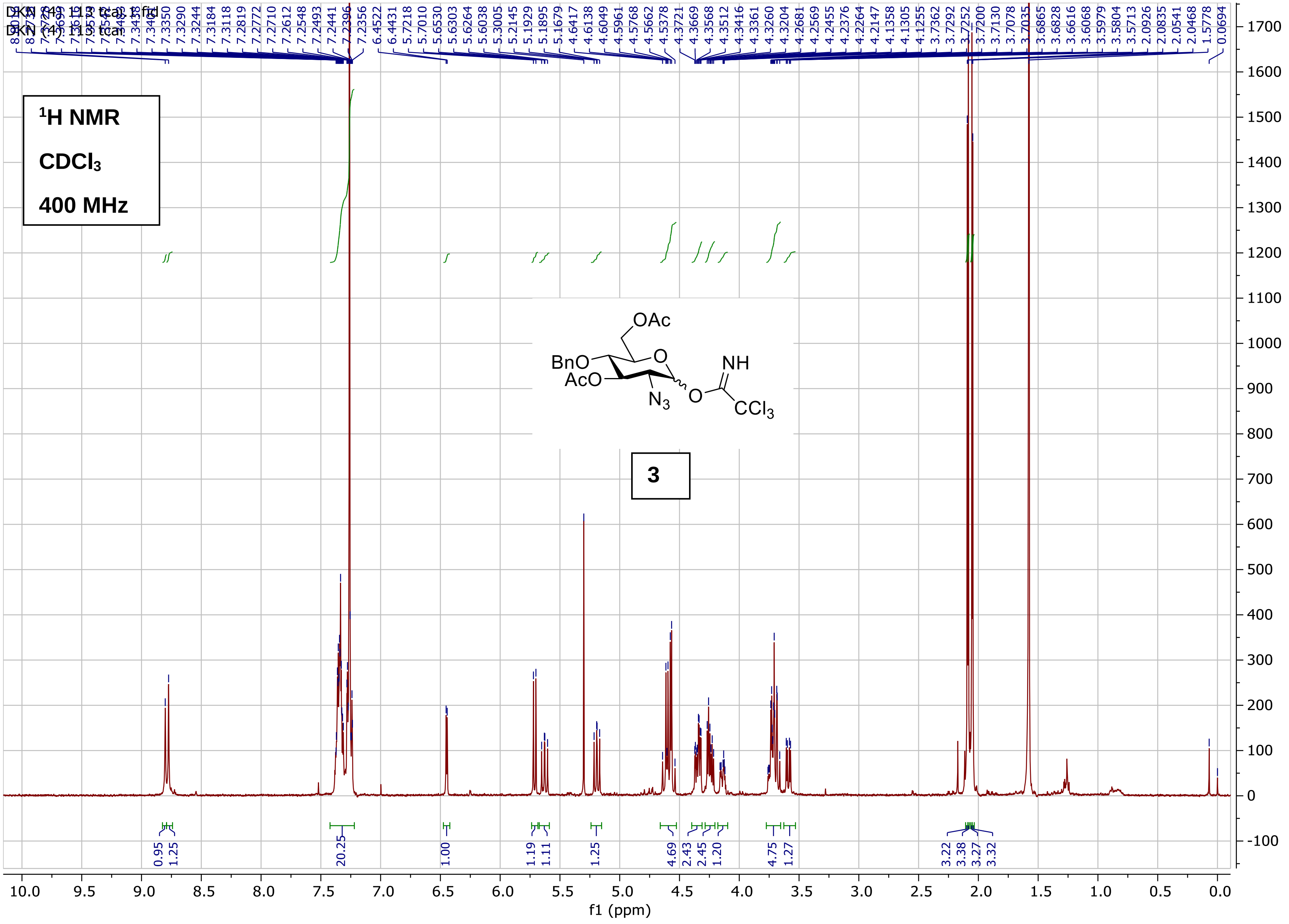

1H NMR
CDCl3
400 MHz
OAc
BnO
AcO
O
N3
NH
CCl3
3
f1 (ppm)

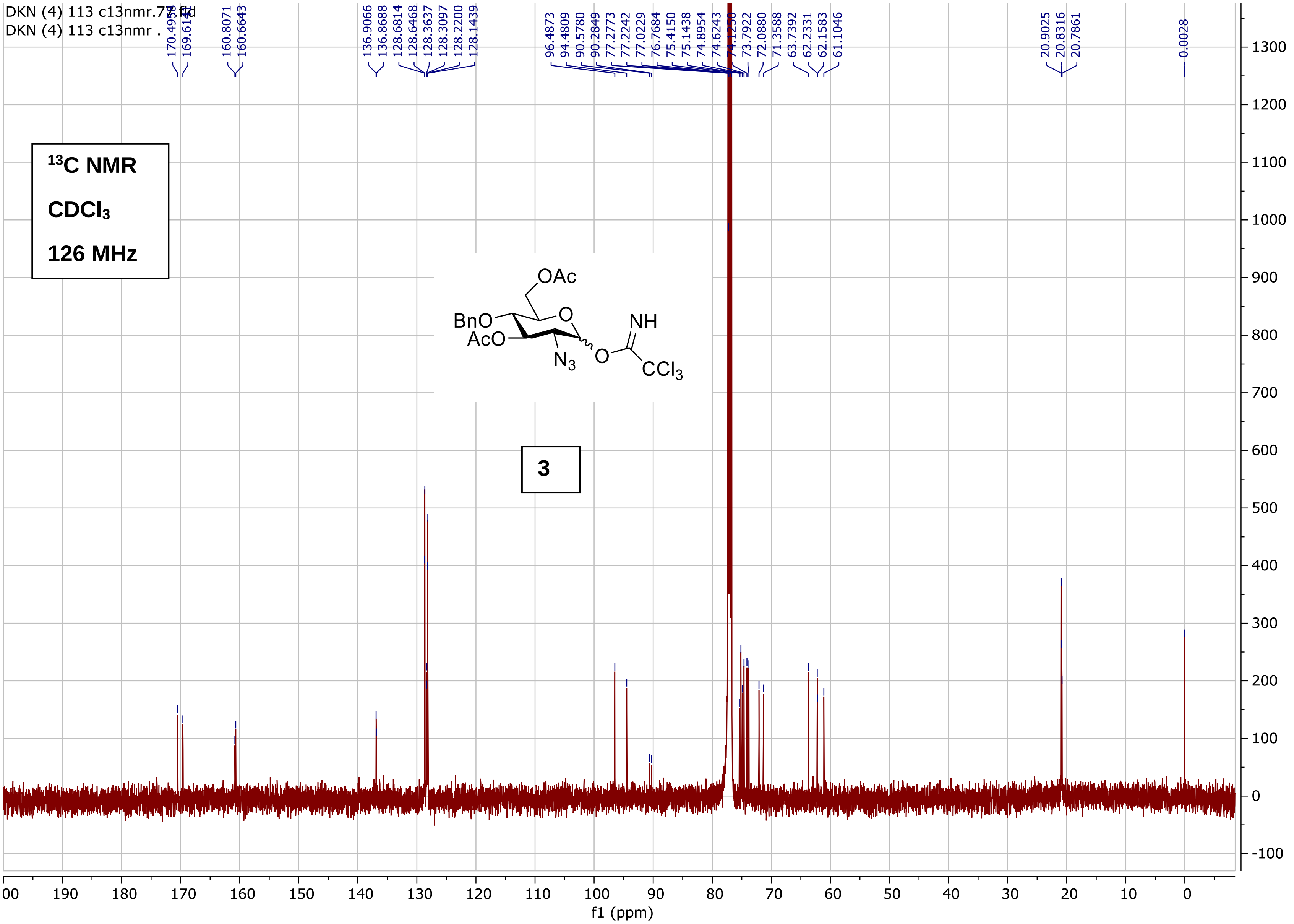

DKN (4) 113 c13nmr.77.fid
DKN (4) 113 c13nmr .
170.4958
169.6141
160.8071
160.6643
136.9066
136.8688
128.6814
128.6468
128.3637
128.3097
128.2200
128.1439
96.4873
94.4809
90.5780
90.2849
77.2773
77.2242
77.0229
76.7684
75.4150
75.1438
74.8954
74.6243
73.7922
72.0880
71.3588
63.7392
62.2331
62.1583
61.1046
20.9025
20.8316
20.7861
0.0028
^{13}C NMR
CDCl$_3$
126 MHz
OAc
BnO
AcO
O
N$_3$
O
NH
CCl$_3$
3
f1 (ppm)

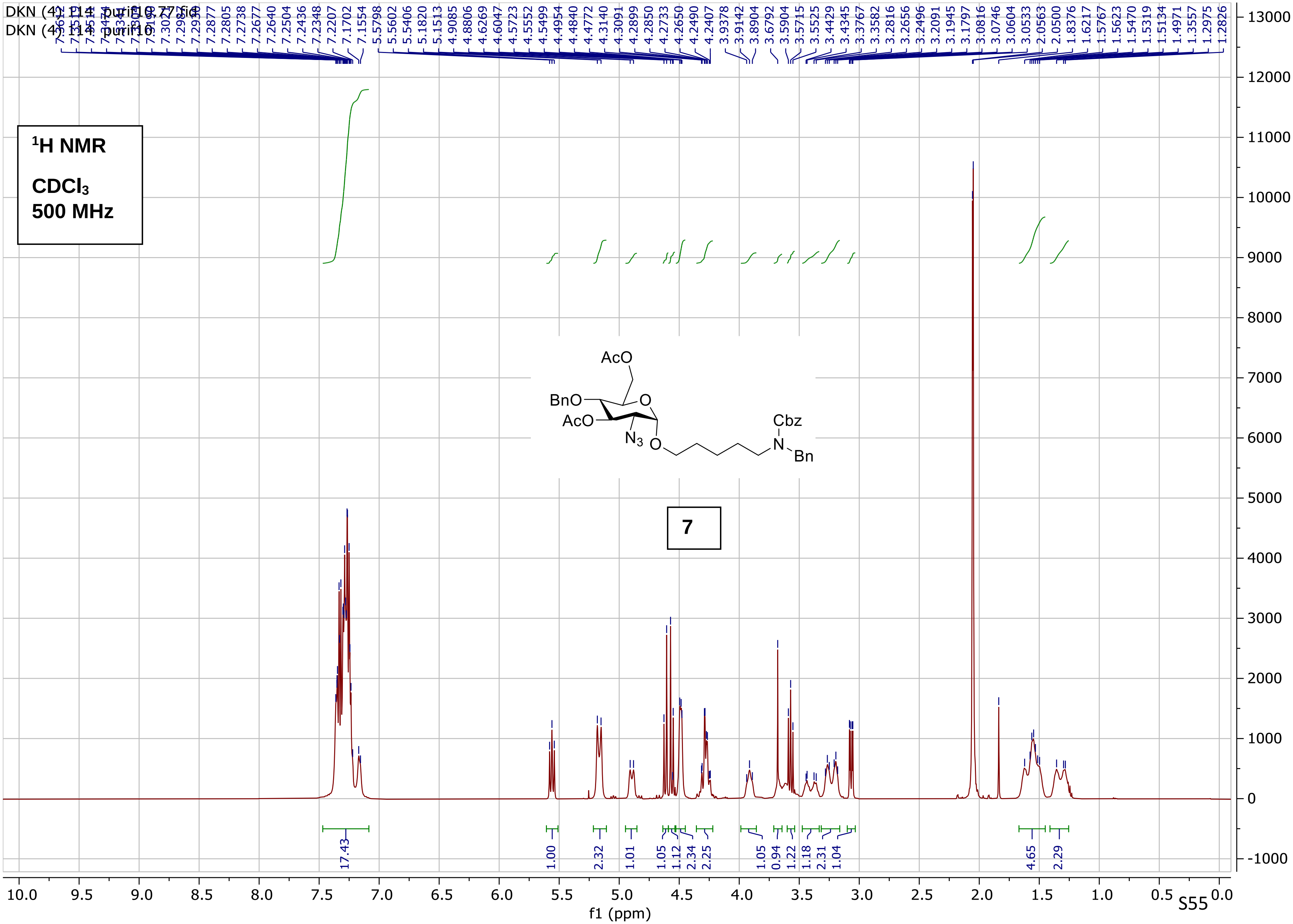
DKN (4) 114 purif10
1H NMR
CDCl3
500 MHz
AcO
BnO
AcO
N3
O
Cbz
N
Bn
7
17.43
1.00
2.32
1.01
1.05
1.12
2.34
2.25
1.05
0.94
1.22
1.18
2.31
1.04
4.65
2.29
f1 (ppm)

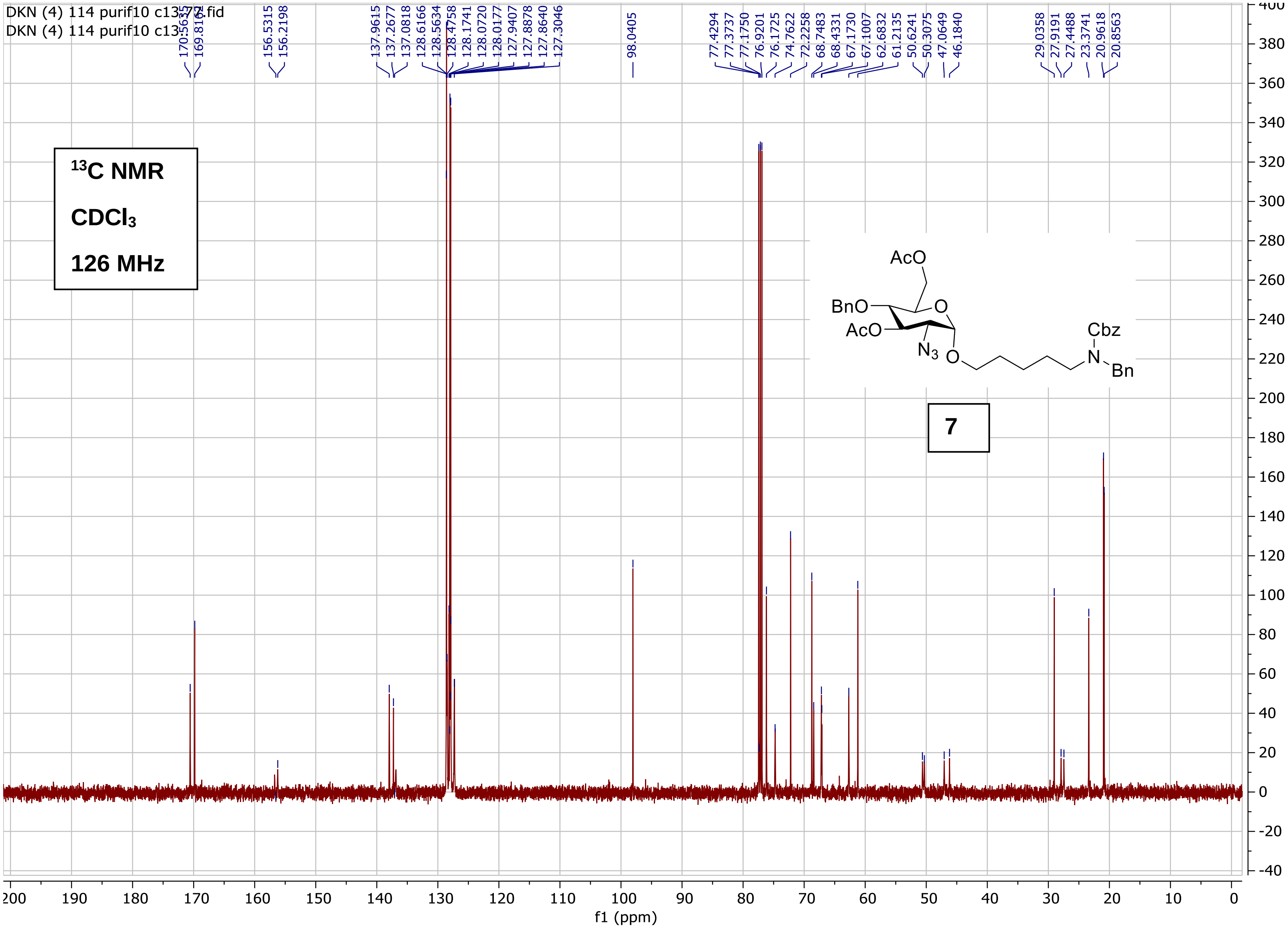
DKN (4) 114 purif10 c13-1.fid
DKN (4) 114 purif10 c13
170.5637
169.8167
156.5315
156.2198
137.9615
137.2677
137.0818
128.6166
128.5634
128.4758
128.1741
128.0720
128.0177
127.9407
127.8878
127.8640
127.3046
98.0405
77.4294
77.3737
77.1750
76.9201
76.1725
74.7622
72.2258
68.7483
68.4331
67.1730
67.1007
62.6832
61.2135
50.6241
50.3075
47.0649
46.1840
29.0358
27.9191
27.4488
23.3741
20.9618
20.8563
^{13}C NMR
$CDCl_3$
126 MHz
AcO
BnO
O
AcO
N_3
O
Cbz
N
Bn
7
f1 (ppm)

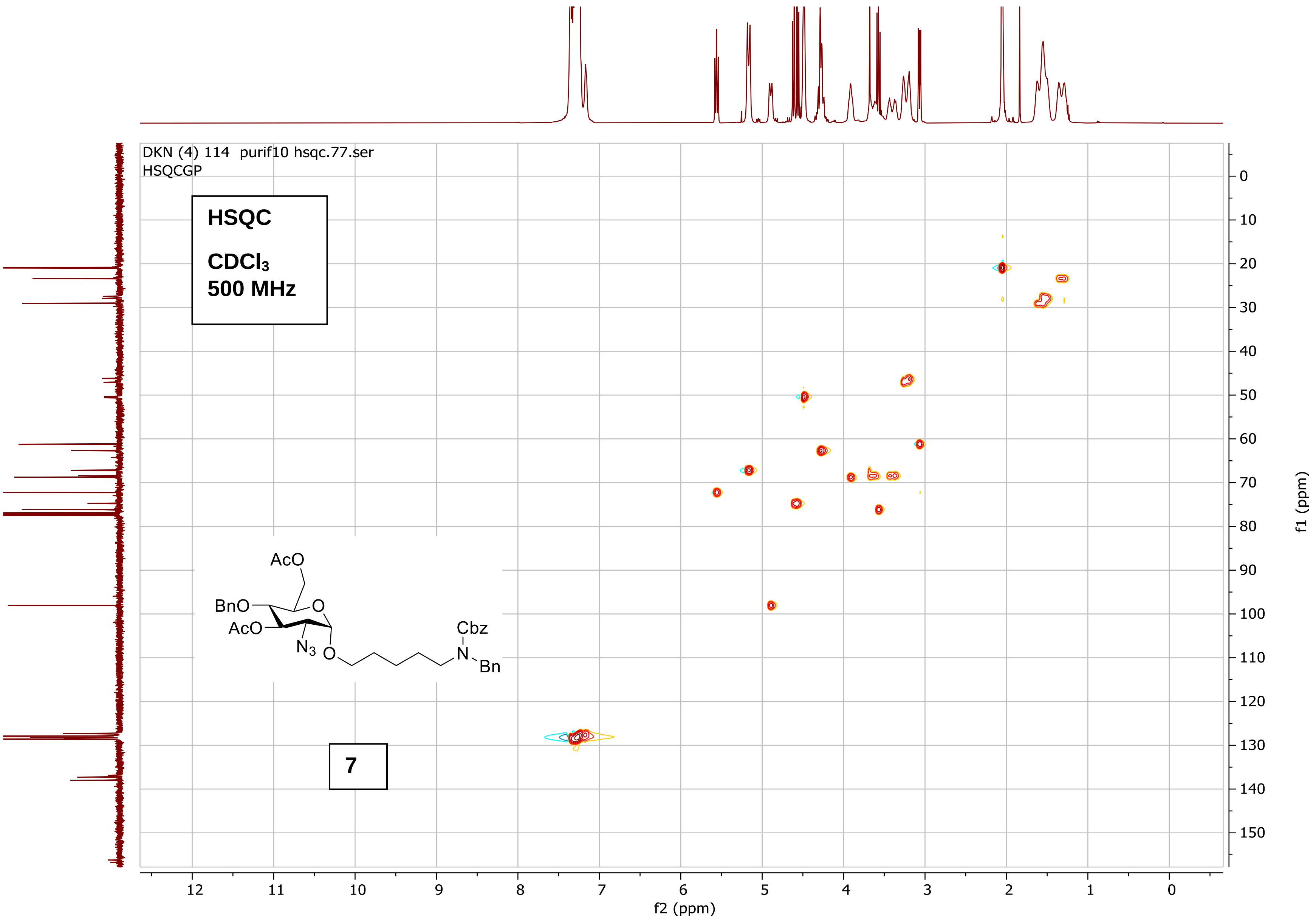

DKN (4) 114 purif10 hsqc.77.ser
HSQCGP
HSQC
CDCl3
500 MHz
AcO
BnO
O
AcO
N3
O
Cbz
N
Bn
7
f2 (ppm)
f1 (ppm)

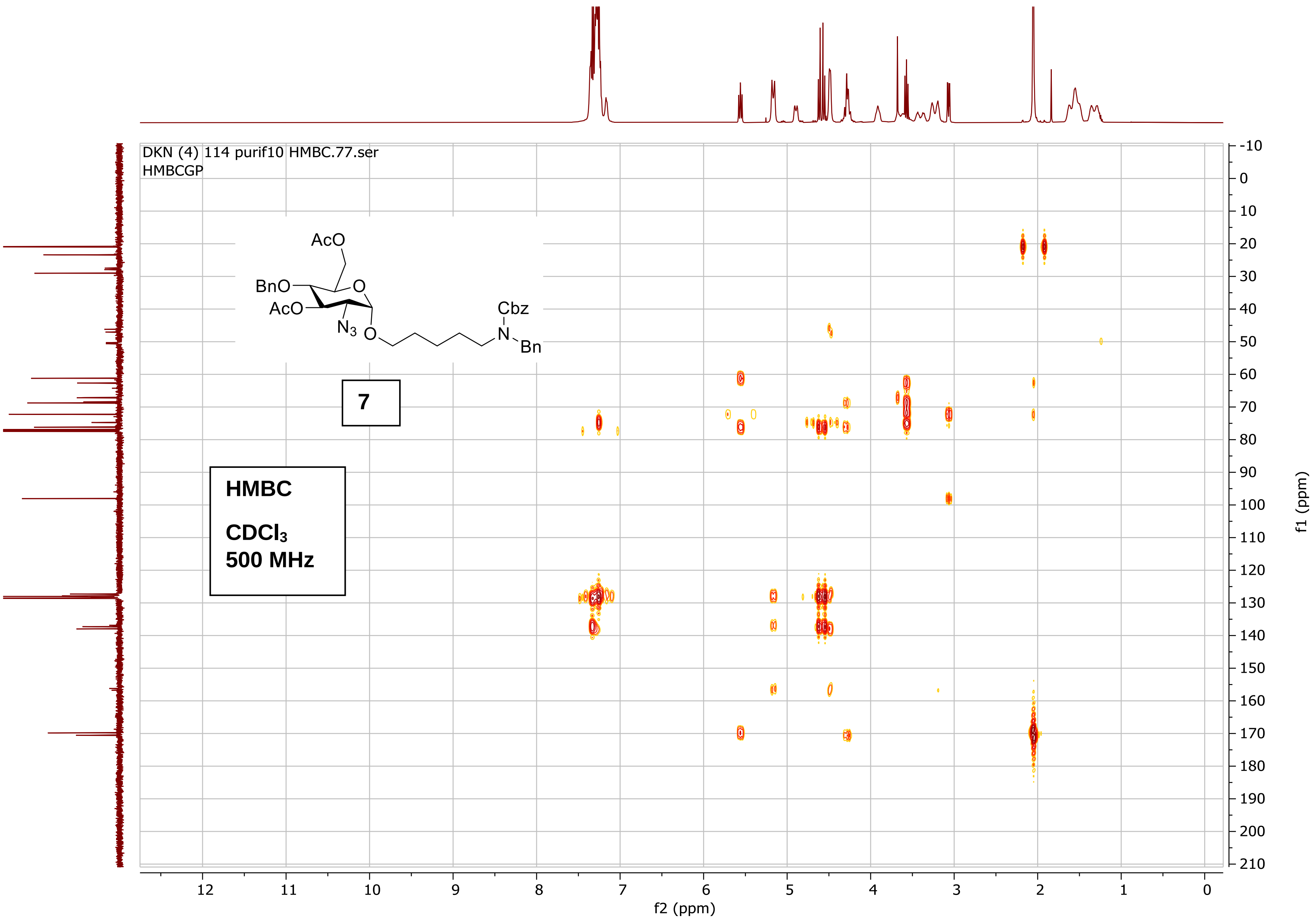
DKN (4) 114 purif10 HMBC.77.ser
HMBCGP
AcO
BnO
AcO
O
N3
O
Cbz
N
Bn
7
HMBC
CDCl3
500 MHz
f1 (ppm)
f2 (ppm)

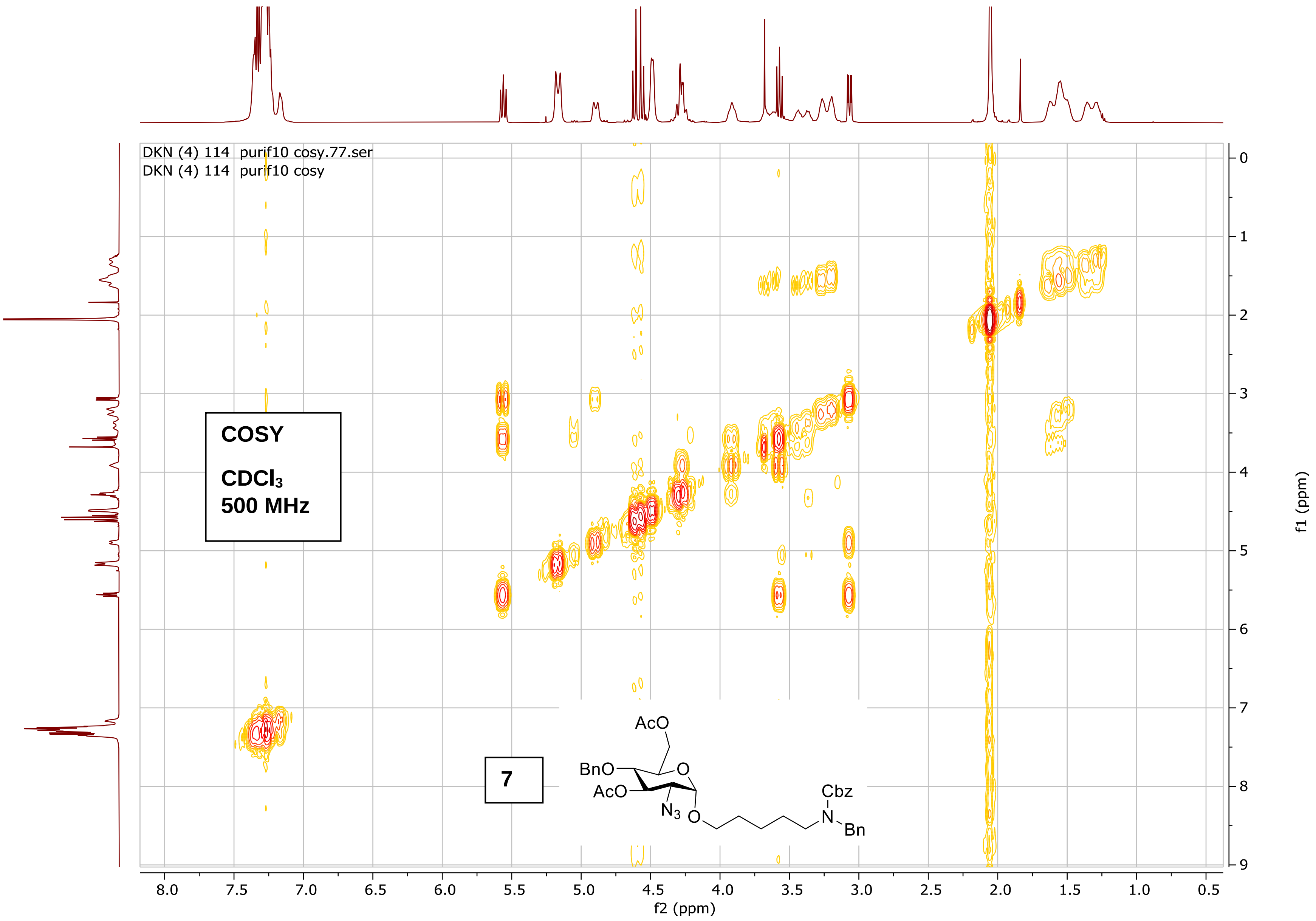
DKN (4) 114 purif10 cosy.77.ser
DKN (4) 114 purif10 cosy
COSY
$CDCl_3$
500 MHz
7
AcO
BnO
O
AcO
N_3
O
Cbz
N
Bn
f2 (ppm)
f1 (ppm)

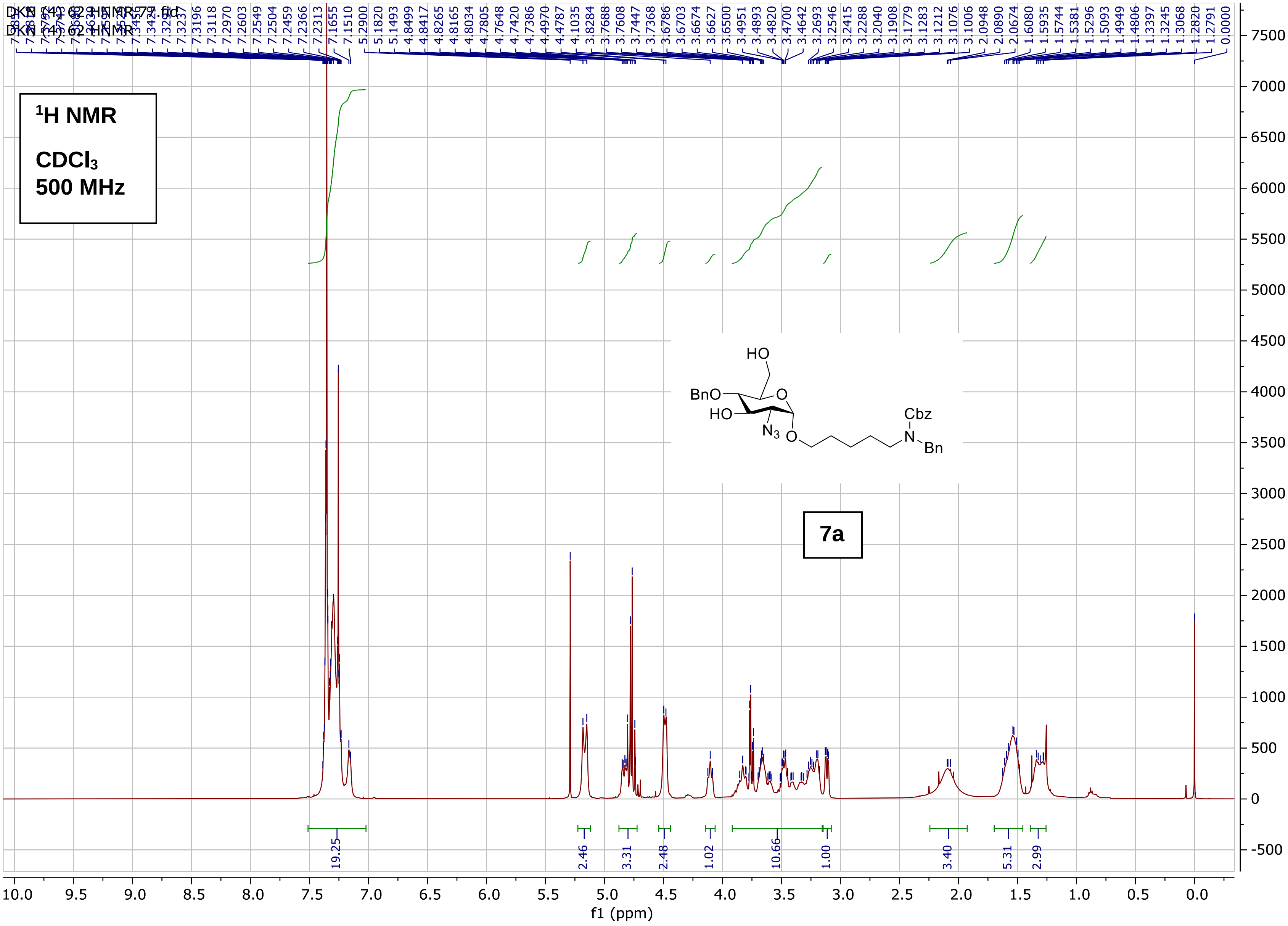

DKN (4) 62 HNMR
¹H NMR
CDCl3
500 MHz
HO
BnO
O
HO
N3
O
Cbz
N
Bn
7a
19.25
2.46
3.31
2.48
1.02
10.66
1.00
3.40
5.31
2.99
f1 (ppm)

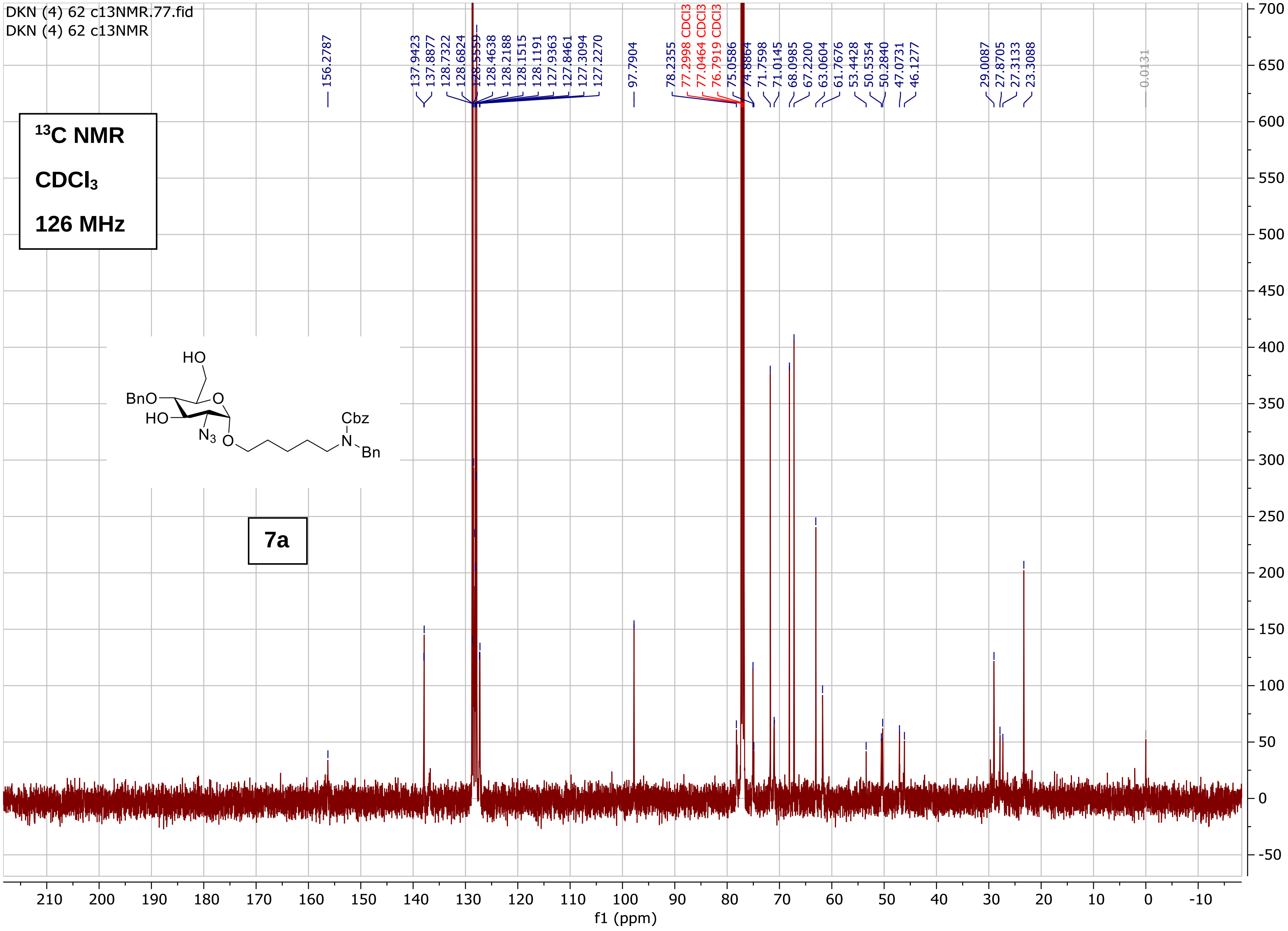
DKN (4) 62 c13NMR.77.fid
DKN (4) 62 c13NMR
^{13}C NMR
$CDCl_3$
126 MHz
156.2787
137.9423
137.8877
128.7322
128.6824
128.5559
128.4638
128.2188
128.1515
128.1191
127.9363
127.8461
127.3094
127.2270
97.7904
78.2355
77.2998 CDCl3
77.0464 CDCl3
76.7919 CDCl3
75.0586
74.8864
71.7598
71.0145
68.0985
67.2200
63.0604
61.7676
53.4428
50.5354
50.2840
47.0731
46.1277
29.0087
27.8705
27.3133
23.3088
0.0131
HO
BnO
HO
O
N_3
O
Cbz
N
Bn
f1 (ppm)

7a

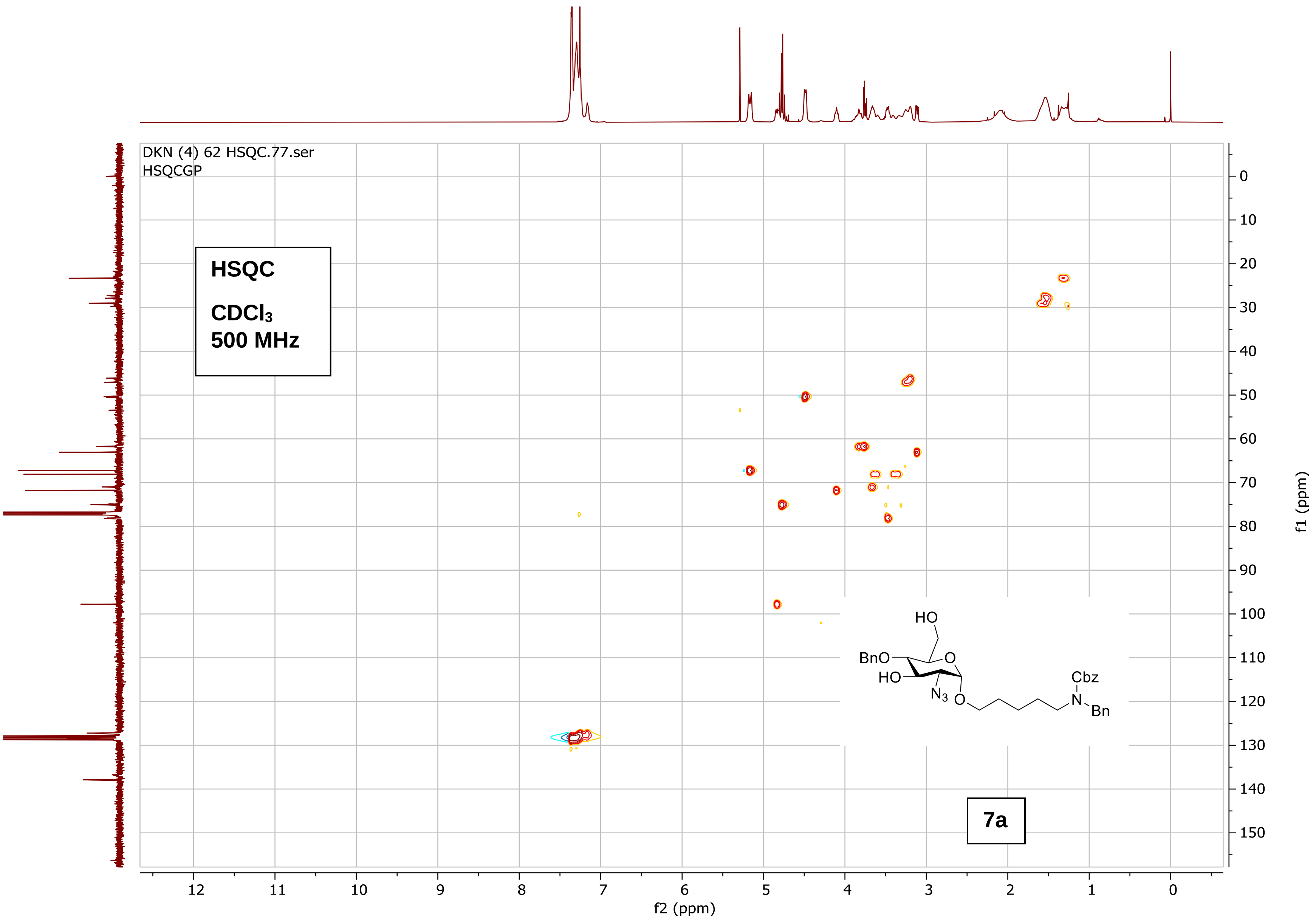
DKN (4) 62 HSQC.77.ser
HSQCGP
HSQC
$CDCl_3$
500 MHz
HO
BnO
O
HO
N_3
O
Cbz
N
Bn
7a
f2 (ppm)
f1 (ppm)

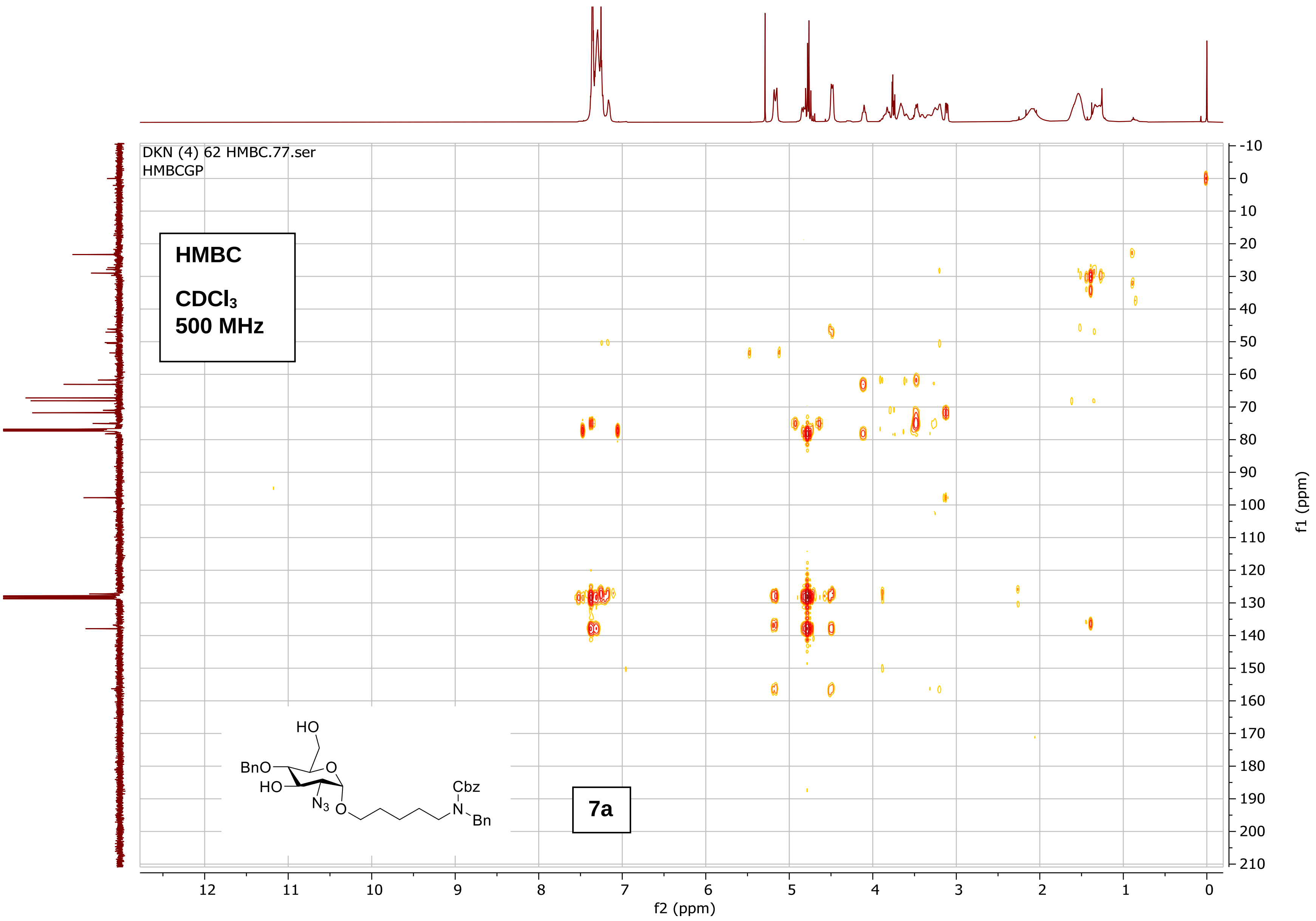

DKN (4) 62 HMBC.77.ser
HMBCGP
HMBC
$CDCl_3$
500 MHz
HO
BnO
O
HO
N_3
O
Cbz
N
Bn
7a
f2 (ppm)
f1 (ppm)

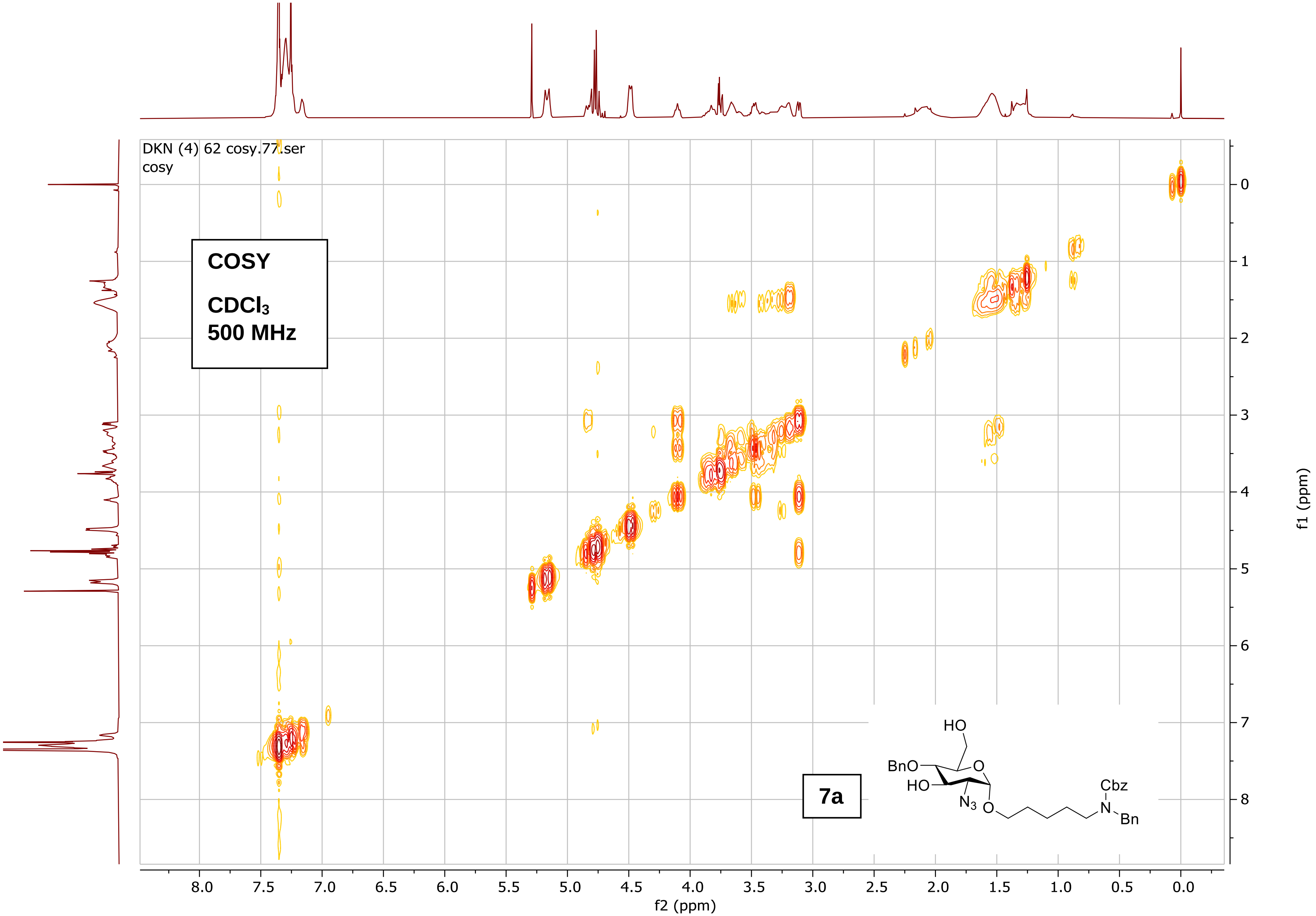
DKN (4) 62 cosy.77.ser
cosy
COSY
CDCl3
500 MHz
7a
HO
BnO
O
HO
N3
O
Cbz
N
Bn
f2 (ppm)
f1 (ppm)

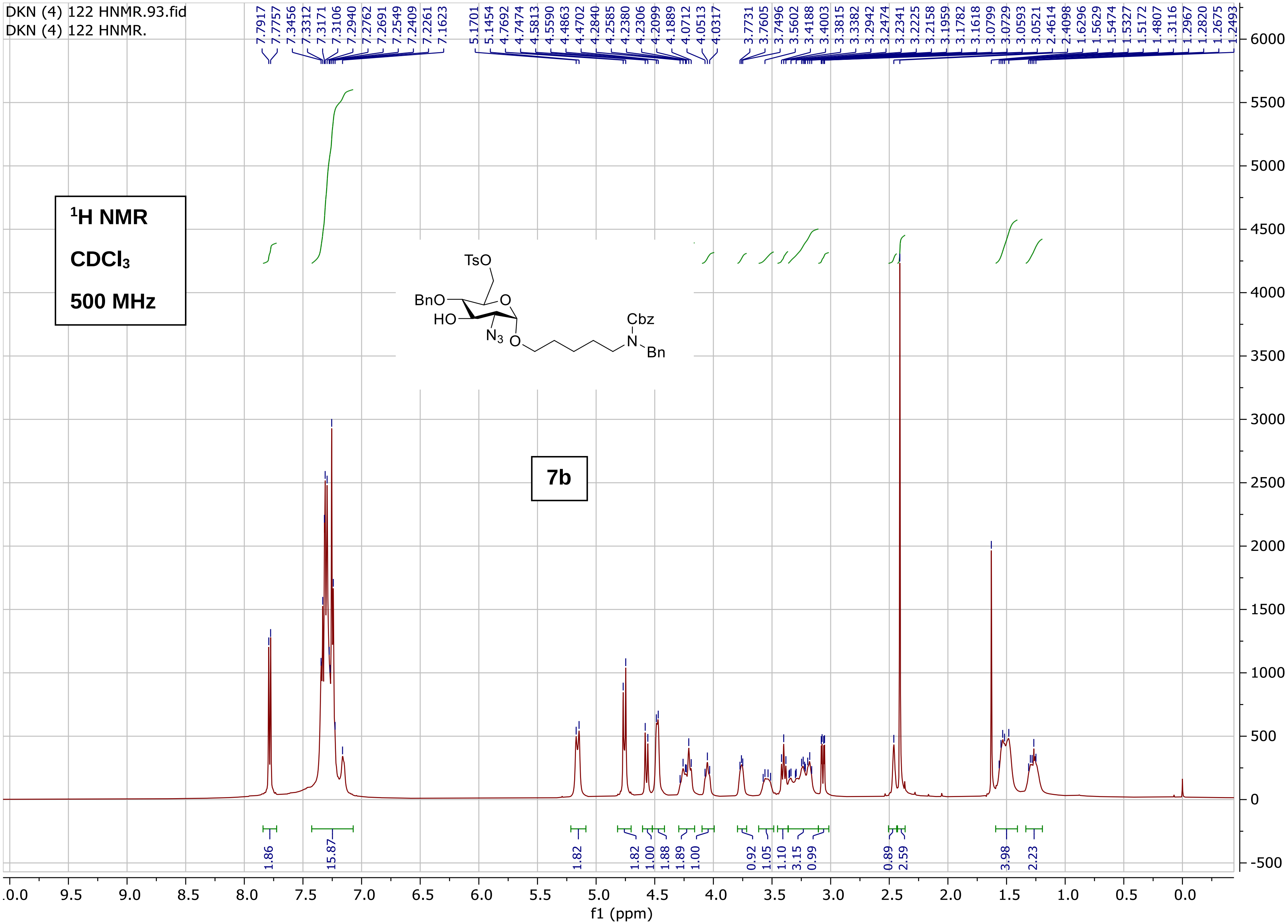
DKN (4) 122 HNMR.93.fid
DKN (4) 122 HNMR.
¹H NMR
CDCl3
500 MHz
TsO
BnO
HO
N3
O
Cbz
N
Bn
7b
7.7917 7.7757 7.3456 7.3312 7.3171 7.3106 7.2940 7.2762 7.2691 7.2549 7.2409 7.2261 7.1623
5.1701 5.1454 4.7692 4.7474 4.5813 4.5590 4.4863 4.4702 4.2840 4.2585 4.2380 4.2306 4.2099 4.1889 4.0712 4.0513 4.0317
3.7731 3.7605 3.7496 3.5602 3.4188 3.4003 3.3815 3.3382 3.2942 3.2474 3.2341 3.2225 3.2158 3.1959 3.1782 3.1618 3.0799 3.0729 3.0593 3.0521 2.4614 2.4098 1.6296 1.5629 1.5474 1.5327 1.5172 1.4807 1.3116 1.2967 1.2820 1.2675 1.2493
1.86 15.87 1.82 1.82 1.00 1.88 1.89 1.00 0.92 1.05 1.10 3.15 0.99 0.89 2.59 3.98 2.23
f1 (ppm)

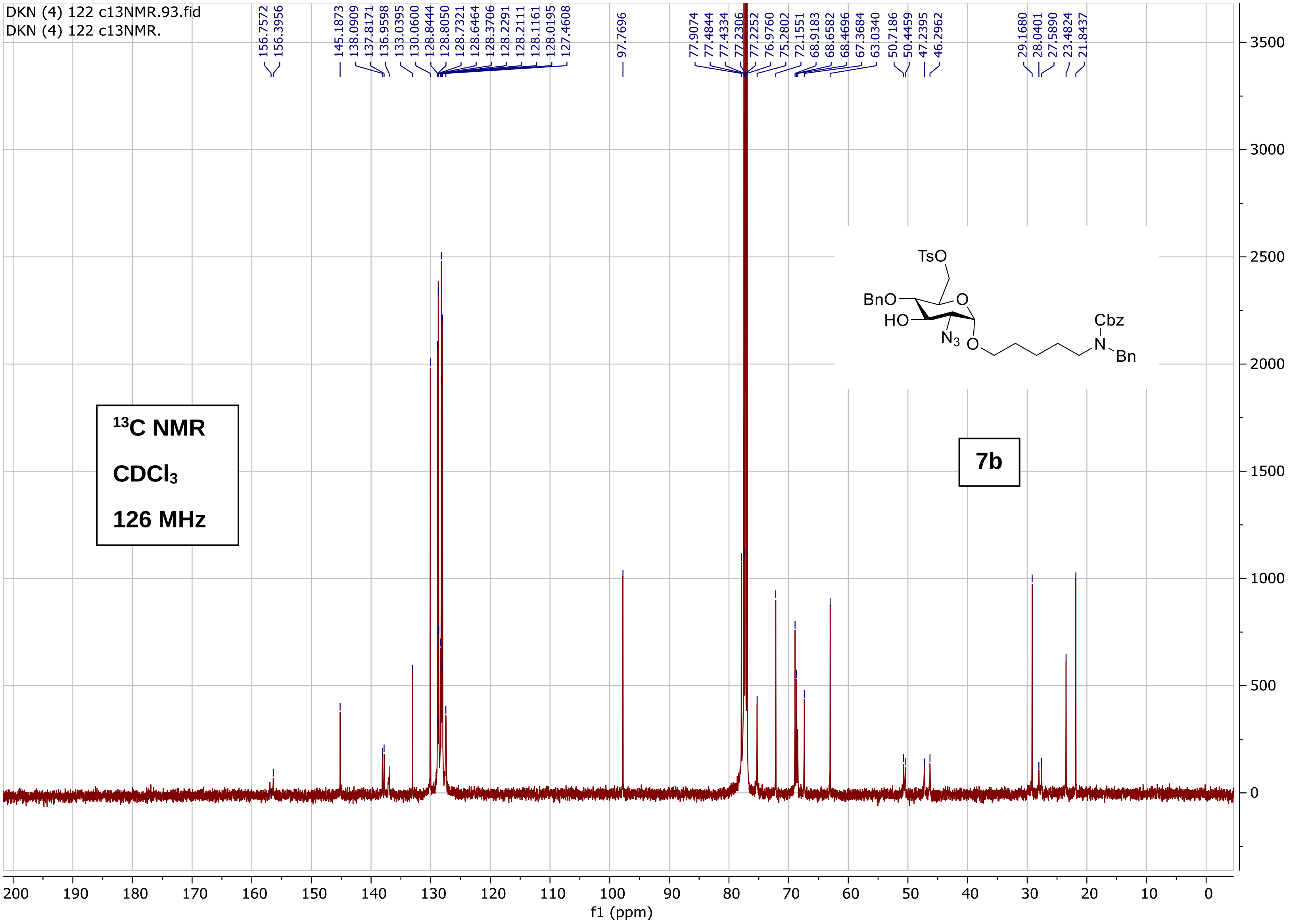
DKN (4) 122 c13NMR.93.fid
DKN (4) 122 c13NMR.
156.7572
156.3956
145.1873
138.0909
137.8171
136.9598
133.0395
130.0600
128.8444
128.8050
128.7321
128.6464
128.3706
128.2291
128.2111
128.1161
128.0195
127.4608
97.7696
77.9074
77.4844
77.4334
77.2306
77.2252
76.9760
75.2802
72.1551
68.9183
68.6582
68.4696
67.3684
63.0340
50.7186
50.4459
47.2395
46.2962
29.1680
28.0401
27.5890
23.4824
21.8437
TsO
BnO
O
HO
N_3
O
Cbz
N
Bn
^{13}C NMR
$CDCl_3$
126 MHz
7b
f1 (ppm)

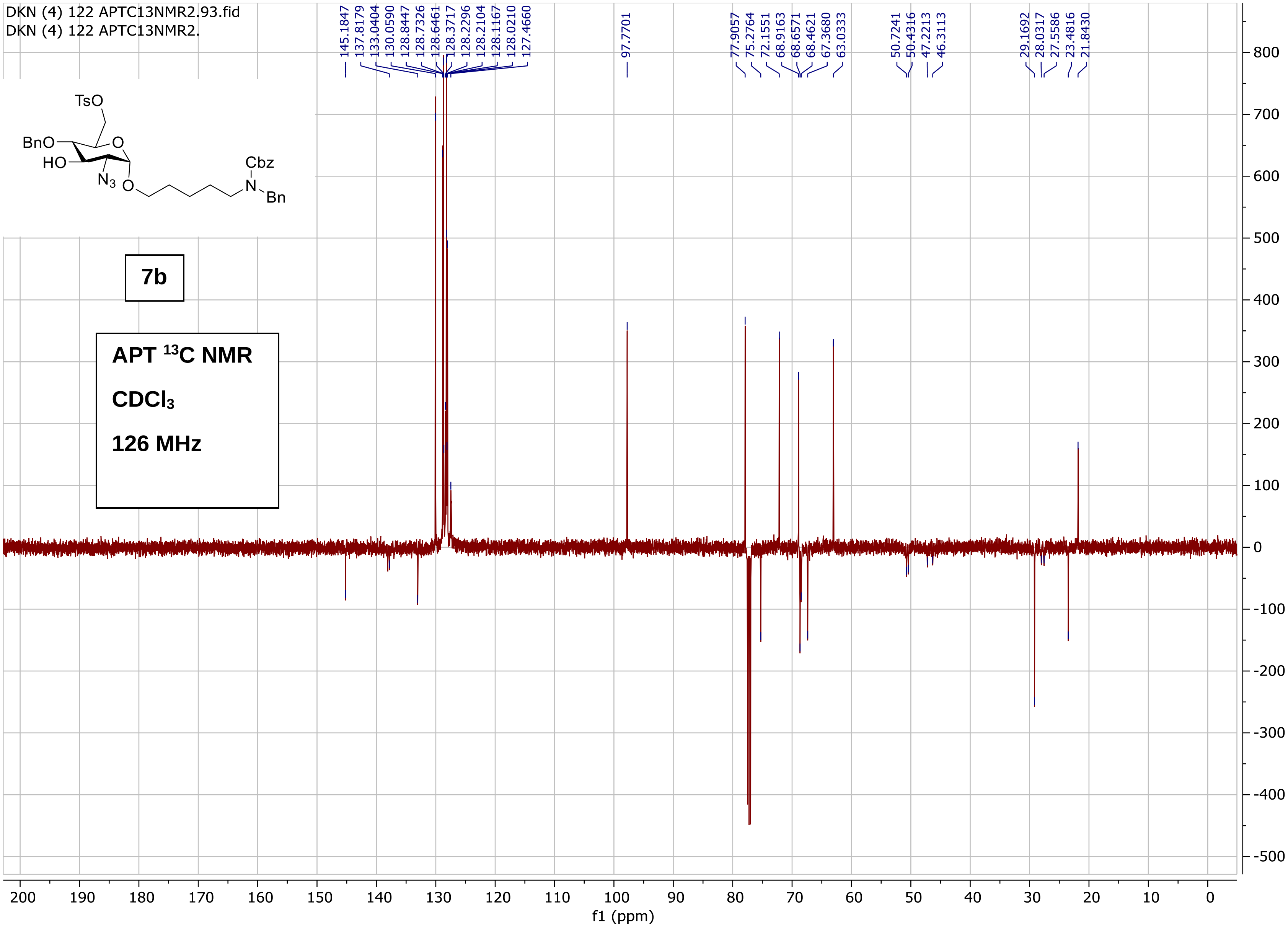

DKN (4) 122 APTC13NMR2.93.fid
DKN (4) 122 APTC13NMR2.
145.1847
137.8179
133.0404
130.0590
128.8447
128.7326
128.6461
128.3717
128.2296
128.2104
128.1167
128.0210
127.4660
97.7701
77.9057
75.2764
72.1551
68.9163
68.6571
68.4621
67.3680
63.0333
50.7241
50.4316
47.2213
46.3113
29.1692
28.0317
27.5586
23.4816
21.8430
TsO
BnO
O
HO
N_3
O
Cbz
N
Bn
7b
APT ^{13}C NMR
$CDCl_3$
126 MHz
f1 (ppm)

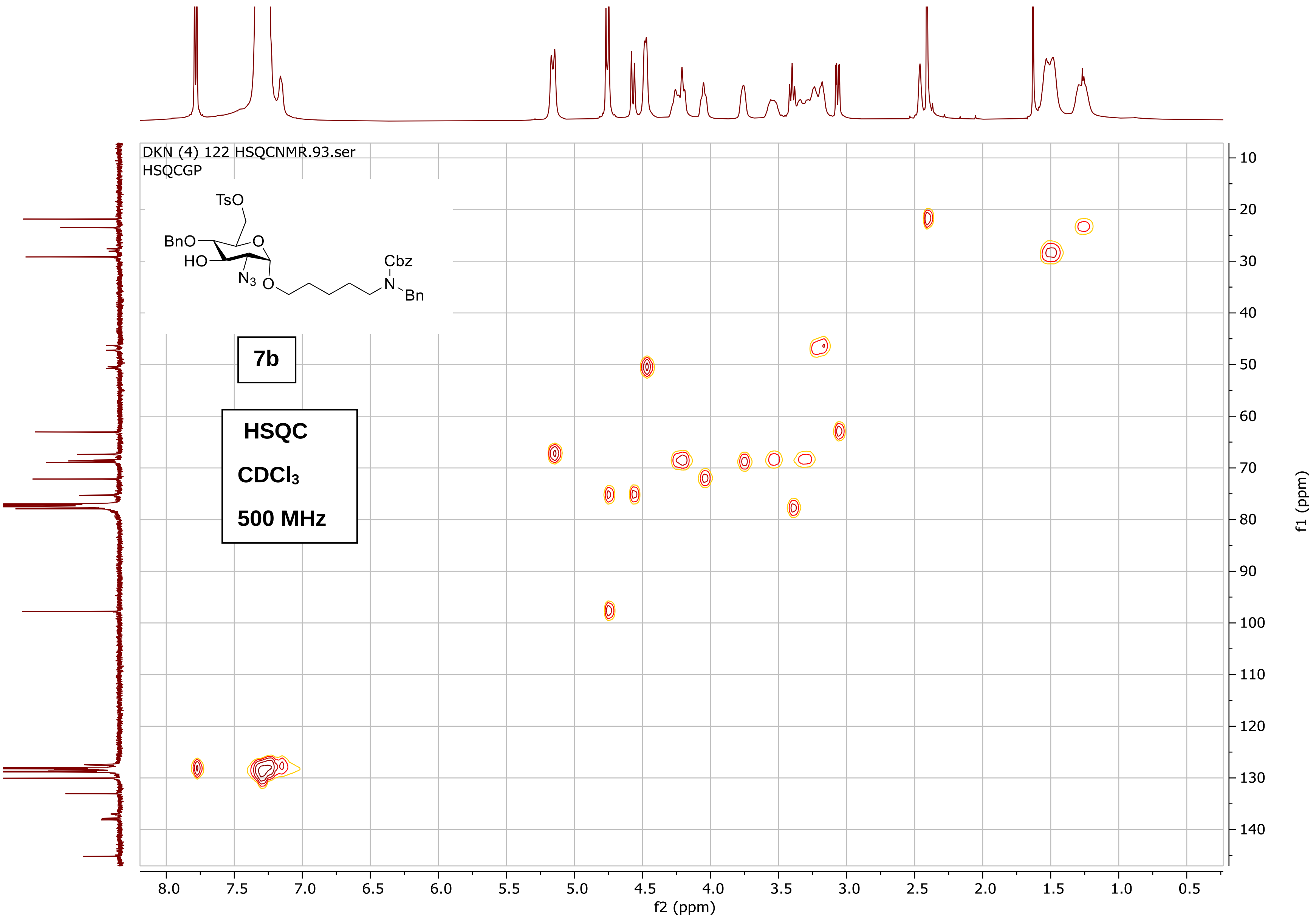
DKN (4) 122 HSQCNMR.93.ser
HSQCGP
TsO
BnO
HO
N3
O
O
Cbz
N
Bn
7b
HSQC
CDCl3
500 MHz
f2 (ppm)
f1 (ppm)

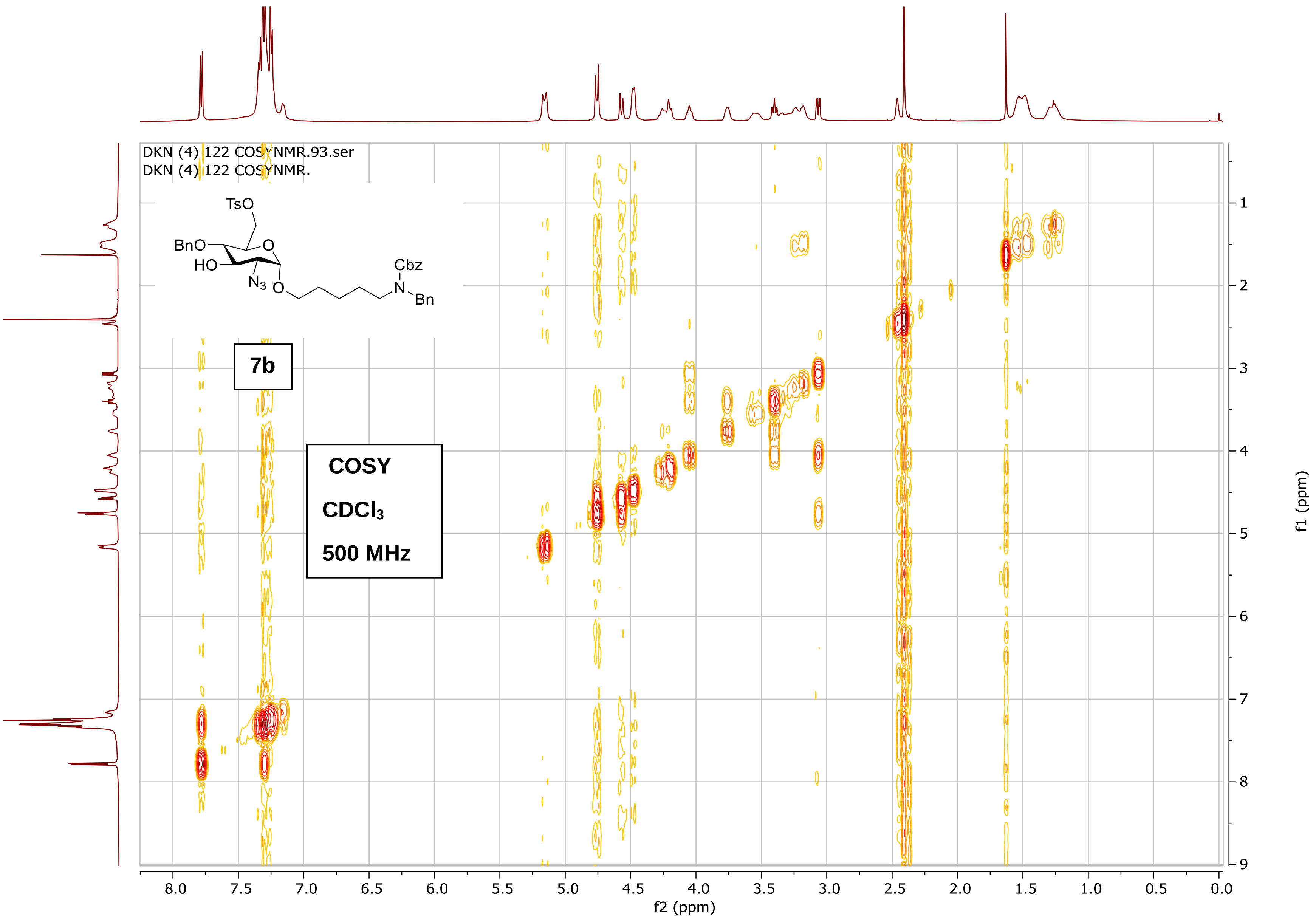
DKN (4) 122 COSYNMR.93.ser
DKN (4) 122 COSYNMR.
TsO
BnO
O
HO
N3
O
Cbz
N
Bn
7b
COSY
CDCl3
500 MHz
f2 (ppm)
f1 (ppm)

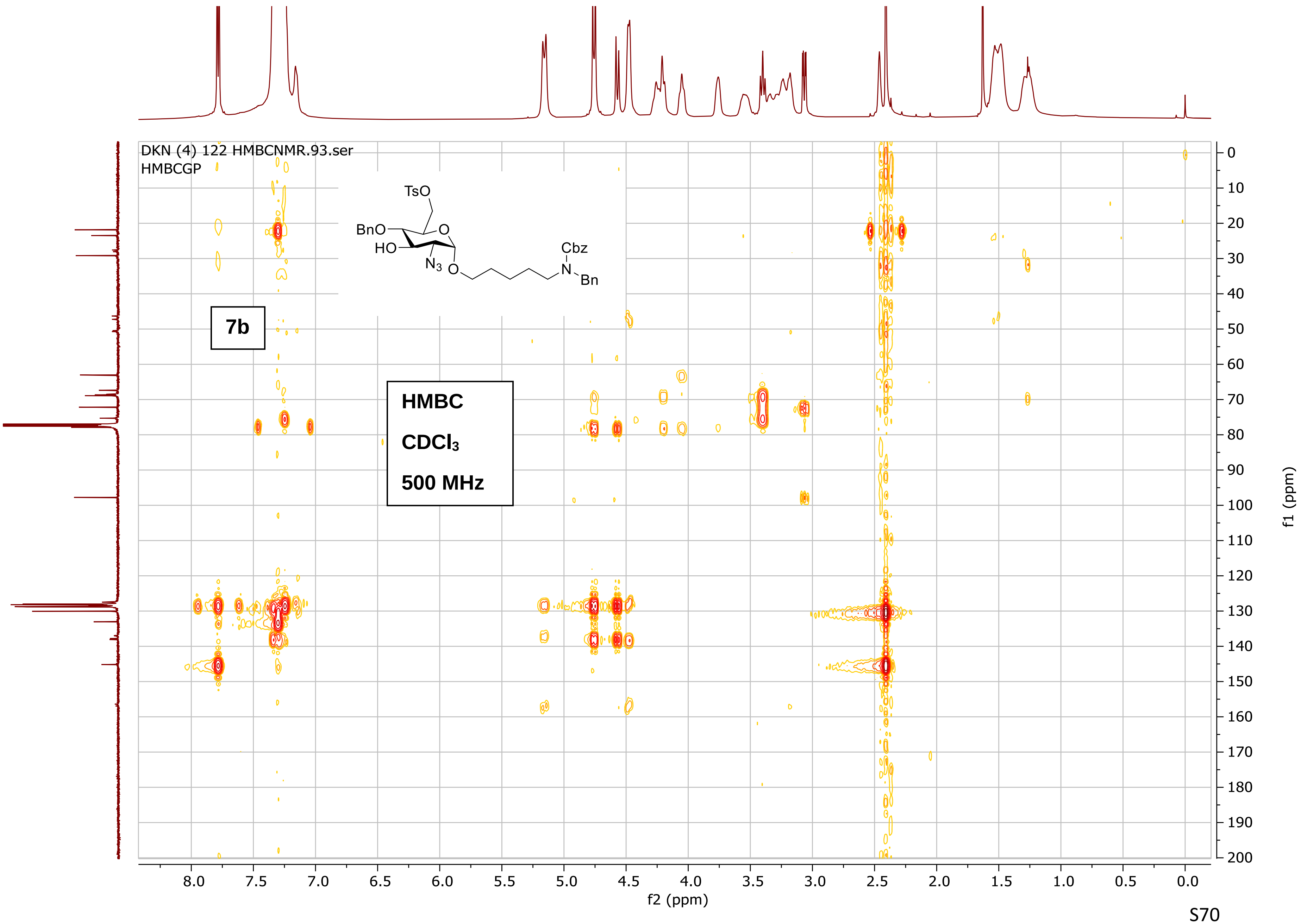
DKN (4) 122 HMBCNMR.93.ser
HMBCGP
TsO
BnO
HO
O
N3
O
Cbz
N
Bn
7b
HMBC
CDCl3
500 MHz
f2 (ppm)
f1 (ppm)

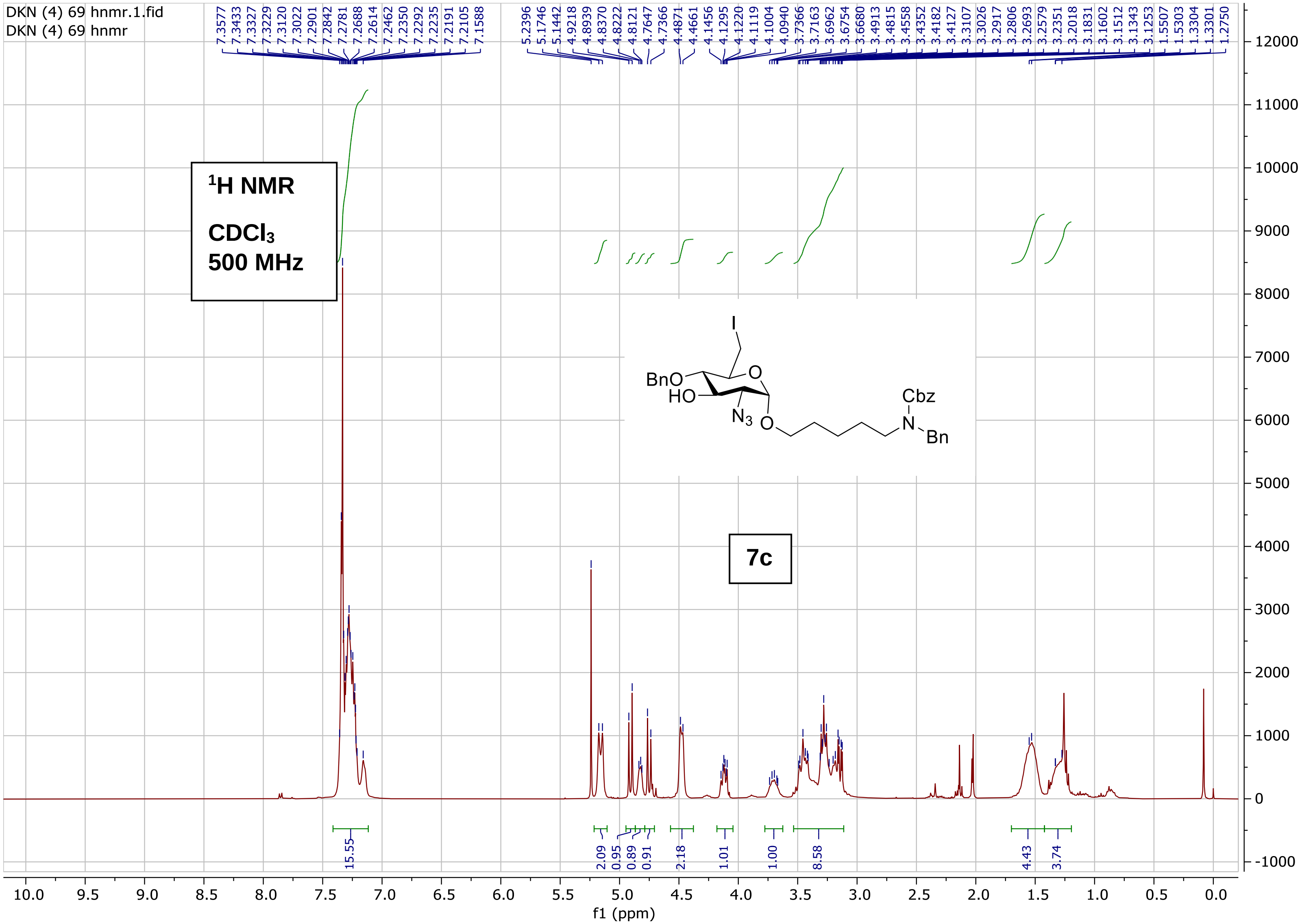

DKN (4) 69 hnmr.1.fid
DKN (4) 69 hnmr
¹H NMR
CDCl₃
500 MHz
7c
BnO
HO
N₃
O
I
Cbz
N
Bn
f1 (ppm)

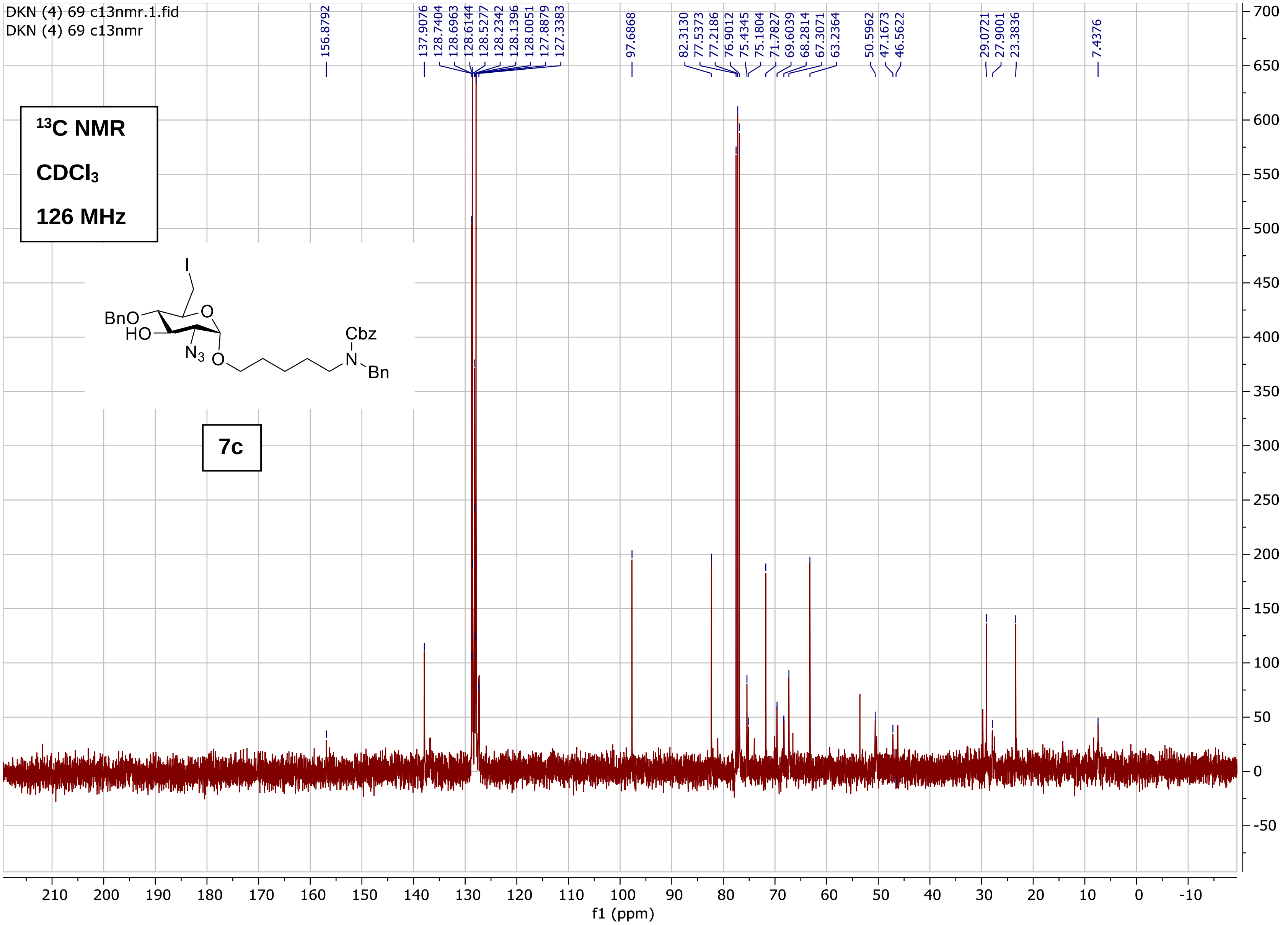
DKN (4) 69 c13nmr.1.fid
DKN (4) 69 c13nmr
13C NMR
CDCl3
126 MHz
7c
156.8792
137.9076
128.7404
128.6963
128.6144
128.5277
128.2342
128.1396
128.0051
127.8879
127.3383
97.6868
82.3130
77.5373
77.2186
76.9012
75.4345
75.1804
71.7827
69.6039
68.2814
67.3071
63.2364
50.5962
47.1673
46.5622
29.0721
27.9001
23.3836
7.4376
f1 (ppm)

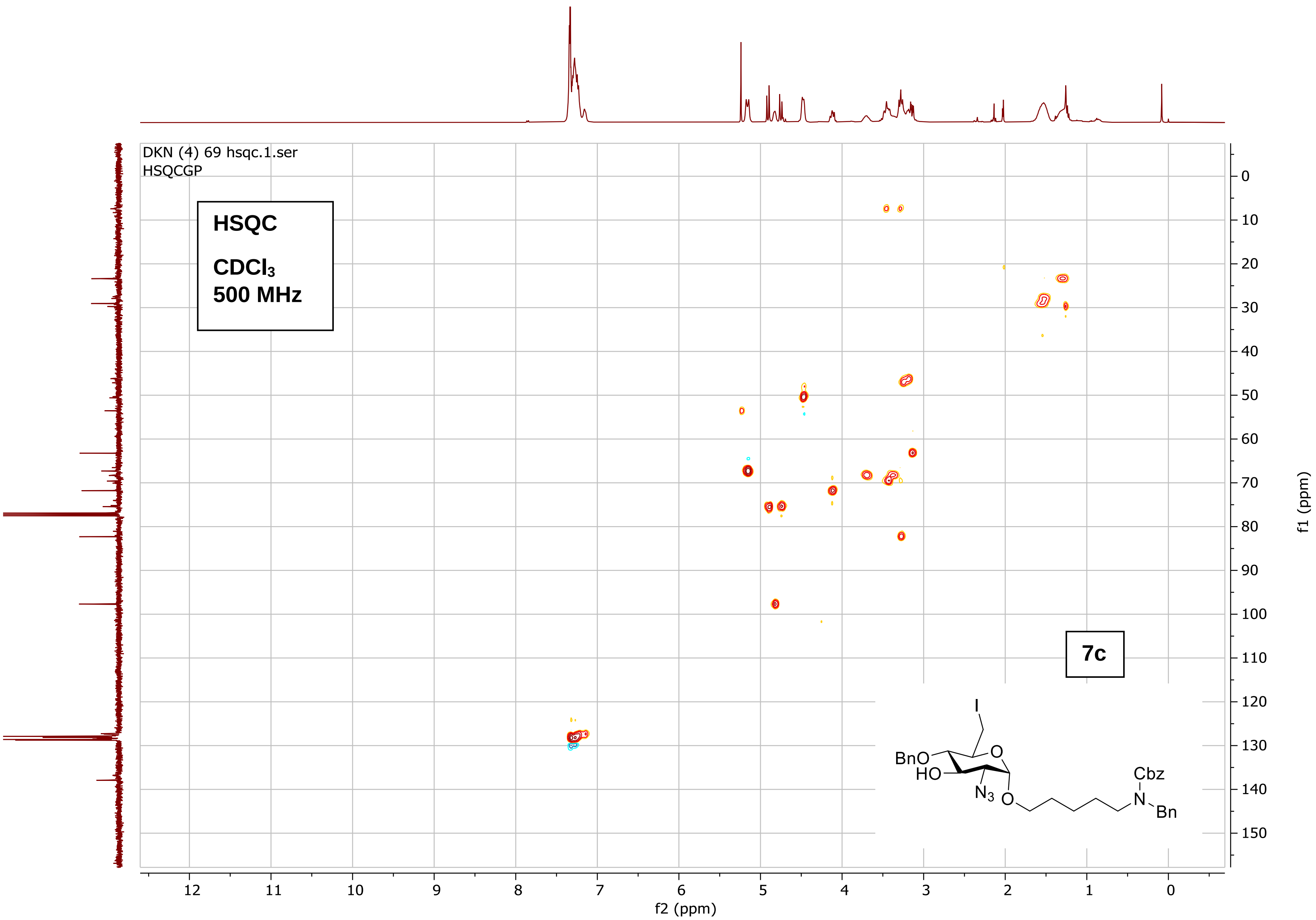
DKN (4) 69 hsqc.1.ser
HSQCGP
HSQC
CDCl3
500 MHz
7c
BnO
HO
O
N3
O
I
Cbz
N
Bn
f2 (ppm)
f1 (ppm)

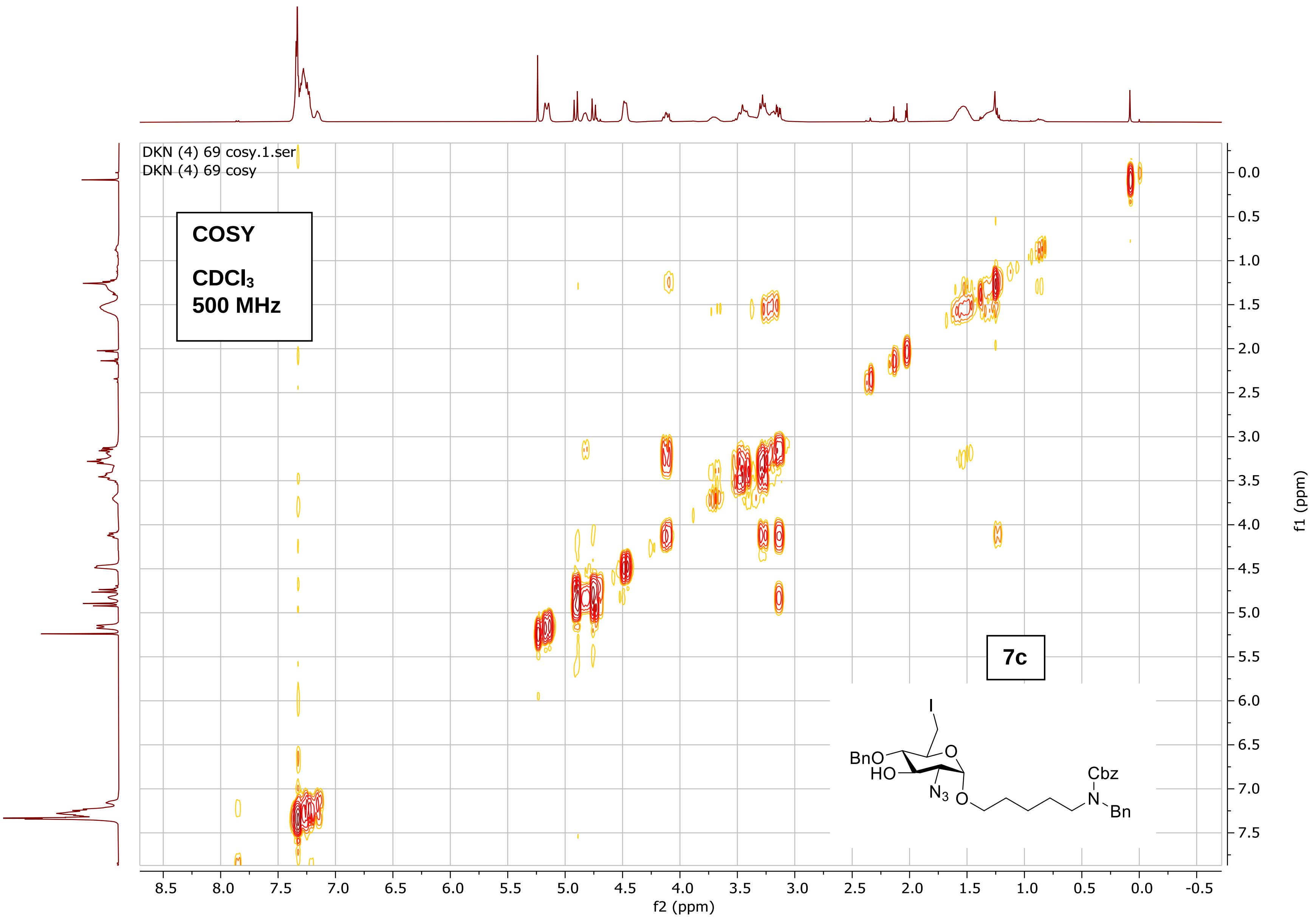
DKN (4) 69 cosy.1.ser
DKN (4) 69 cosy
COSY
CDCl3
500 MHz
7c
BnO
HO
N3
O
I
Cbz
N
Bn
f2 (ppm)
f1 (ppm)

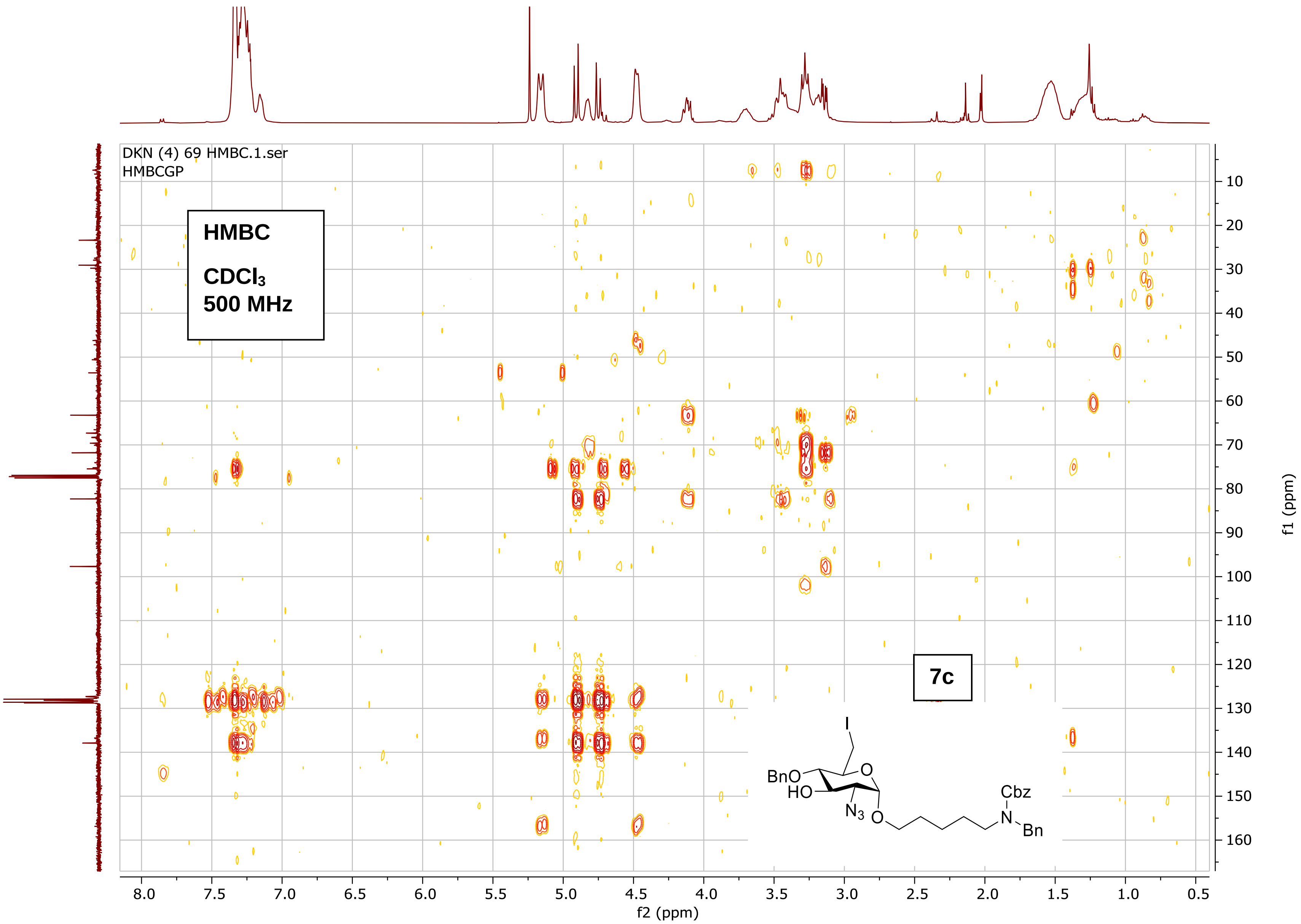

DKN (4) 69 HMBC.1.ser
HMBCGP
HMBC
CDCl3
500 MHz
7c
I
O
BnO
HO
N3
O
Cbz
N
Bn
f2 (ppm)
f1 (ppm)

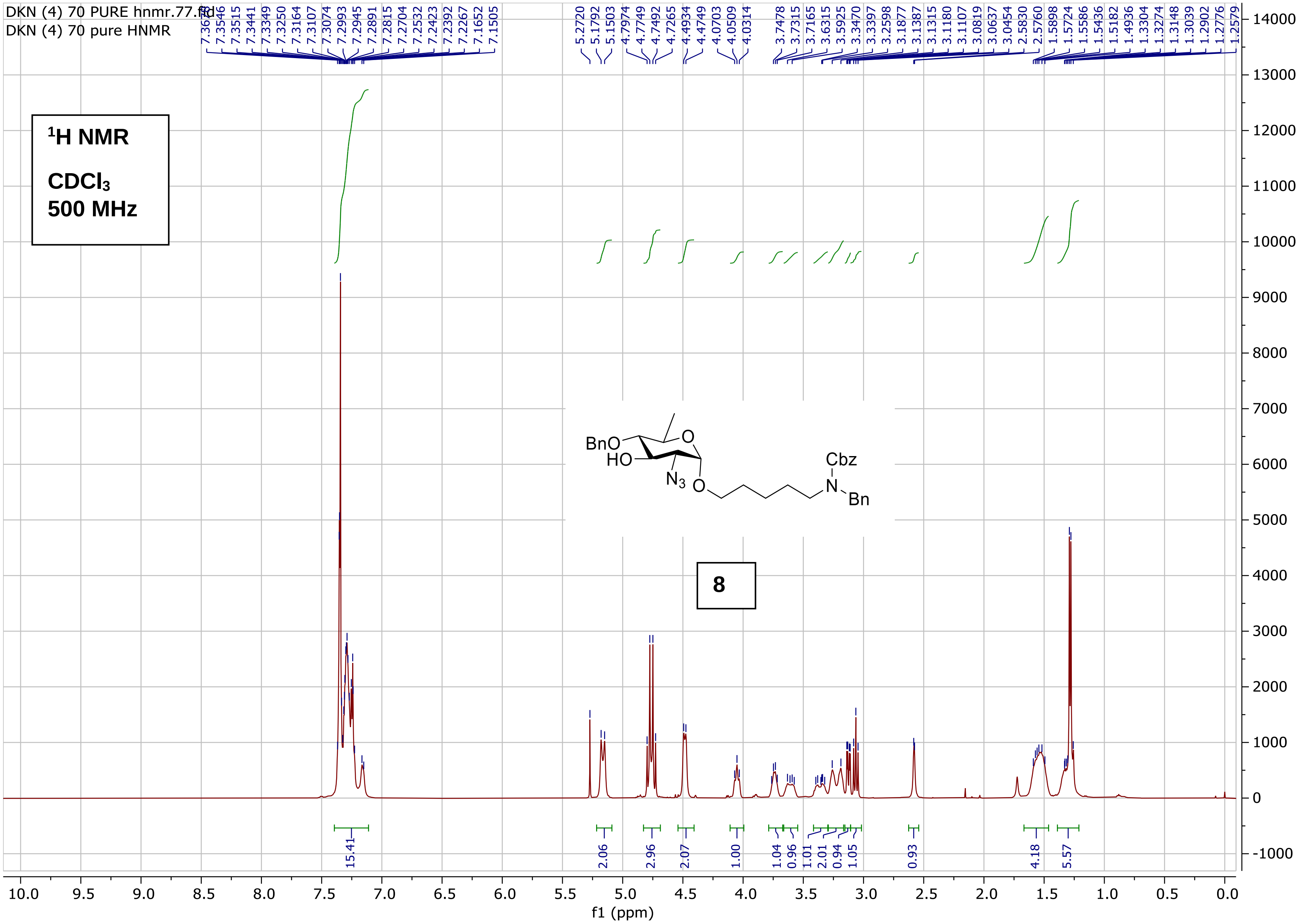
DKN (4) 70 PURE hnmr.77.fid
DKN (4) 70 pure HNMR
¹H NMR
CDCl₃
500 MHz
BnO
HO
N₃
O
O
Cbz
N
Bn
8
f1 (ppm)

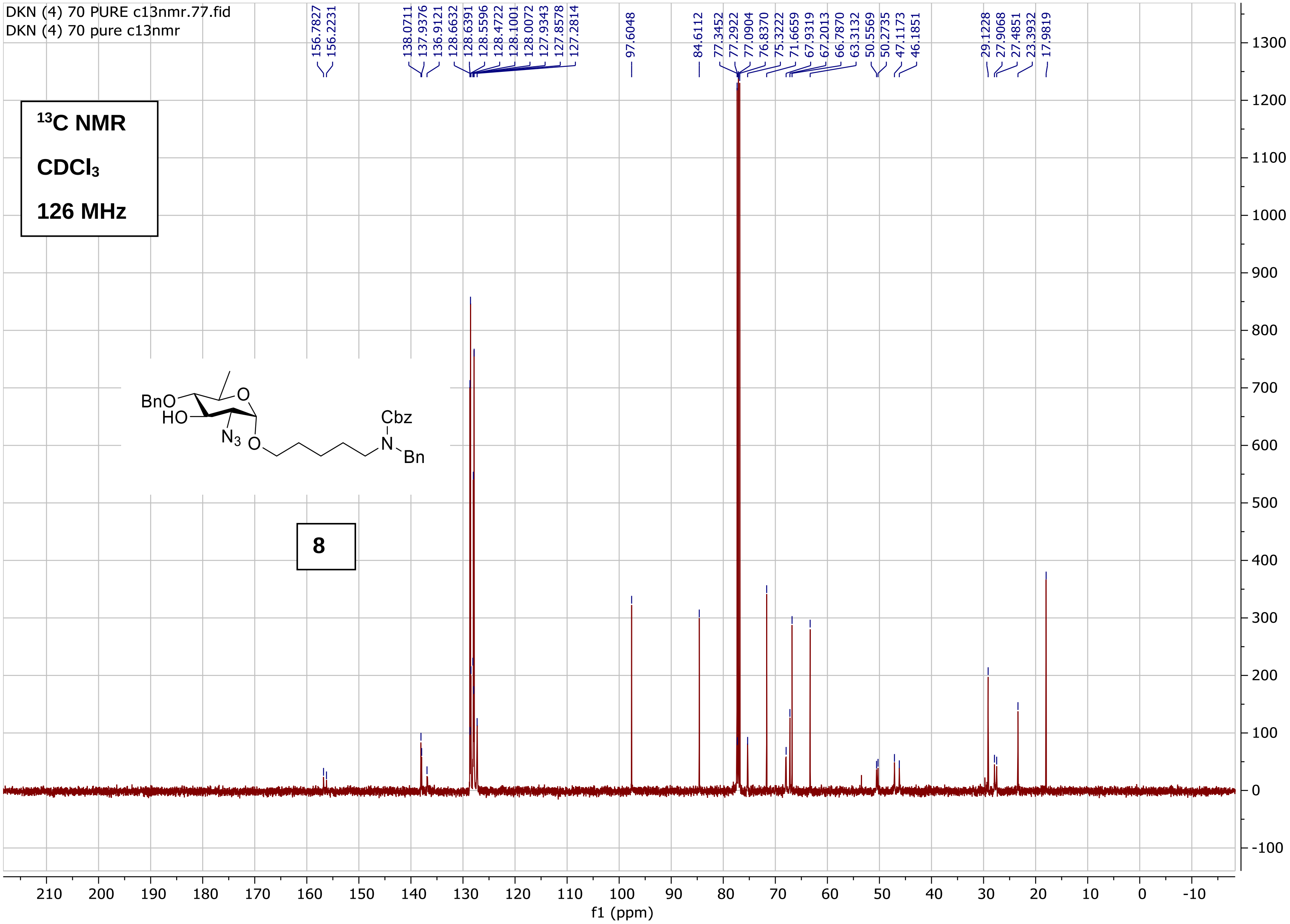
DKN (4) 70 PURE c13nmr.77.fid
DKN (4) 70 pure c13nmr
13C NMR
CDCl3
126 MHz
156.7827
156.2231
138.0711
137.9376
136.9121
128.6632
128.6391
128.5596
128.4722
128.1001
128.0072
127.9343
127.8578
127.2814
97.6048
84.6112
77.3452
77.2922
77.0904
76.8370
75.3222
71.6659
67.9319
67.2013
66.7870
63.3132
50.5569
50.2735
47.1173
46.1851
29.1228
27.9068
27.4851
23.3932
17.9819
BnO
HO
O
N3
O
Cbz
N
Bn
f1 (ppm)

8

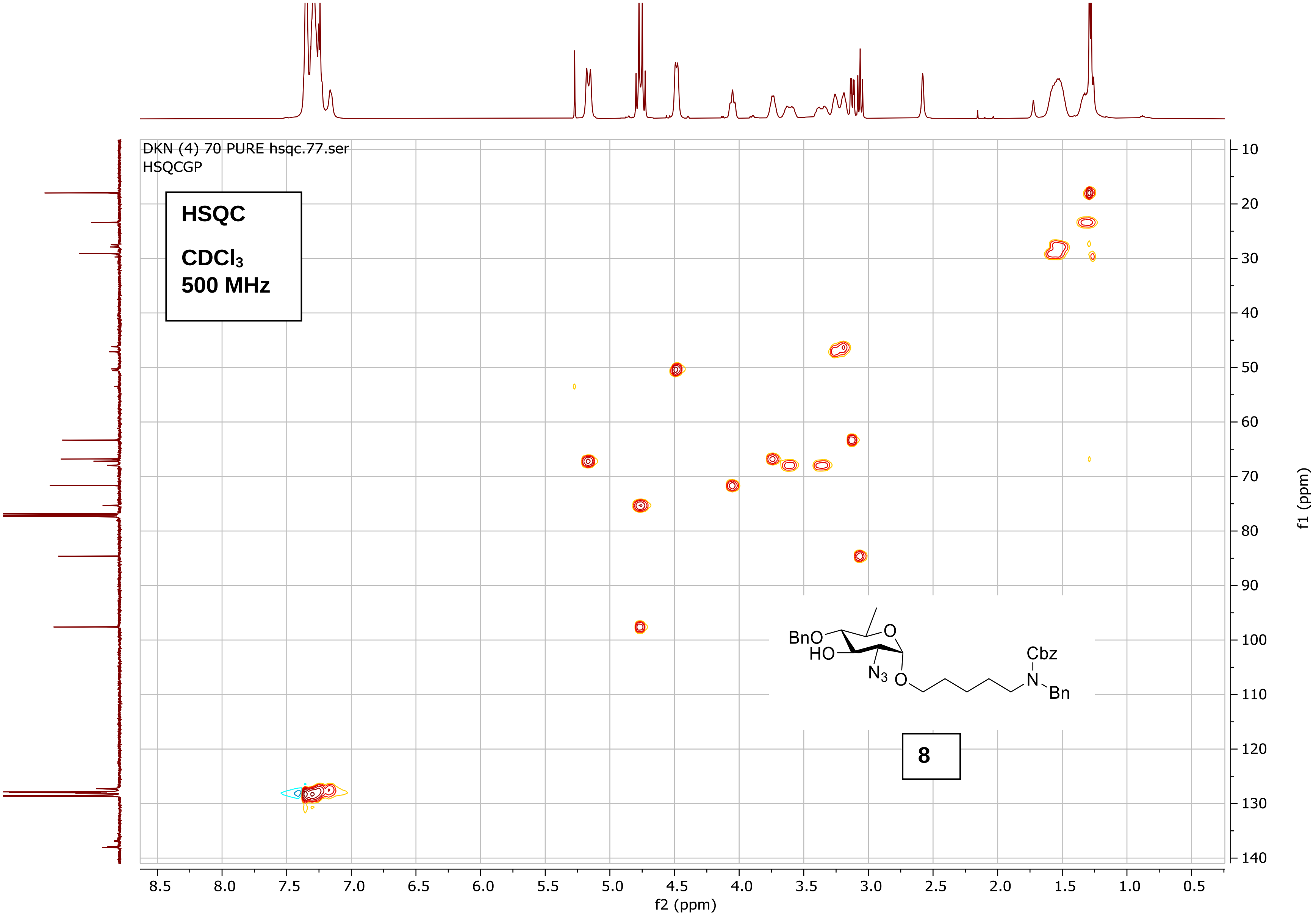
DKN (4) 70 PURE hsqc.77.ser
HSQCGP
HSQC
$CDCl_3$
500 MHz
BnO
HO
O
N_3
O
Cbz
N
Bn
8
f2 (ppm)
f1 (ppm)

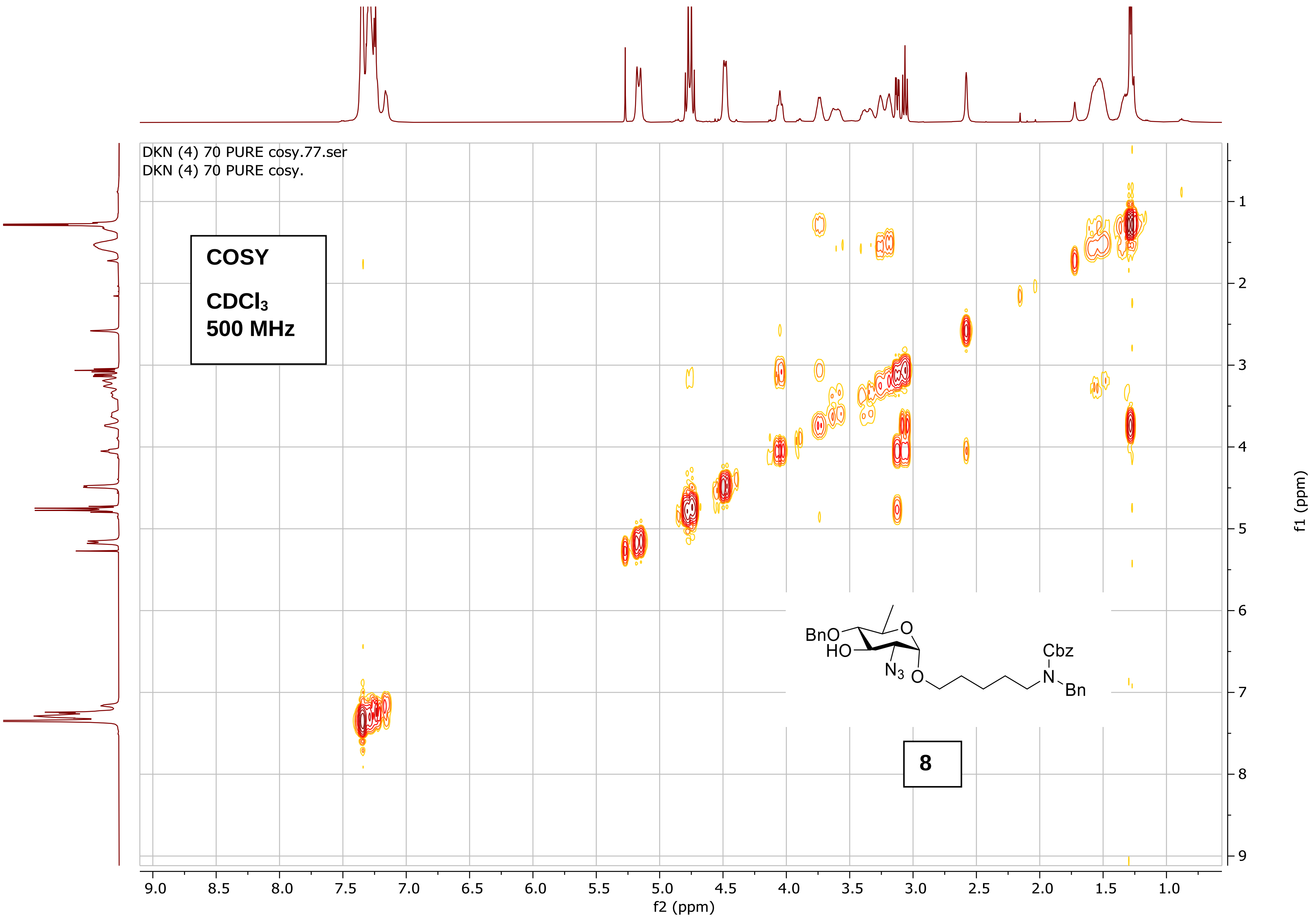
DKN (4) 70 PURE cosy.77.ser
DKN (4) 70 PURE cosy.
COSY
CDCl3
500 MHz
BnO
HO
O
N3
O
Cbz
N
Bn
8
f2 (ppm)
f1 (ppm)

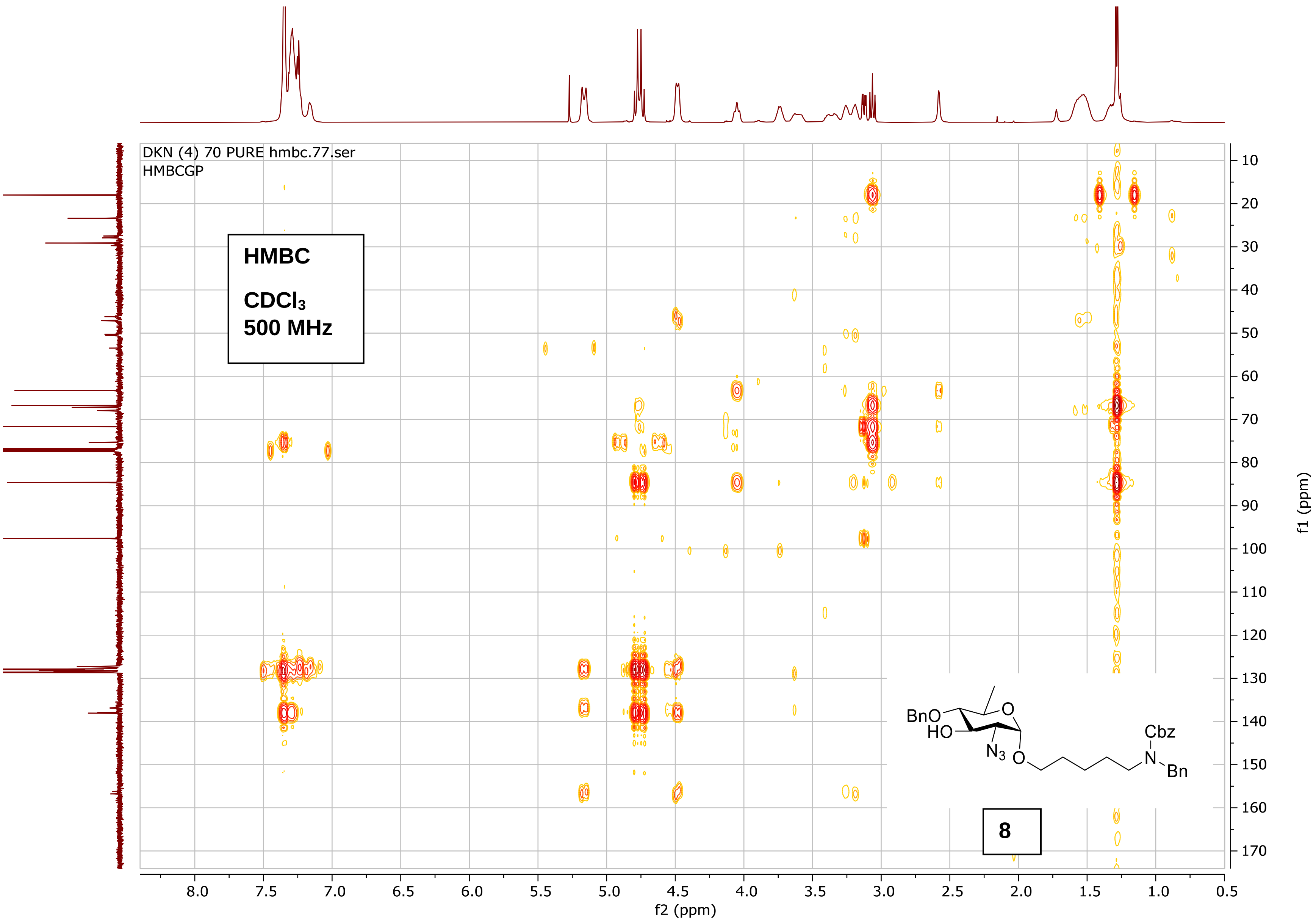
DKN (4) 70 PURE hmbc.77.ser
HMBCGP
HMBC
CDCl3
500 MHz
BnO
HO
N3
O
O
Cbz
N
Bn
8
8.0
7.5
7.0
6.5
6.0
5.5
5.0
4.5
4.0
3.5
3.0
2.5
2.0
1.5
1.0
0.5
f2 (ppm)
10
20
30
40
50
60
70
80
90
100
110
120
130
140
150
160
170
f1 (ppm)

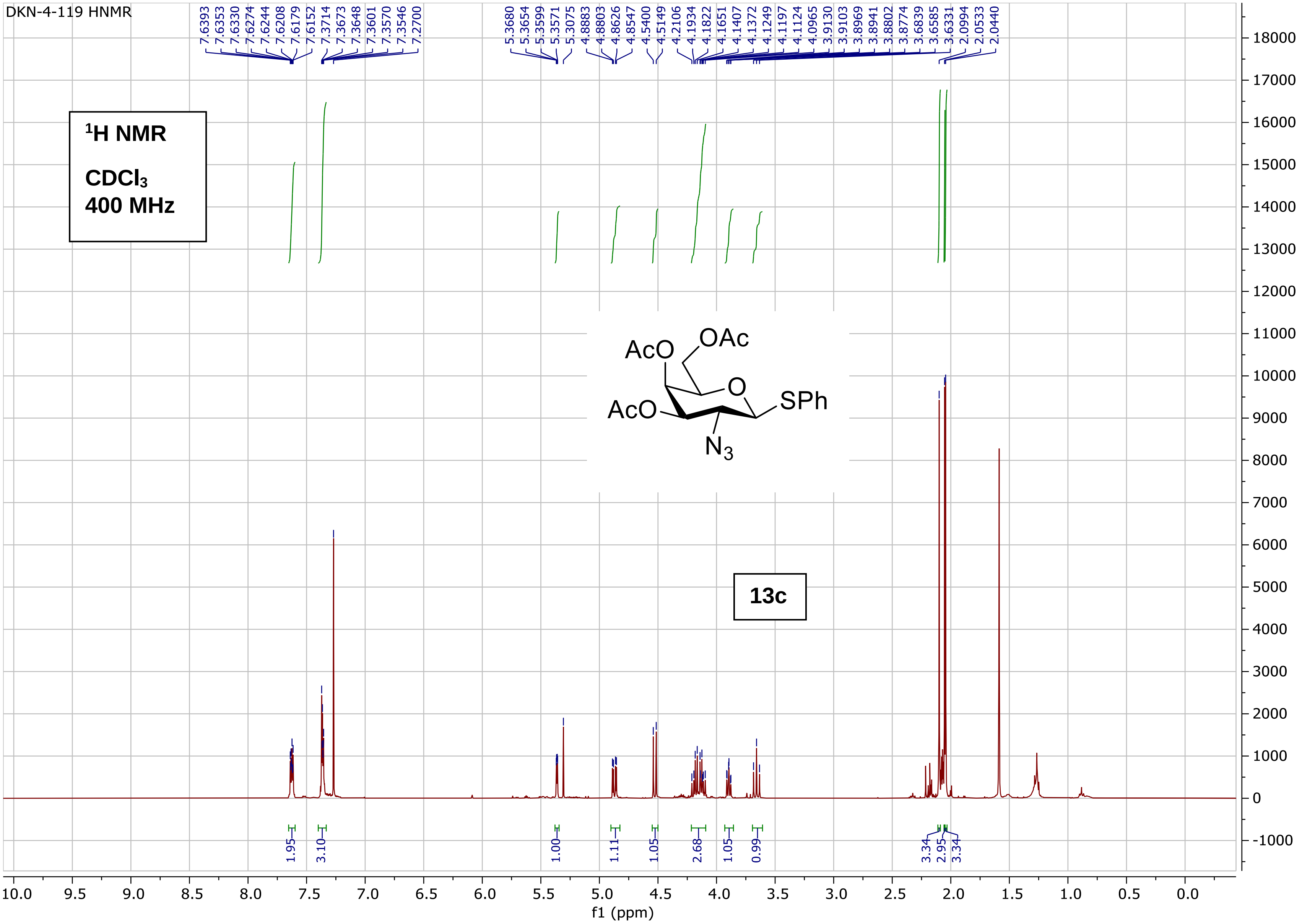
DKN-4-119 HNMR
7.6393 7.6353 7.6330 7.6274 7.6244 7.6208 7.6179 7.6152 7.3714 7.3673 7.3648 7.3601 7.3570 7.3546 7.2700
5.3680 5.3654 5.3599 5.3571 5.3075 4.8883 4.8803 4.8626 4.8547 4.5400 4.5149 4.2106 4.1934 4.1822 4.1651 4.1407 4.1372 4.1249 4.1197 4.1124 4.0965 3.9130 3.9103 3.8969 3.8941 3.8802 3.8774 3.6839 3.6585 3.6331 2.0994 2.0533 2.0440
1H NMR
CDCl3
400 MHz
AcO
OAc
O
SPh
N3
13c
1.95
3.10
1.00
1.11
1.05
2.68
1.05
0.99
3.34
2.95
3.34
f1 (ppm)

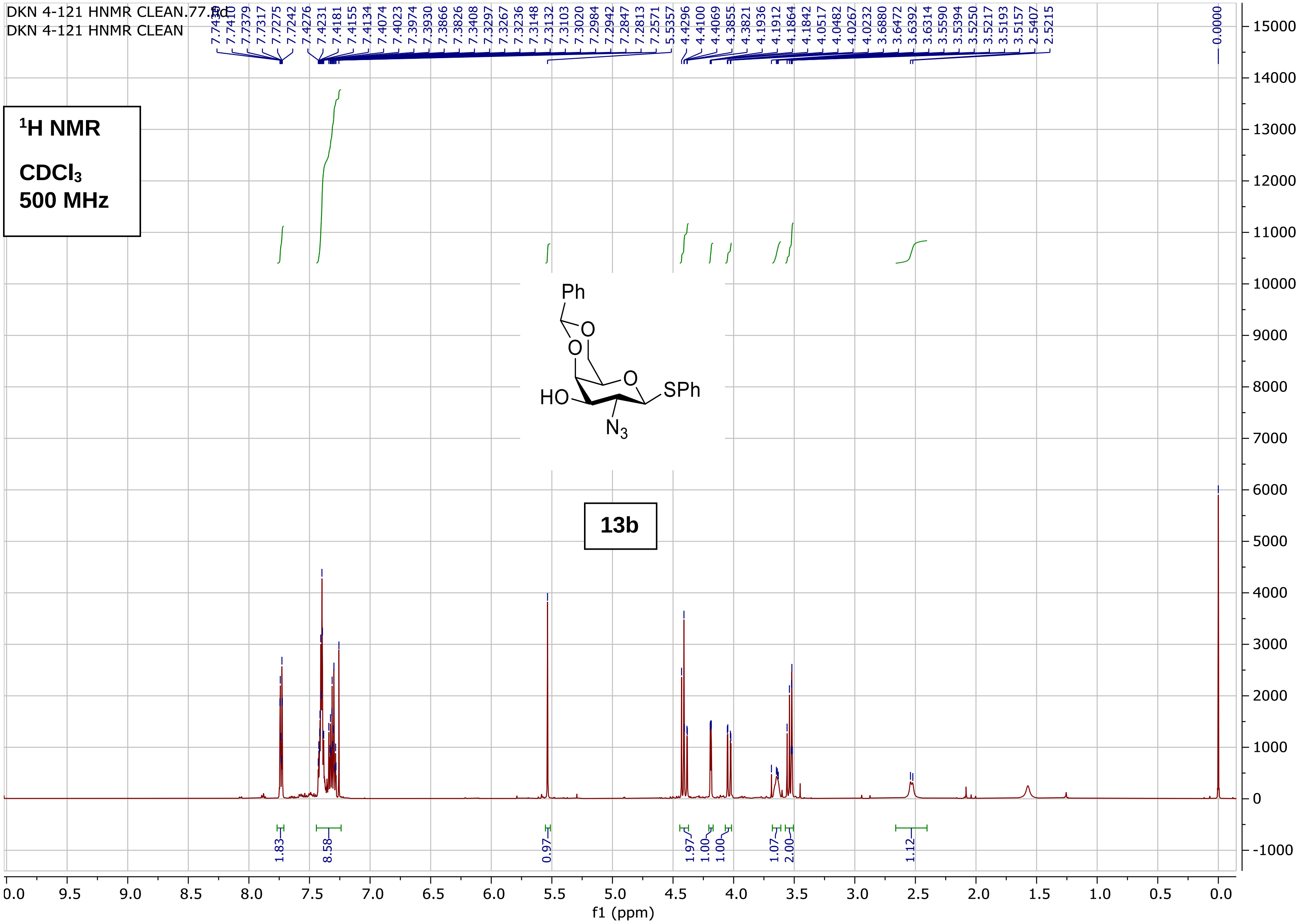
DKN 4-121 HNMR CLEAN.77.fid
DKN 4-121 HNMR CLEAN
7.7430
7.7410
7.7379
7.7317
7.7275
7.7242
7.4276
7.4231
7.4181
7.4155
7.4134
7.4074
7.4023
7.3974
7.3930
7.3866
7.3826
7.3408
7.3297
7.3267
7.3236
7.3148
7.3132
7.3103
7.3020
7.2984
7.2942
7.2847
7.2813
7.2571
5.5357
4.4296
4.4100
4.4069
4.3855
4.3821
4.1936
4.1912
4.1864
4.1842
4.0517
4.0482
4.0267
4.0232
3.6880
3.6472
3.6392
3.6314
3.5590
3.5394
3.5250
3.5217
3.5193
3.5157
2.5407
2.5215
0.0000
¹H NMR
CDCl3
500 MHz
Ph
O
O
O
HO
SPh
N3
13b
1.83
8.58
0.97
1.97
1.00
1.00
1.07
2.00
1.12
f1 (ppm)

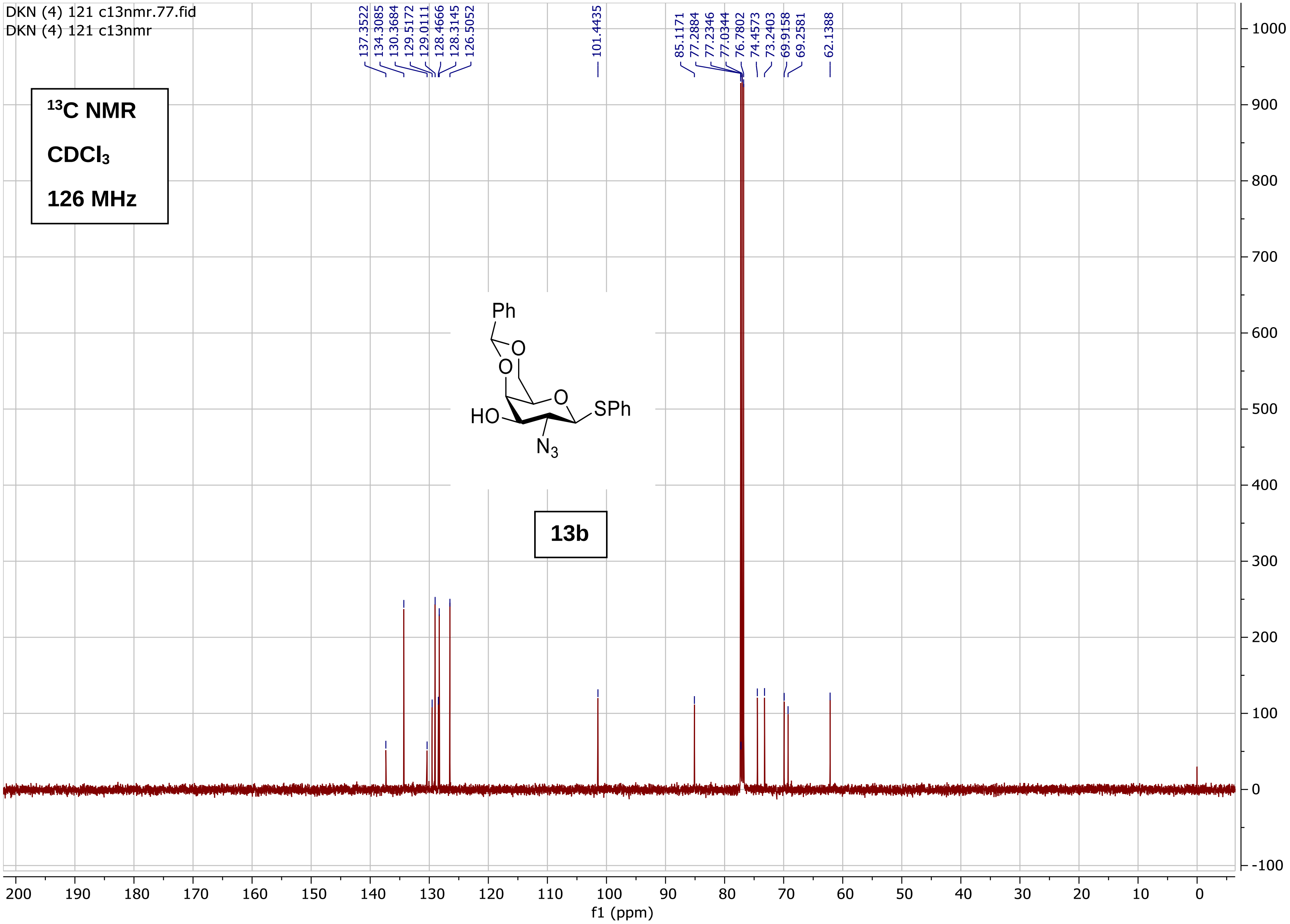
DKN (4) 121 c13nmr.77.fid
DKN (4) 121 c13nmr
13C NMR
CDCl3
126 MHz
137.3522
134.3085
130.3684
129.5172
129.0111
128.4666
128.3145
126.5052
101.4435
85.1171
77.2884
77.2346
77.0344
76.7802
74.4573
73.2403
69.9158
69.2581
62.1388
Ph
O
O
O
HO
SPh
N3
13b
f1 (ppm)

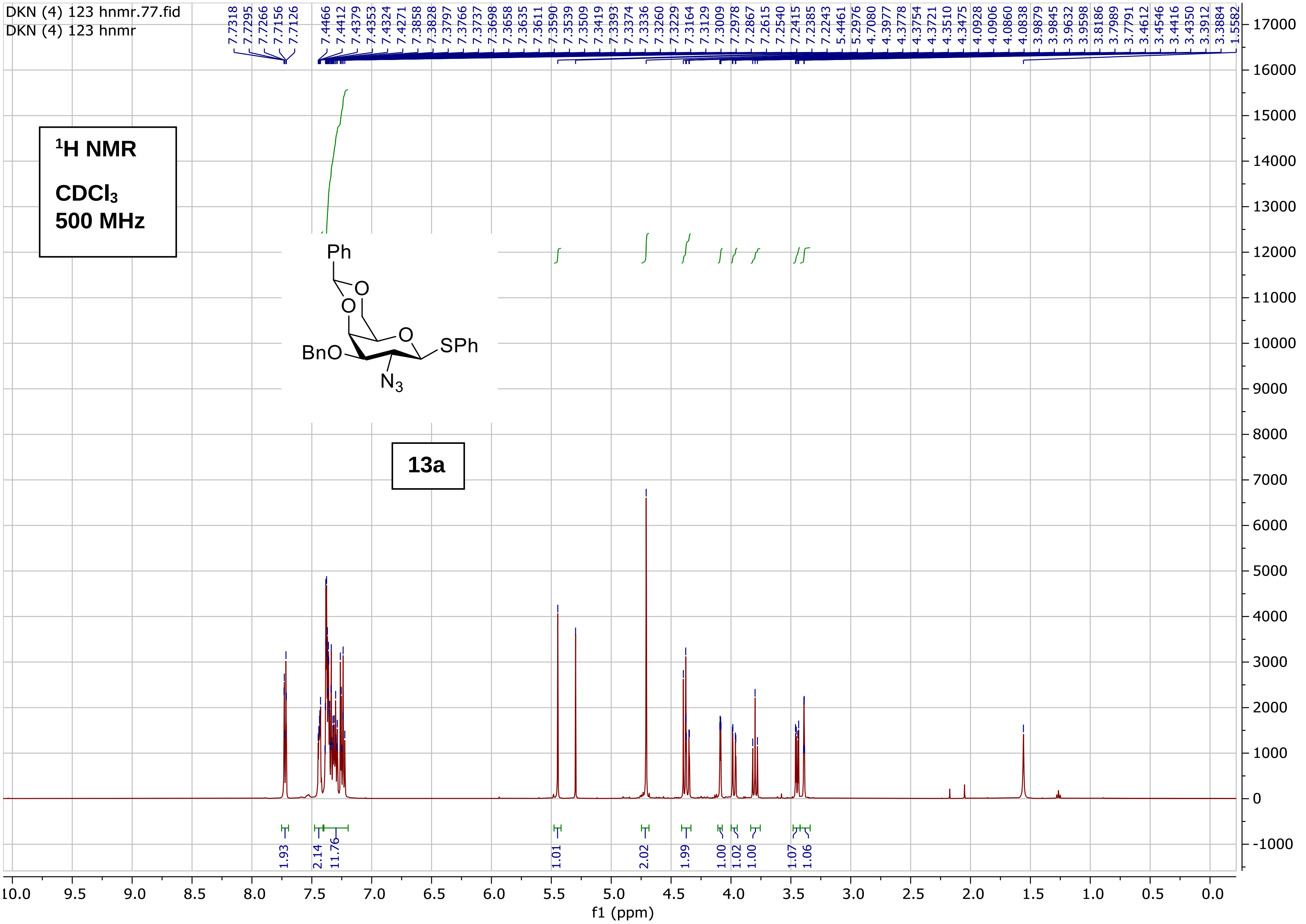
DKN (4) 123 hnmr.77.fid
DKN (4) 123 hnmr
1H NMR
CDCl3
500 MHz
Ph
O
O
O
BnO
SPh
N3
13a
7.7318
7.7295
7.7266
7.7156
7.7126
7.4466
7.4412
7.4379
7.4353
7.4324
7.4271
7.3858
7.3828
7.3797
7.3766
7.3737
7.3698
7.3658
7.3635
7.3611
7.3590
7.3539
7.3509
7.3419
7.3393
7.3374
7.3336
7.3260
7.3229
7.3164
7.3129
7.3009
7.2978
7.2867
7.2615
7.2540
7.2415
7.2385
7.2243
5.4461
5.2976
4.7080
4.3977
4.3778
4.3754
4.3721
4.3510
4.3475
4.0928
4.0906
4.0860
4.0838
3.9879
3.9845
3.9632
3.9598
3.8186
3.7989
3.7791
3.4612
3.4546
3.4416
3.4350
3.3912
3.3884
1.5582
1.93
2.14
11.76
1.01
2.02
1.99
1.00
1.02
1.00
1.07
1.06
f1 (ppm)

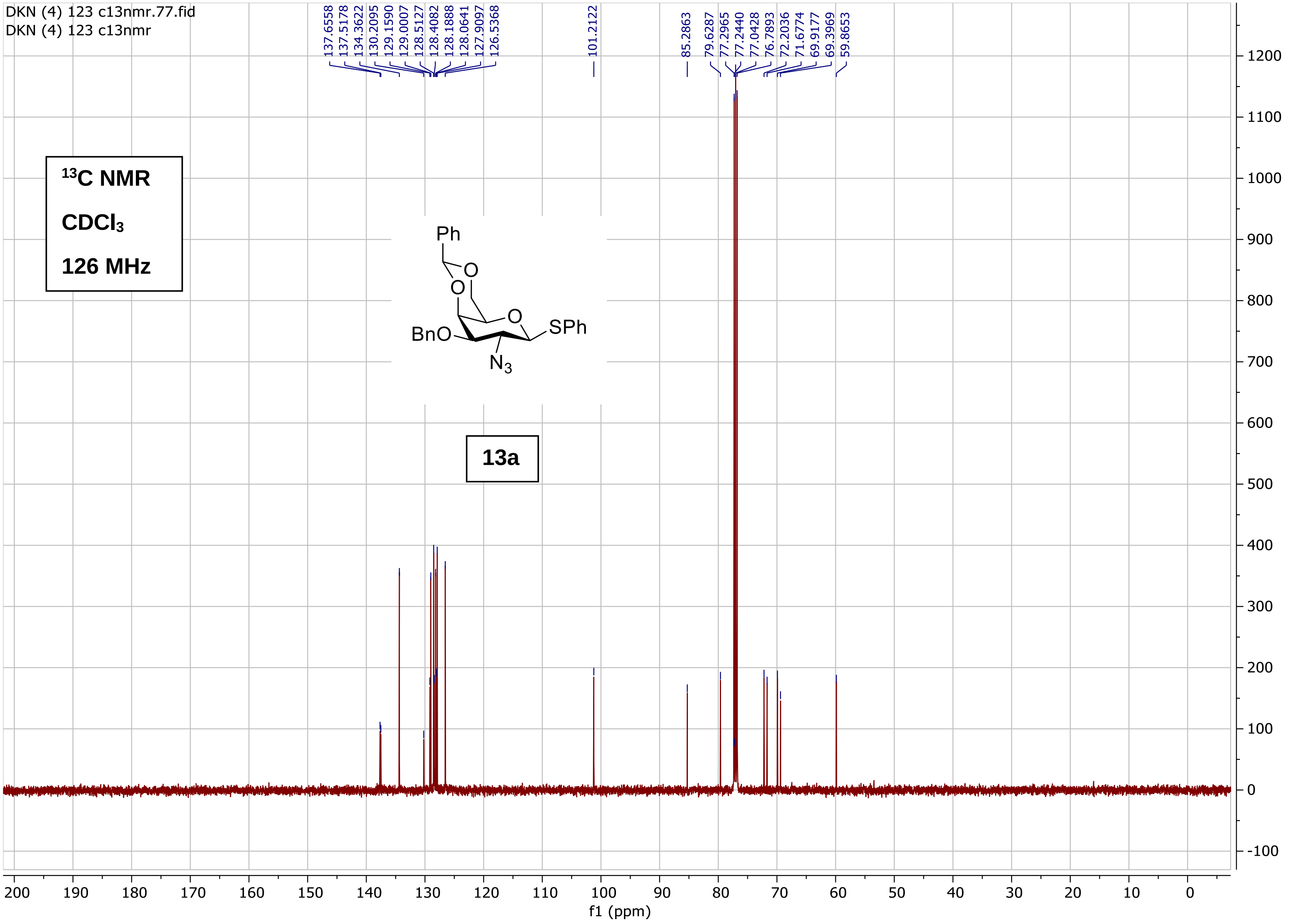

DKN (4) 123 c13nmr.77.fid
DKN (4) 123 c13nmr
137.6558
137.5178
134.3622
130.2095
129.1590
129.0007
128.5127
128.4082
128.1888
128.0641
127.9097
126.5368
101.2122
85.2863
79.6287
77.2965
77.2440
77.0428
76.7893
72.2036
71.6774
69.9177
69.3969
59.8653
13C NMR
CDCl3
126 MHz
Ph
O
O
O
BnO
SPh
N3
13a
f1 (ppm)

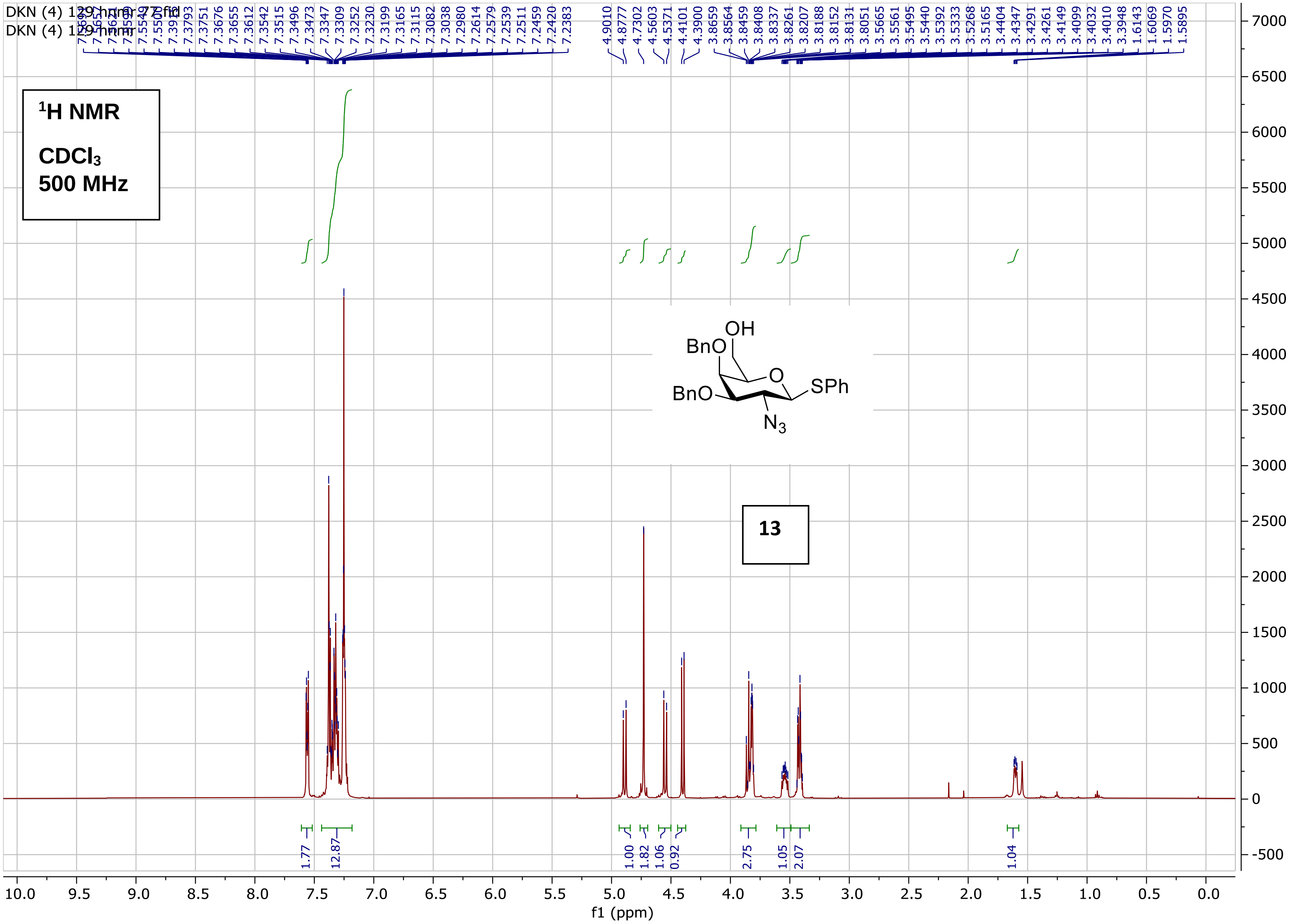
1H NMR
CDCl3
500 MHz
OH
BnO
O
SPh
BnO
N3
13
f1 (ppm)

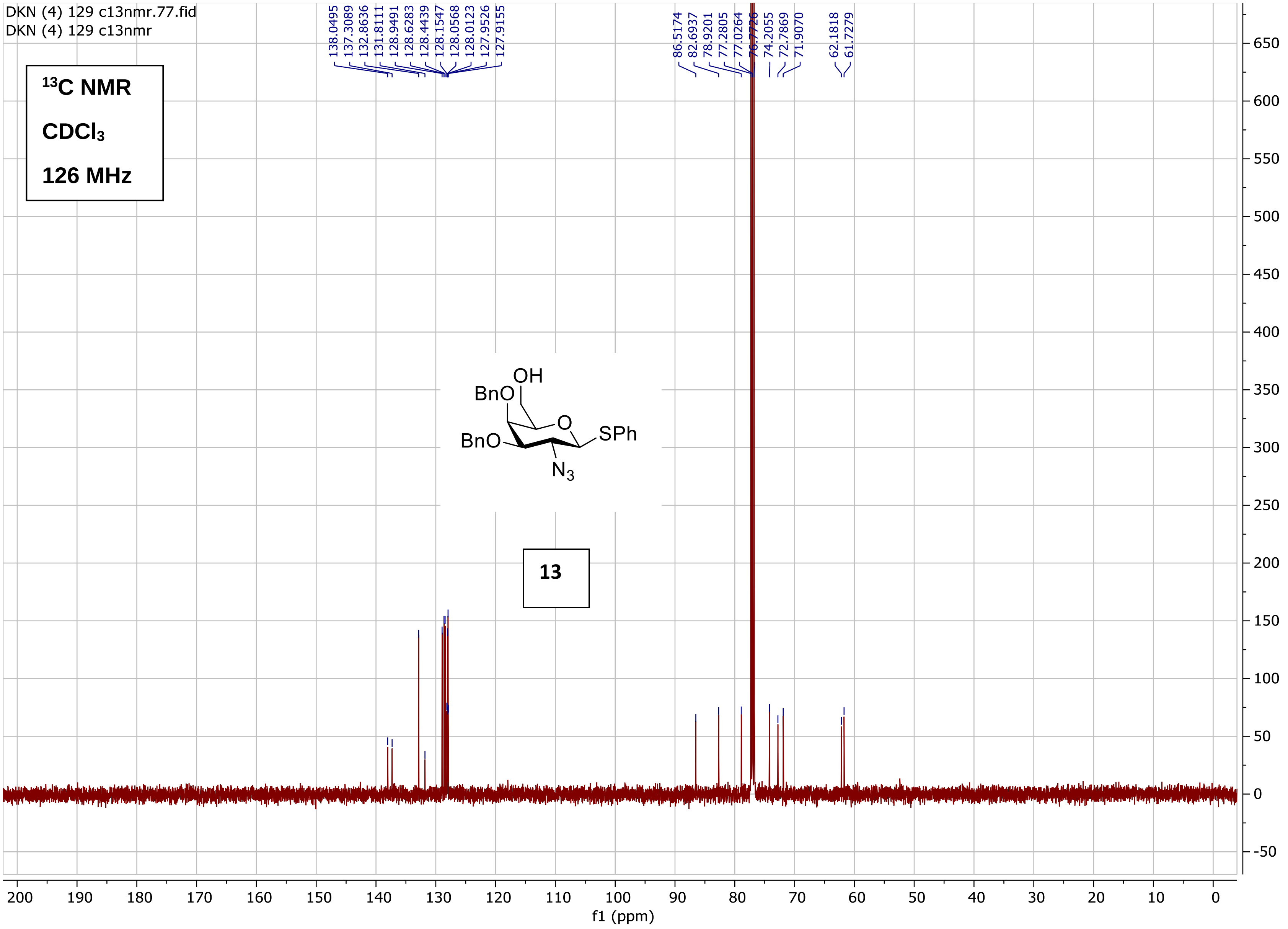
DKN (4) 129 c13nmr.77.fid
DKN (4) 129 c13nmr
13C NMR
CDCl3
126 MHz
138.0495
137.3089
132.8636
131.8111
128.9491
128.6283
128.4439
128.1547
128.0568
128.0123
127.9526
127.9155
86.5174
82.6937
78.9201
77.2805
77.0264
76.7726
74.2055
72.7869
71.9070
62.1818
61.7279
OH
BnO
O
SPh
BnO
N3
13
f1 (ppm)

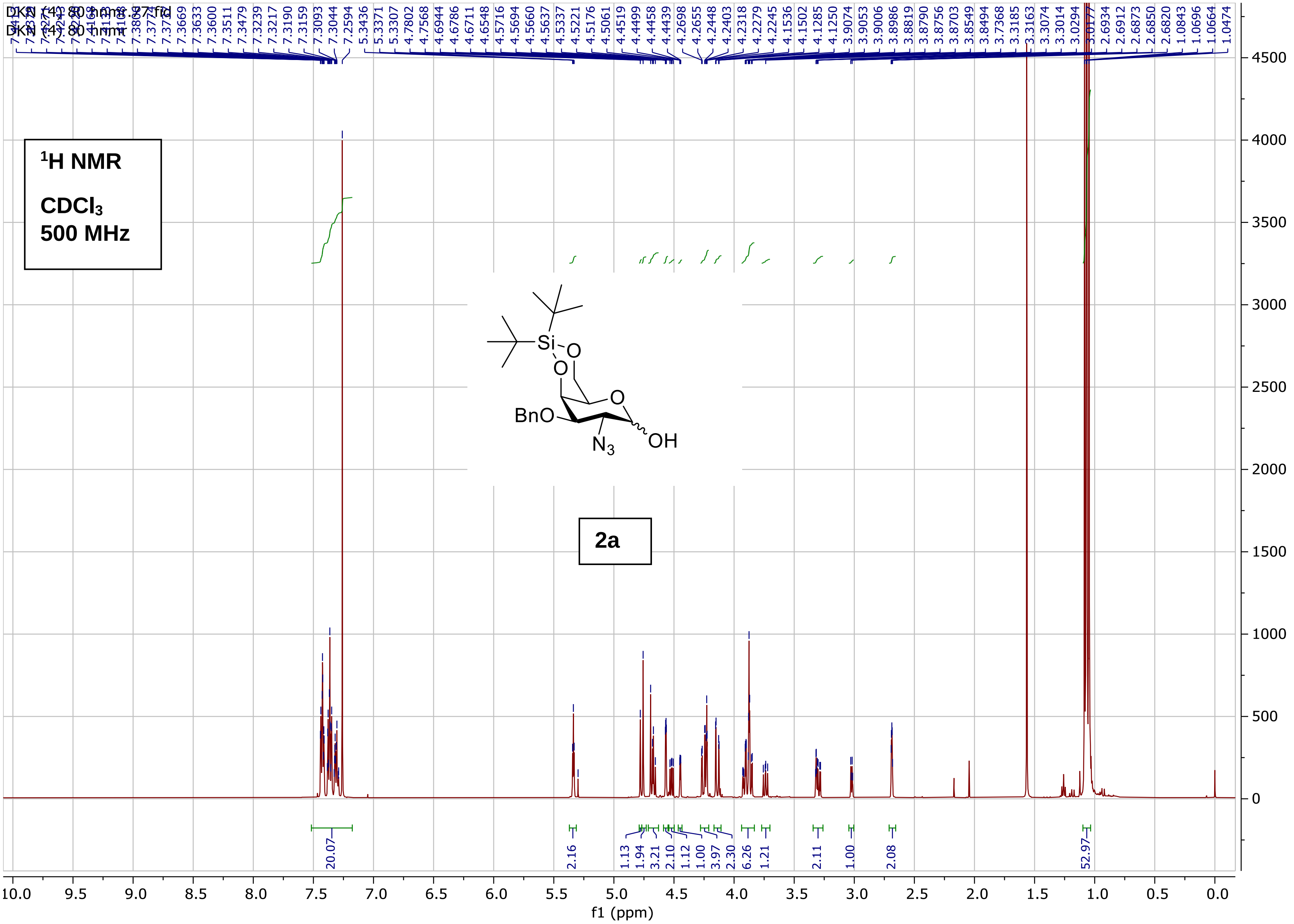
1H NMR
CDCl3
500 MHz
BnO
N3
OH
Si
O
2a
f1 (ppm)

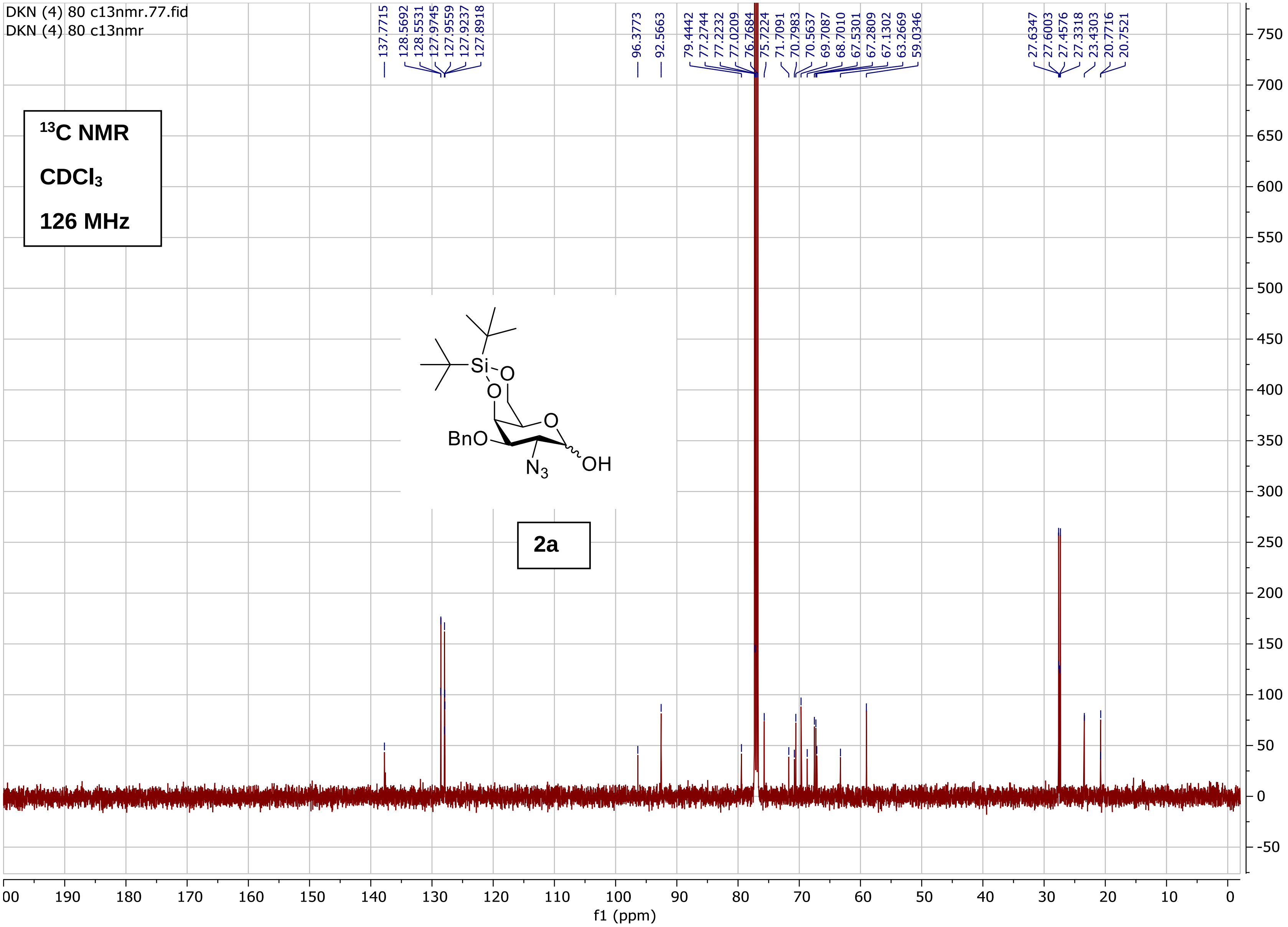
DKN (4) 80 c13nmr.77.fid
DKN (4) 80 c13nmr
137.7715
128.5692
128.5531
127.9745
127.9559
127.9237
127.8918
96.3773
92.5663
79.4442
77.2744
77.2232
77.0209
76.7684
75.7224
71.7091
70.7983
70.5637
69.7087
68.7010
67.5301
67.2809
67.1302
63.2669
59.0346
27.6347
27.6003
27.4576
27.3318
23.4303
20.7716
20.7521
^{13}C NMR
$CDCl_3$
126 MHz
Si
O
O
O
BnO
N_3
OH
2a
f1 (ppm)

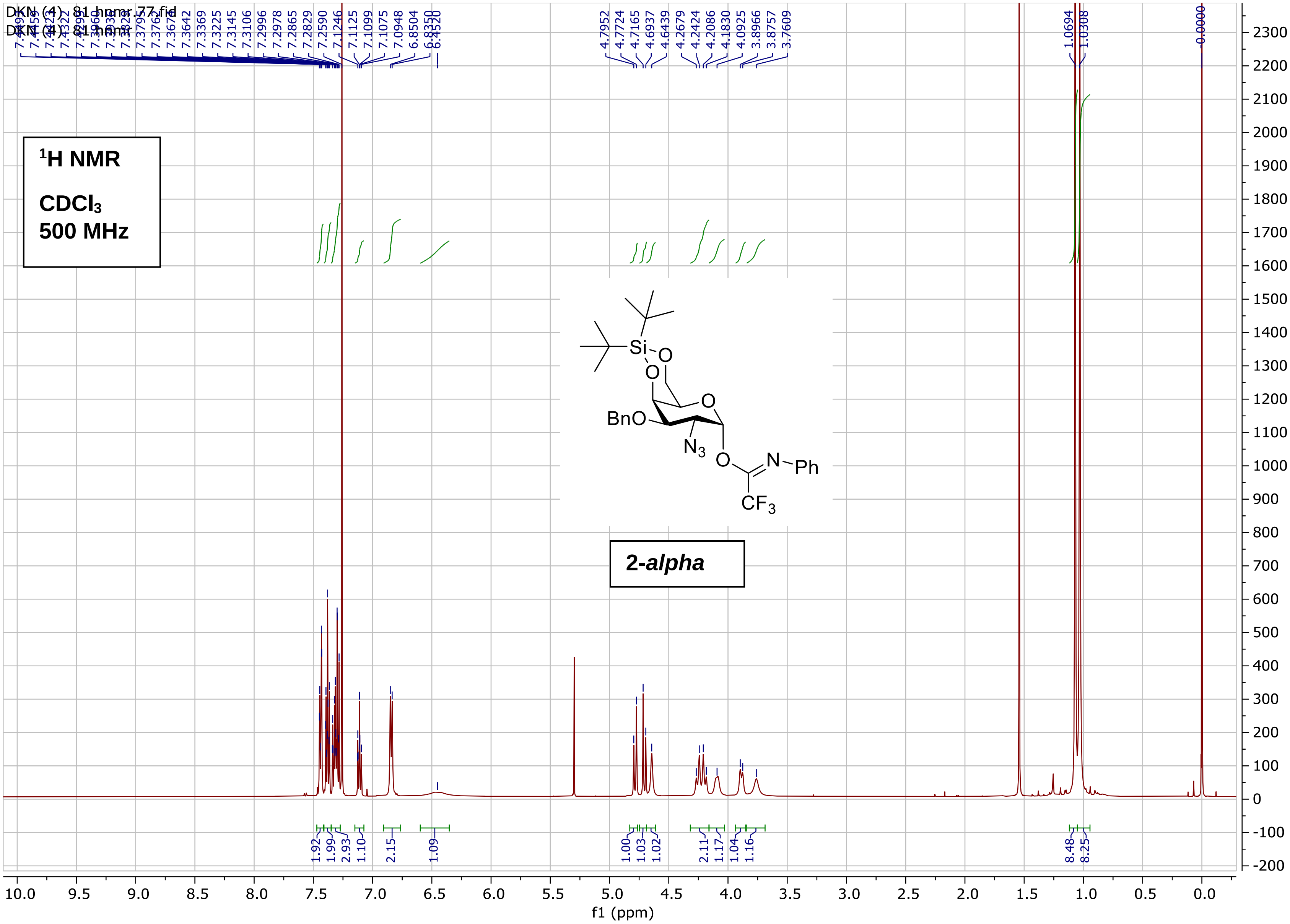

1H NMR
CDCl3
500 MHz
2-alpha
BnO
N3
O
Si
N
Ph
CF3
f1 (ppm)

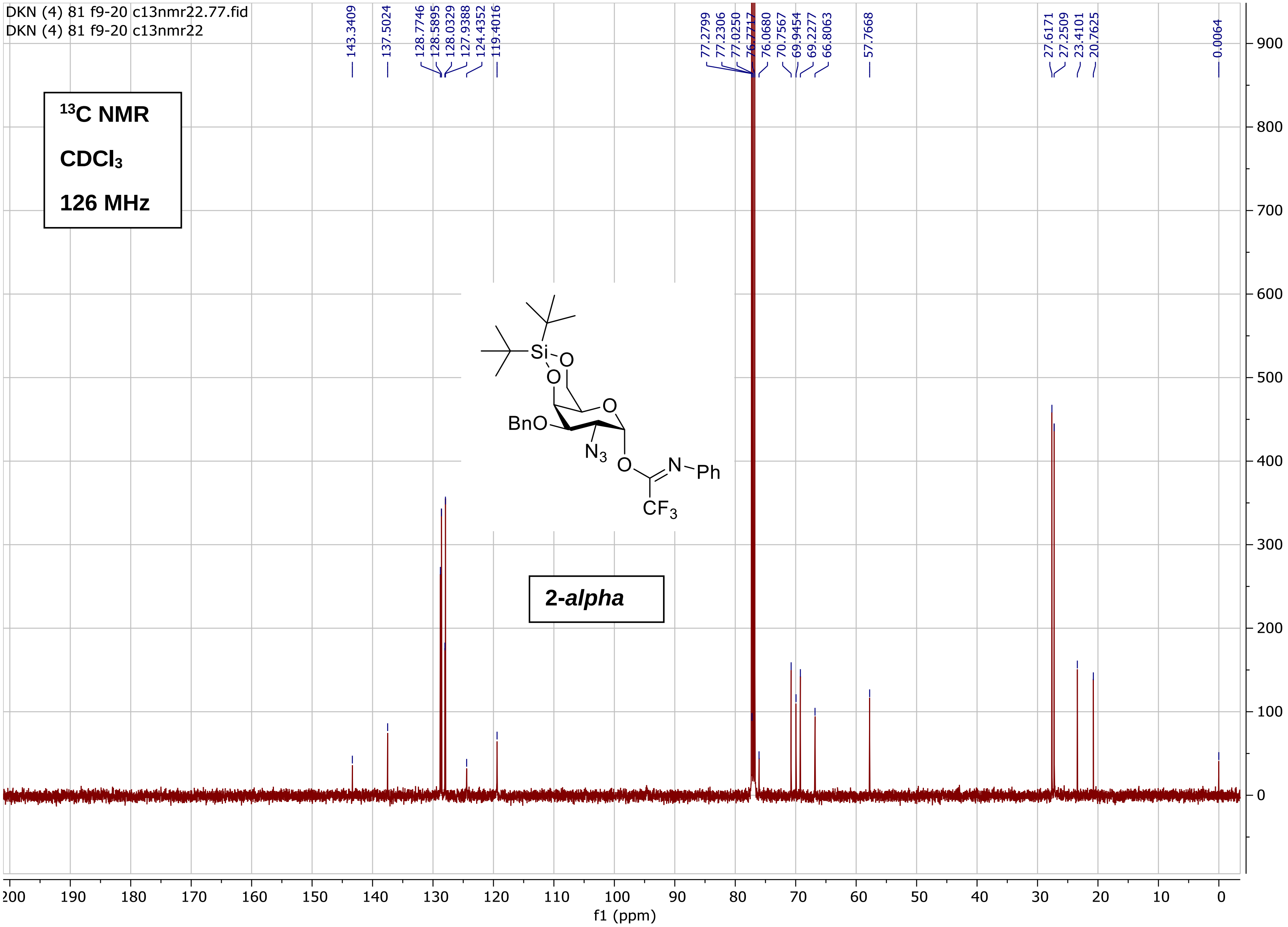

DKN (4) 81 f9-20 c13nmr22.77.fid
DKN (4) 81 f9-20 c13nmr22
13C NMR
CDCl3
126 MHz
143.3409
137.5024
128.7746
128.5895
128.0329
127.9388
124.4352
119.4016
77.2799
77.2306
77.0250
76.7717
76.0680
70.7567
69.9454
69.2277
66.8063
57.7668
27.6171
27.2509
23.4101
20.7625
0.0064
Si
O
O
O
BnO
N3
O
N
Ph
CF3
2-alpha
f1 (ppm)

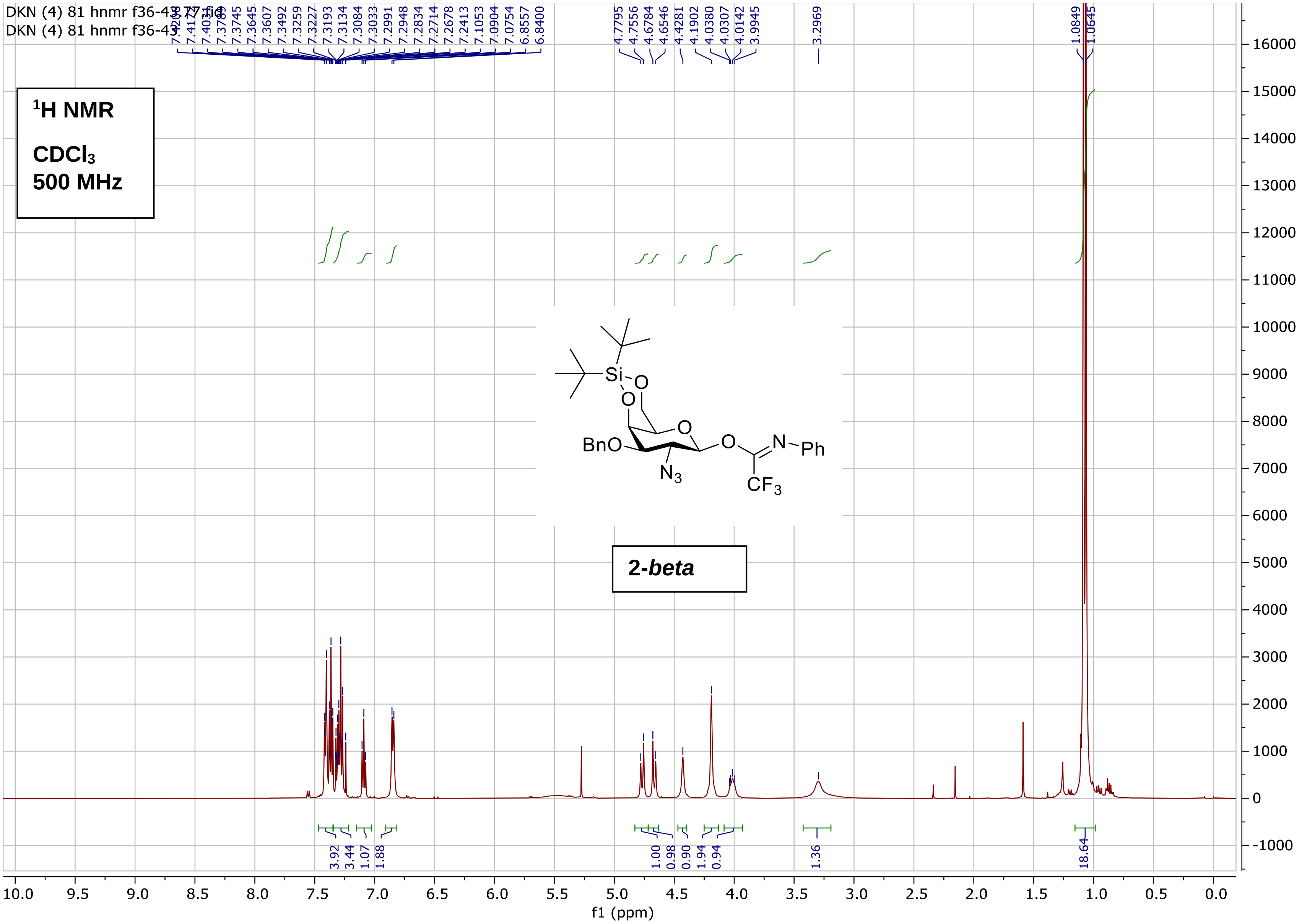
DKN (4) 81 hnmr f36-43
1H NMR
CDCl3
500 MHz
7.4177
7.4031
7.3745
7.3645
7.3607
7.3492
7.3259
7.3227
7.3193
7.3134
7.3084
7.3033
7.2991
7.2948
7.2834
7.2714
7.2678
7.2413
7.1053
7.0904
7.0754
6.8557
6.8400
4.7795
4.7556
4.6784
4.6546
4.4281
4.1902
4.0380
4.0307
4.0142
3.9945
3.2969
1.0849
1.0645
BnO
O
Si
N3
N
Ph
CF3
2-beta
3.92
3.44
1.07
1.88
1.00
0.98
0.90
1.94
0.94
1.36
18.64
f1 (ppm)

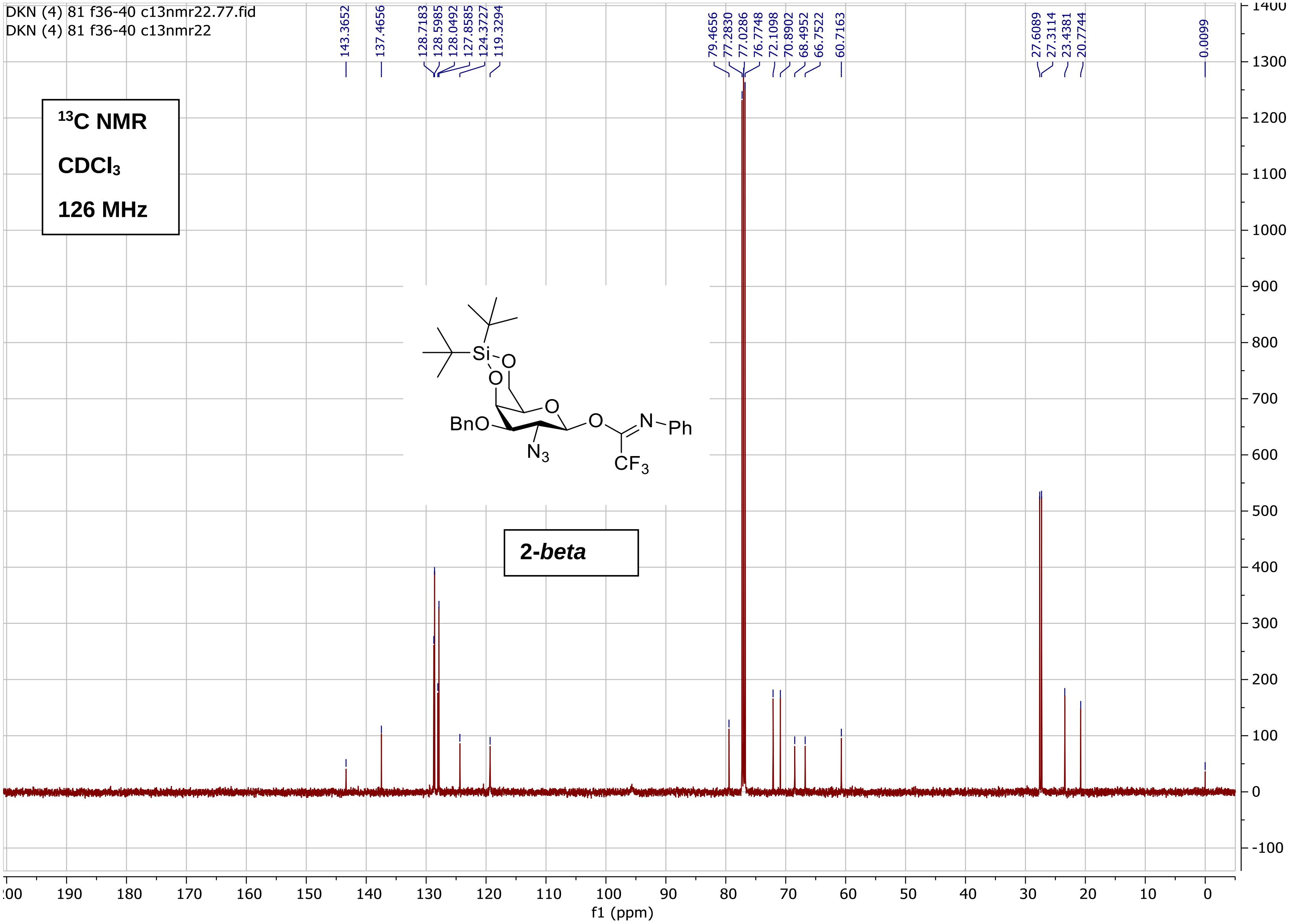
DKN (4) 81 f36-40 c13nmr22.77.fid
DKN (4) 81 f36-40 c13nmr22
13C NMR
CDCl3
126 MHz
2-beta
143.3652
137.4656
128.7183
128.5985
128.0492
127.8585
124.3727
119.3294
79.4656
77.2830
77.0286
76.7748
72.1098
70.8902
68.4952
66.7522
60.7163
27.6089
27.3114
23.4381
20.7744
0.0099
BnO
N3
CF3
N
Ph
Si
O
f1 (ppm)

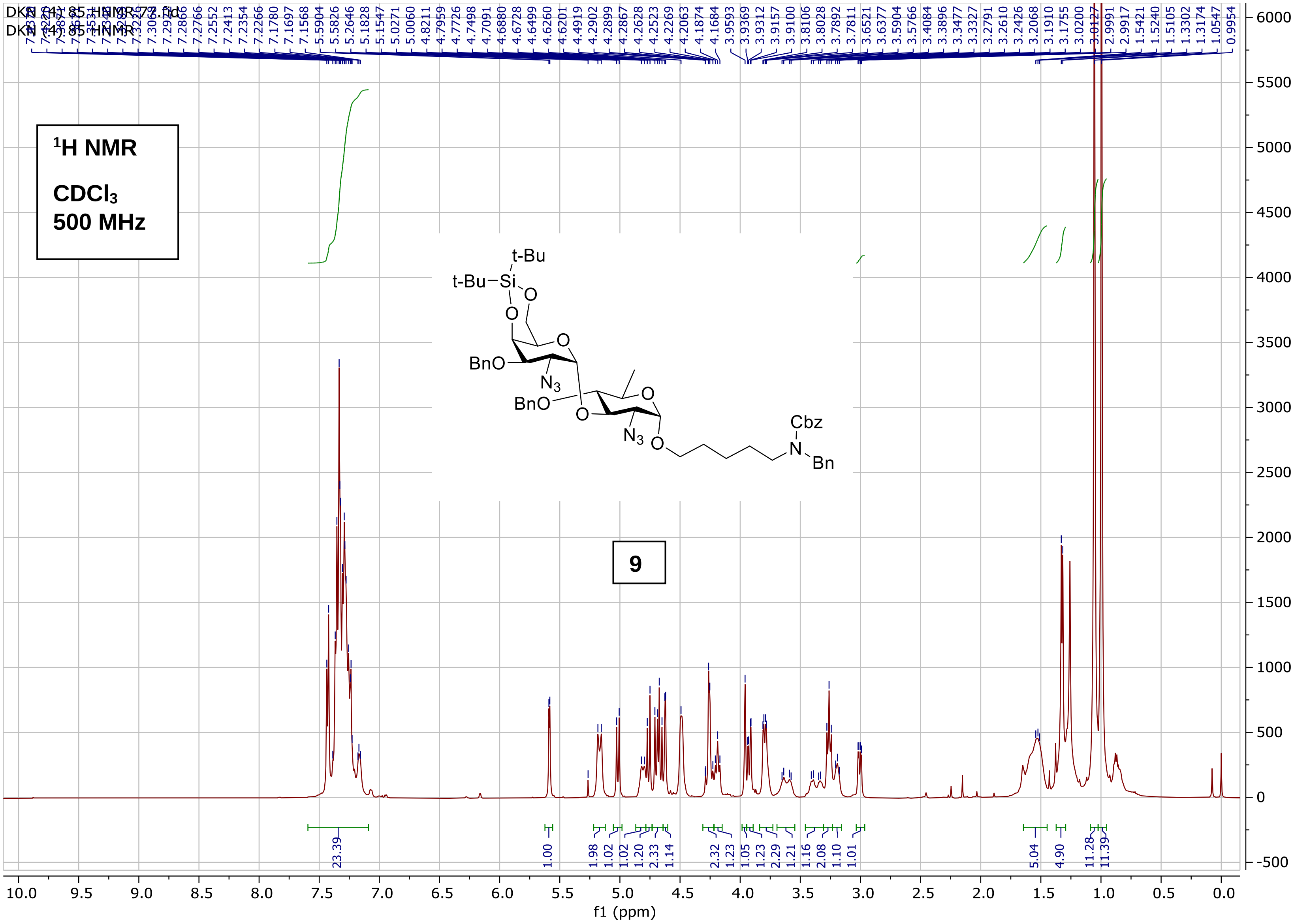
1H NMR
CDCl3
500 MHz
9
t-Bu
t-Bu−Si
BnO
N3
Cbz
N
Bn
f1 (ppm)

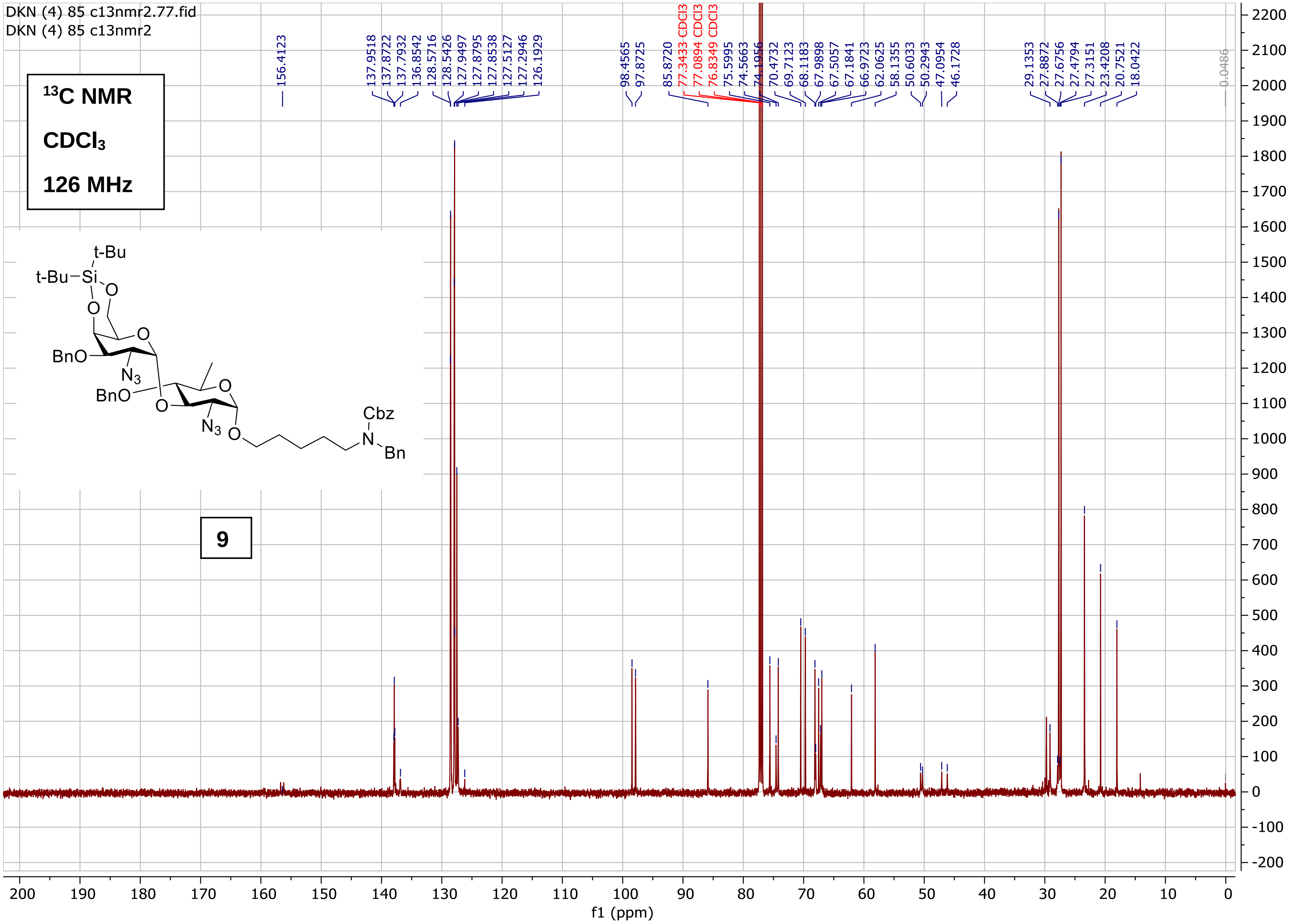
DKN (4) 85 c13nmr2.77.fid
DKN (4) 85 c13nmr2
^{13}C NMR
$CDCl_3$
126 MHz
9
t-Bu
t-Bu
Si
BnO
BnO
N_3
N_3
Cbz
N
Bn
156.4123
137.9518
137.8722
137.7932
136.8542
128.5716
128.5426
127.9497
127.8795
127.8538
127.5127
127.2946
126.1929
98.4565
97.8725
85.8720
77.3433 CDCl3
77.0894 CDCl3
76.8349 CDCl3
75.5995
74.5663
74.1956
70.4732
69.7123
68.1183
67.9898
67.5057
67.1841
66.9723
62.0625
58.1355
50.6033
50.2943
47.0954
46.1728
29.1353
27.8872
27.6756
27.4794
27.3151
23.4208
20.7521
18.0422
0.0486
f1 (ppm)

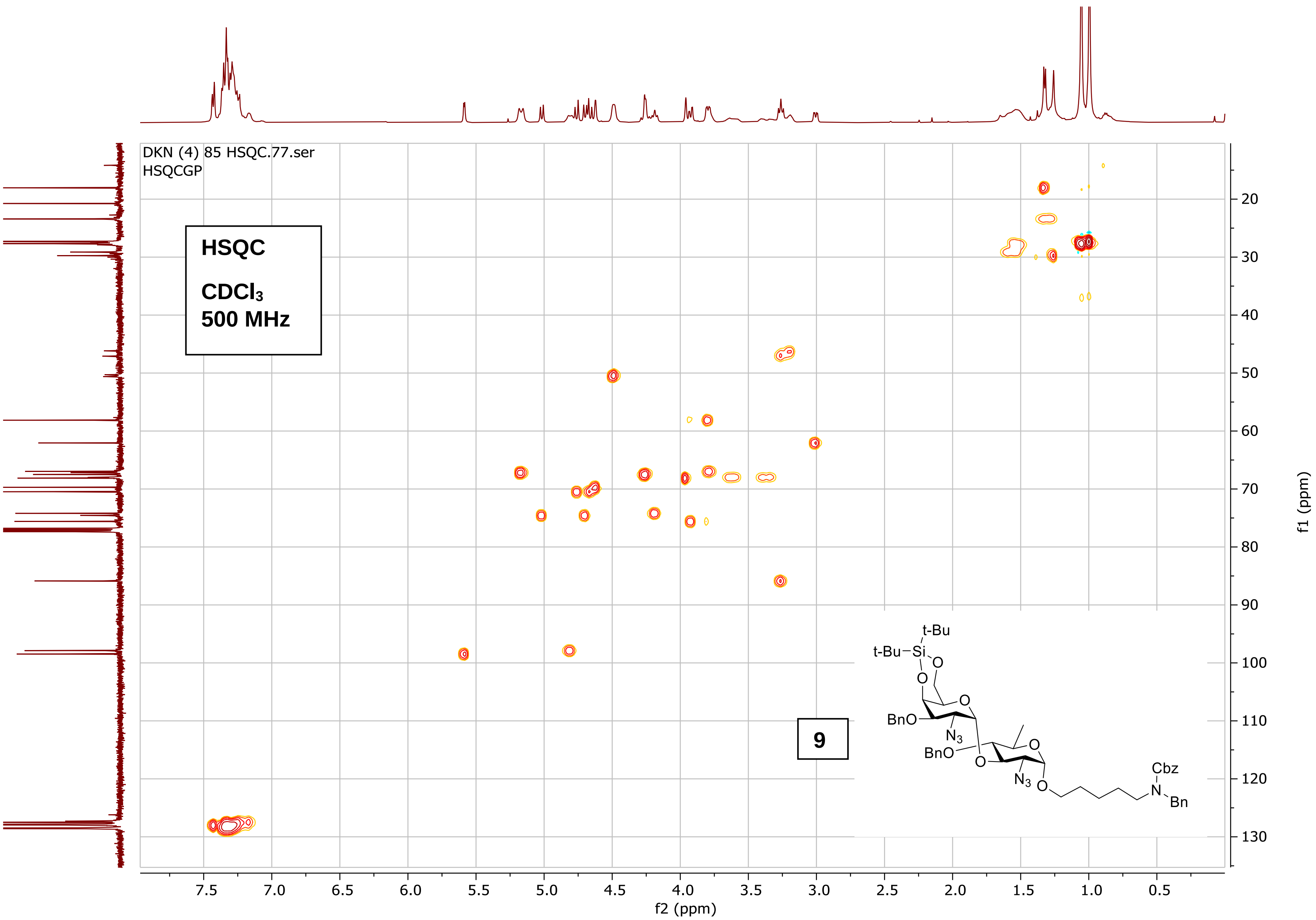

DKN (4) 85 HSQC.77.ser
HSQCGP
HSQC
CDCl3
500 MHz
9
t-Bu
t-Bu
Si
O
O
BnO
N3
BnO
O
N3
O
Cbz
N
Bn
f2 (ppm)
f1 (ppm)

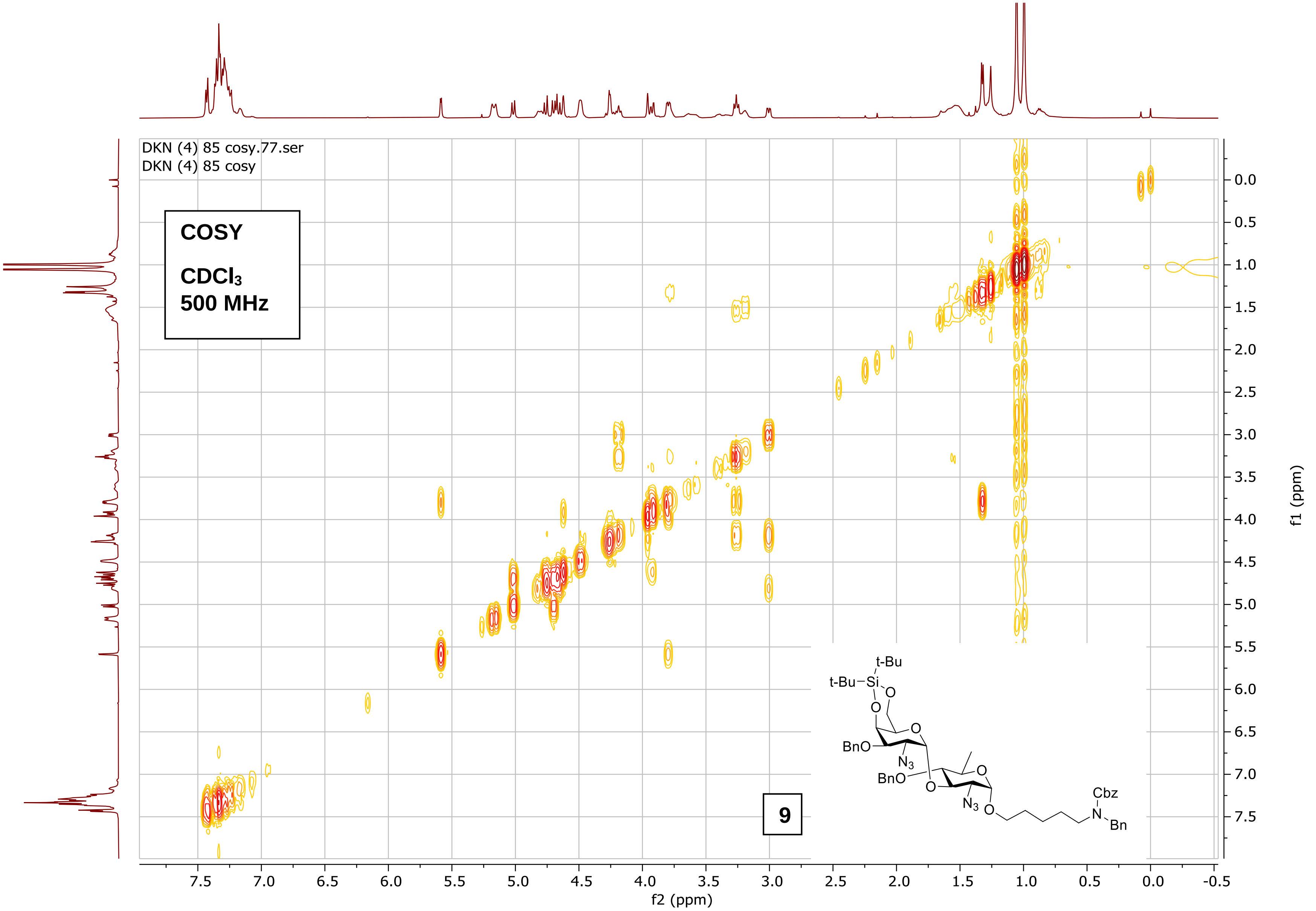
DKN (4) 85 cosy.77.ser
DKN (4) 85 cosy
COSY
CDCl3
500 MHz
t-Bu
t-Bu–Si
O
O
O
BnO
N3
BnO
O
O
N3
O
Cbz
N
Bn
9
f2 (ppm)
f1 (ppm)

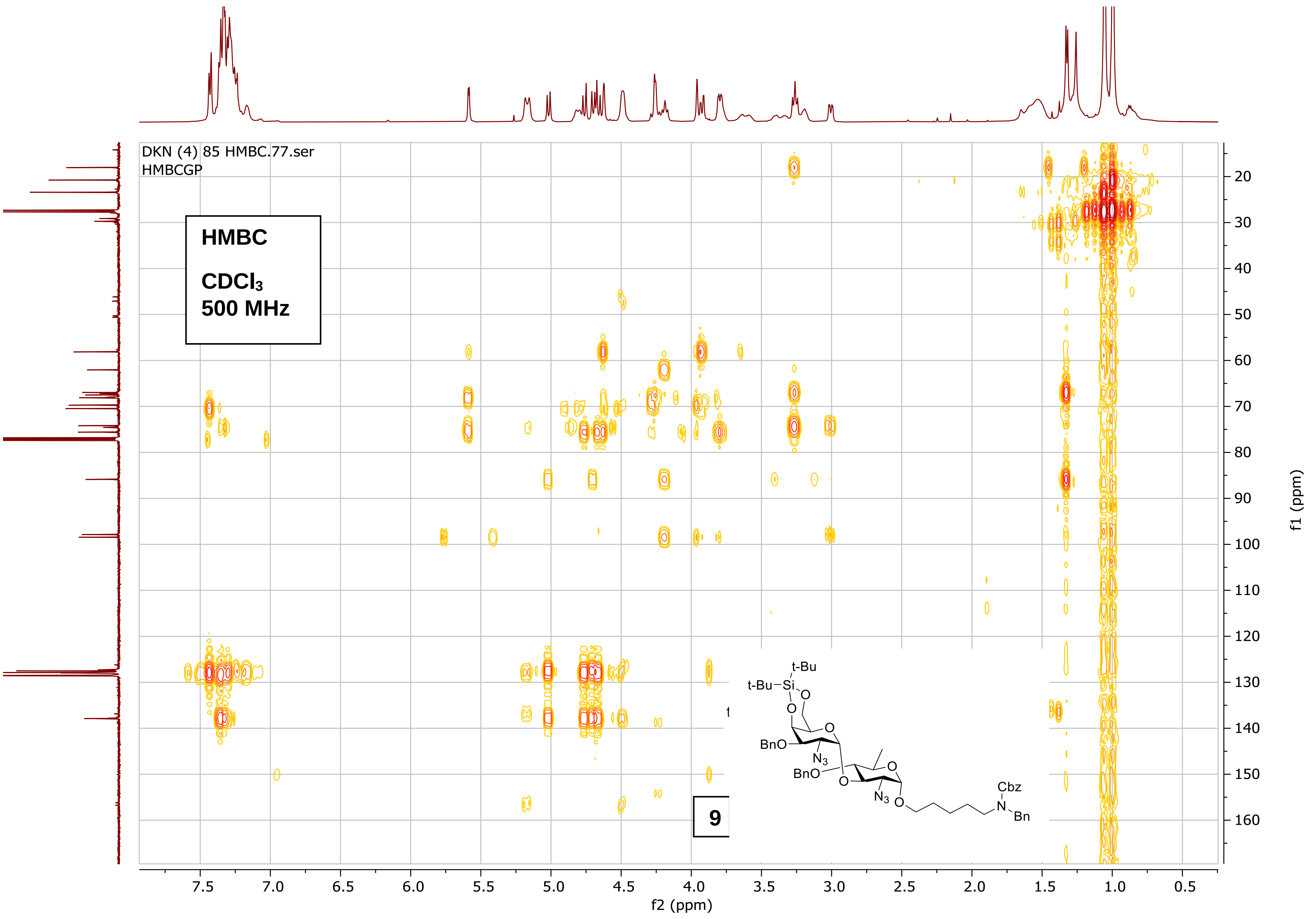
DKN (4) 85 HMBC.77.ser
HMBCGP
HMBC
CDCl3
500 MHz
t-Bu
t-Bu–Si
O
BnO
N3
BnO
N3
Cbz
N
Bn
9
f2 (ppm)
f1 (ppm)

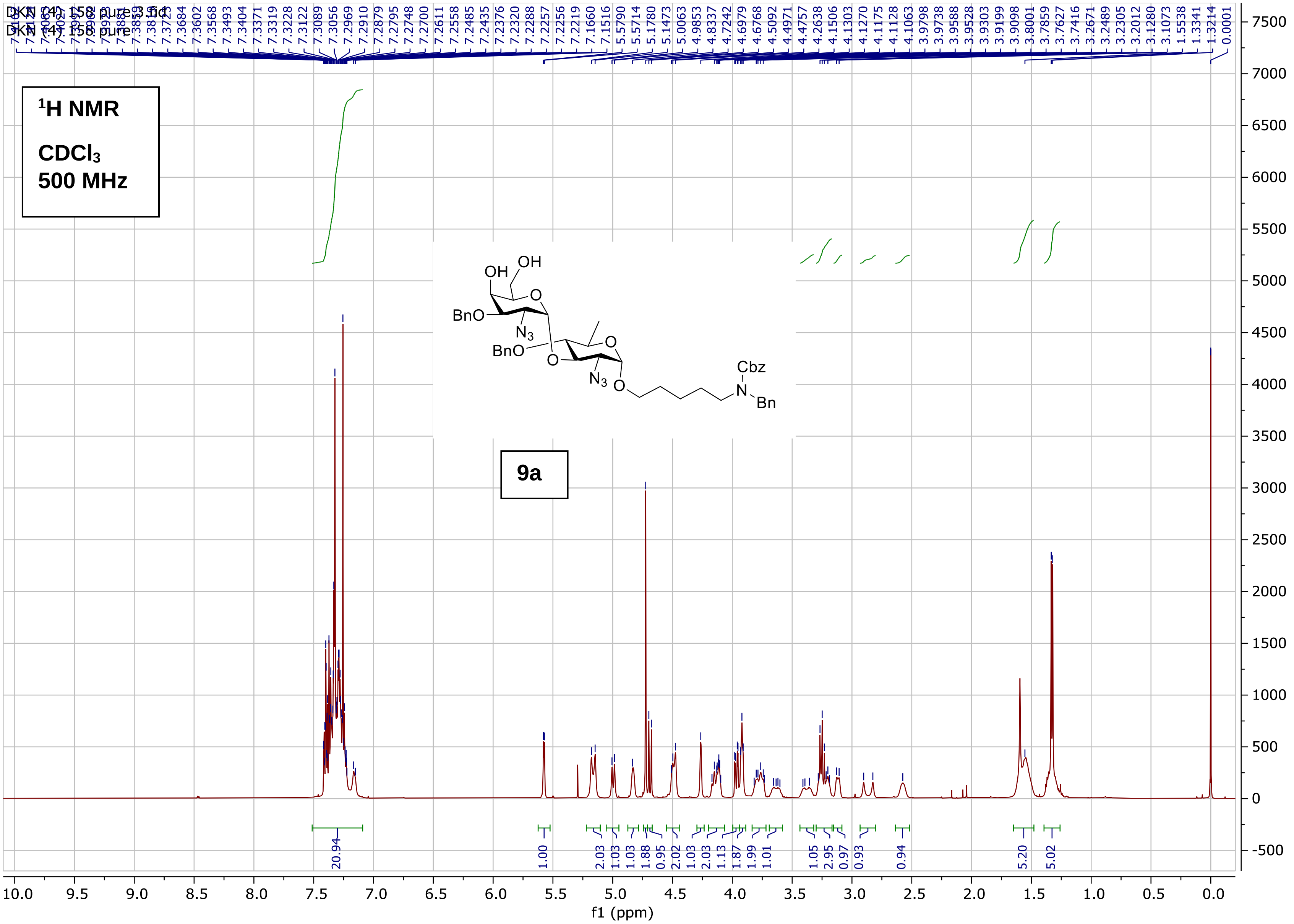
1H NMR
CDCl3
500 MHz
9a
f1 (ppm)

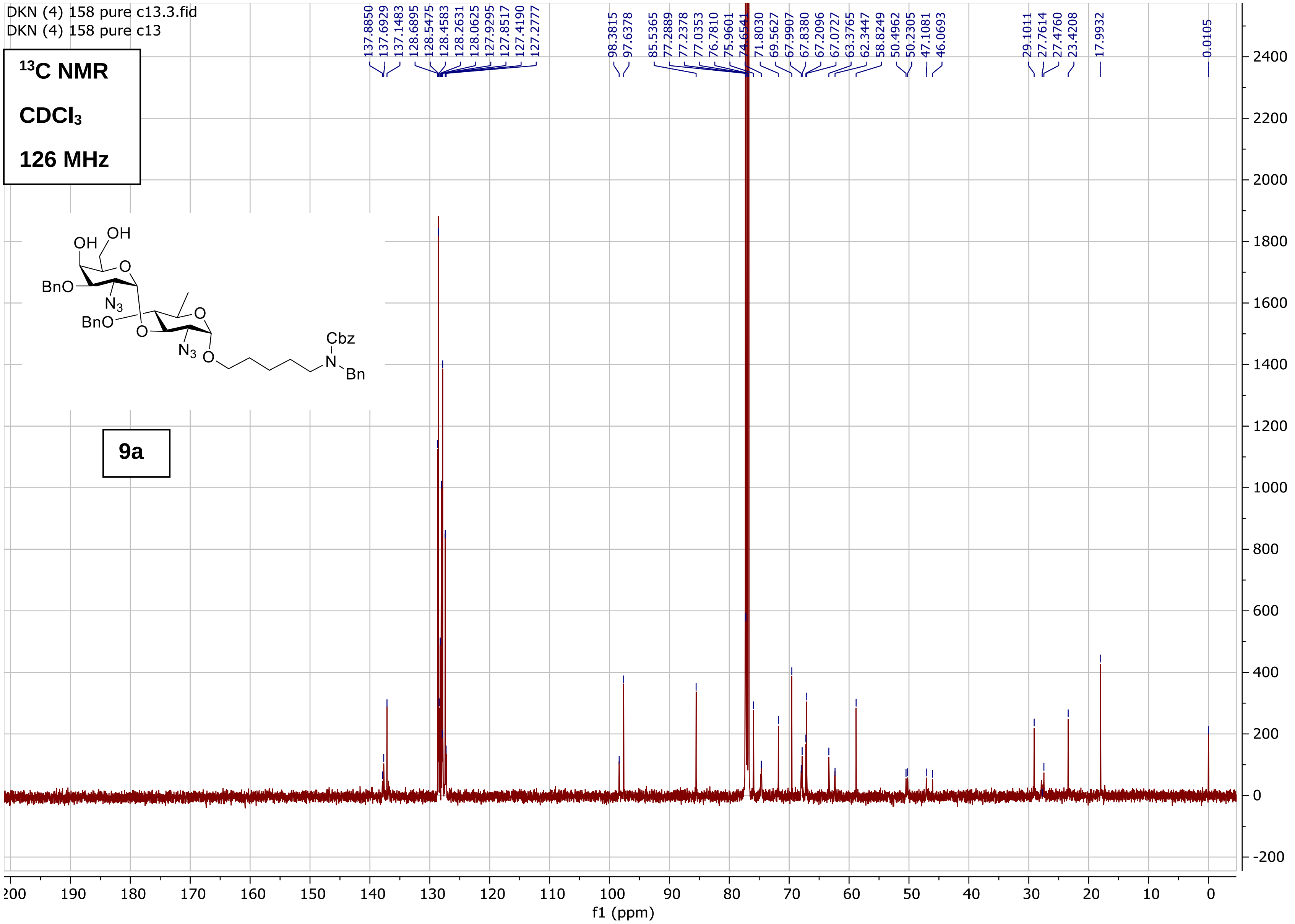
DKN (4) 158 pure c13.3.fid
DKN (4) 158 pure c13
^{13}C NMR
CDCl$_3$
126 MHz
OH
OH
O
BnO
N$_3$
BnO
O
O
N$_3$
O
Cbz
N
Bn
9a
137.8850
137.6929
137.1483
128.6895
128.5475
128.4583
128.2631
128.0625
127.9295
127.8517
127.4190
127.2777
98.3815
97.6378
85.5365
77.2889
77.2378
77.0353
76.7810
75.9601
74.6541
71.8030
69.5627
67.9907
67.8380
67.2096
67.0727
63.3765
62.3447
58.8249
50.4962
50.2305
47.1081
46.0693
29.1011
27.7614
27.4760
23.4208
17.9932
0.0105
f1 (ppm)

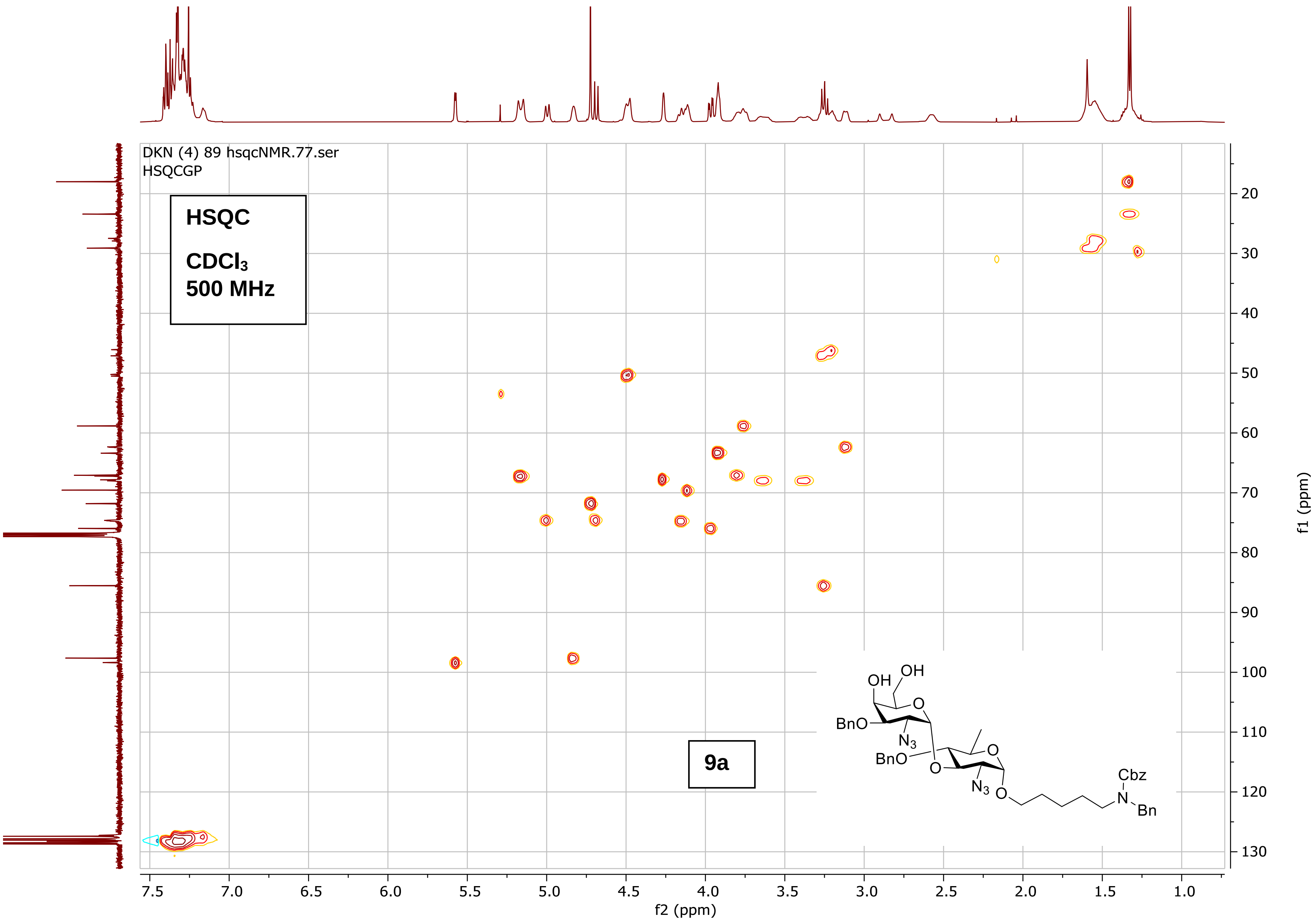
DKN (4) 89 hsqcNMR.77.ser
HSQCGP
HSQC
CDCl3
500 MHz
9a
OH
OH
O
BnO
N3
BnO
O
O
N3
O
Cbz
N
Bn
f2 (ppm)
f1 (ppm)

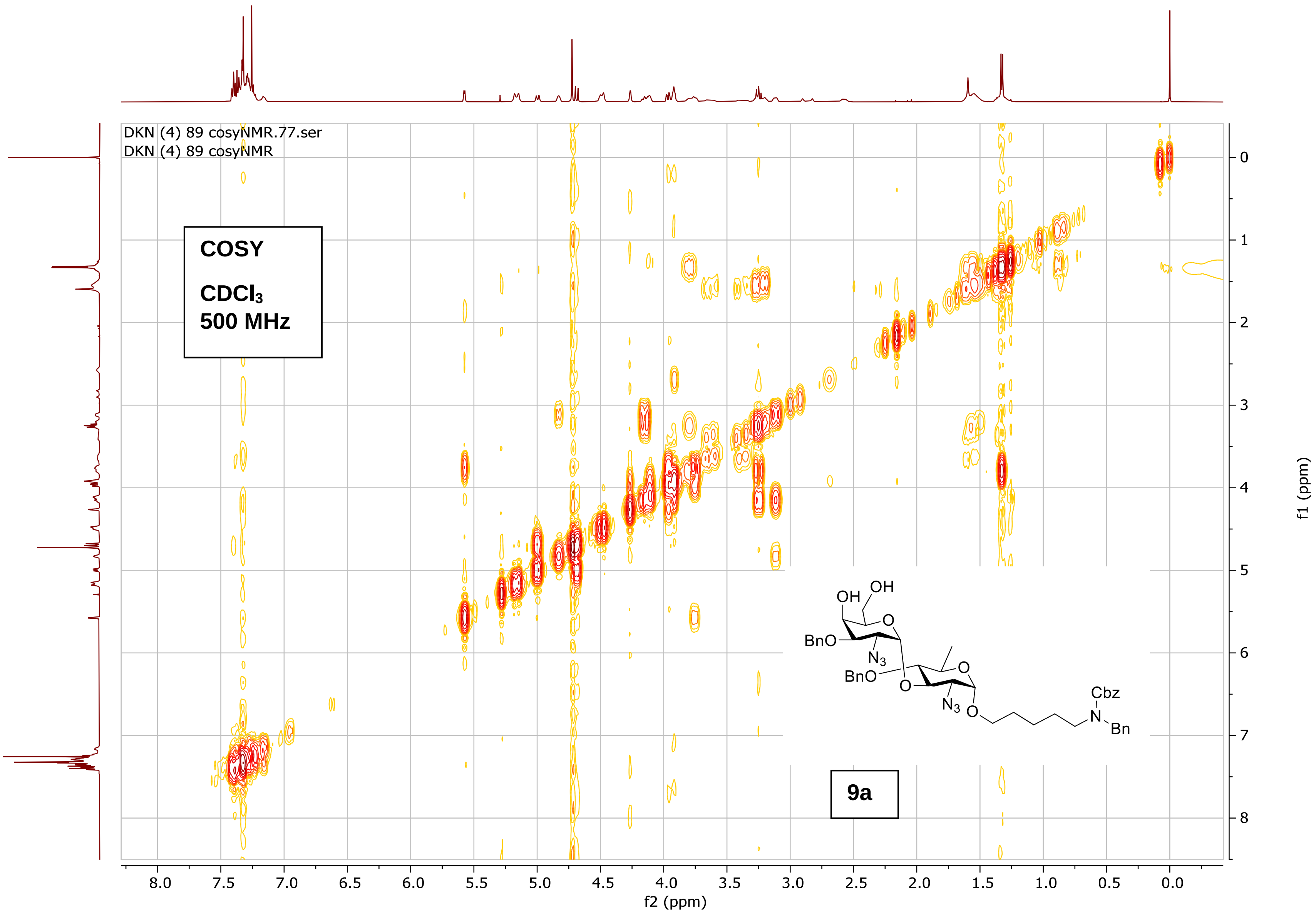
DKN (4) 89 cosyNMR.77.ser
DKN (4) 89 cosyNMR
COSY
CDCl3
500 MHz
9a
f2 (ppm)
f1 (ppm)

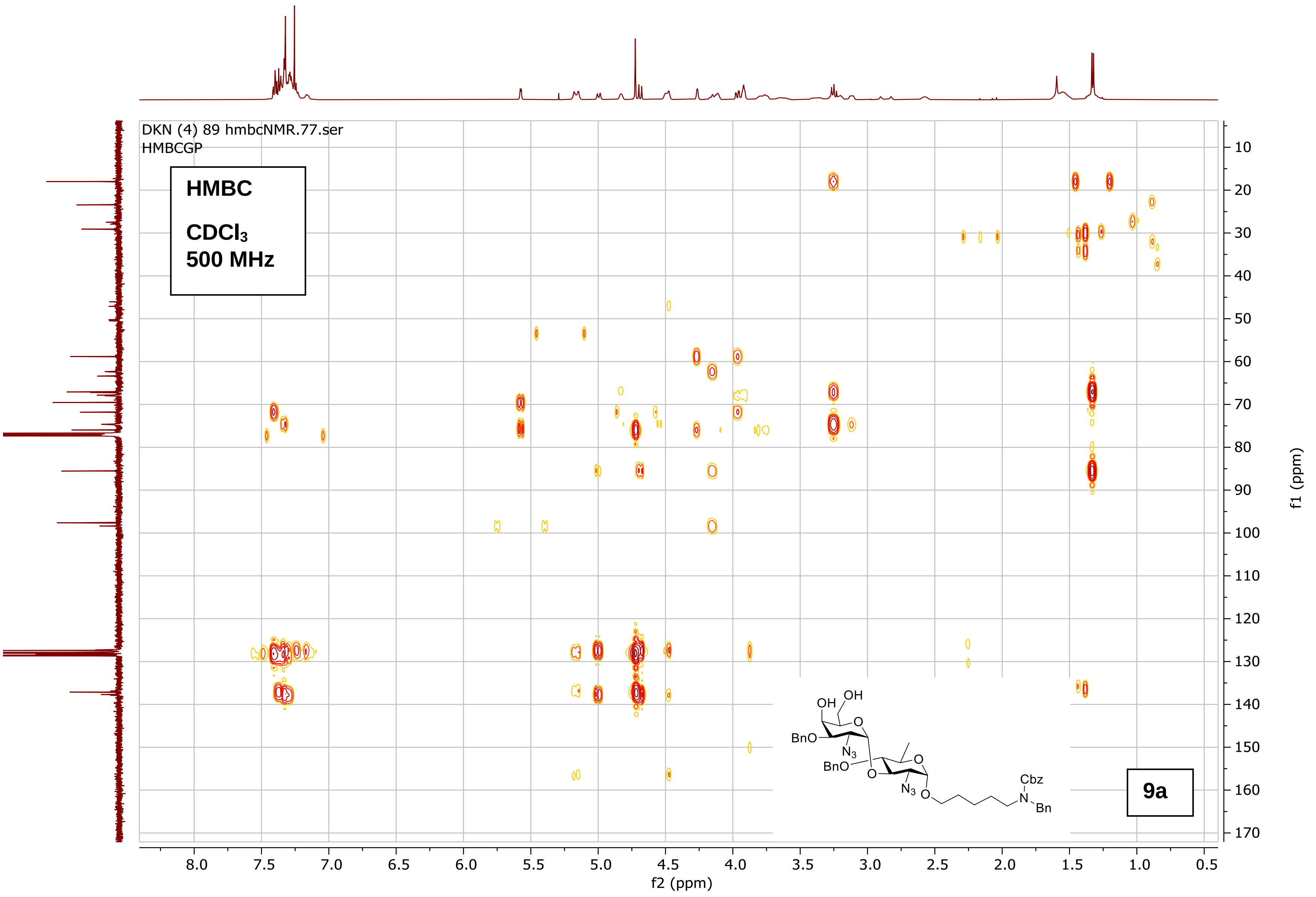
DKN (4) 89 hmbcNMR.77.ser
HMBCGP
HMBC
CDCl3
500 MHz
f2 (ppm)
f1 (ppm)
OH
OH
BnO
N3
BnO
N3
Cbz
N
Bn
9a

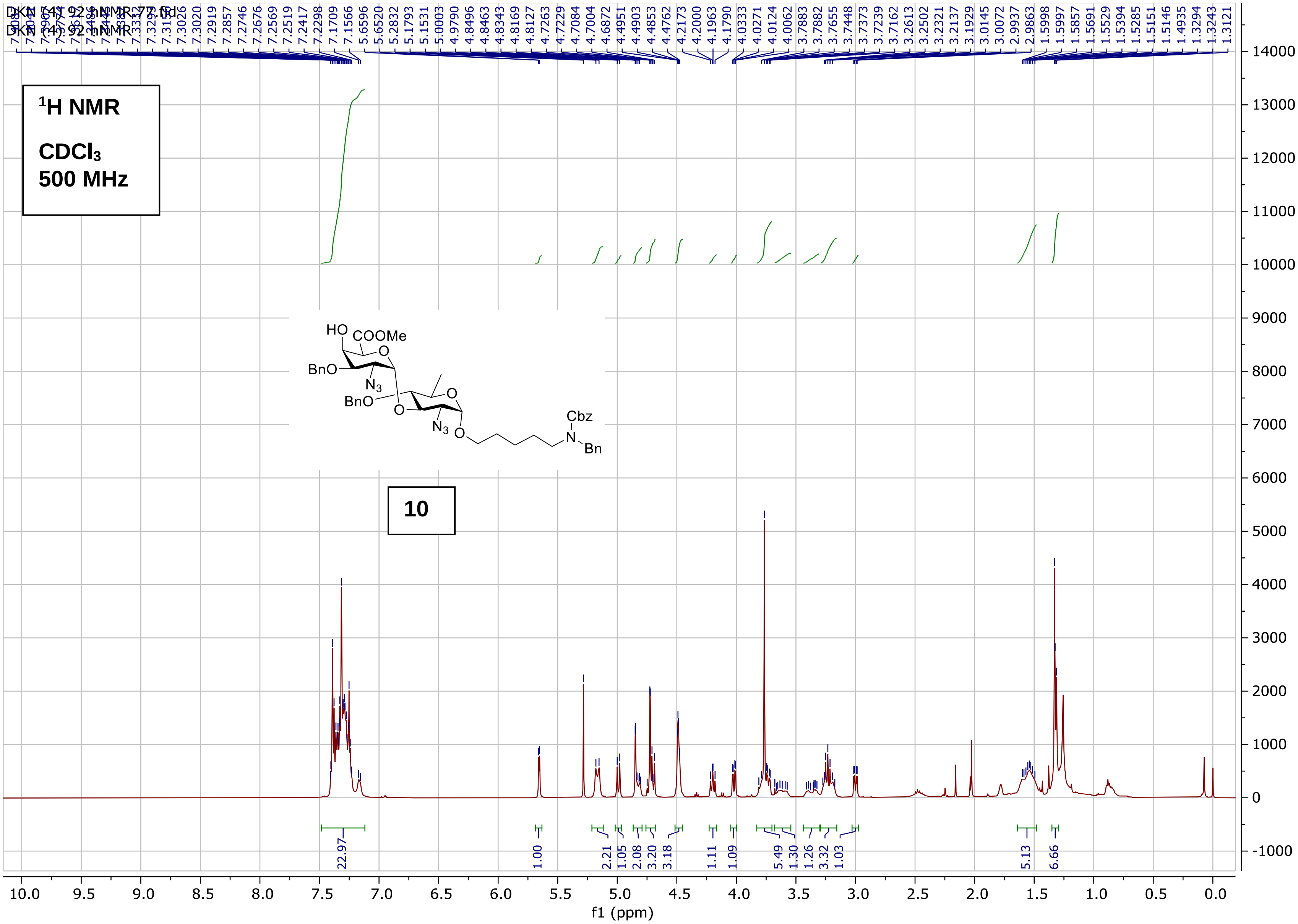

1H NMR
CDCl3
500 MHz
10
HO
COOMe
BnO
N3
BnO
N3
Cbz
N
Bn
f1 (ppm)

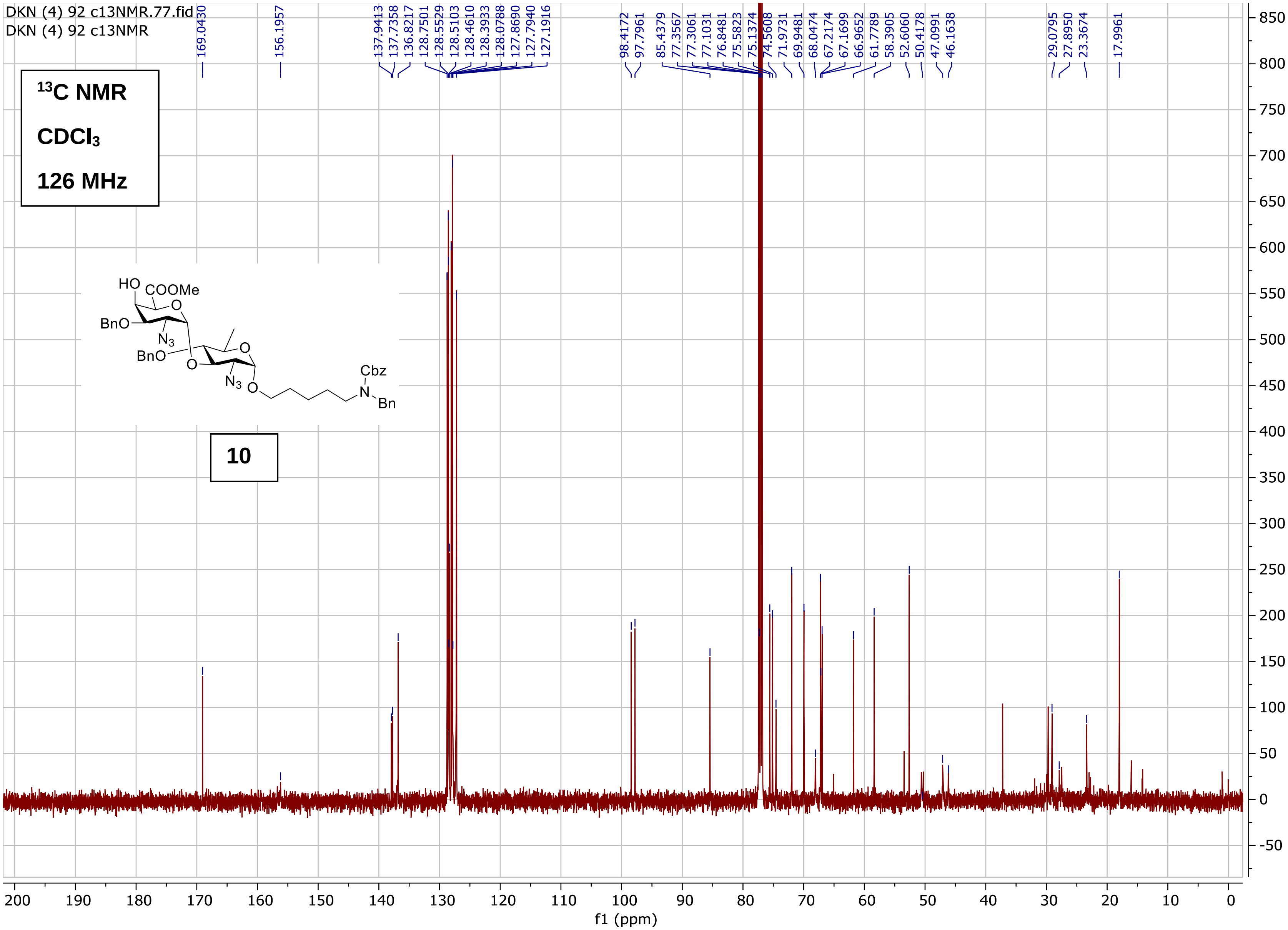

DKN (4) 92 c13NMR.77.fid
DKN (4) 92 c13NMR
13C NMR
CDCl3
126 MHz
HO
COOMe
O
BnO
N3
BnO
O
O
N3
O
Cbz
N
Bn
10
169.0430
156.1957
137.9413
137.7358
136.8217
128.7501
128.5529
128.5103
128.4610
128.3933
128.0788
127.8690
127.7940
127.1916
98.4172
97.7961
85.4379
77.3567
77.3061
77.1031
76.8481
75.5823
75.1374
74.5608
71.9731
69.9481
68.0474
67.2174
67.1699
66.9652
61.7789
58.3905
52.6060
50.4178
47.0991
46.1638
29.0795
27.8950
23.3674
17.9961
f1 (ppm)

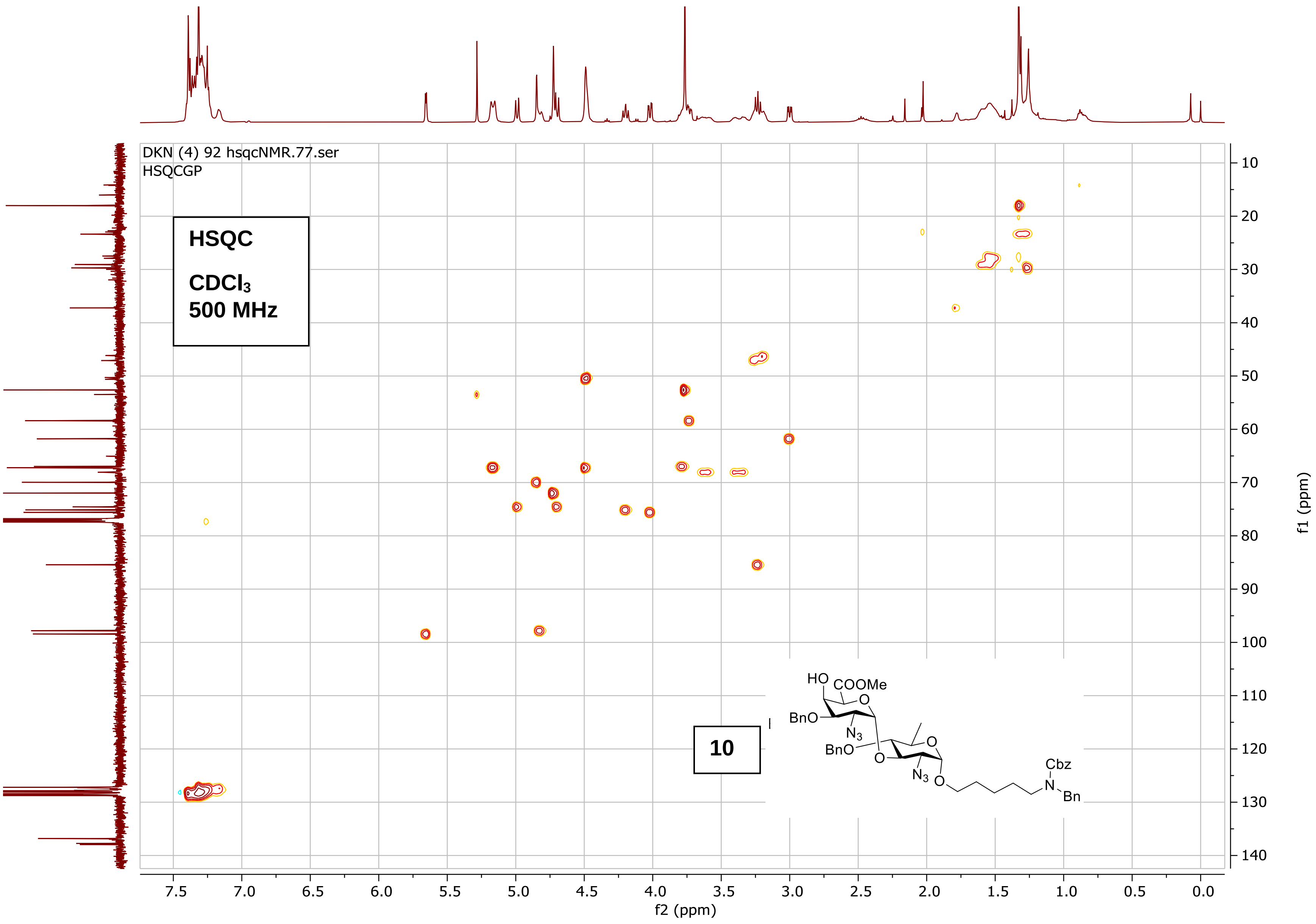
DKN (4) 92 hsqcNMR.77.ser
HSQCGP
HSQC
CDCl3
500 MHz
10
HO
COOMe
BnO
N3
BnO
O
Cbz
N
Bn
f2 (ppm)
f1 (ppm)

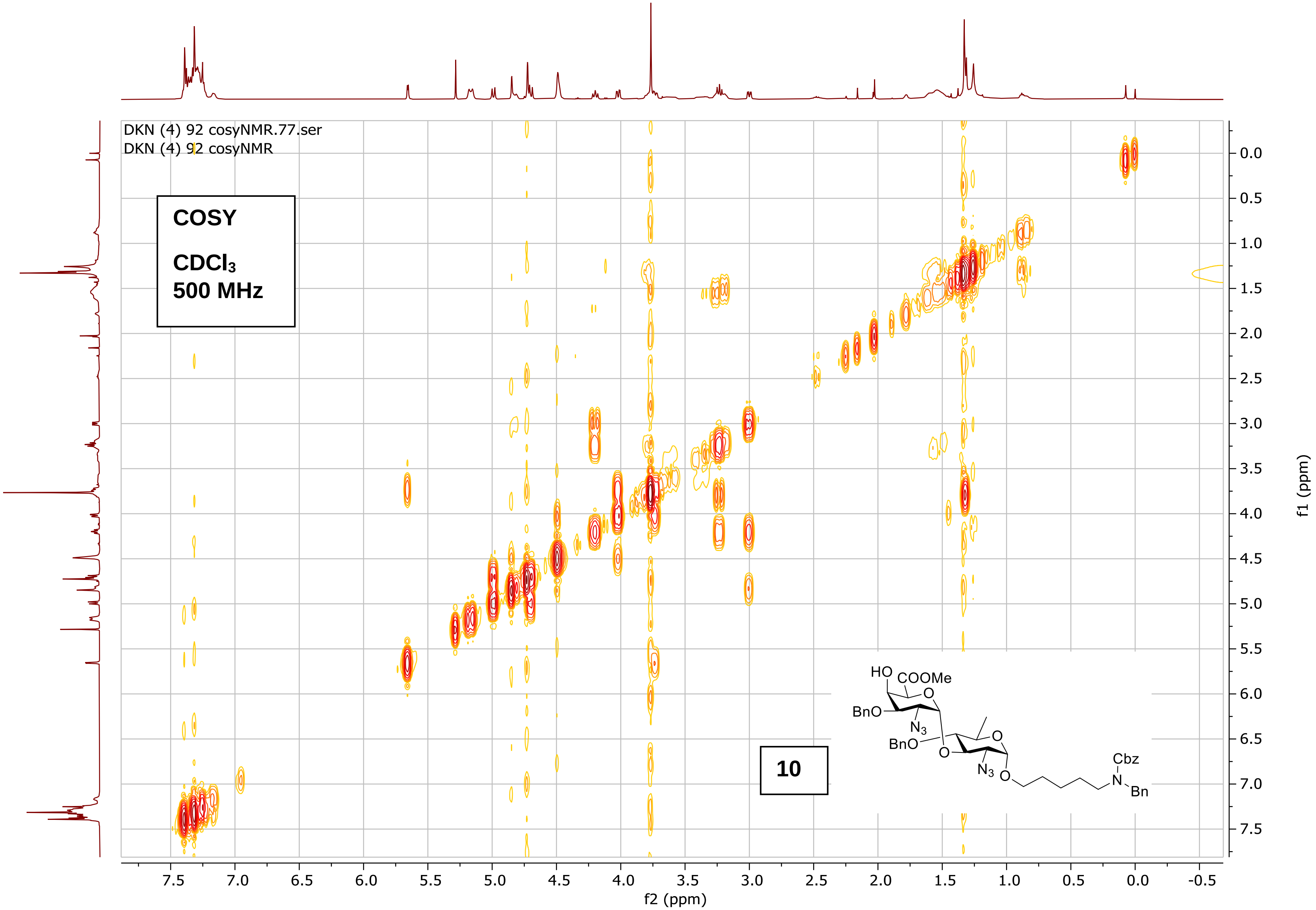

DKN (4) 92 cosyNMR.77.ser
DKN (4) 92 cosyNMR
COSY
CDCl3
500 MHz
10
HO
COOMe
BnO
N3
BnO
O
N3
O
Cbz
N
Bn
f2 (ppm)
f1 (ppm)

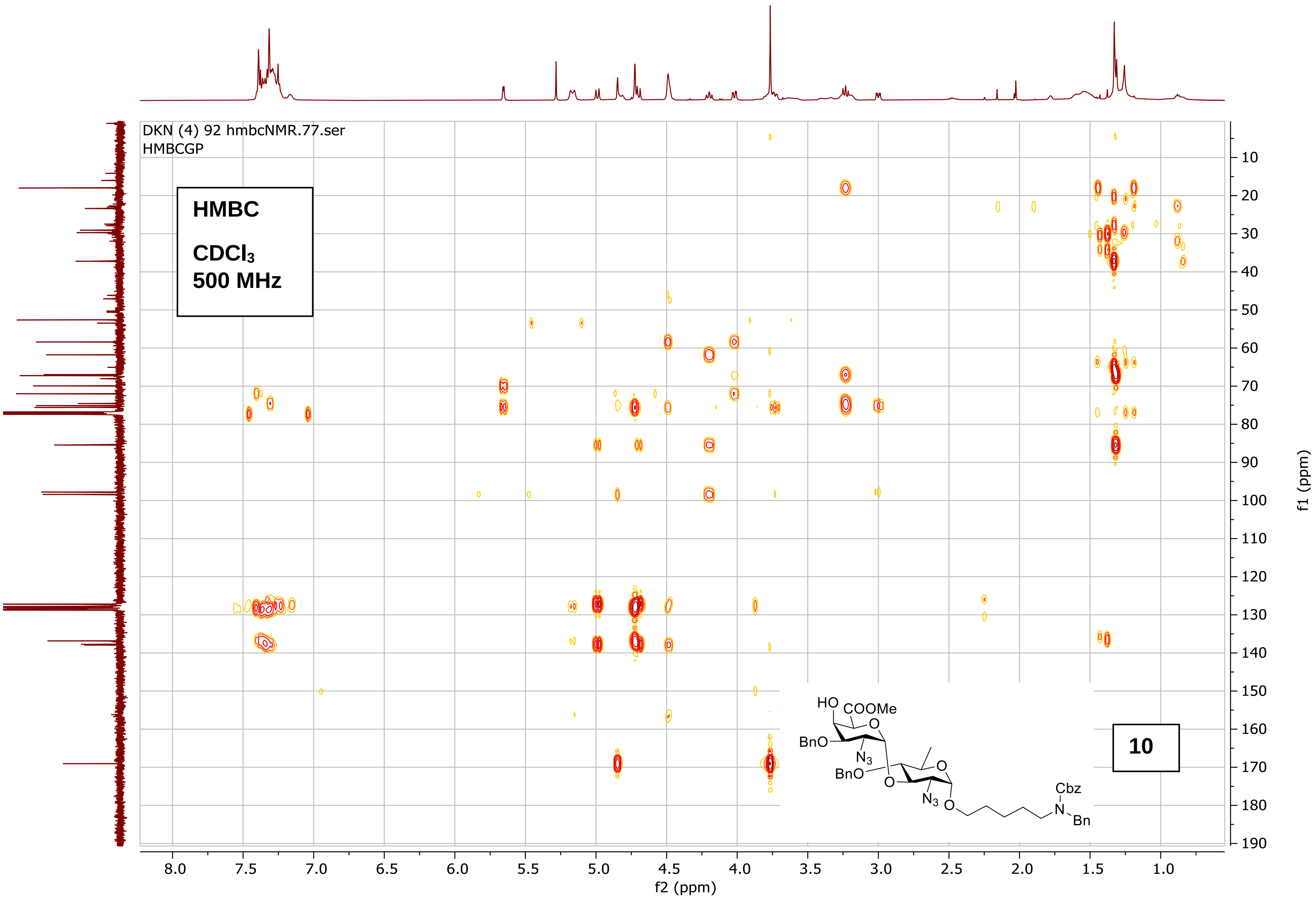
DKN (4) 92 hmbcNMR.77.ser
HMBCGP
HMBC
$CDCl_3$
500 MHz
HO
COOMe
O
BnO
N_3
BnO
O
O
N_3
O
Cbz
N
Bn
10
f2 (ppm)
f1 (ppm)

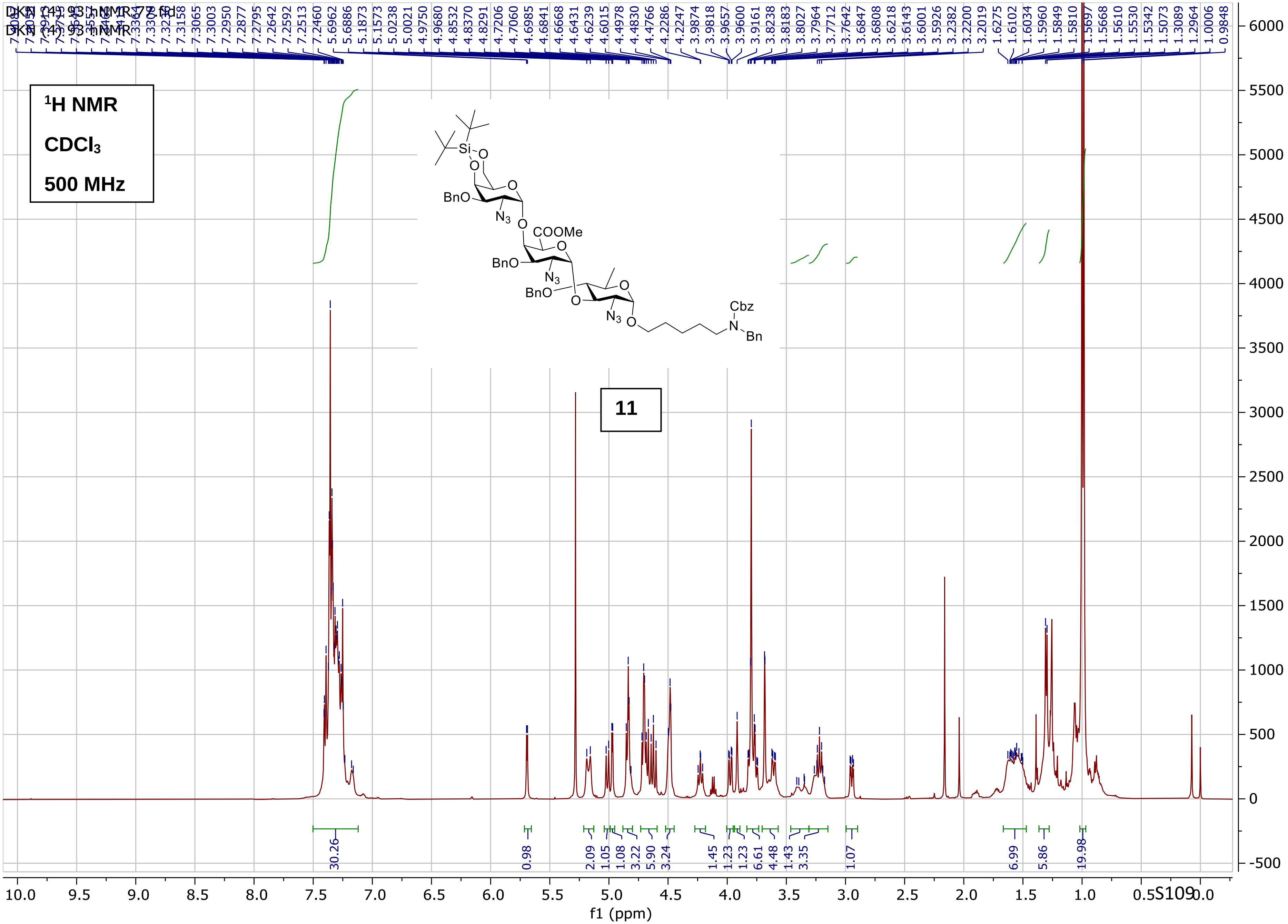

1H NMR
CDCl3
500 MHz
11
f1 (ppm)

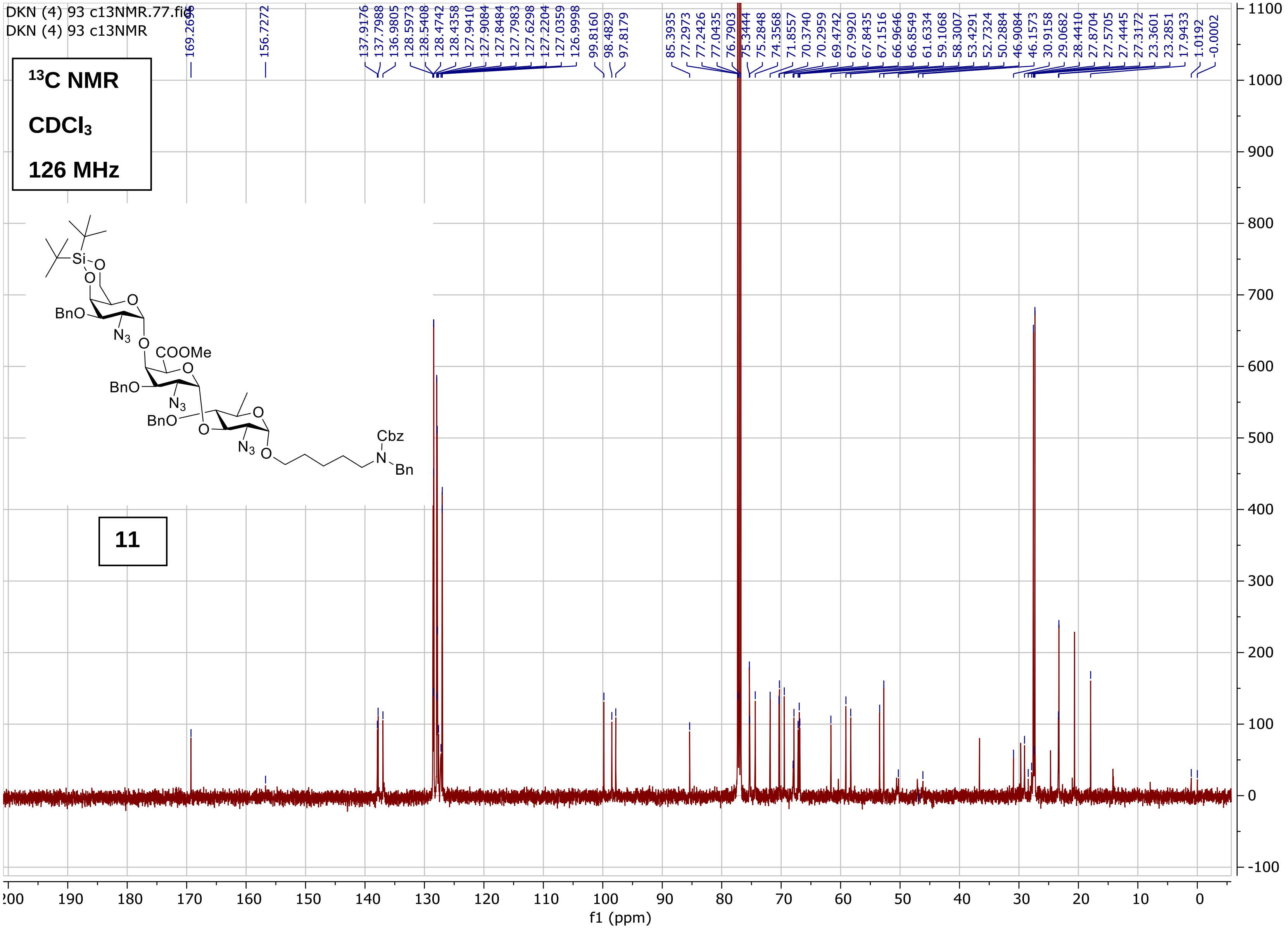
DKN (4) 93 c13NMR.77.fid
DKN (4) 93 c13NMR
169.2696
156.7272
137.9176
137.7988
136.9805
128.5973
128.5408
128.4742
128.4358
127.9410
127.9084
127.8484
127.7983
127.6298
127.2204
127.0359
126.9998
99.8160
98.4829
97.8179
85.3935
77.2973
77.2426
77.0435
76.7903
75.3444
75.2848
74.3568
71.8557
70.3740
70.2959
69.4742
67.9920
67.8435
67.1516
66.9646
66.8549
61.6334
59.1068
58.3007
53.4291
52.7324
50.2884
46.9084
46.1573
30.9158
29.0682
28.4410
27.8704
27.5705
27.4445
27.3172
23.3601
23.2851
17.9433
1.0192
-0.0002
^{13}C NMR
$CDCl_3$
126 MHz
BnO
N_3
COOMe
Cbz
N
Bn
11
f1 (ppm)

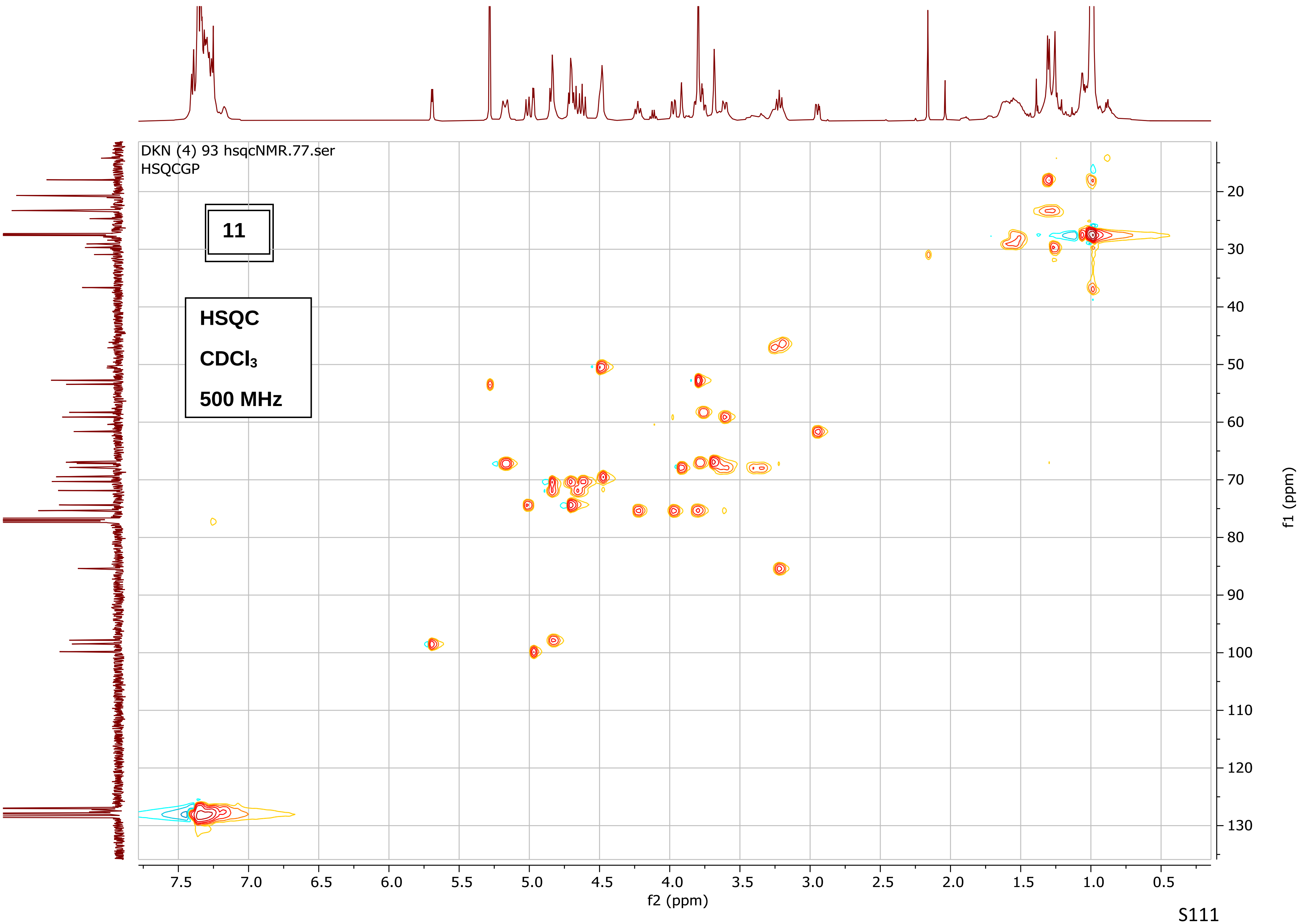

DKN (4) 93 hsqcNMR.77.ser
HSQCGP
11
HSQC
CDCl3
500 MHz
f2 (ppm)
f1 (ppm)

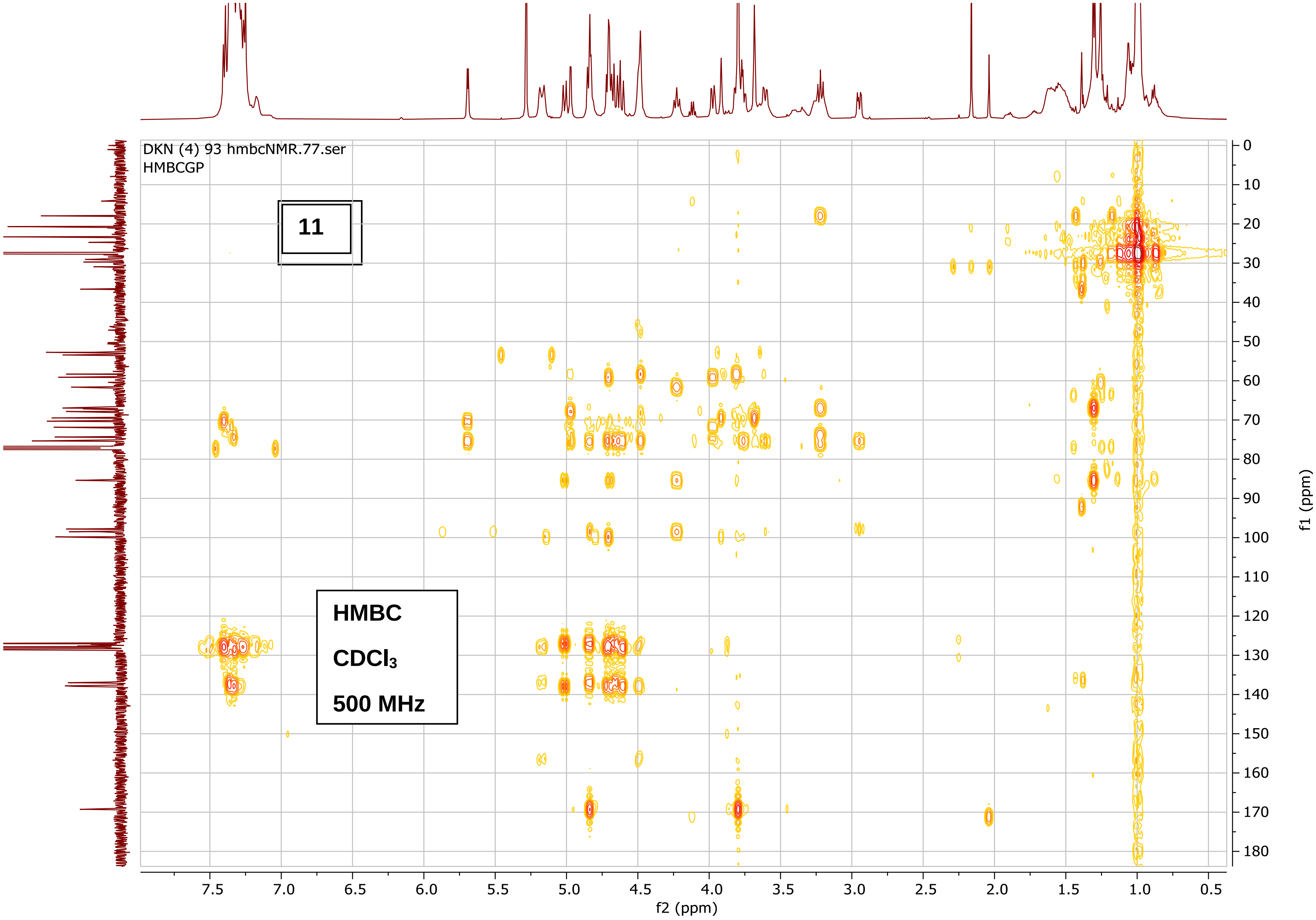
DKN (4) 93 hmbcNMR.77.ser
HMBCGP
11
HMBC
CDCl3
500 MHz
f2 (ppm)
f1 (ppm)

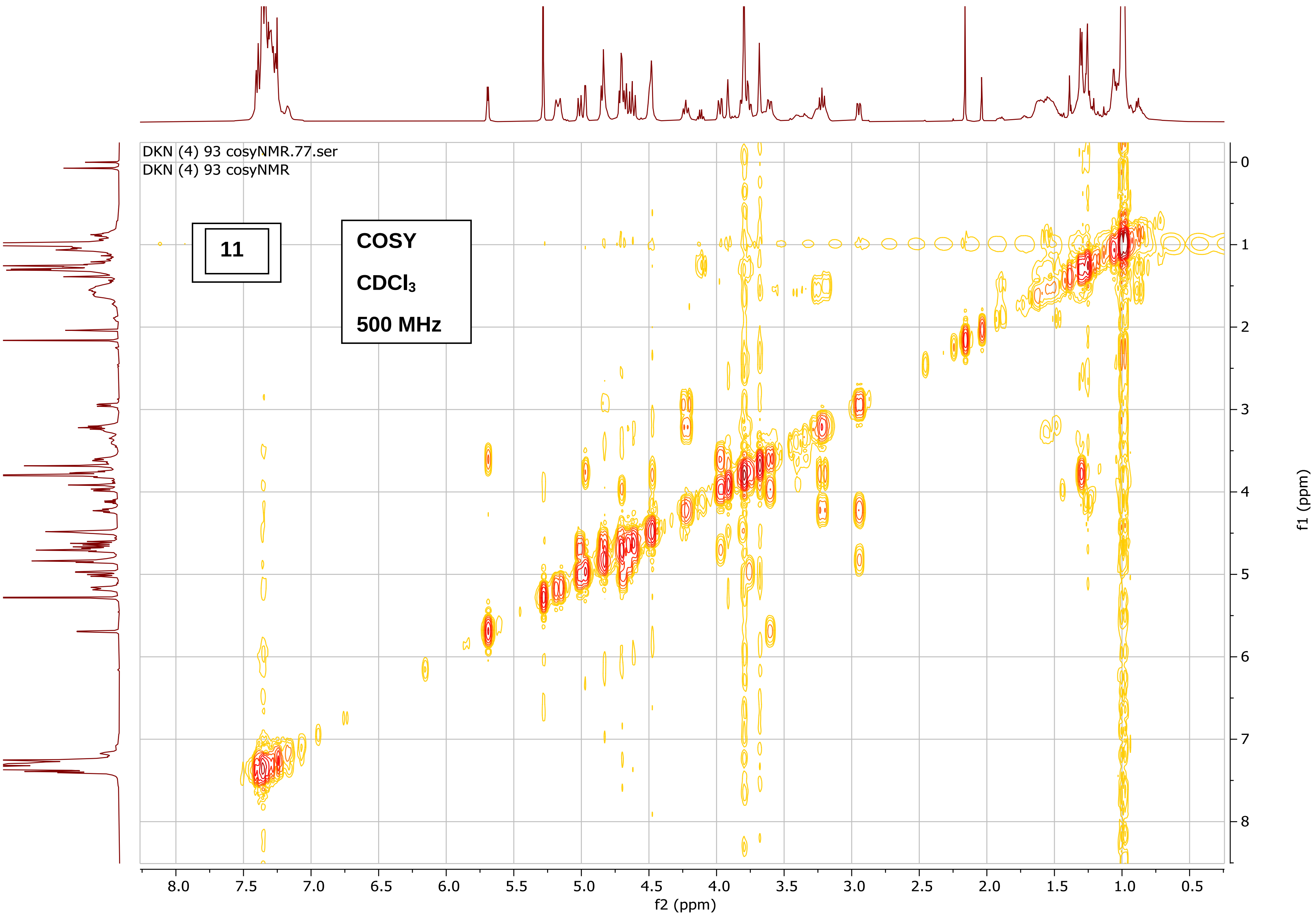

DKN (4) 93 cosyNMR.77.ser
DKN (4) 93 cosyNMR
11
COSY
CDCl3
500 MHz
f2 (ppm)
f1 (ppm)

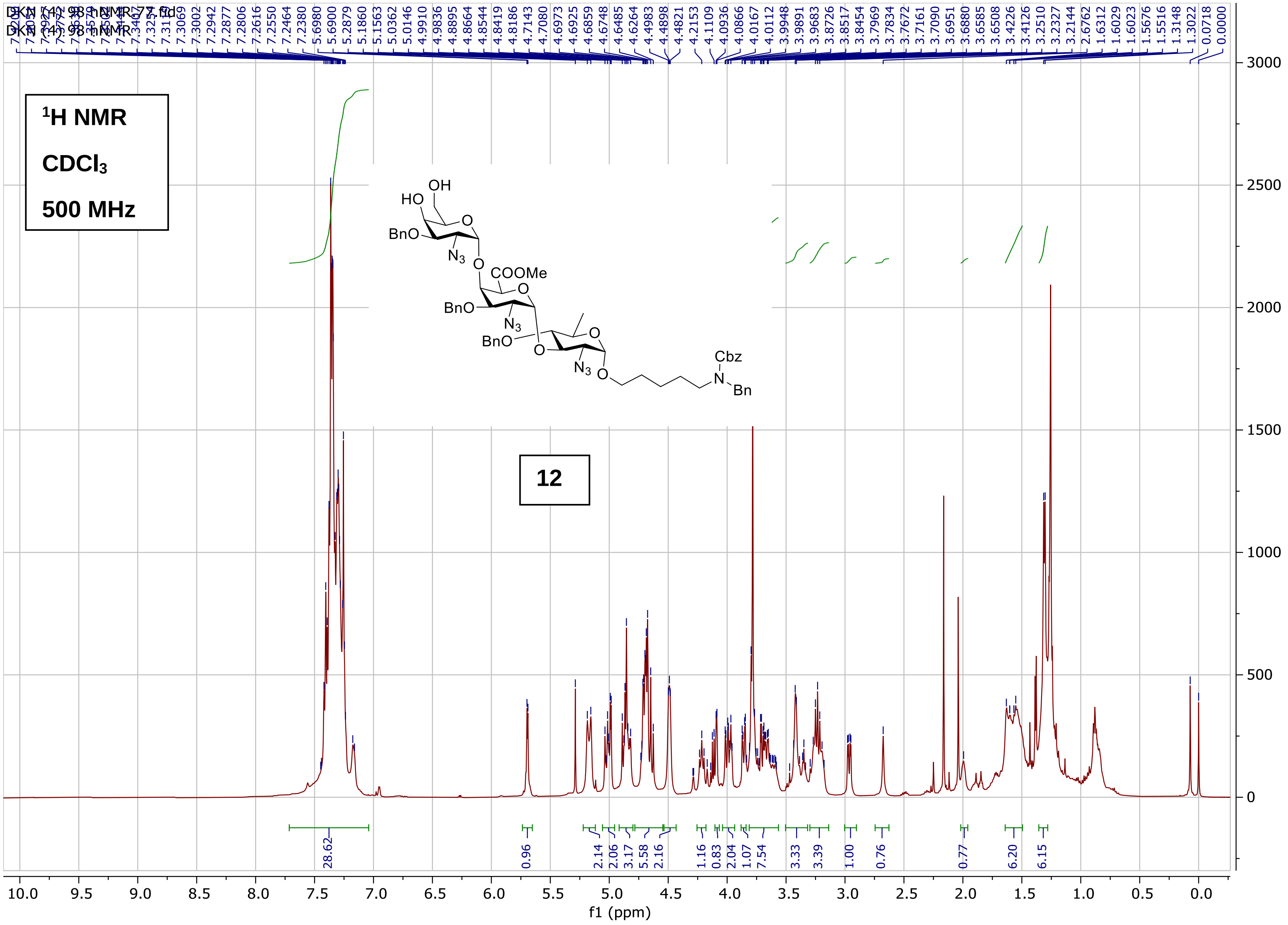

¹H NMR
CDCl3
500 MHz
12
f1 (ppm)

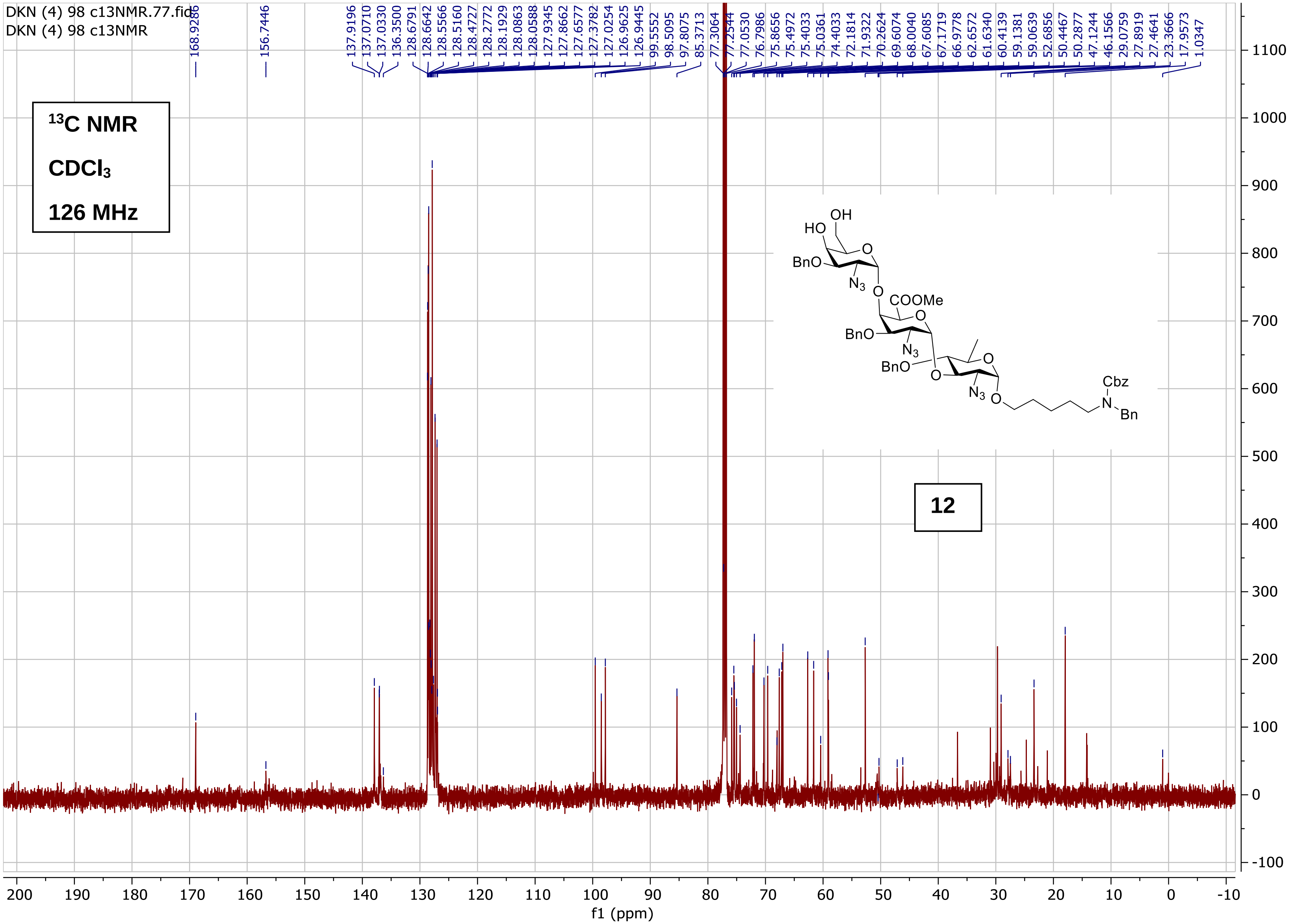
DKN (4) 98 c13NMR.77.fid
DKN (4) 98 c13NMR
168.9286
156.7446
137.9196
137.0710
137.0330
136.3500
128.6791
128.6642
128.5566
128.5160
128.4727
128.2772
128.1929
128.0863
128.0588
127.9345
127.8662
127.6577
127.3782
127.0254
126.9625
126.9445
99.5552
98.5095
97.8075
85.3713
77.3064
77.2544
77.0530
76.7986
75.8656
75.4972
75.4033
75.0361
74.4033
72.1814
71.9322
70.2624
69.6074
68.0040
67.6085
67.1719
66.9778
62.6572
61.6340
60.4139
59.1381
59.0639
52.6856
50.4467
50.2877
47.1244
46.1566
29.0759
27.8919
27.4641
23.3666
17.9573
1.0347
13C NMR
CDCl3
126 MHz
HO
OH
O
BnO
N3
O
COOMe
O
BnO
N3
BnO
O
O
N3
O
Cbz
N
Bn
12
f1 (ppm)

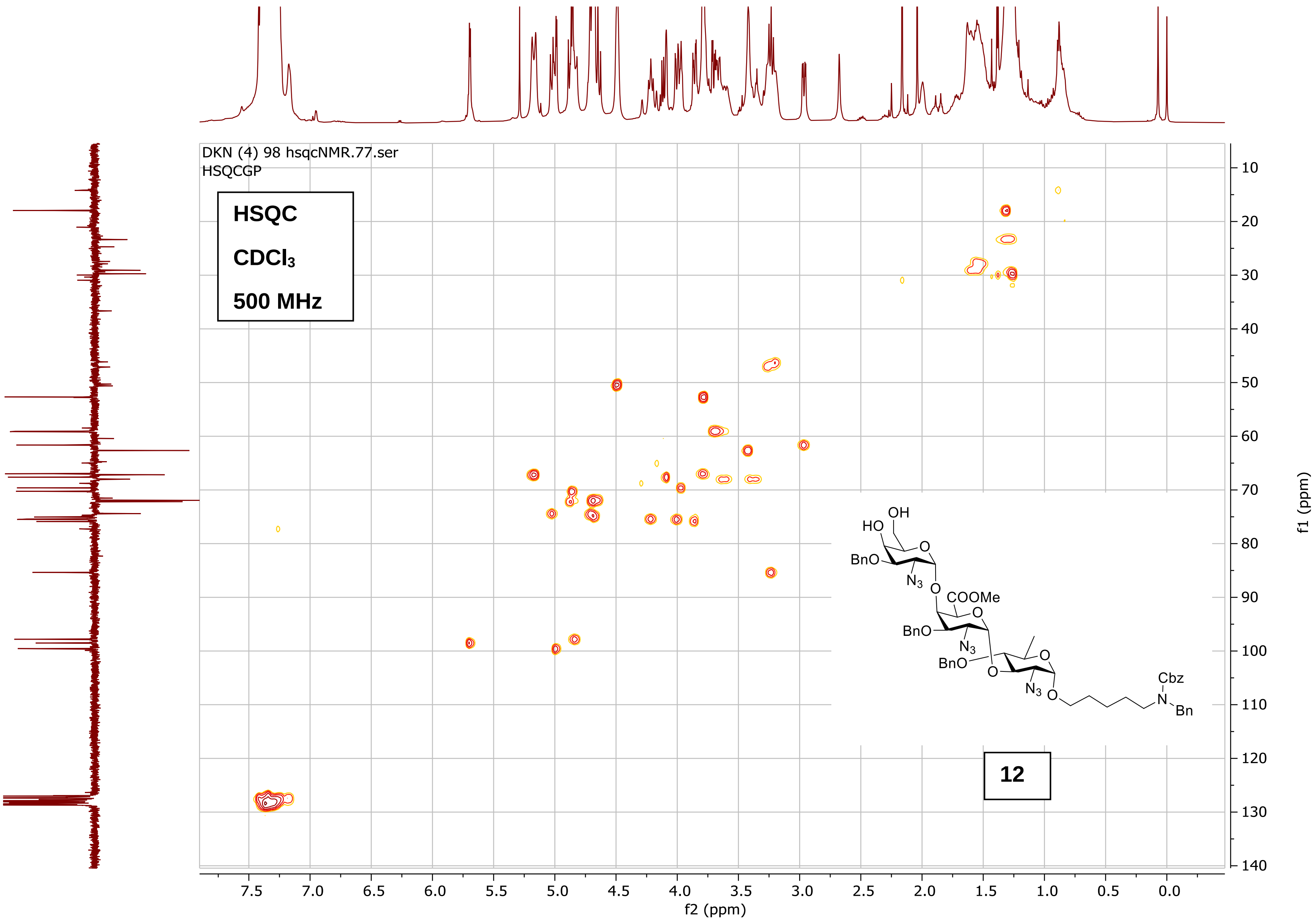

DKN (4) 98 hsqcNMR.77.ser
HSQCGP
HSQC
CDCl3
500 MHz
OH
HO
O
BnO
N3
O
COOMe
O
BnO
N3
BnO
O
O
N3
O
Cbz
N
Bn
12
f2 (ppm)
f1 (ppm)

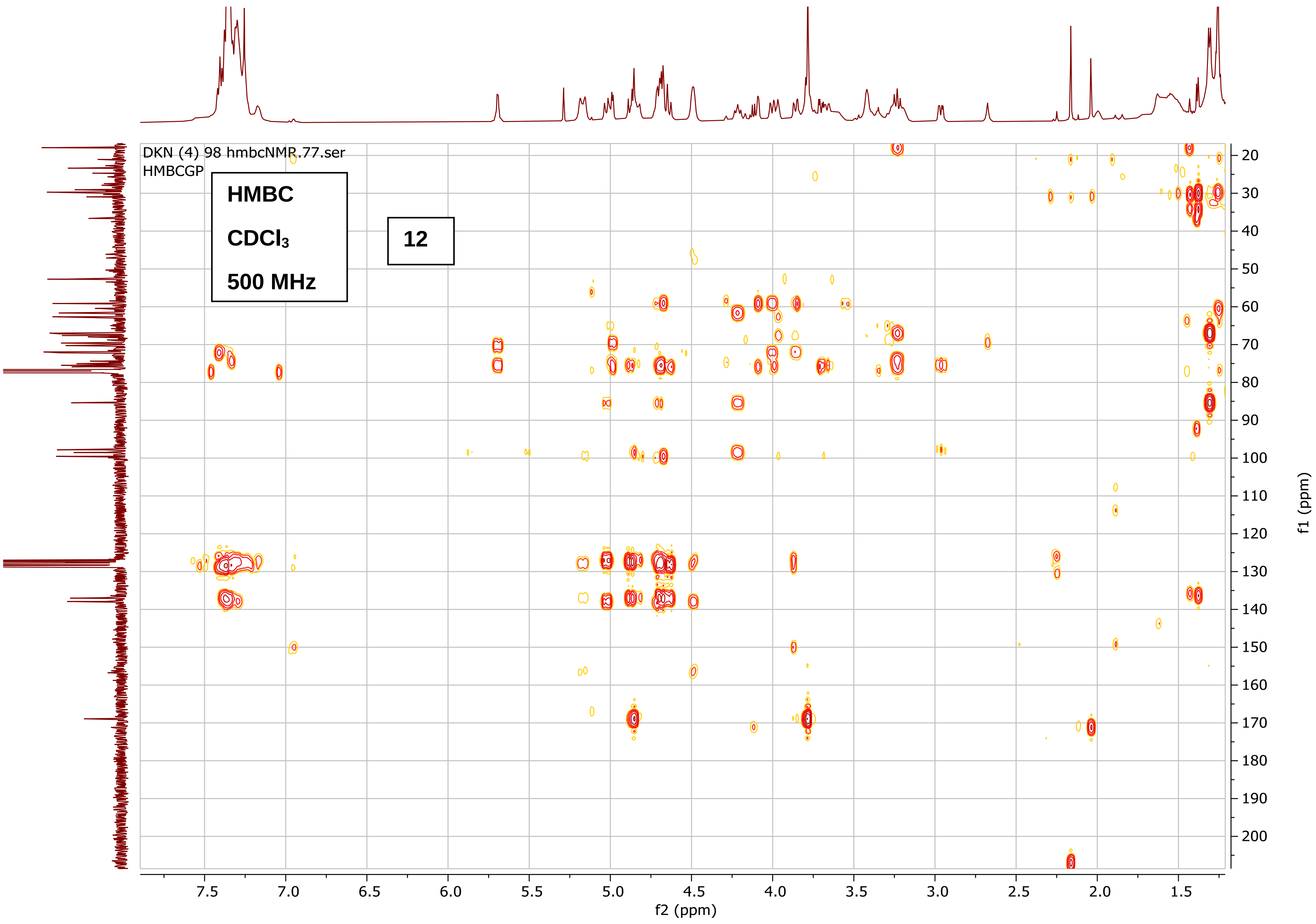
DKN (4) 98 hmbcNMR.77.ser
HMBCGP
HMBC
CDCl3
500 MHz
12
f2 (ppm)
f1 (ppm)

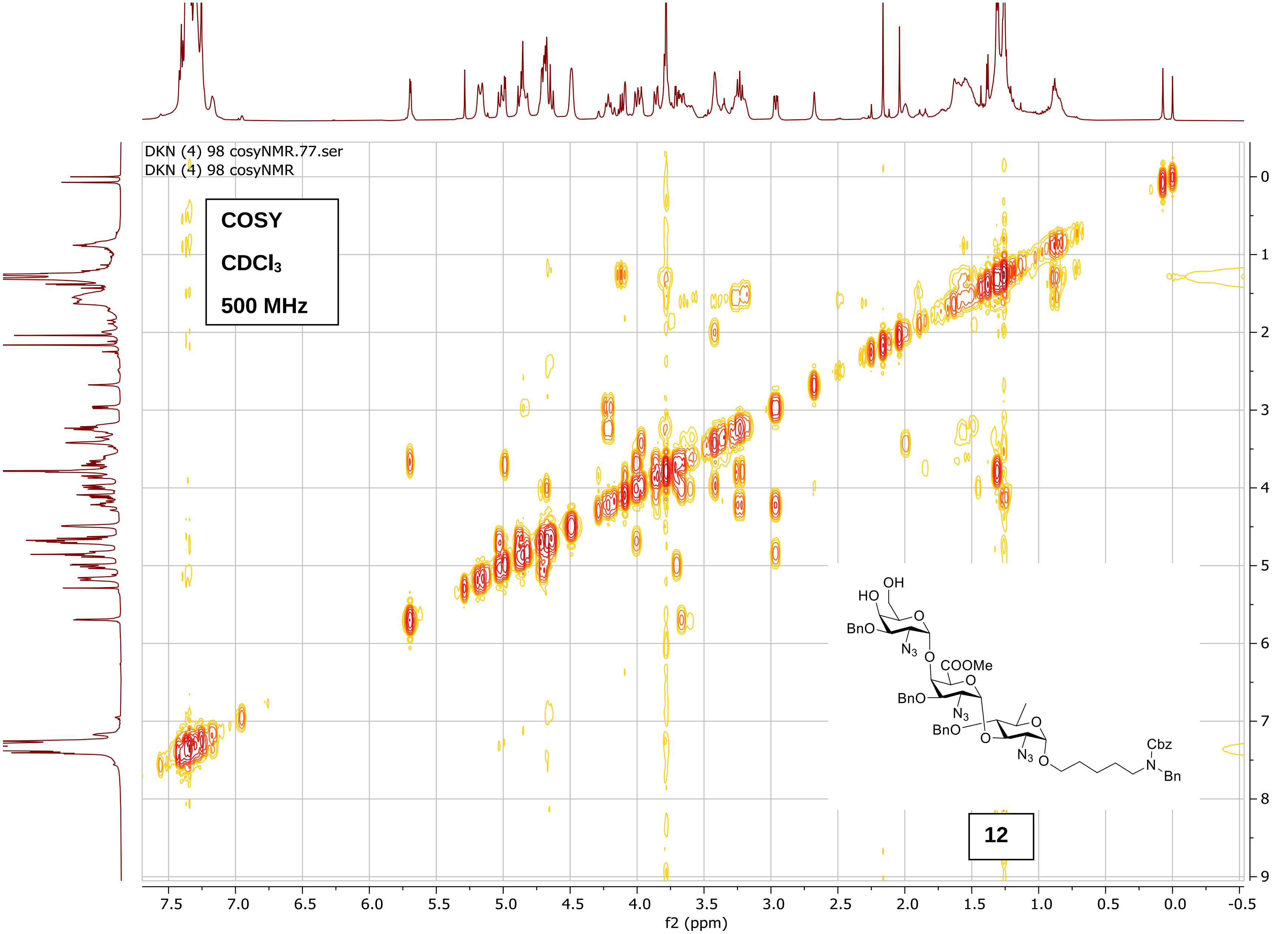

DKN (4) 98 cosyNMR.77.ser
DKN (4) 98 cosyNMR
COSY
CDCl3
500 MHz
HO
OH
BnO
N3
O
COOMe
BnO
N3
BnO
O
N3
Cbz
N
Bn
12
f2 (ppm)
f1 (ppm)

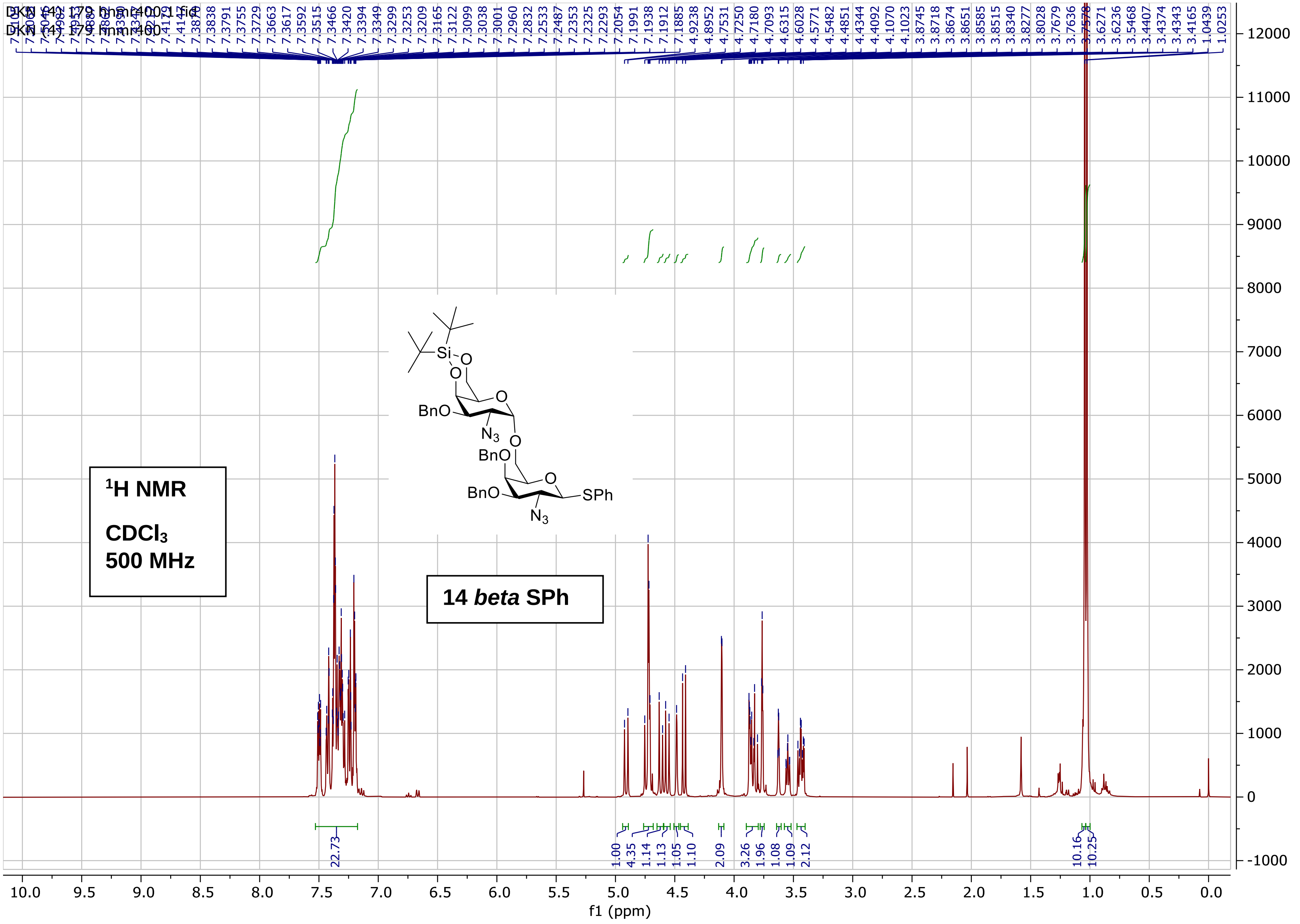
1H NMR
CDCl3
500 MHz
14 beta SPh
BnO
N3
SPh
f1 (ppm)

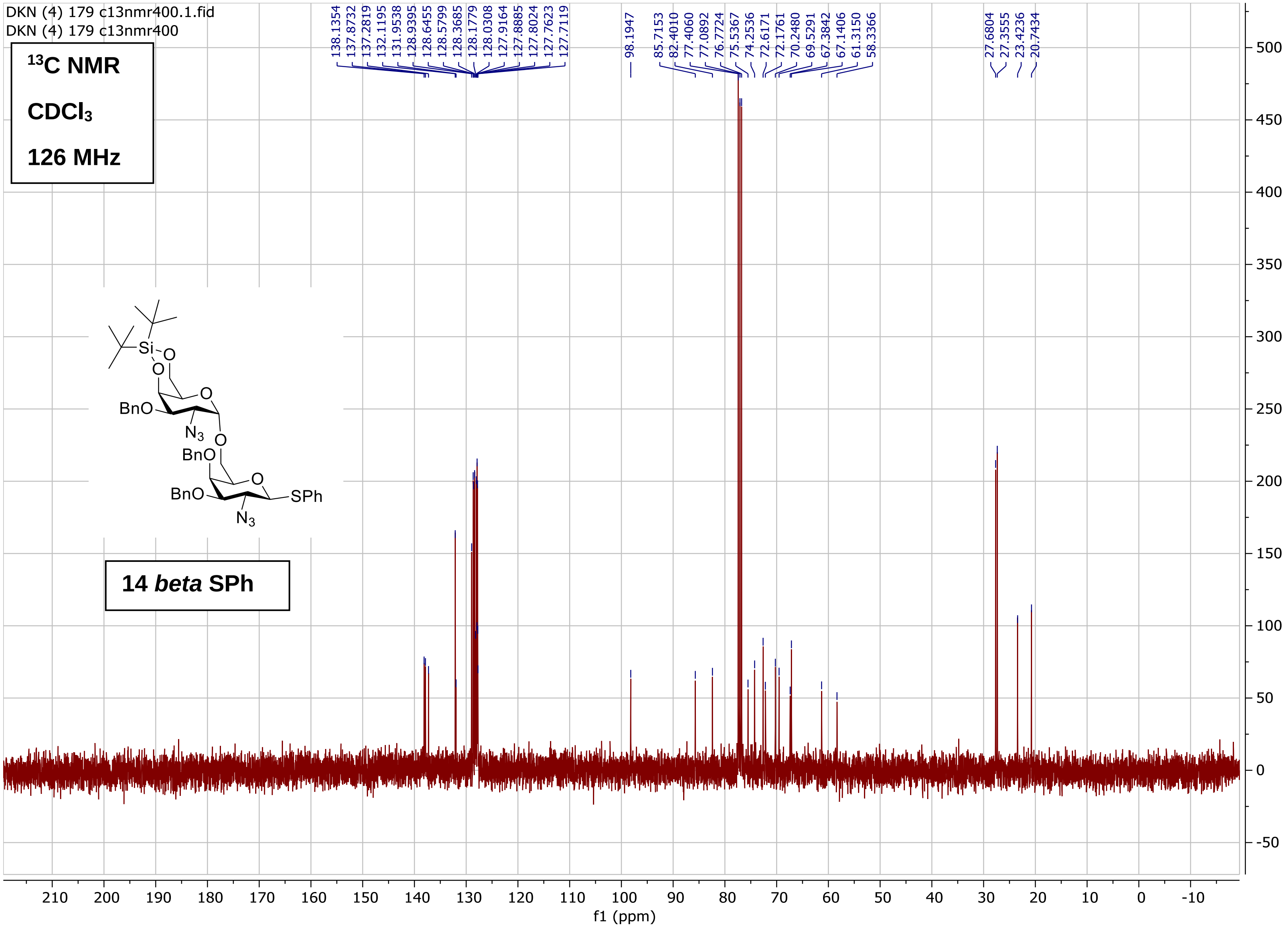
DKN (4) 179 c13nmr400.1.fid
DKN (4) 179 c13nmr400
^{13}C NMR
$CDCl_3$
126 MHz
138.1354
137.8732
137.2819
132.1195
131.9538
128.9395
128.6455
128.5799
128.3685
128.1779
128.0308
127.9164
127.8885
127.8024
127.7623
127.7119
98.1947
85.7153
82.4010
77.4060
77.0892
76.7724
75.5367
74.2536
72.6171
72.1761
70.2480
69.5291
67.3842
67.1406
61.3150
58.3366
27.6804
27.3555
23.4236
20.7434
Si
O
O
O
BnO
N_3
O
BnO
O
BnO
SPh
N_3
14 *beta* SPh
f1 (ppm)

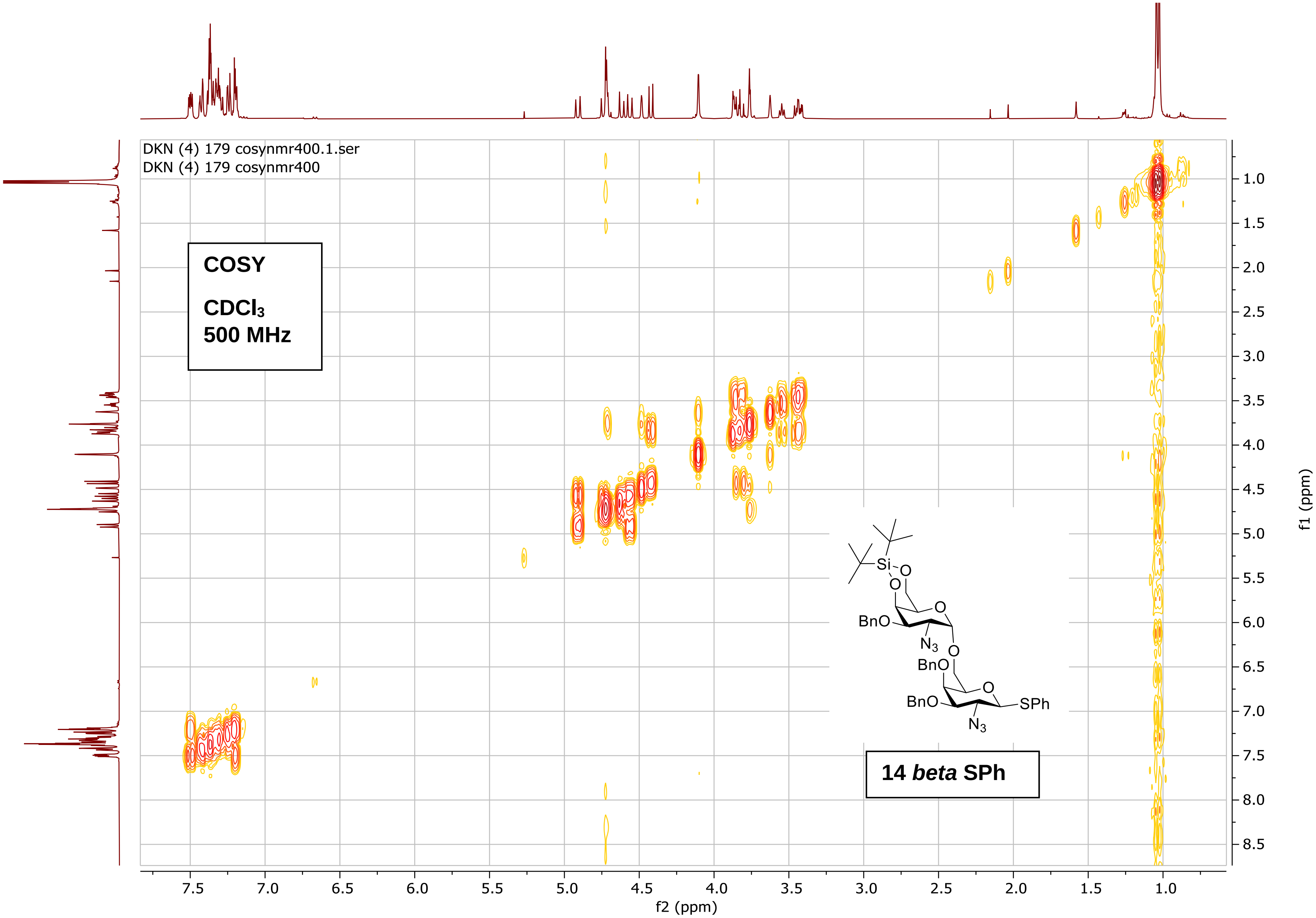
DKN (4) 179 cosynmr400.1.ser
DKN (4) 179 cosynmr400
COSY
CDCl3
500 MHz
14 beta SPh
BnO
N3
O
Si
SPh
f2 (ppm)
f1 (ppm)

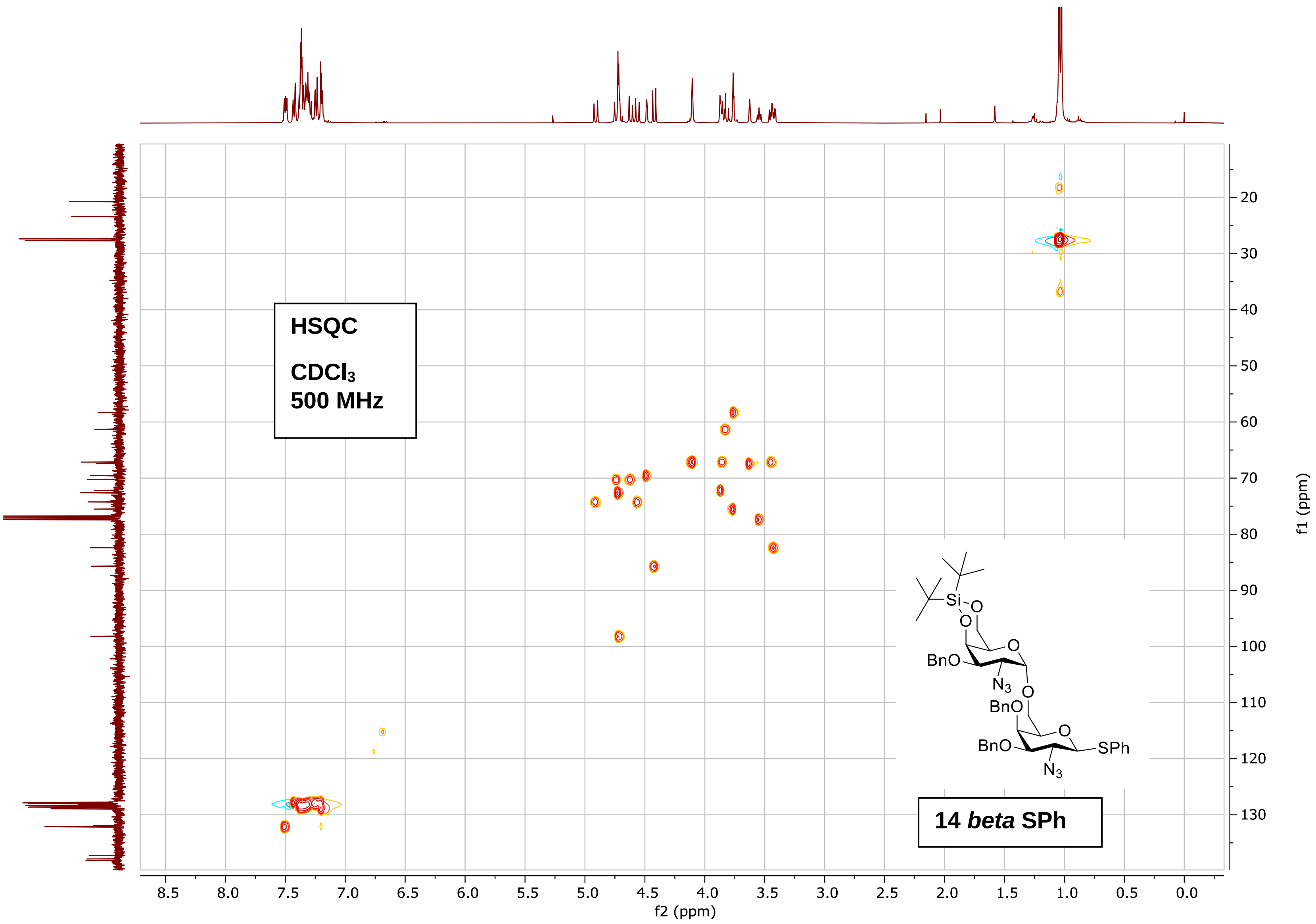
HSQC
CDCl3
500 MHz
14 beta SPh
f2 (ppm)
f1 (ppm)
BnO
N3
O
SPh
Si

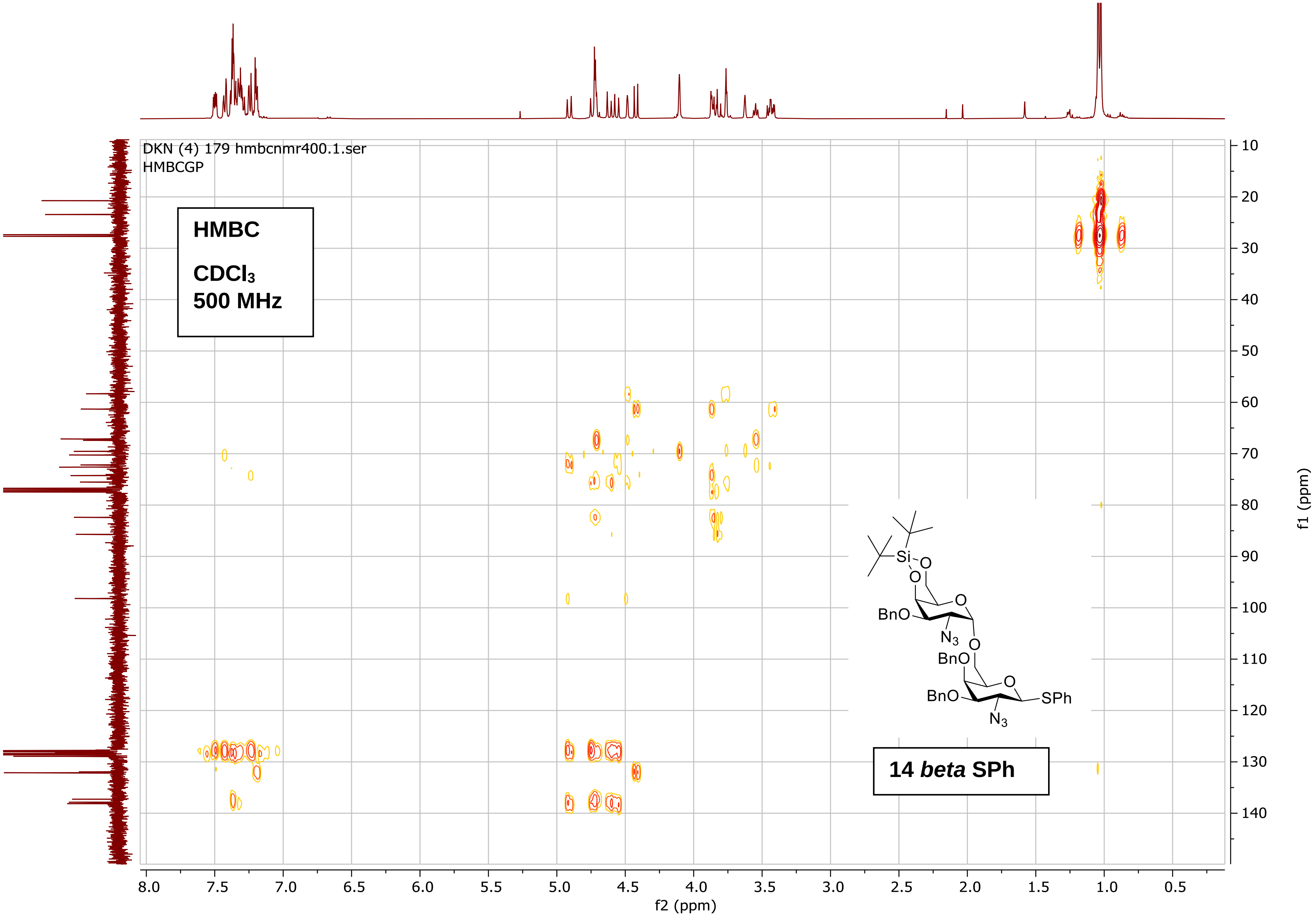
DKN (4) 179 hmbcnmr400.1.ser
HMBCGP
HMBC
CDCl3
500 MHz
14 beta SPh
f2 (ppm)
f1 (ppm)
Si
O
BnO
N3
SPh

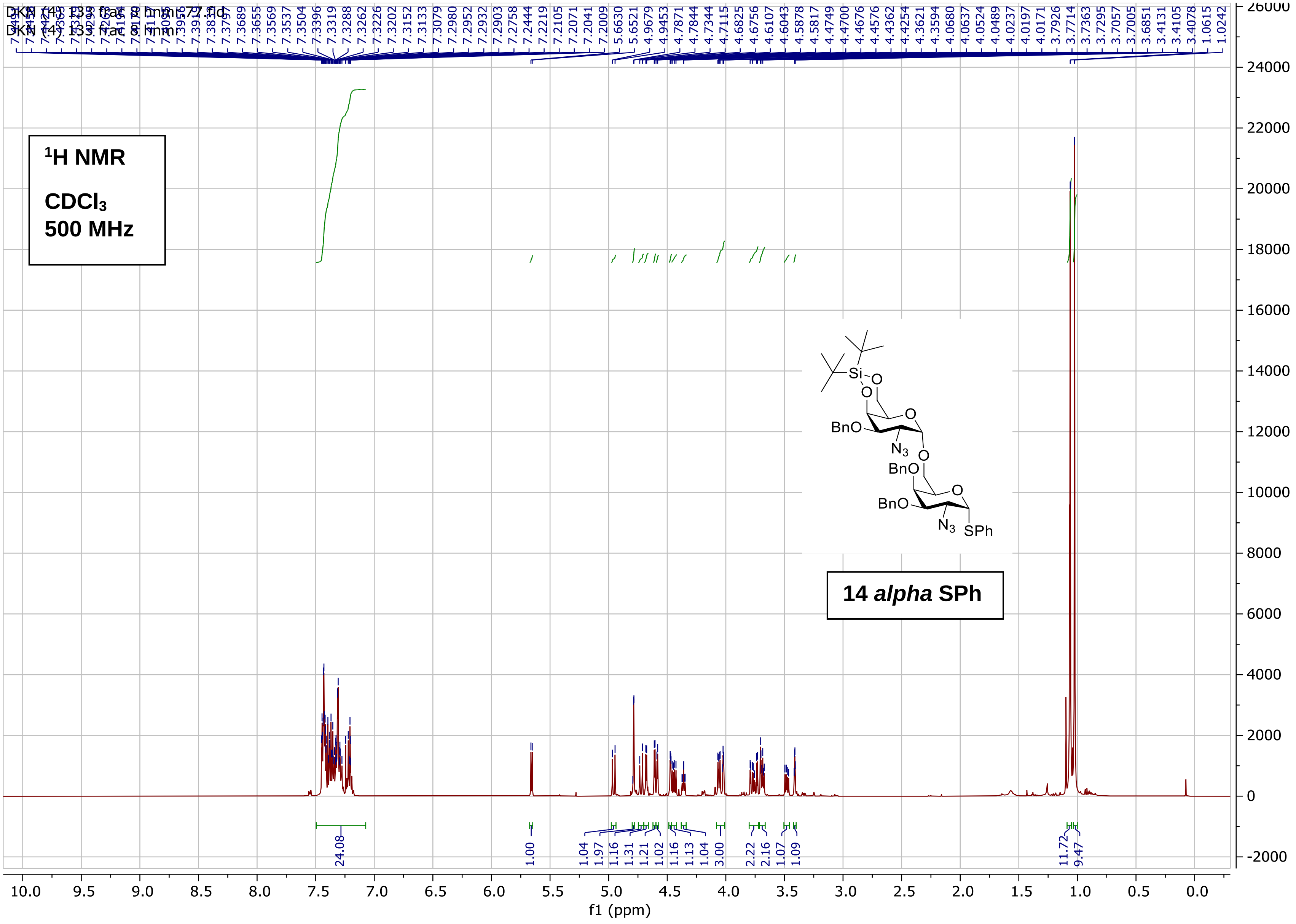

1H NMR
CDCl3
500 MHz
14 alpha SPh
BnO
N3
SPh
f1 (ppm)

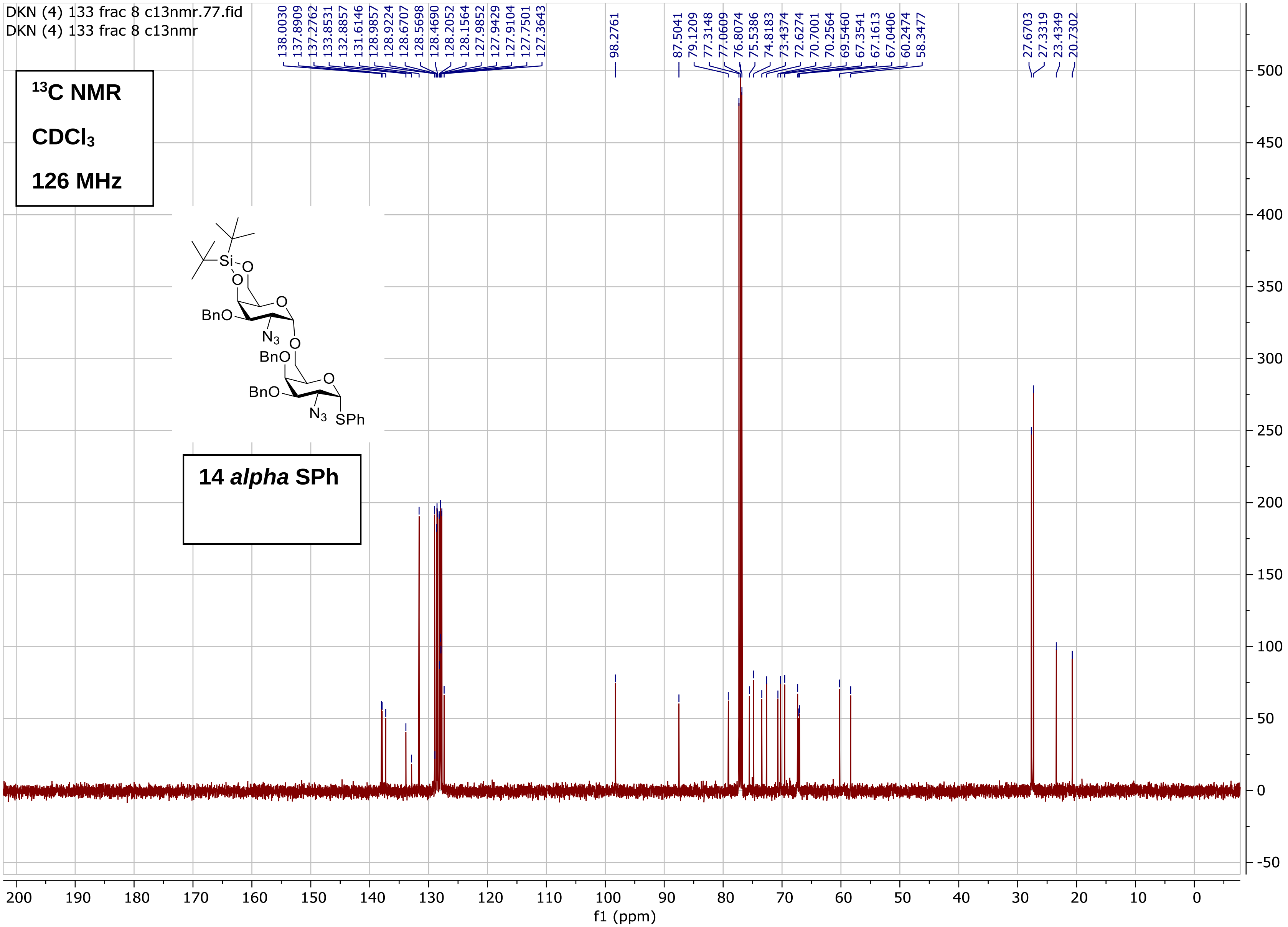
DKN (4) 133 frac 8 c13nmr.77.fid
DKN (4) 133 frac 8 c13nmr
13C NMR
CDCl3
126 MHz
14 alpha SPh
BnO
N3
SPh
138.0030
137.8909
137.2762
133.8531
132.8857
131.6146
128.9857
128.9224
128.6707
128.5698
128.4690
128.2052
128.1564
127.9852
127.9429
127.9104
127.7501
127.3643
98.2761
87.5041
79.1209
77.3148
77.0609
76.8074
75.5386
74.8183
73.4374
72.6274
70.7001
70.2564
69.5460
67.3541
67.1613
67.0406
60.2474
58.3477
27.6703
27.3319
23.4349
20.7302
f1 (ppm)

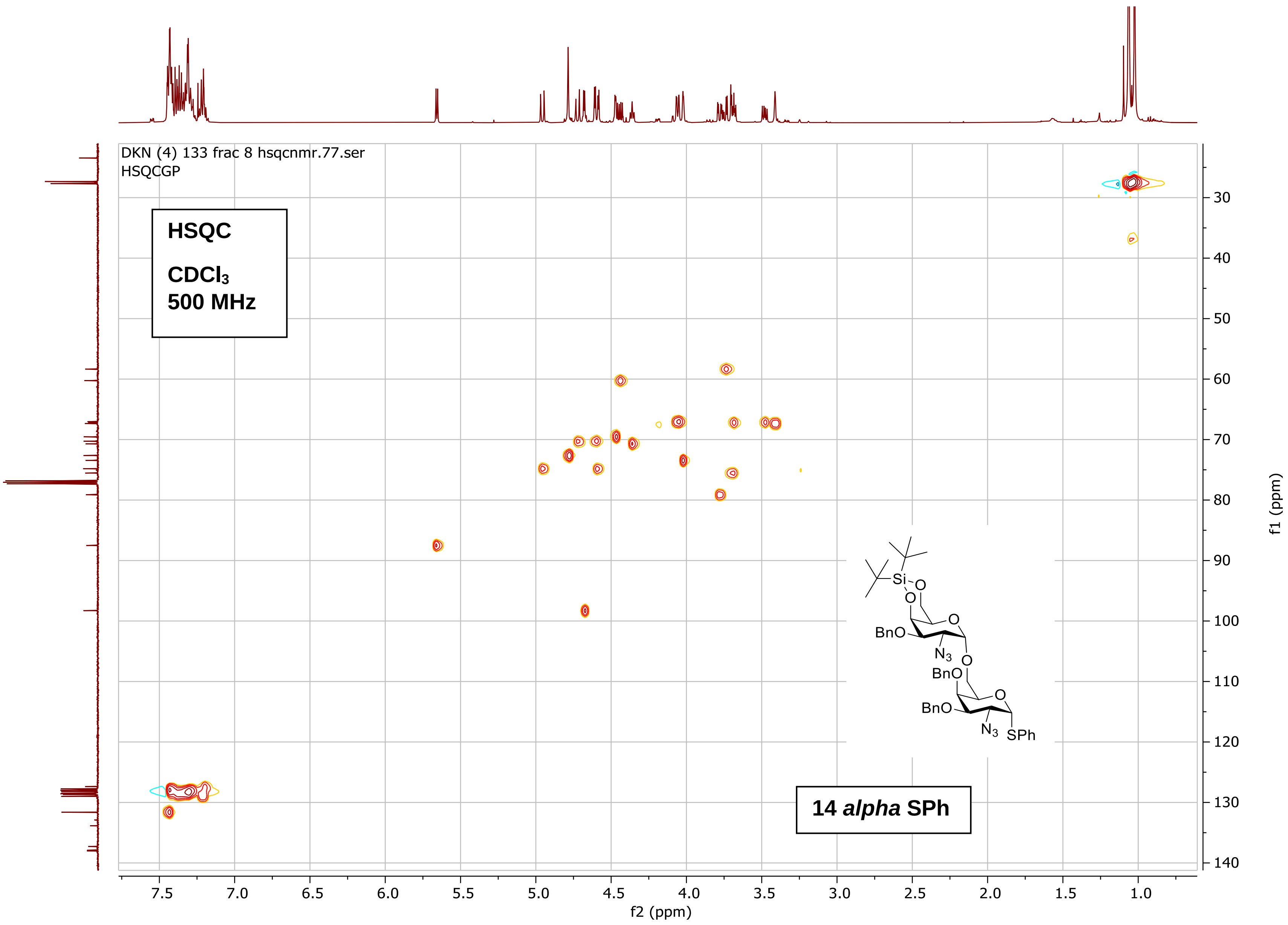
DKN (4) 133 frac 8 hsqcnmr.77.ser
HSQCGP
HSQC
CDCl3
500 MHz
14 alpha SPh
BnO
N3
BnO
BnO
N3
SPh
Si
f2 (ppm)
f1 (ppm)

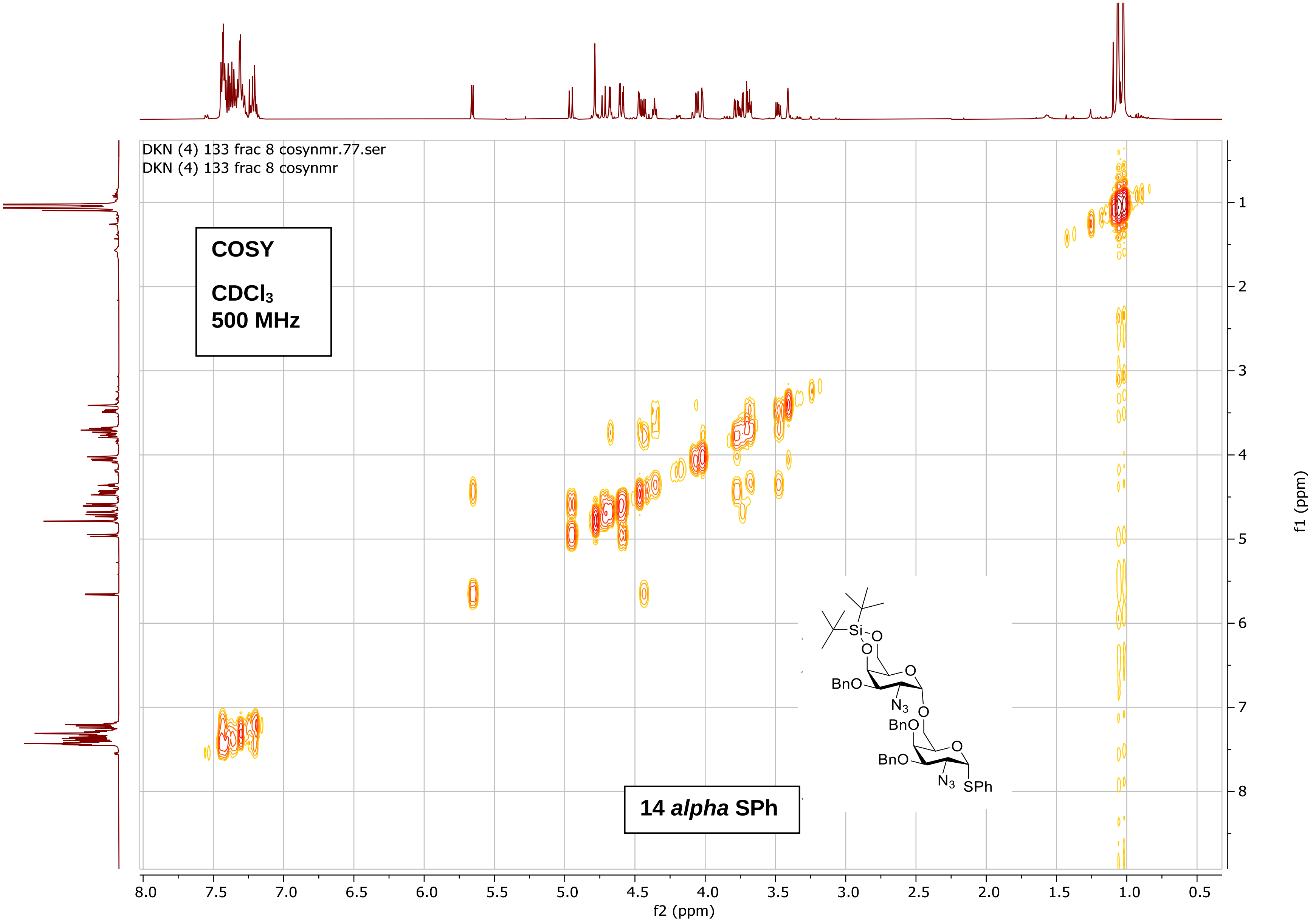

DKN (4) 133 frac 8 cosynmr.77.ser
DKN (4) 133 frac 8 cosynmr
COSY
CDCl3
500 MHz
14 alpha SPh
BnO
N3
O
BnO
BnO
SPh
Si
f2 (ppm)
f1 (ppm)
8.0
7.5
7.0
6.5
6.0
5.5
5.0
4.5
4.0
3.5
3.0
2.5
2.0
1.5
1.0
0.5
1
2
3
4
5
6
7
8

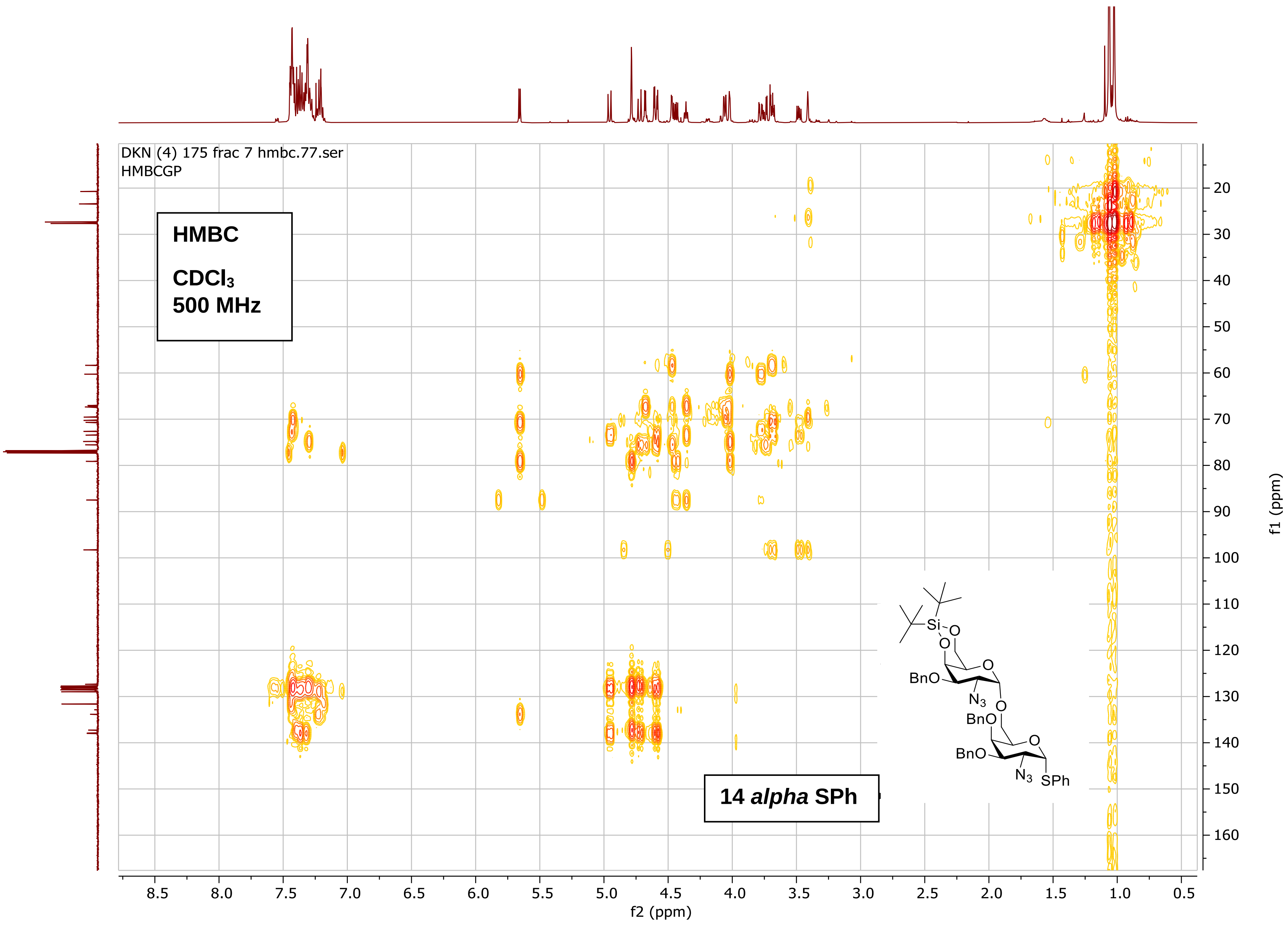

DKN (4) 175 frac 7 hmbc.77.ser
HMBCGP
HMBC
CDCl3
500 MHz
14 alpha SPh
f2 (ppm)
f1 (ppm)
Si
O
BnO
N3
BnO
BnO
SPh

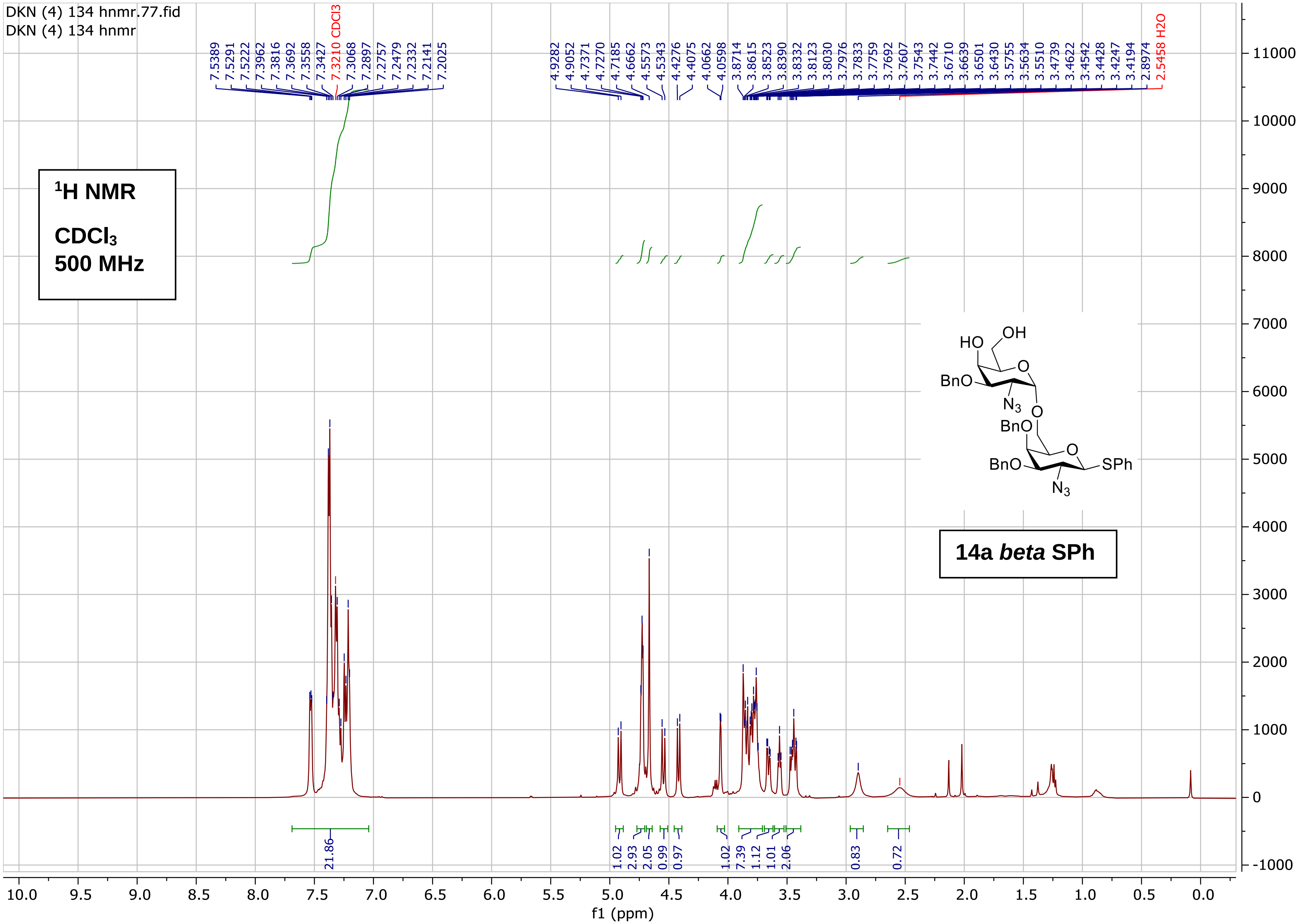

DKN (4) 134 hnmr.77.fid
DKN (4) 134 hnmr
7.5389 7.5291 7.5222 7.3962 7.3816 7.3692 7.3558 7.3427 7.3210 CDCl3 7.3068 7.2897 7.2757 7.2479 7.2332 7.2141 7.2025
4.9282 4.9052 4.7371 4.7270 4.7185 4.6662 4.5573 4.5343 4.4276 4.4075 4.0662 4.0598 3.8714 3.8615 3.8523 3.8390 3.8332 3.8123 3.8030 3.7976 3.7833 3.7759 3.7692 3.7607 3.7543 3.7442 3.6710 3.6639 3.6501 3.6430 3.5755 3.5634 3.5510 3.4739 3.4622 3.4542 3.4428 3.4247 3.4194 2.8974 2.5458 H2O
1H NMR
CDCl3
500 MHz
HO
OH
O
BnO
N3
O
BnO
O
BnO
SPh
N3
14a beta SPh
21.86
1.02 2.93 2.05 0.99 0.97 1.02 7.39 1.12 1.01 2.06 0.83 0.72
f1 (ppm)

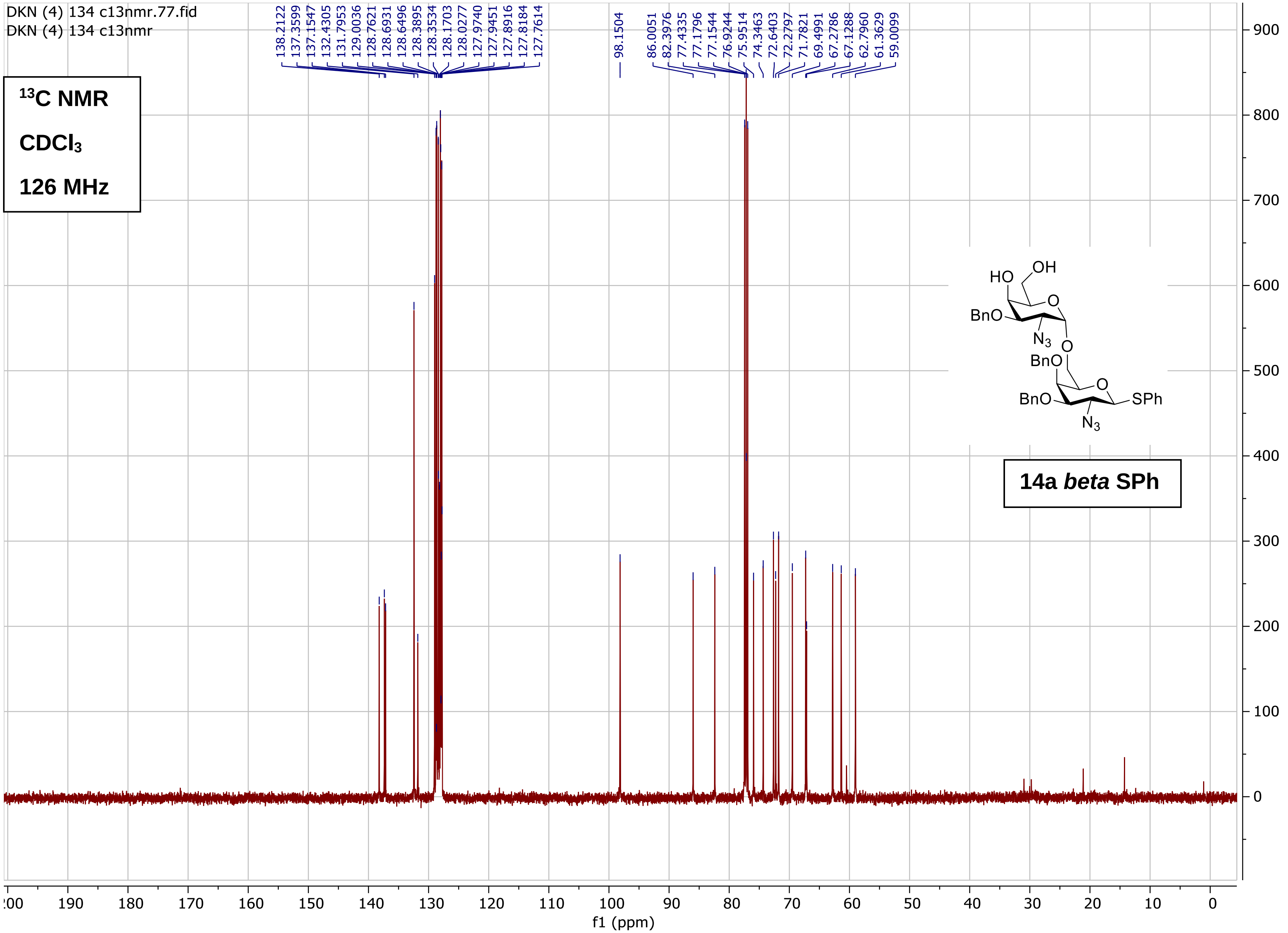
DKN (4) 134 c13nmr.77.fid
DKN (4) 134 c13nmr
13C NMR
CDCl3
126 MHz
138.2122
137.3599
137.1547
132.4305
131.7953
129.0036
128.7621
128.6931
128.6496
128.3895
128.3534
128.1703
128.0277
127.9740
127.9451
127.8916
127.8184
127.7614
98.1504
86.0051
82.3976
77.4335
77.1796
77.1544
76.9244
75.9514
74.3463
72.6403
72.2797
71.7821
69.4991
67.2786
67.1288
62.7960
61.3629
59.0099
HO
OH
O
BnO
N3
O
BnO
O
BnO
SPh
N3
14a beta SPh
f1 (ppm)

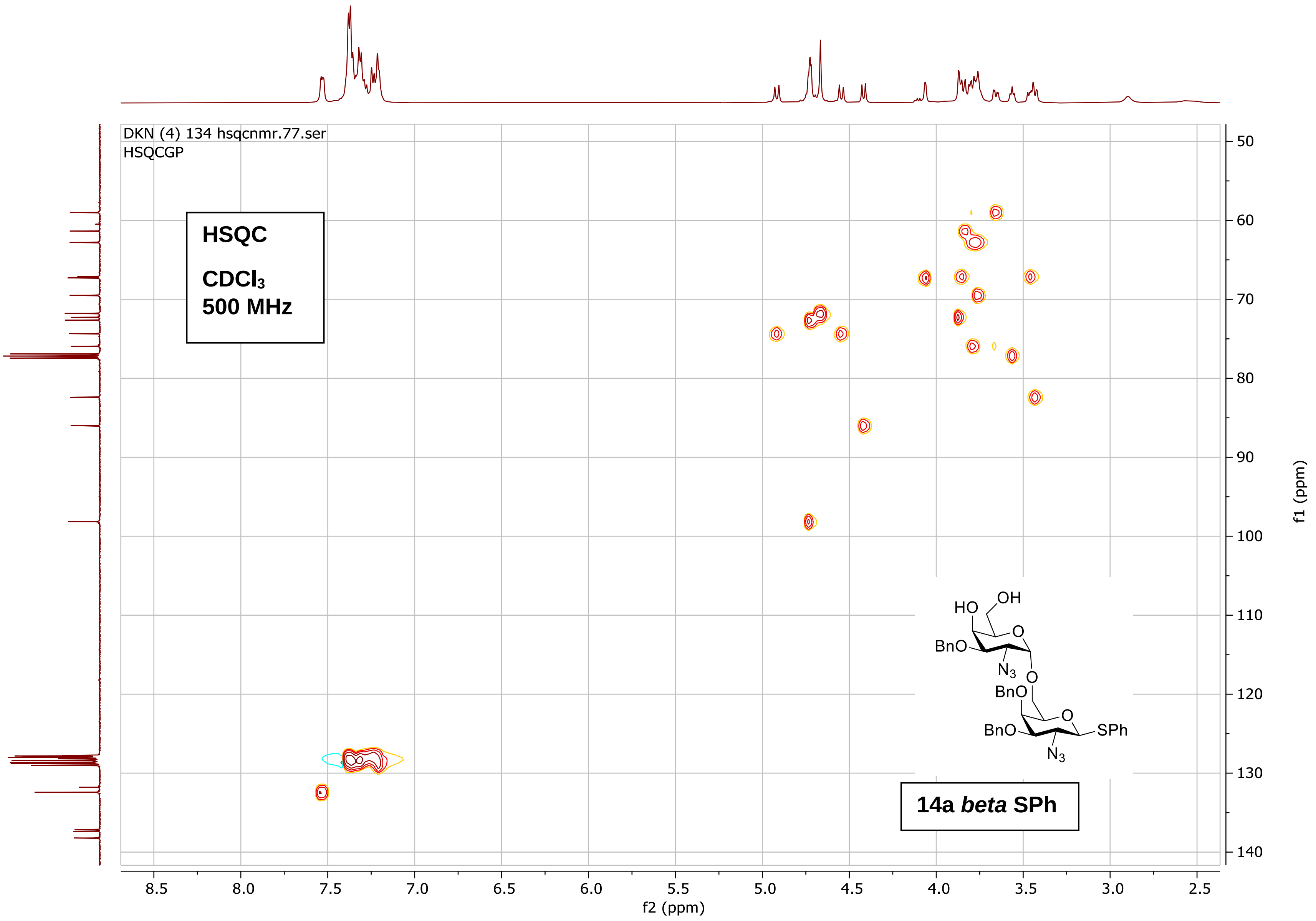
DKN (4) 134 hsqcnmr.77.ser
HSQCGP
HSQC
CDCl3
500 MHz
HO
OH
O
BnO
N3
O
BnO
O
BnO
SPh
N3
14a beta SPh
f2 (ppm)
f1 (ppm)

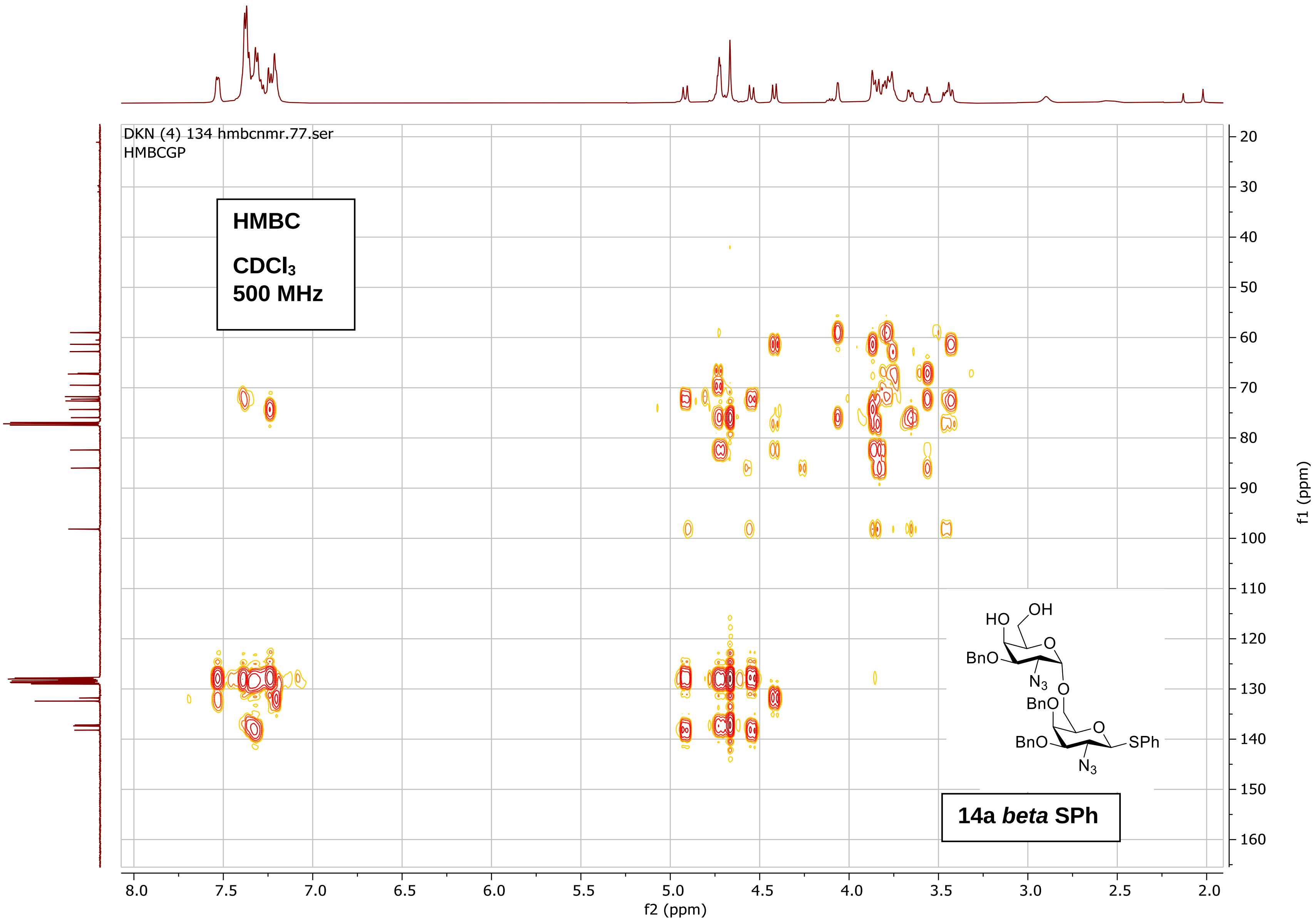

DKN (4) 134 hmbcnmr.77.ser
HMBCGP
HMBC
CDCl3
500 MHz
HO
OH
O
BnO
N3
O
BnO
O
BnO
SPh
N3
14a beta SPh
f1 (ppm)
f2 (ppm)

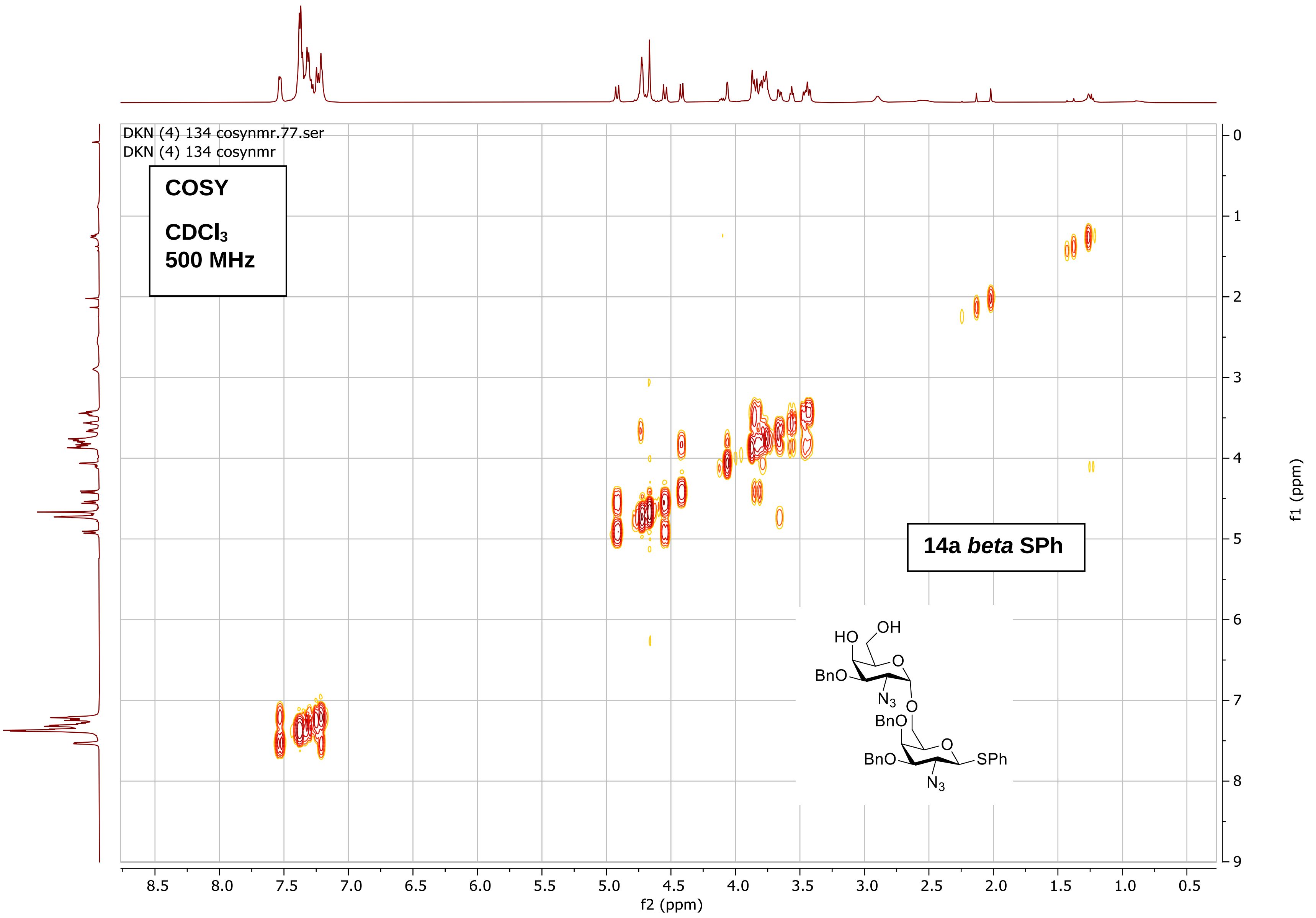

DKN (4) 134 cosynmr.77.ser
DKN (4) 134 cosynmr
COSY
CDCl3
500 MHz
14a beta SPh
HO
OH
O
BnO
N3
O
BnO
O
BnO
SPh
N3
f2 (ppm)
f1 (ppm)

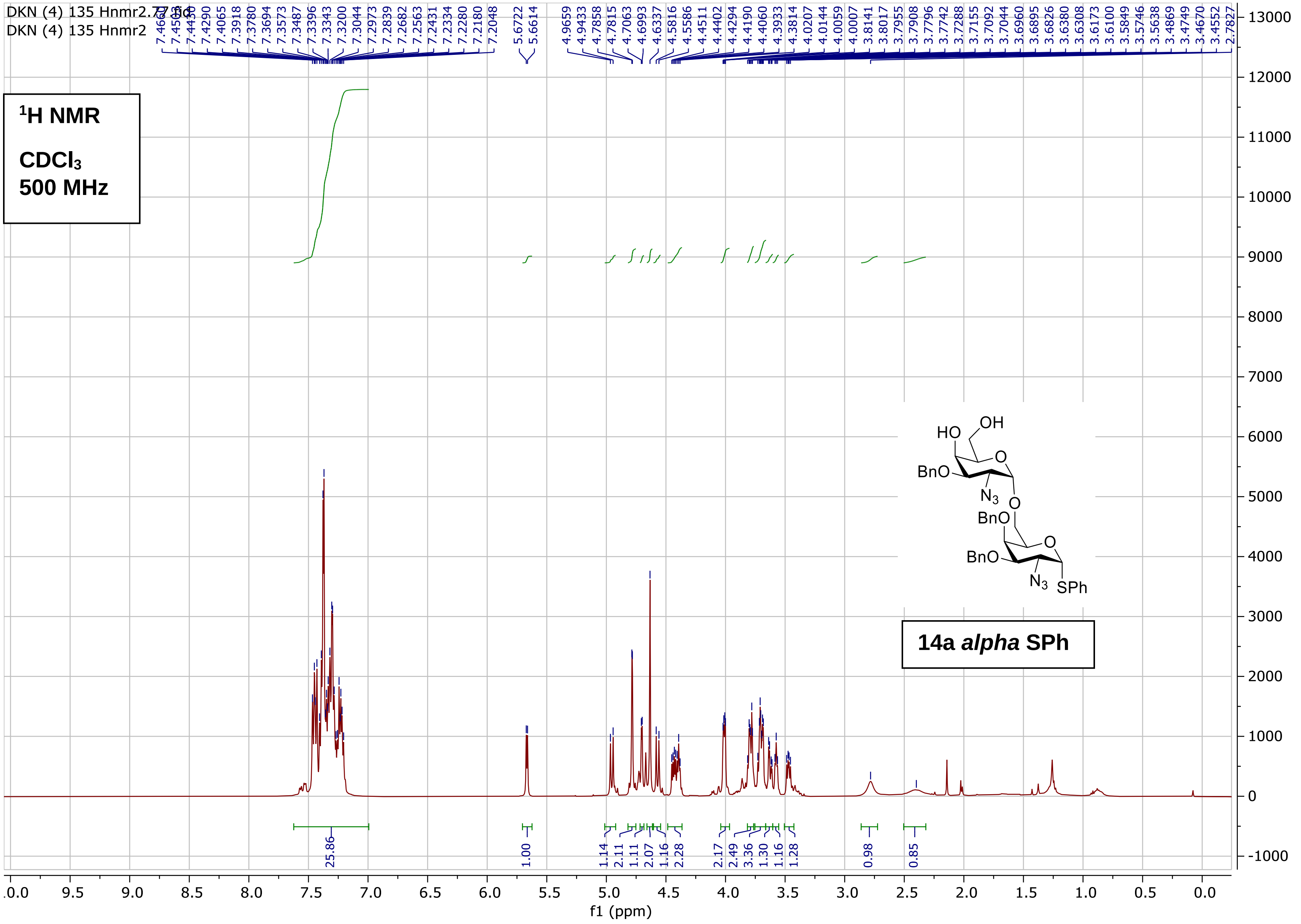

DKN (4) 135 Hnmr2.7.fid
DKN (4) 135 Hnmr2
^{1}H NMR
$CDCl_3$
500 MHz
HO
OH
O
BnO
N_3
O
BnO
O
BnO
N_3
SPh
14a *alpha* SPh
7.4661 7.4503 7.4430 7.4290 7.4065 7.3918 7.3780 7.3694 7.3573 7.3487 7.3396 7.3343 7.3200 7.3044 7.2973 7.2839 7.2682 7.2563 7.2431 7.2334 7.2280 7.2180 7.2048
5.6722 5.6614
4.9659 4.9433 4.7858 4.7815 4.7063 4.6993 4.6337 4.5816 4.5586 4.4511 4.4402 4.4294 4.4190 4.4060 4.3933 4.3814 4.0207 4.0144 4.0059 4.0007 3.8141 3.8017 3.7955 3.7908 3.7796 3.7742 3.7288 3.7155 3.7092 3.7044 3.6960 3.6895 3.6826 3.6380 3.6308 3.6173 3.6100 3.5849 3.5746 3.5638 3.4869 3.4749 3.4670 3.4552 2.7827
25.86 1.00 1.14 2.11 1.11 2.07 1.16 2.28 2.17 2.49 3.36 1.30 1.16 1.28 0.98 0.85
f1 (ppm)

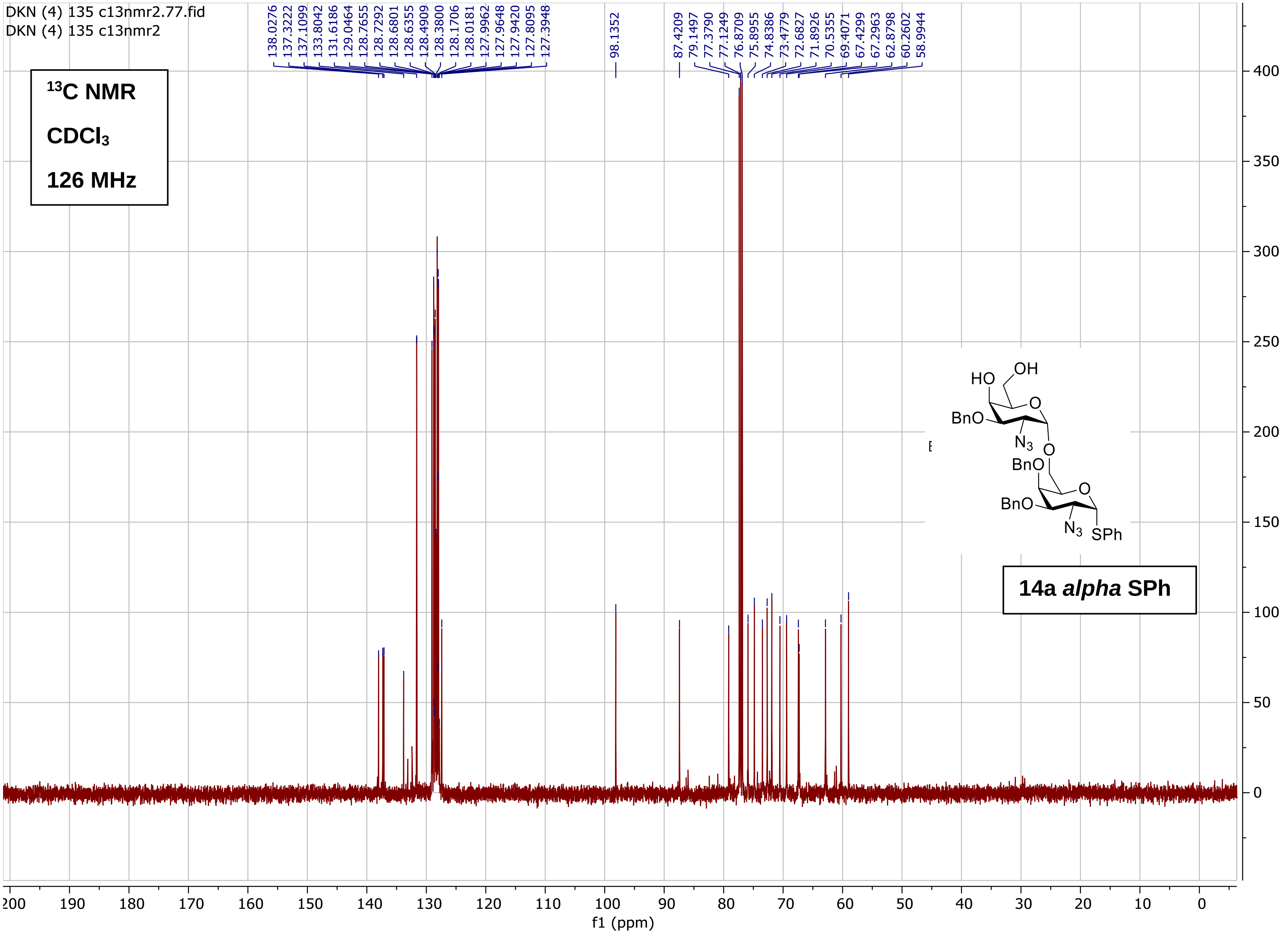
DKN (4) 135 c13nmr2.77.fid
DKN (4) 135 c13nmr2
^{13}C NMR
CDCl$_3$
126 MHz
138.0276
137.3222
137.1099
133.8042
131.6186
129.0464
128.7655
128.7292
128.6801
128.6355
128.4909
128.3800
128.1706
128.0181
127.9962
127.9648
127.9420
127.8095
127.3948
98.1352
87.4209
79.1497
77.3790
77.1249
76.8709
75.8955
74.8386
73.4779
72.6827
71.8926
70.5355
69.4071
67.4299
67.2963
62.8798
60.2602
58.9944
HO
OH
O
BnO
N$_3$
O
BnO
O
BnO
N$_3$
SPh
14a *alpha* SPh
f1 (ppm)

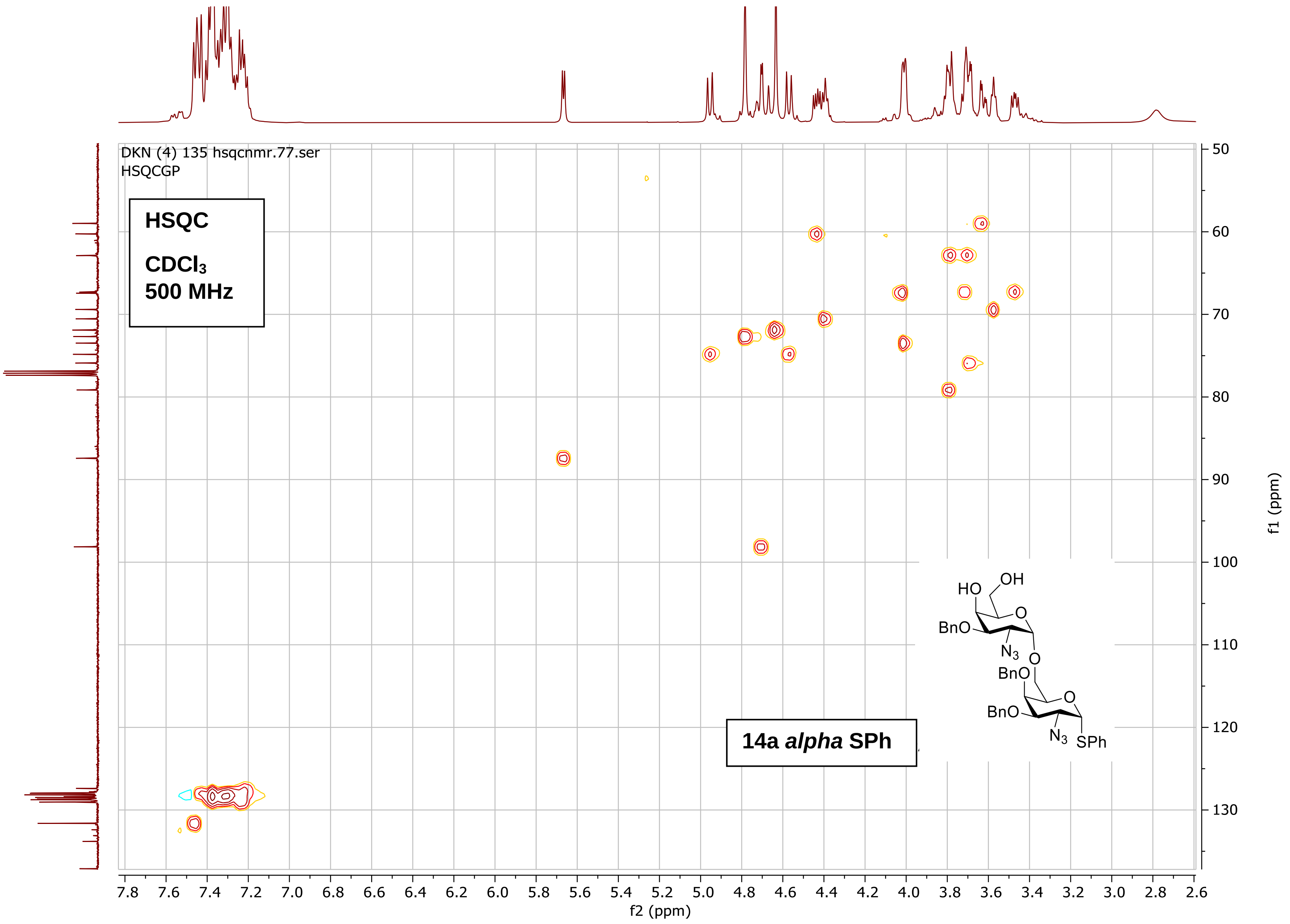
DKN (4) 135 hsqcnmr.77.ser
HSQCGP
HSQC
CDCl3
500 MHz
14a alpha SPh
HO
OH
O
BnO
N3
O
BnO
O
BnO
N3
SPh
f2 (ppm)
f1 (ppm)

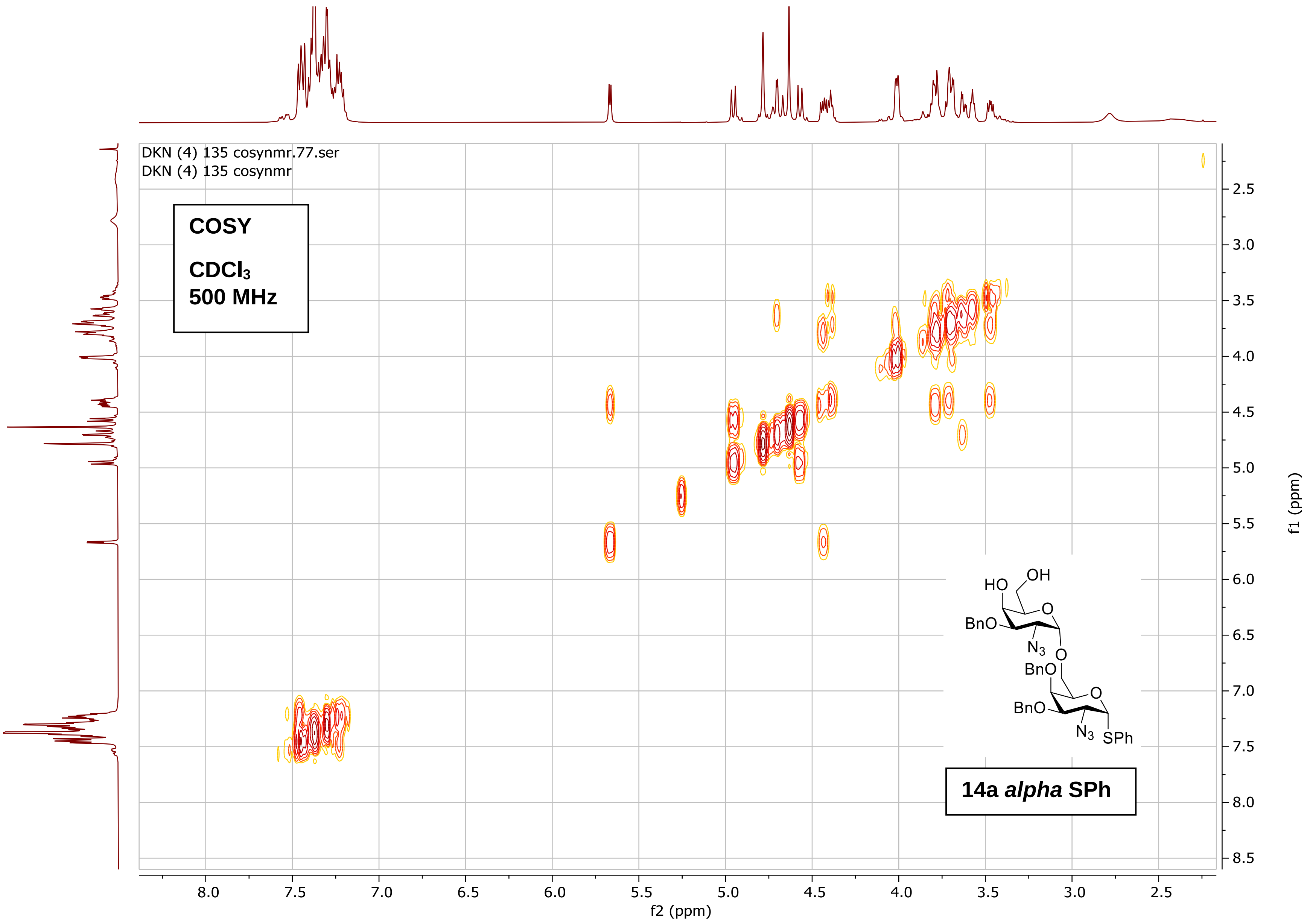
DKN (4) 135 cosynmr.77.ser
DKN (4) 135 cosynmr
COSY
CDCl3
500 MHz
HO
OH
O
BnO
N3
O
BnO
O
BnO
N3
SPh
14a alpha SPh
f2 (ppm)
f1 (ppm)

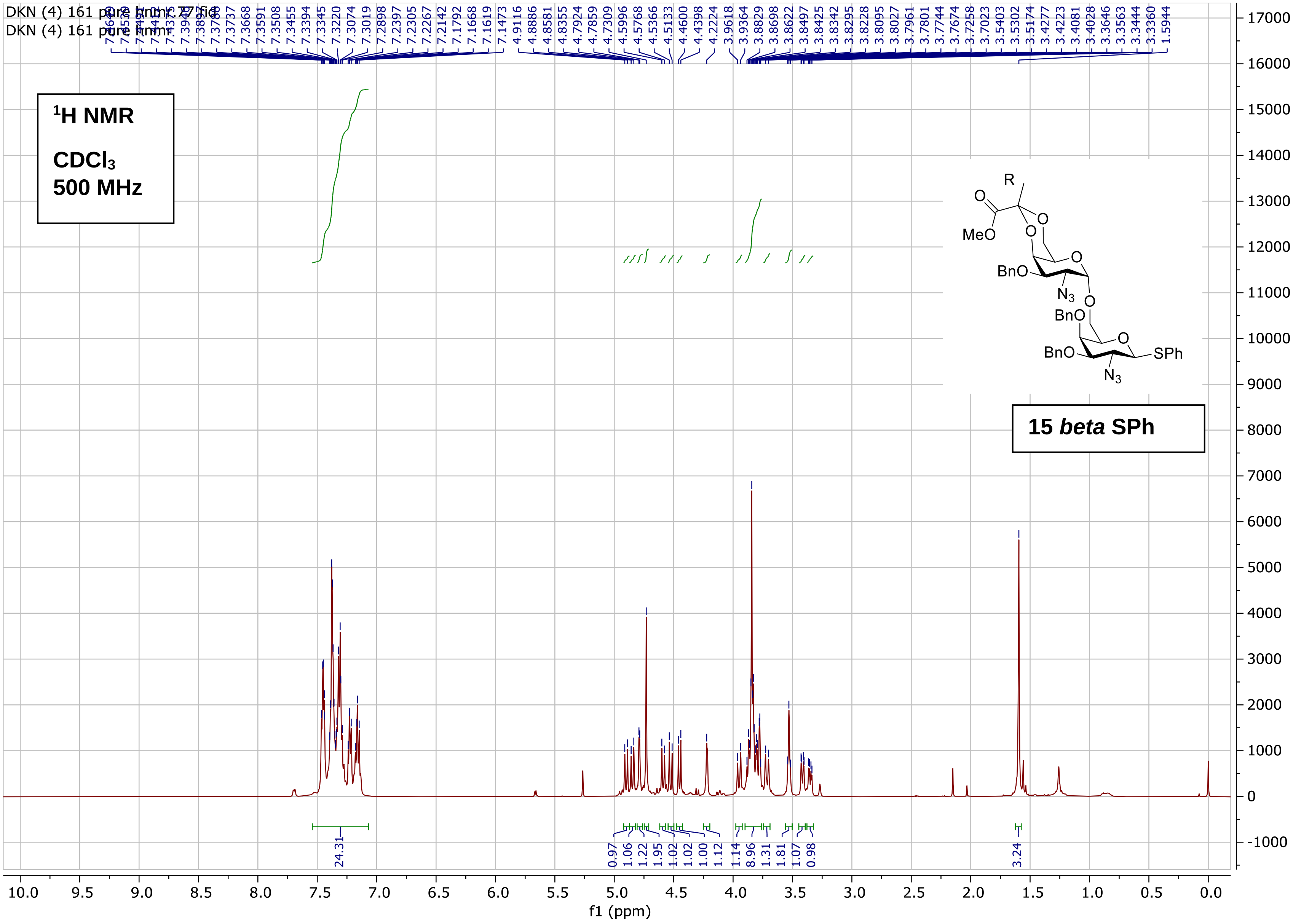

DKN (4) 161 pure 1h.1.fid
DKN (4) 161 pure hnmr
1H NMR
CDCl3
500 MHz
15 beta SPh
MeO
BnO
N3
SPh
R
f1 (ppm)

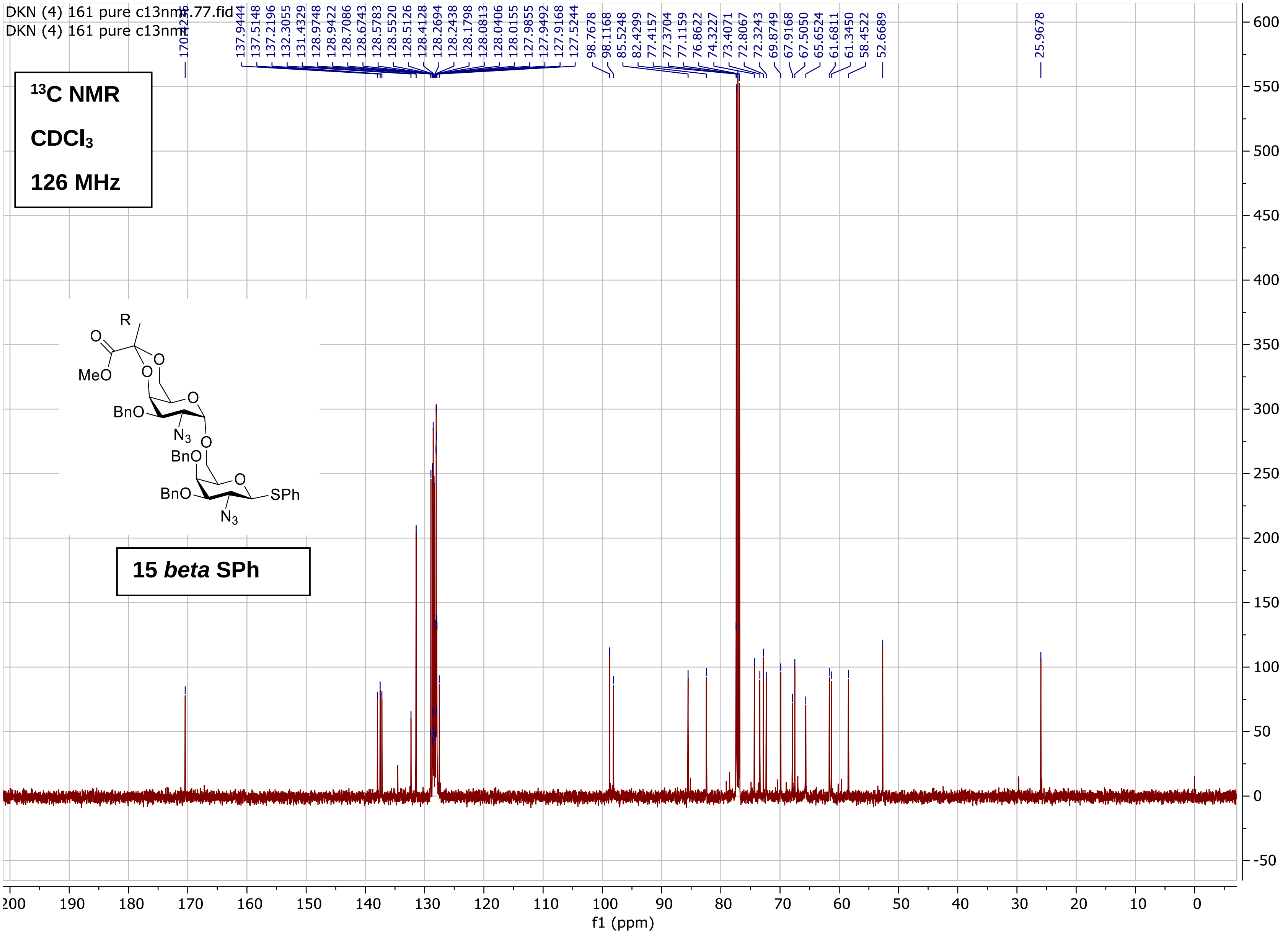
DKN (4) 161 pure c13nmr.77.fid
^{13}C NMR
CDCl$_3$
126 MHz
137.9444
137.5148
137.2196
132.3055
131.4329
128.9748
128.9422
128.7086
128.6743
128.5783
128.5520
128.5126
128.4128
128.2694
128.2438
128.1798
128.0813
128.0406
128.0155
127.9855
127.9492
127.9168
127.5244
98.7678
98.1168
85.5248
82.4299
77.4157
77.3704
77.1159
76.8622
74.3227
73.4071
72.8067
72.3243
69.8749
67.9168
67.5050
65.6524
61.6811
61.3450
58.4522
52.6689
25.9678
R
O
MeO
O
O
O
BnO
N$_3$
O
BnO
O
BnO
SPh
N$_3$
15 *beta* SPh
f1 (ppm)

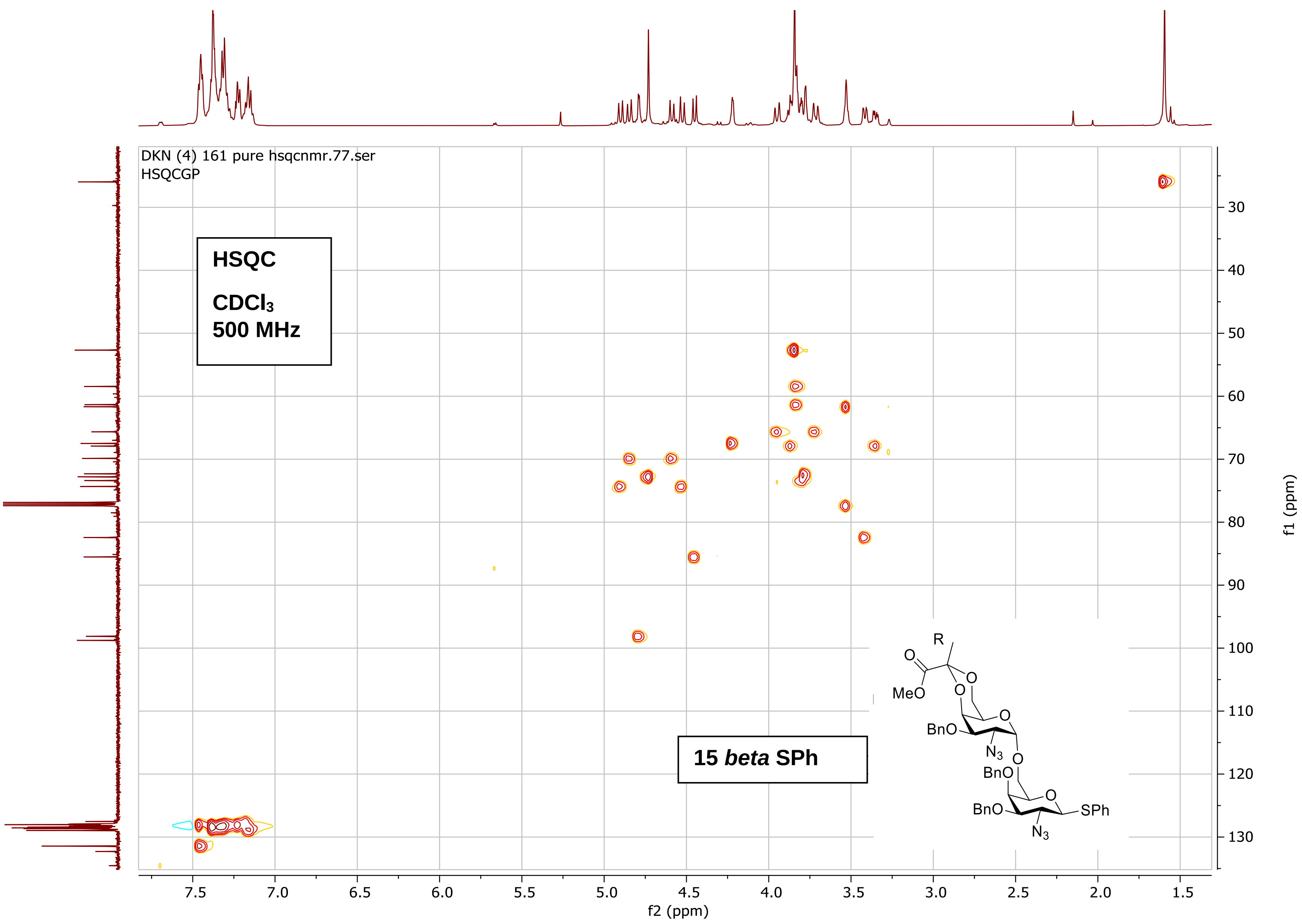

DKN (4) 161 pure hsqcnmr.77.ser
HSQCGP
HSQC
CDCl3
500 MHz
15 beta SPh
R
O
MeO
O
O
O
BnO
N3
O
BnO
O
BnO
SPh
N3
f2 (ppm)
f1 (ppm)

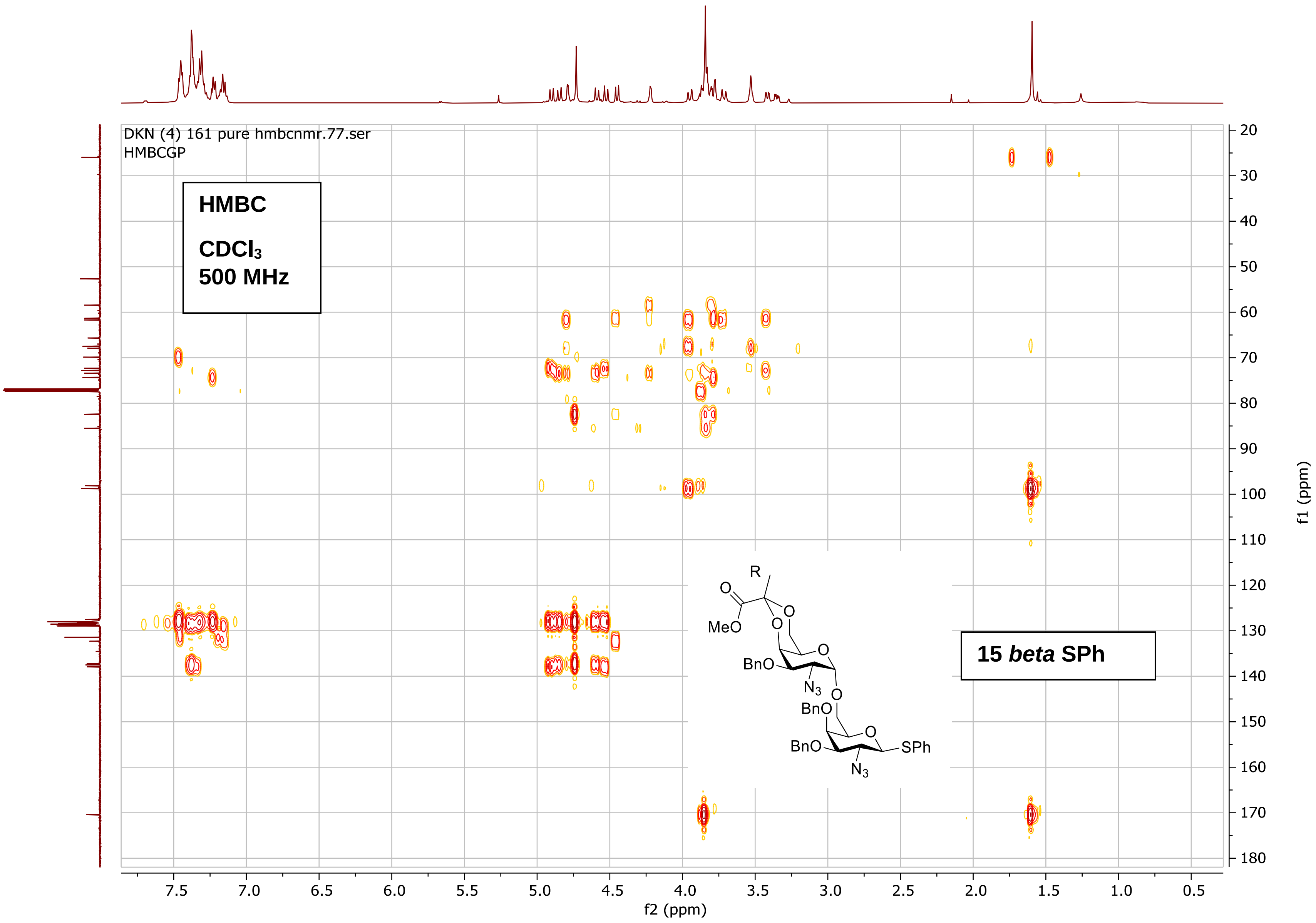

DKN (4) 161 pure hmbcnmr.77.ser
HMBCGP
HMBC
CDCl3
500 MHz
15 beta SPh
R
O
MeO
O
O
O
BnO
N3
O
BnO
O
BnO
SPh
N3
f2 (ppm)
f1 (ppm)

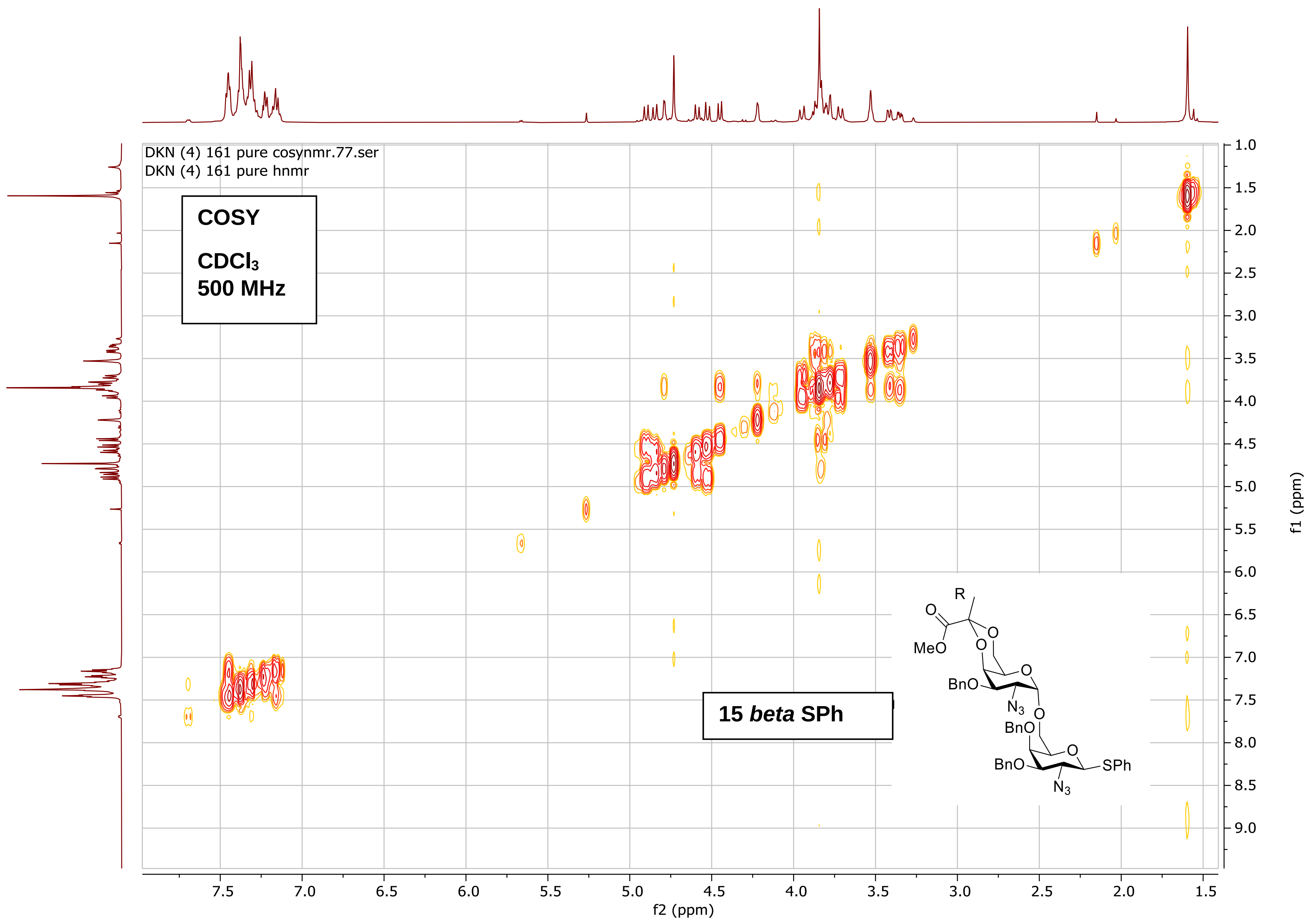
DKN (4) 161 pure cosynmr.77.ser
DKN (4) 161 pure hnmr
COSY
CDCl3
500 MHz
15 beta SPh
R
O
MeO
O
O
BnO
O
N3
O
BnO
BnO
O
SPh
N3
f2 (ppm)
f1 (ppm)

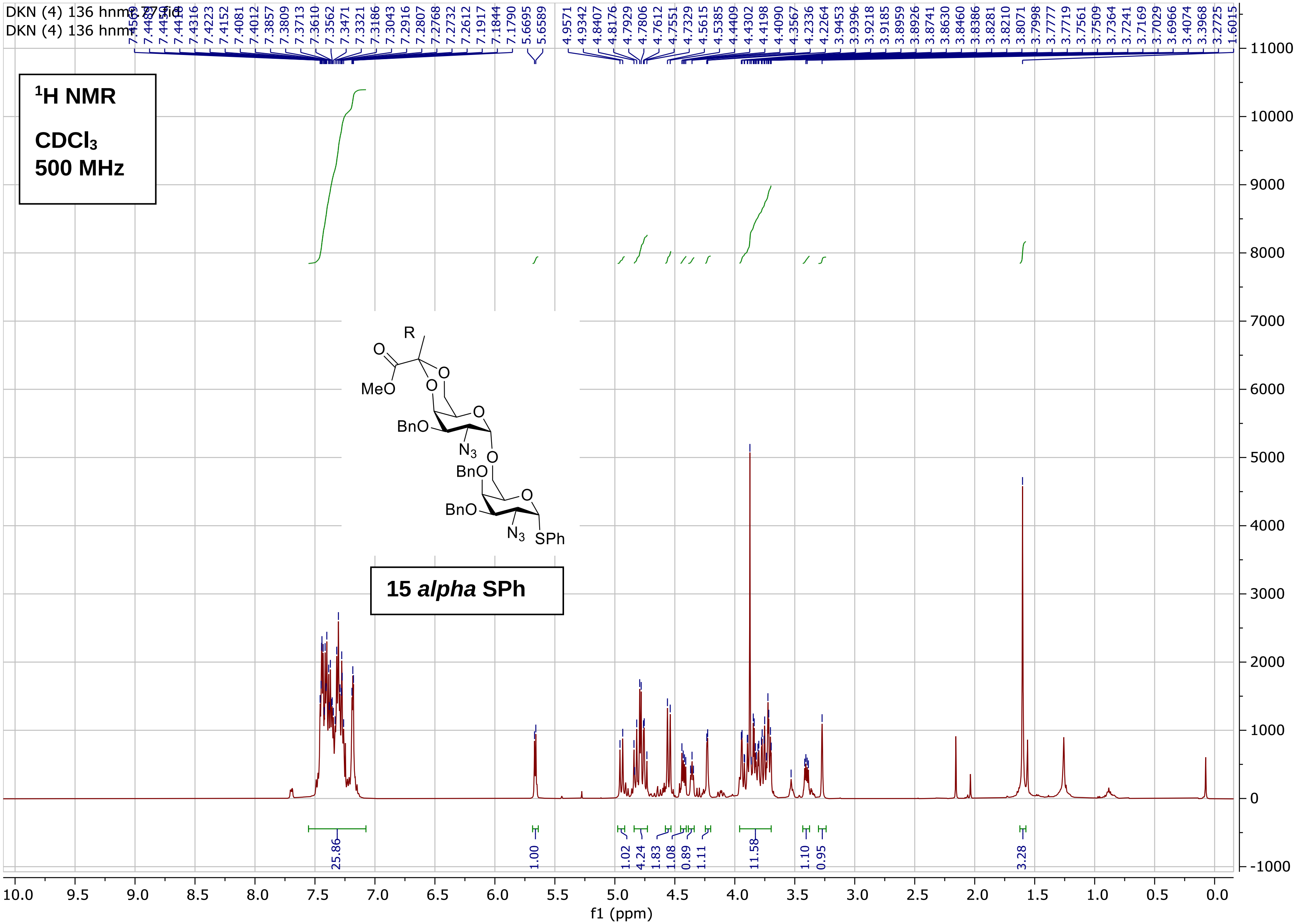

DKN (4) 136 hnmr.27.fid
DKN (4) 136 hnmr
¹H NMR
CDCl₃
500 MHz
15 alpha SPh
R
O
MeO
O
O
O
BnO
N₃
O
BnO
O
BnO
N₃
SPh
f1 (ppm)
25.86
1.00
1.02
4.24
1.83
1.08
0.89
1.11
11.58
1.10
0.95
3.28

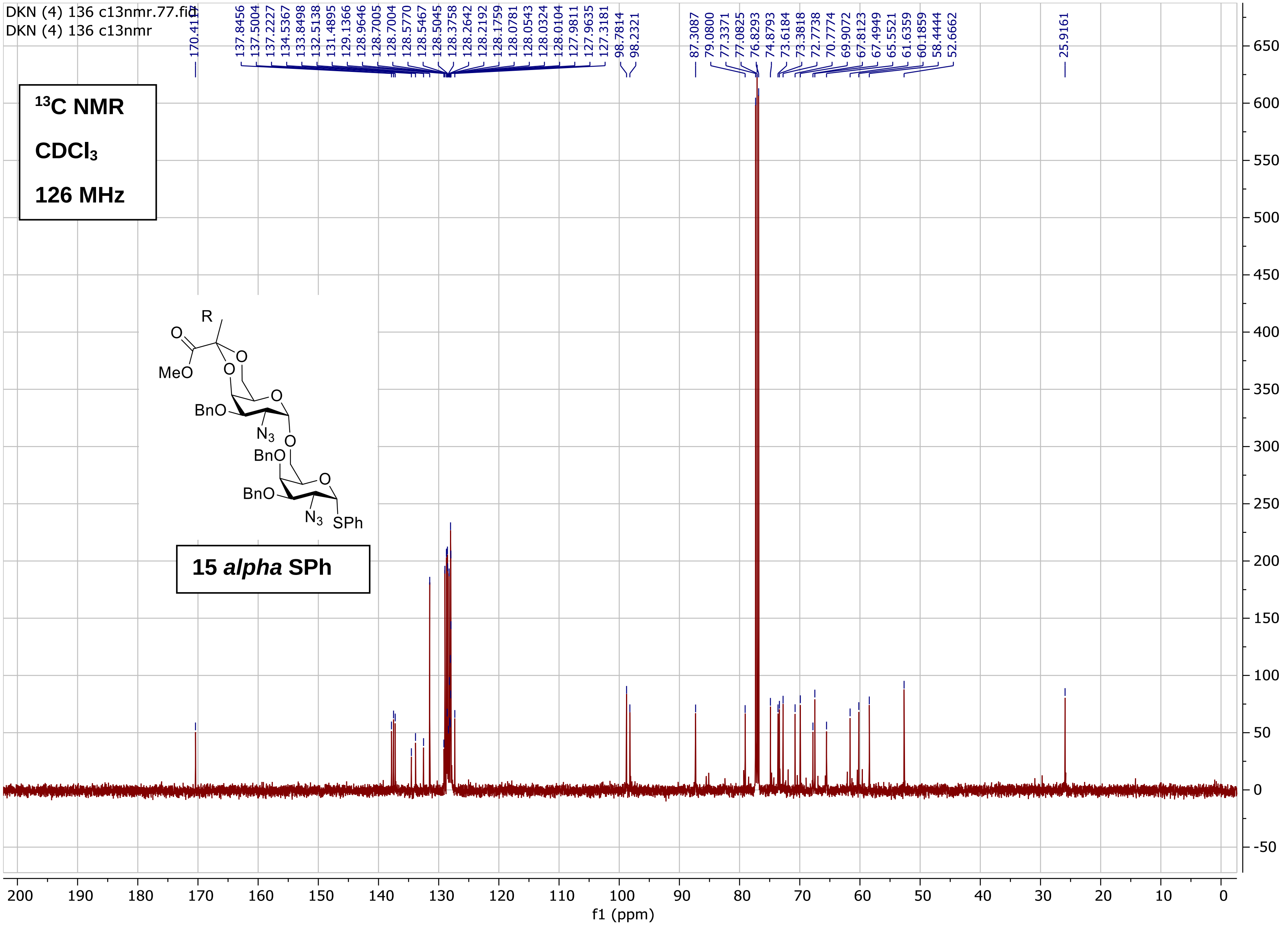
DKN (4) 136 c13nmr.77.fid
DKN (4) 136 c13nmr
170.4117
137.8456
137.5004
137.2227
134.5367
133.8498
132.5138
131.4895
129.1366
128.9646
128.7005
128.7004
128.5770
128.5467
128.5045
128.3758
128.2642
128.2192
128.1759
128.0781
128.0543
128.0324
128.0104
127.9811
127.9635
127.3181
98.7814
98.2321
87.3087
79.0800
77.3371
77.0825
76.8293
74.8793
73.6184
73.3818
72.7738
70.7774
69.9072
67.8123
67.4949
65.5521
61.6359
60.1859
58.4444
52.6662
25.9161
13C NMR
CDCl3
126 MHz
15 alpha SPh
R
O
MeO
BnO
N3
SPh
f1 (ppm)

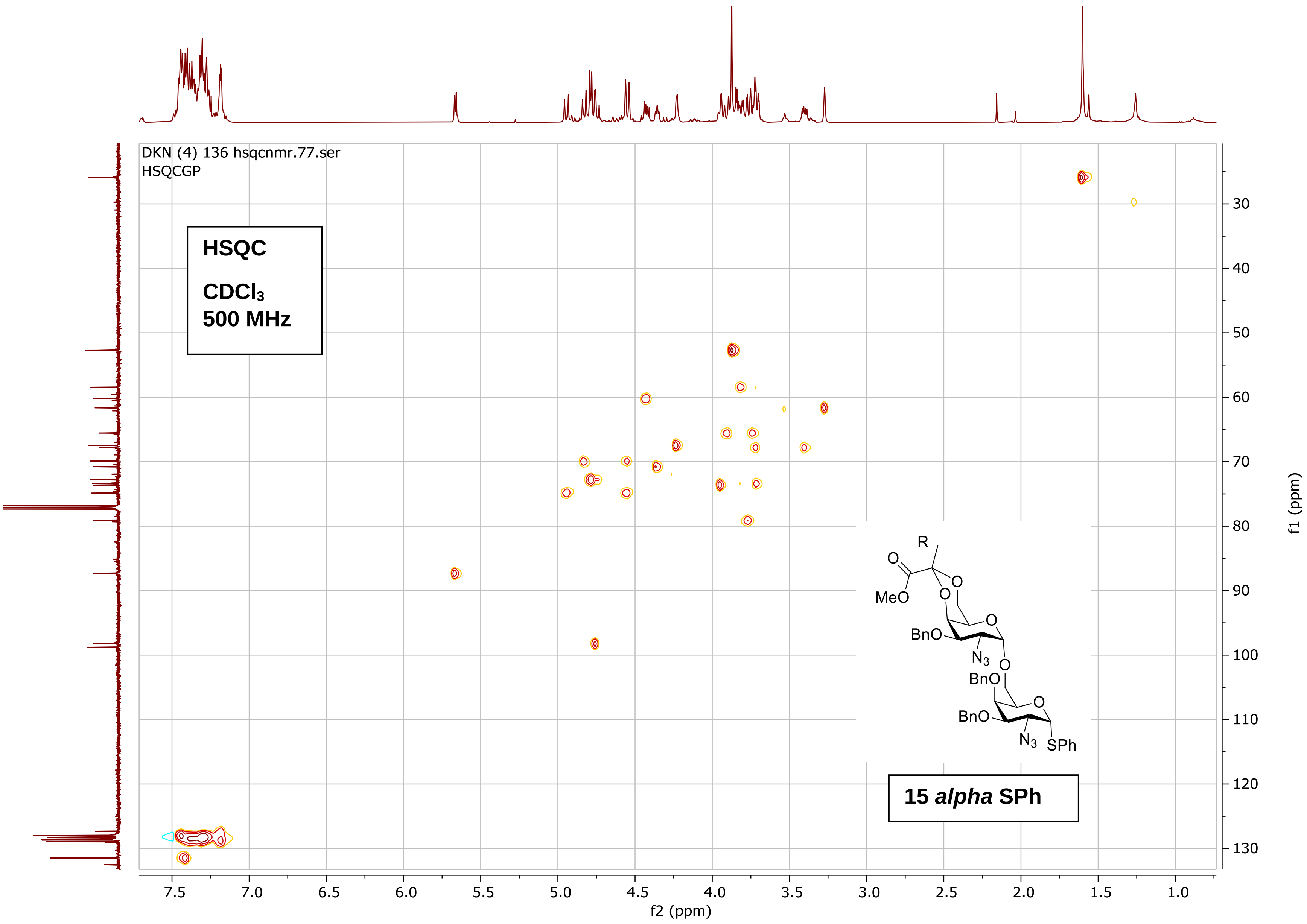

DKN (4) 136 hsqcnmr.77.ser
HSQCGP
HSQC
$CDCl_3$
500 MHz
R
O
O
O
MeO
O
BnO
N_3
O
BnO
O
BnO
N_3
SPh
15 *alpha* SPh
f2 (ppm)
f1 (ppm)

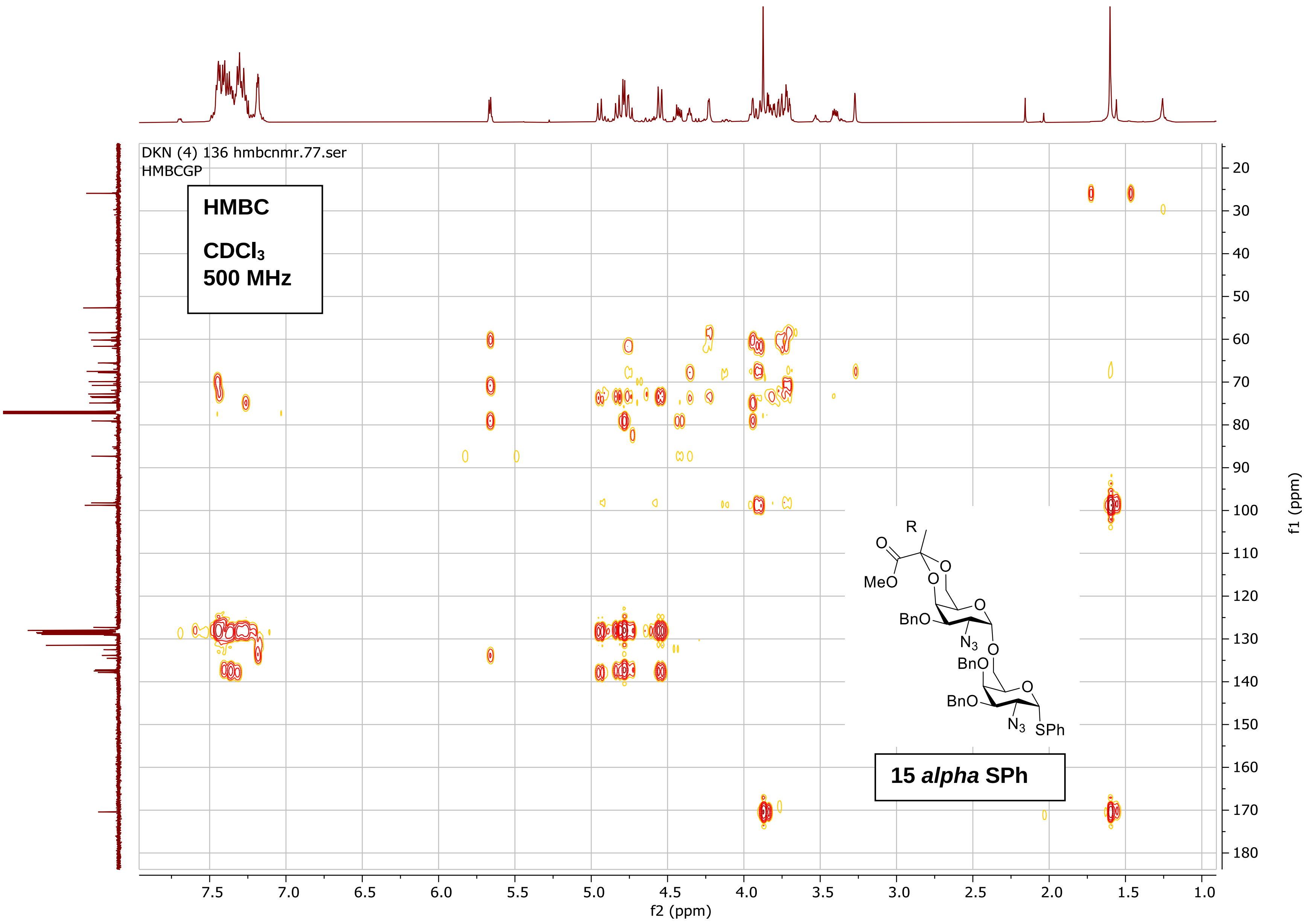
DKN (4) 136 hmbcnmr.77.ser
HMBCGP
HMBC
CDCl3
500 MHz
R
O
MeO
O
O
O
BnO
N3
O
BnO
O
BnO
N3
SPh
15 alpha SPh
f2 (ppm)
f1 (ppm)

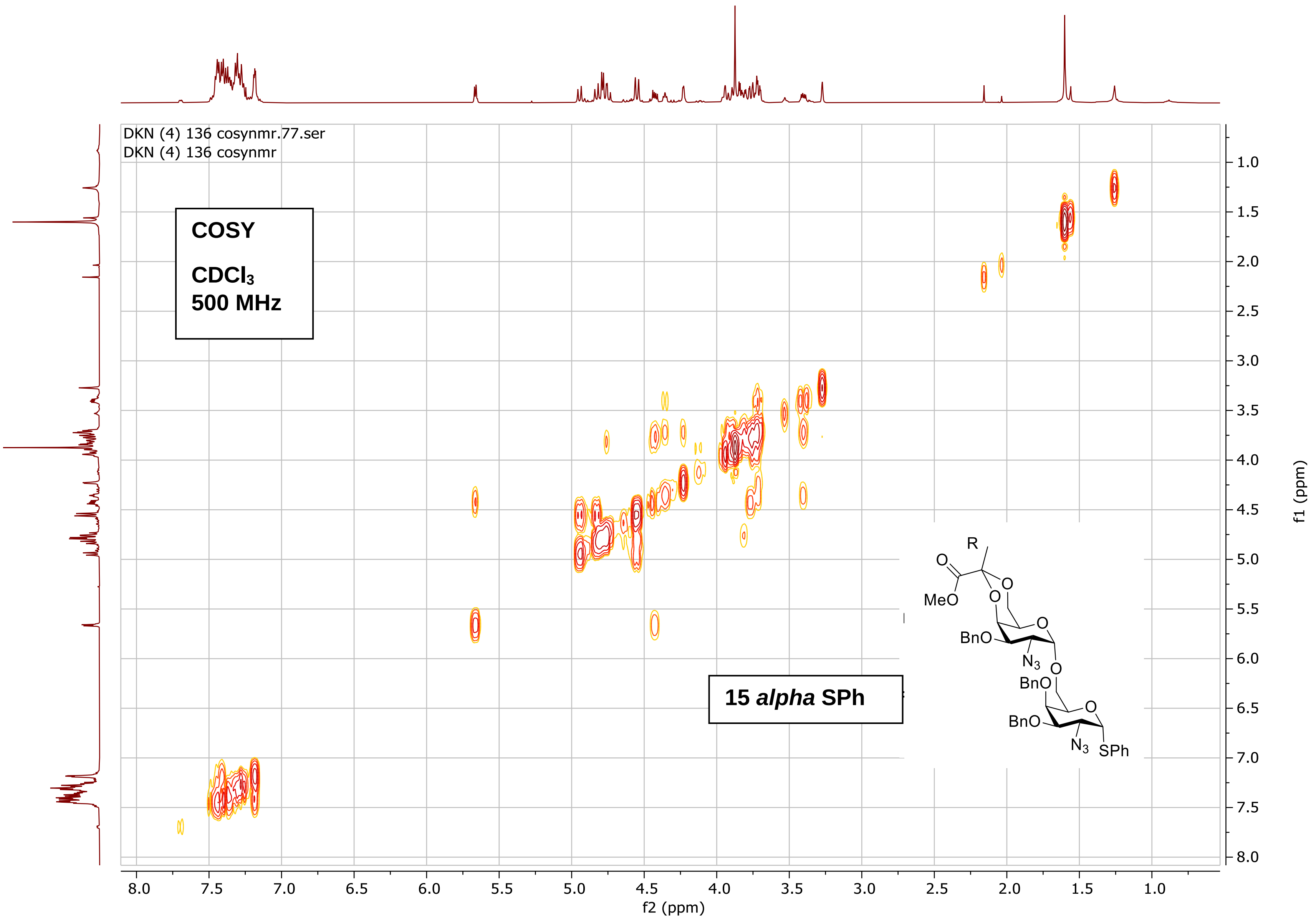
DKN (4) 136 cosynmr.77.ser
DKN (4) 136 cosynmr
COSY
CDCl3
500 MHz
15 alpha SPh
R
O
MeO
O
O
O
BnO
N3
O
BnO
O
BnO
N3
SPh
f2 (ppm)
f1 (ppm)

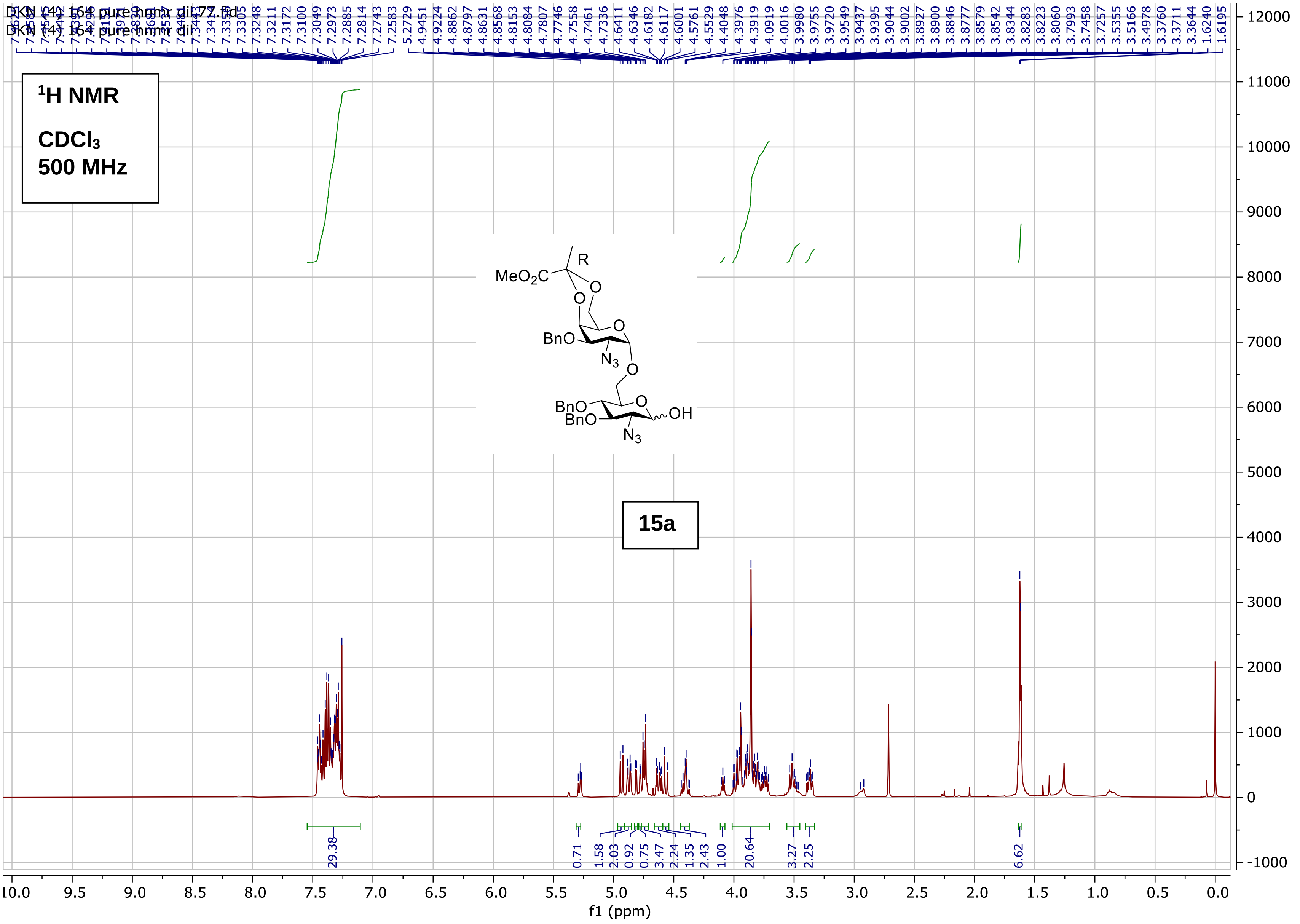

1H NMR
CDCl3
500 MHz
MeO2C
R
O
O
BnO
O
N3
O
BnO
BnO
O
OH
N3
15a
f1 (ppm)

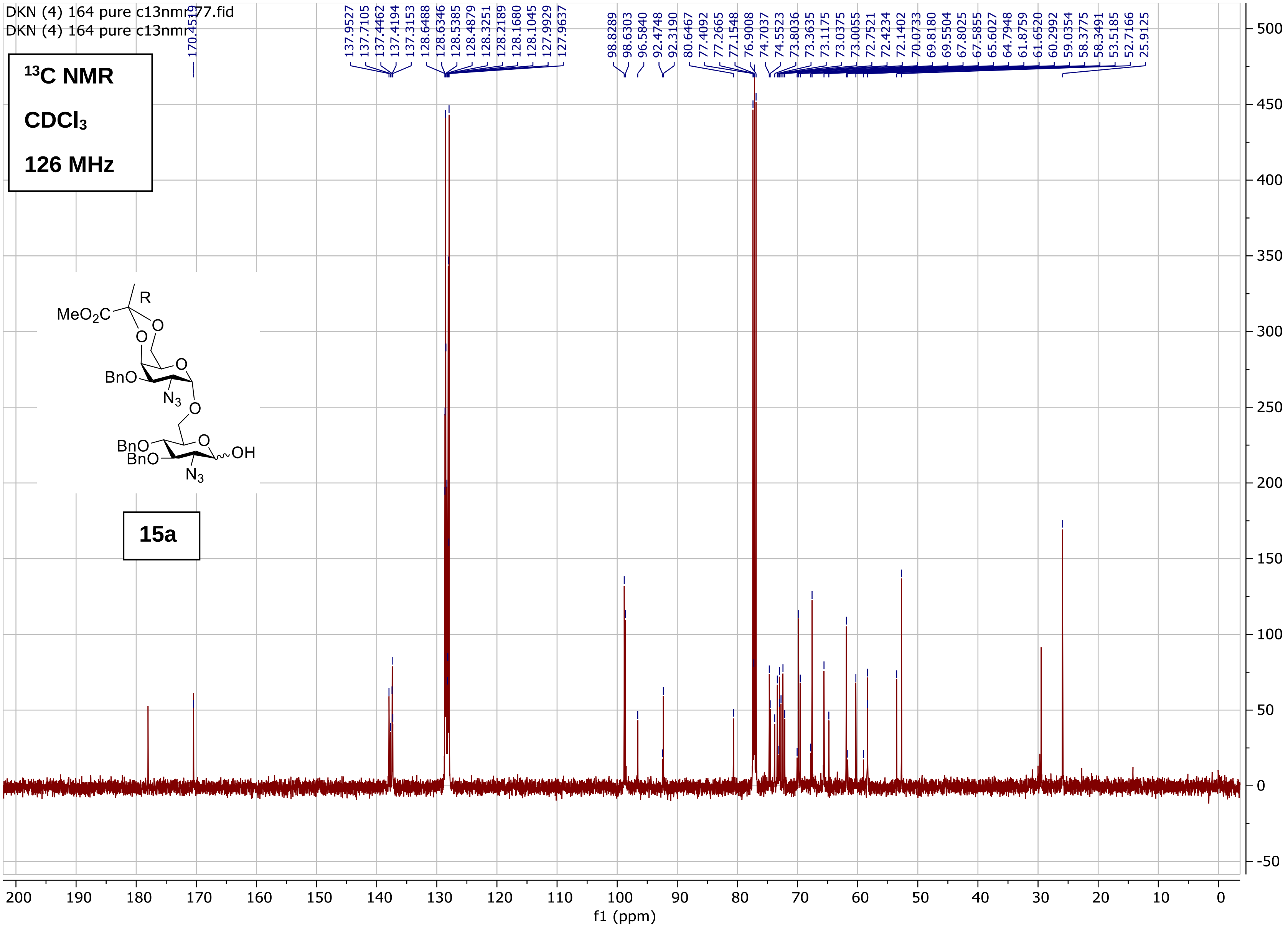
DKN (4) 164 pure c13nmr77.fid
DKN (4) 164 pure c13nmr
^{13}C NMR
CDCl$_3$
126 MHz
170.4519
137.9527
137.7105
137.4462
137.4194
137.3153
128.6488
128.6346
128.5385
128.4879
128.3251
128.2189
128.1680
128.1045
127.9929
127.9637
98.8289
98.6303
96.5840
92.4748
92.3190
80.6467
77.4092
77.2665
77.1548
76.9008
74.7037
74.5523
73.8036
73.3635
73.1175
73.0375
73.0055
72.7521
72.4234
72.1402
70.0733
69.8180
69.5504
67.8025
67.5855
65.6027
64.7948
61.8759
61.6520
60.2992
59.0354
58.3775
58.3491
53.5185
52.7166
25.9125
MeO$_2$C
R
O
O
BnO
O
N$_3$
O
BnO
BnO
O
OH
N$_3$
15a
f1 (ppm)

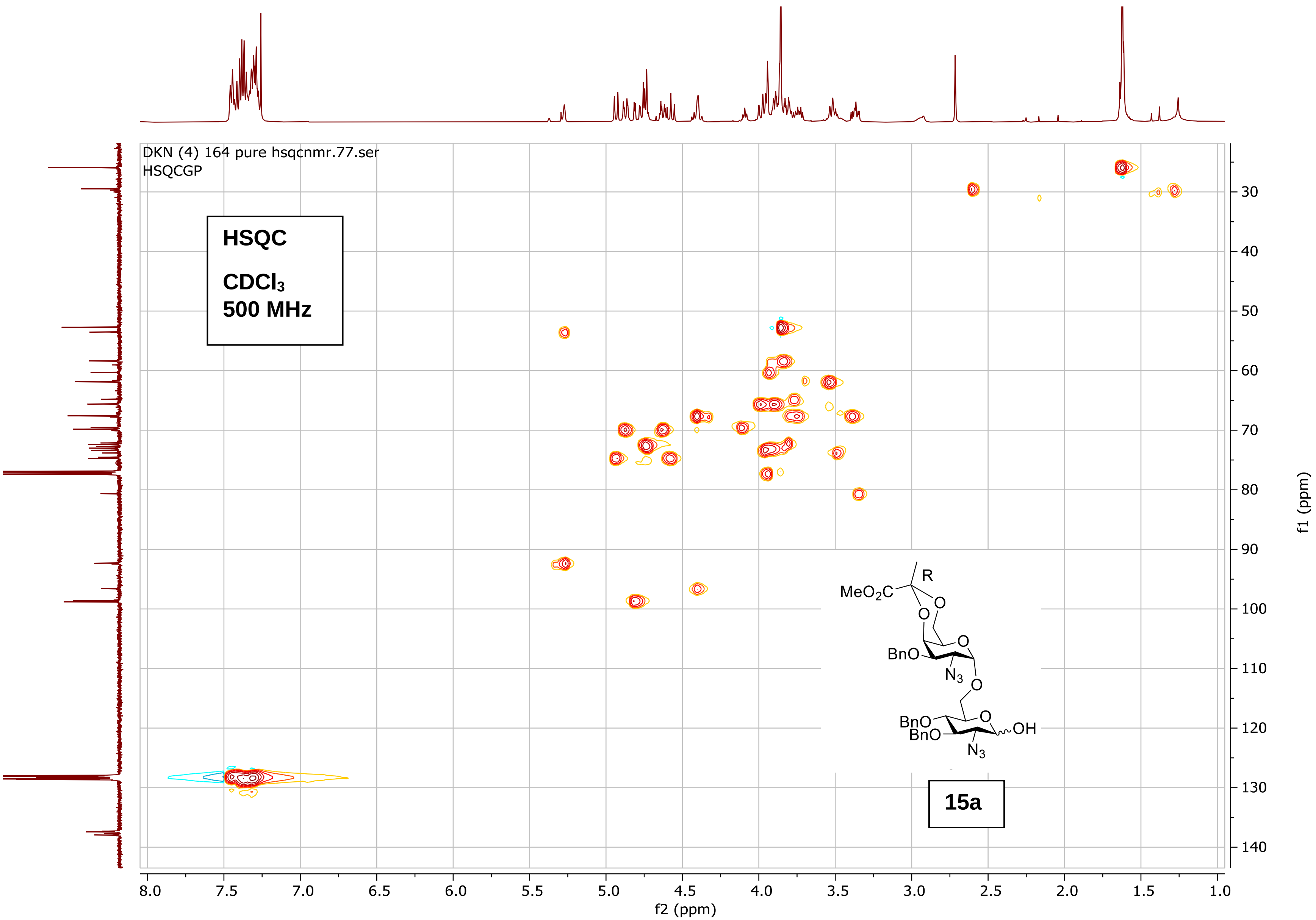
DKN (4) 164 pure hsqcnmr.77.ser
HSQCGP
HSQC
CDCl3
500 MHz
MeO2C
R
O
O
O
BnO
N3
O
BnO
BnO
O
OH
N3
15a
f2 (ppm)
f1 (ppm)
8.0
7.5
7.0
6.5
6.0
5.5
5.0
4.5
4.0
3.5
3.0
2.5
2.0
1.5
1.0
30
40
50
60
70
80
90
100
110
120
130
140

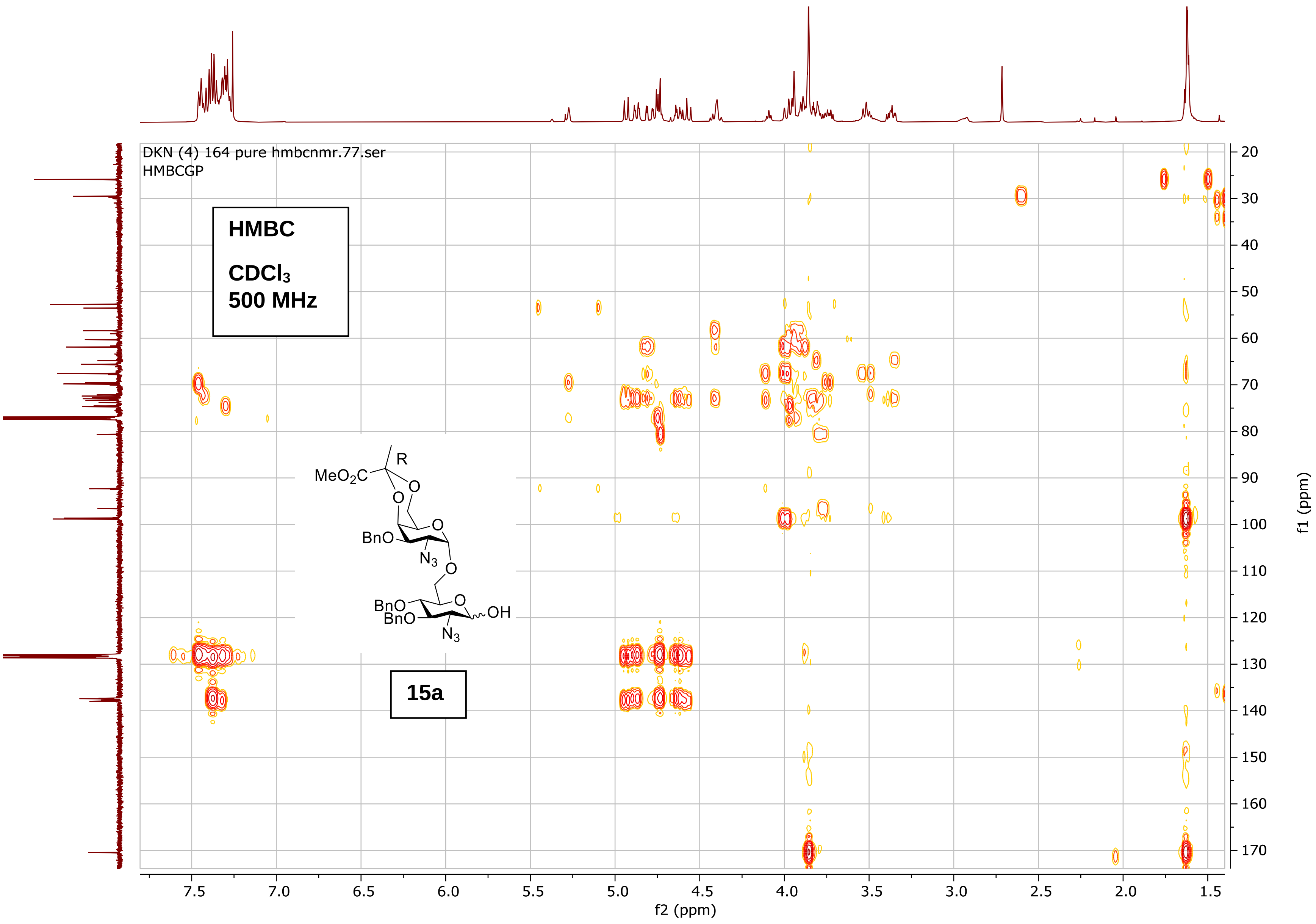

DKN (4) 164 pure hmbcnmr.77.ser
HMBCGP
HMBC
CDCl3
500 MHz
MeO2C
R
O
O
O
BnO
N3
O
BnO
BnO
O
OH
N3
15a
f2 (ppm)
f1 (ppm)

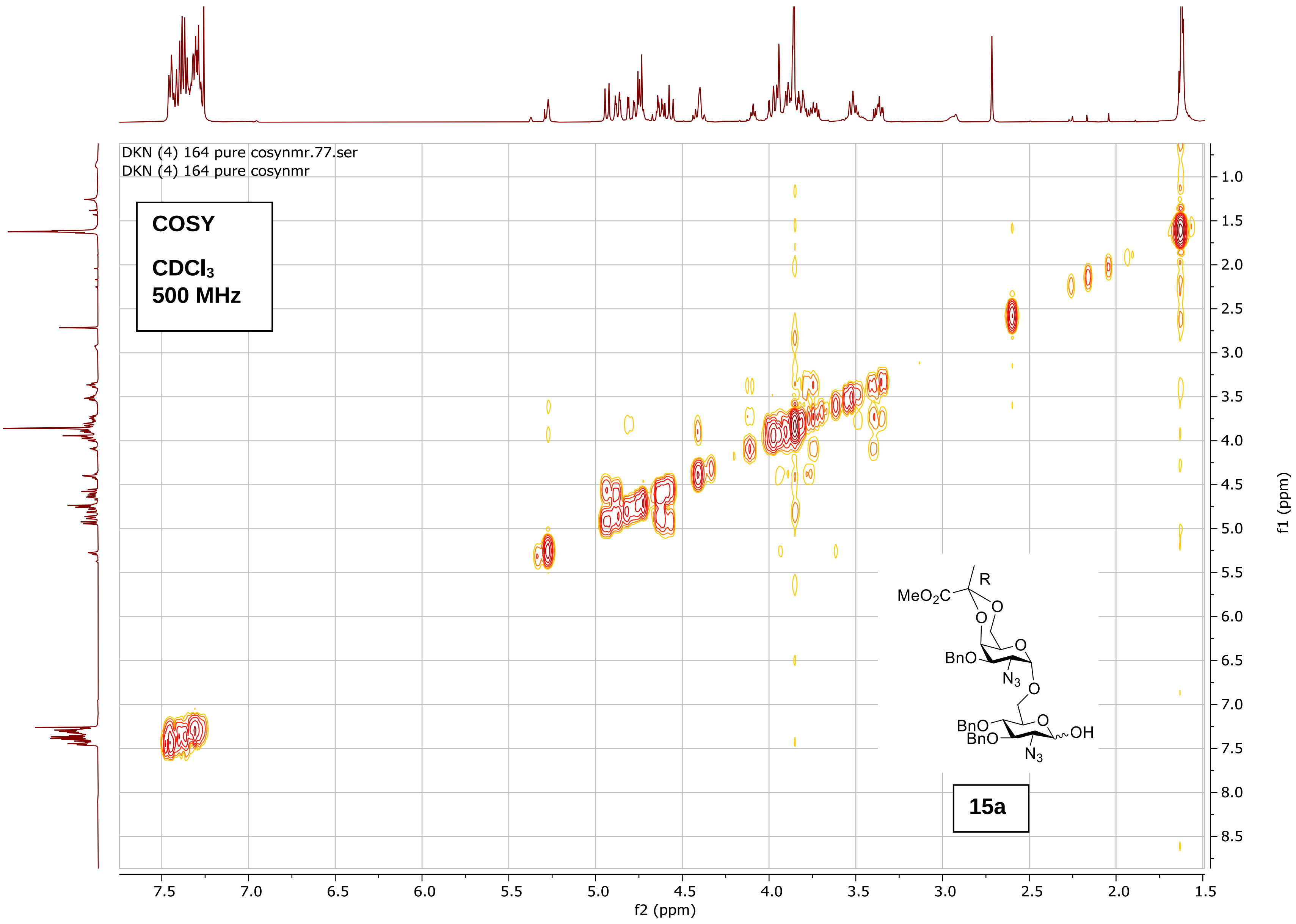
DKN (4) 164 pure cosynmr.77.ser
DKN (4) 164 pure cosynmr
COSY
$CDCl_3$
500 MHz
MeO₂C
R
O
O
O
BnO
N_3
O
BnO
BnO
OH
N_3
f2 (ppm)
f1 (ppm)

15a

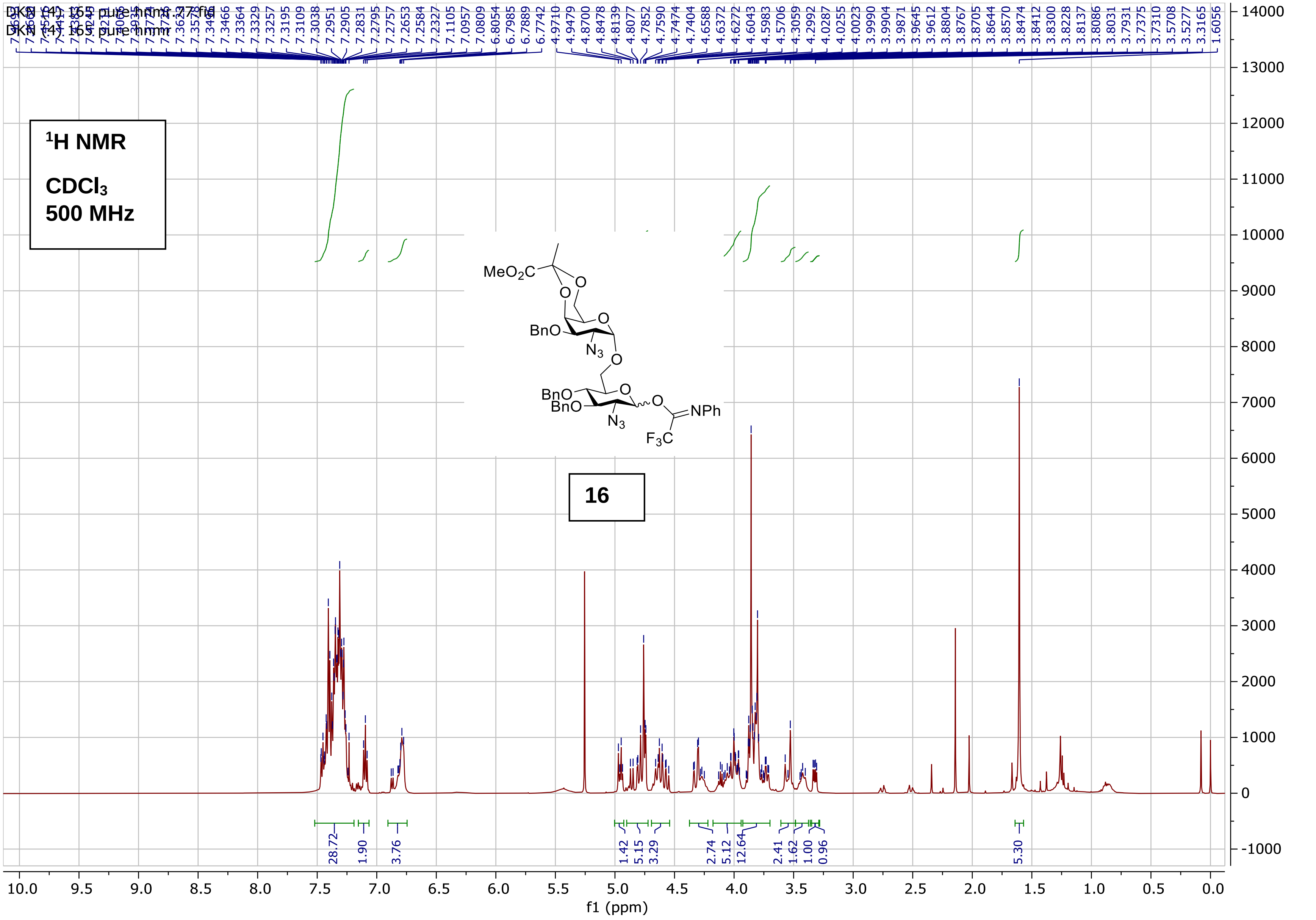

7.3466
7.3364
7.3329
7.3257
7.3195
7.3109
7.3038
7.2951
7.2905
7.2831
7.2795
7.2757
7.2653
7.2584
7.2327
7.1105
7.0957
7.0809
6.8054
6.7985
6.7889
6.7742
4.9710
4.9479
4.8700
4.8478
4.8139
4.8077
4.7852
4.7590
4.7474
4.7404
4.6588
4.6372
4.6272
4.6043
4.5983
4.5706
4.3059
4.2992
4.0287
4.0255
4.0023
3.9990
3.9904
3.9871
3.9645
3.9612
3.8804
3.8767
3.8705
3.8644
3.8570
3.8474
3.8412
3.8300
3.8228
3.8137
3.8086
3.8031
3.7931
3.7375
3.7310
3.5708
3.5277
3.3165
1.6056
¹H NMR
CDCl3
500 MHz
MeO2C
O
O
O
BnO
N3
O
O
BnO
BnO
N3
O
NPh
F3C
16
28.72
1.90
3.76
1.42
5.15
3.29
2.74
5.12
12.64
2.41
1.62
1.00
0.96
5.30
f1 (ppm)

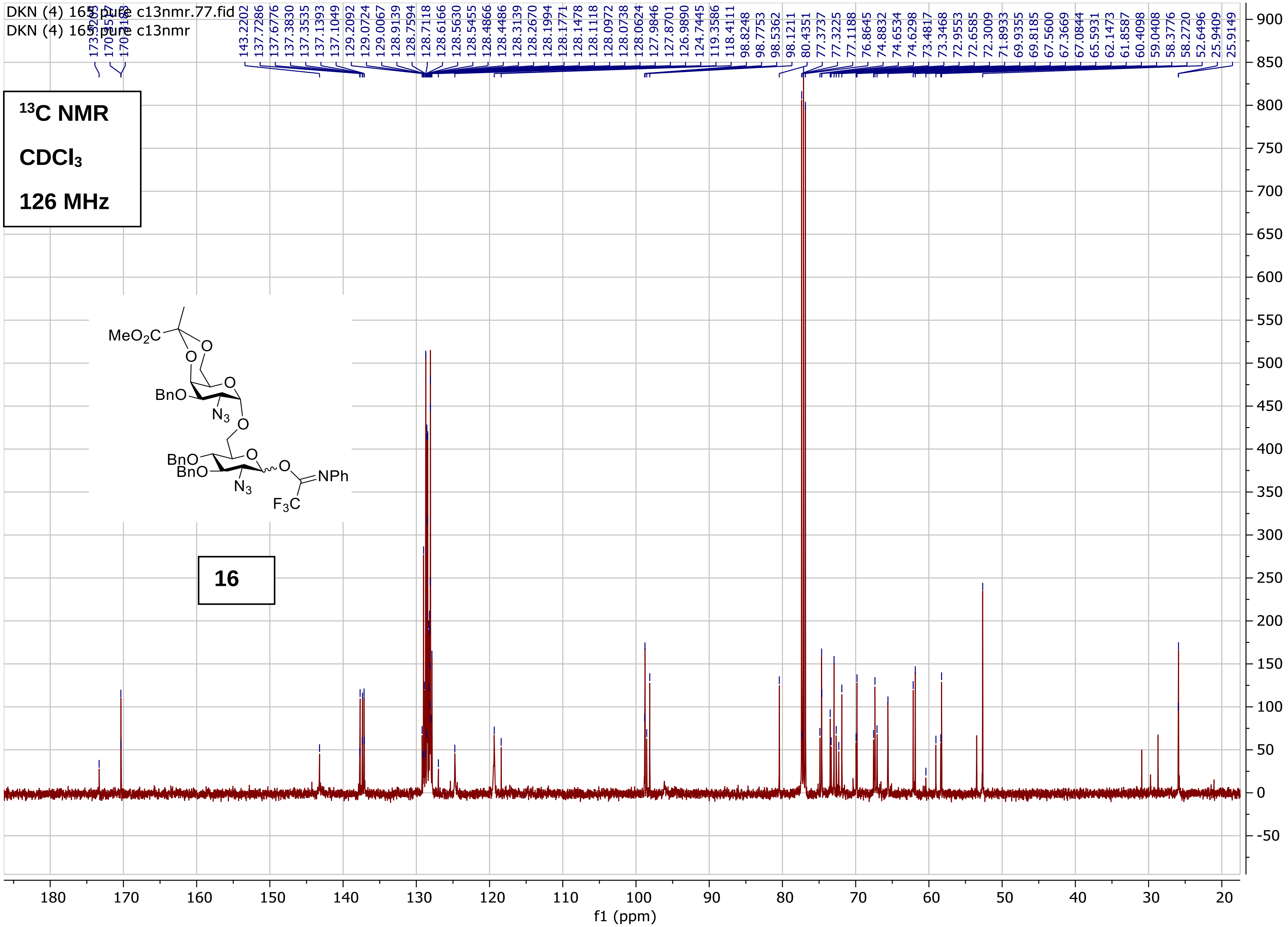

DKN (4) 165 pure c13nmr.77.fid
DKN (4) 165 pure c13nmr
143.2202
137.7286
137.6776
137.3830
137.3535
137.1393
137.1049
129.2092
129.0724
129.0067
128.9139
128.7594
128.7118
128.6166
128.5630
128.5455
128.4866
128.4486
128.3139
128.2670
128.1994
128.1771
128.1478
128.1118
128.0972
128.0738
128.0624
127.9846
127.8701
126.9890
124.7445
119.3586
118.4111
98.8248
98.7753
98.5362
98.1211
80.4351
77.3737
77.3225
77.1188
76.8645
74.8832
74.6534
74.6298
73.4817
73.3468
72.9553
72.6585
72.3009
71.8933
69.9355
69.8185
67.5600
67.3669
67.0844
65.5931
62.1473
61.8587
60.4098
59.0408
58.3776
58.2720
52.6496
25.9409
25.9149
13C NMR
CDCl3
126 MHz
MeO2C
O
BnO
N3
BnO
NPh
F3C
16
f1 (ppm)

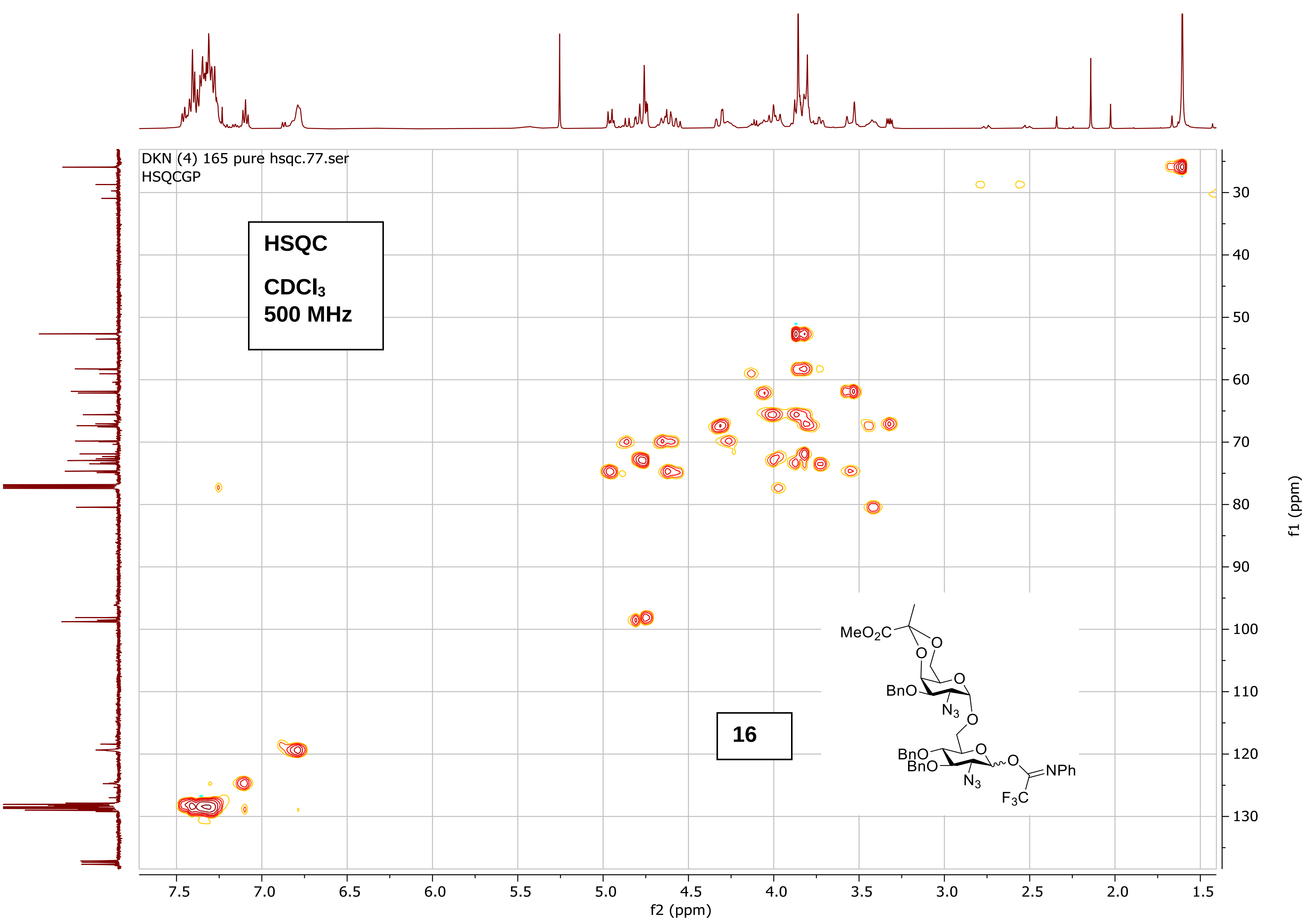

DKN (4) 165 pure hsqc.77.ser
HSQCGP
HSQC
$CDCl_3$
500 MHz
16
MeO_2C
O
O
O
BnO
N_3
O
BnO
BnO
O
N_3
O
NPh
F_3C
f2 (ppm)
f1 (ppm)

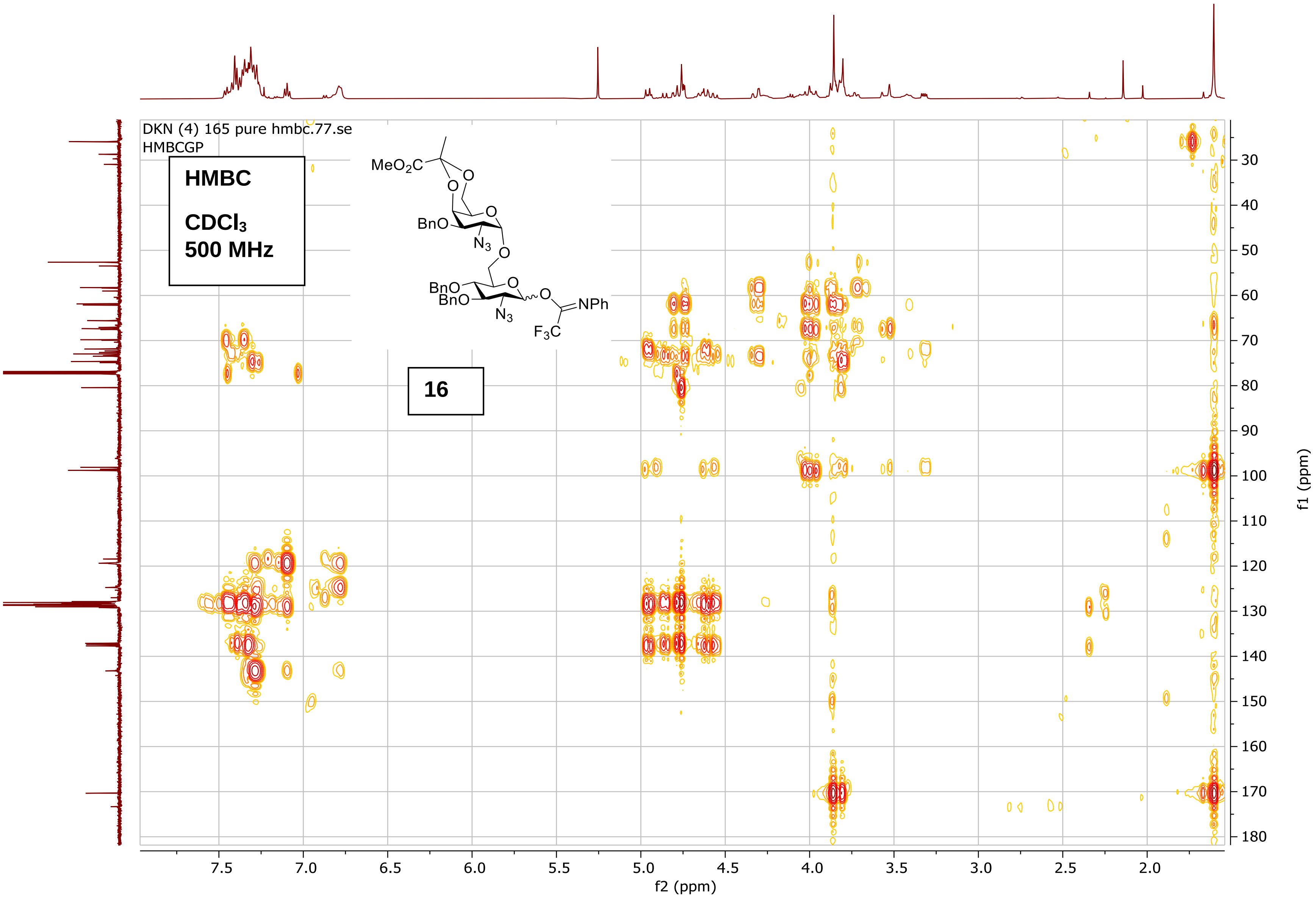

DKN (4) 165 pure hmbc.77.se
HMBCGP
HMBC
CDCl3
500 MHz
16
f2 (ppm)
f1 (ppm)

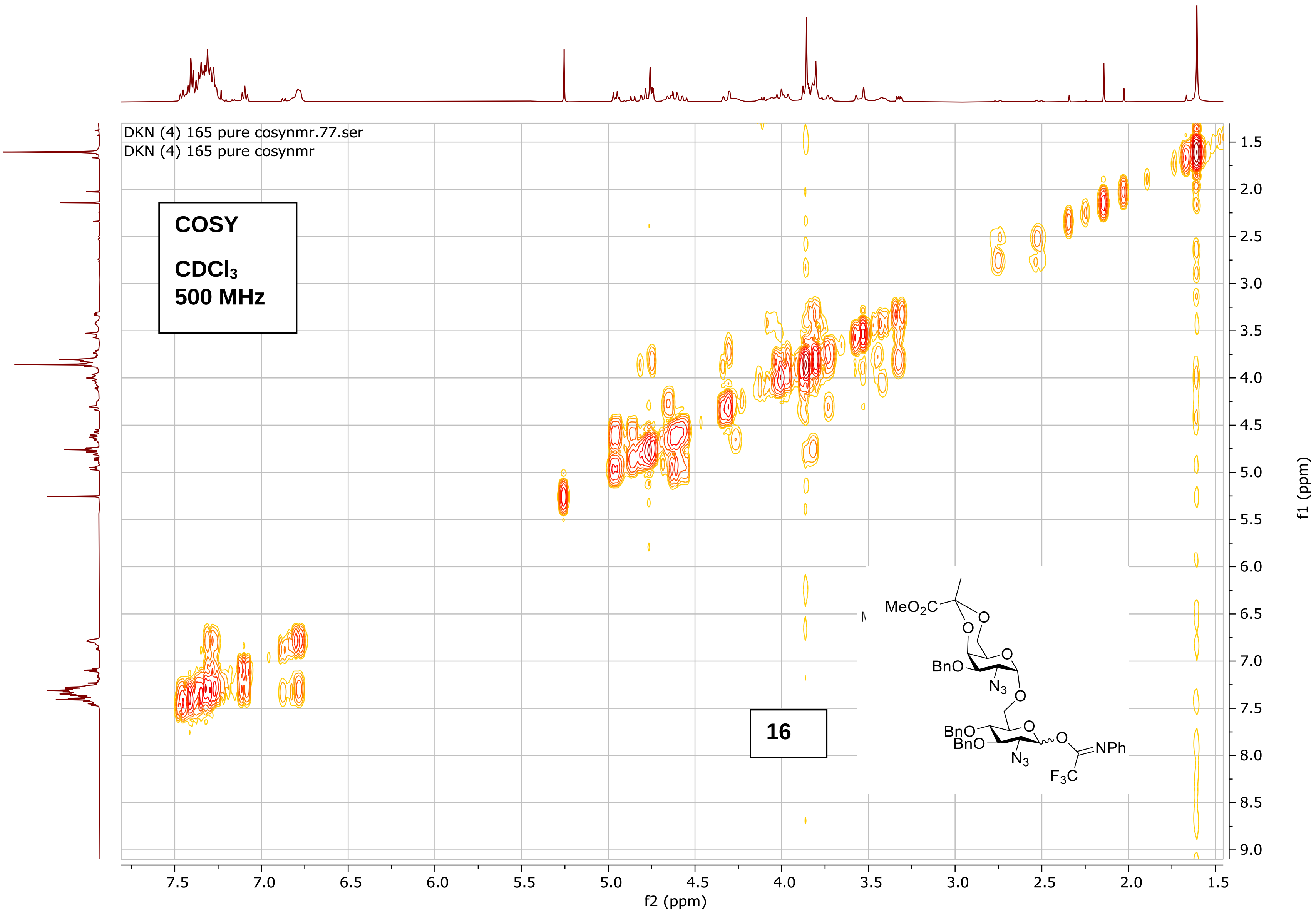
DKN (4) 165 pure cosynmr.77.ser
DKN (4) 165 pure cosynmr
COSY
$CDCl_3$
500 MHz
16
MeO_2C
BnO
N_3
BnO
BnO
N_3
NPh
F_3C
f2 (ppm)
f1 (ppm)

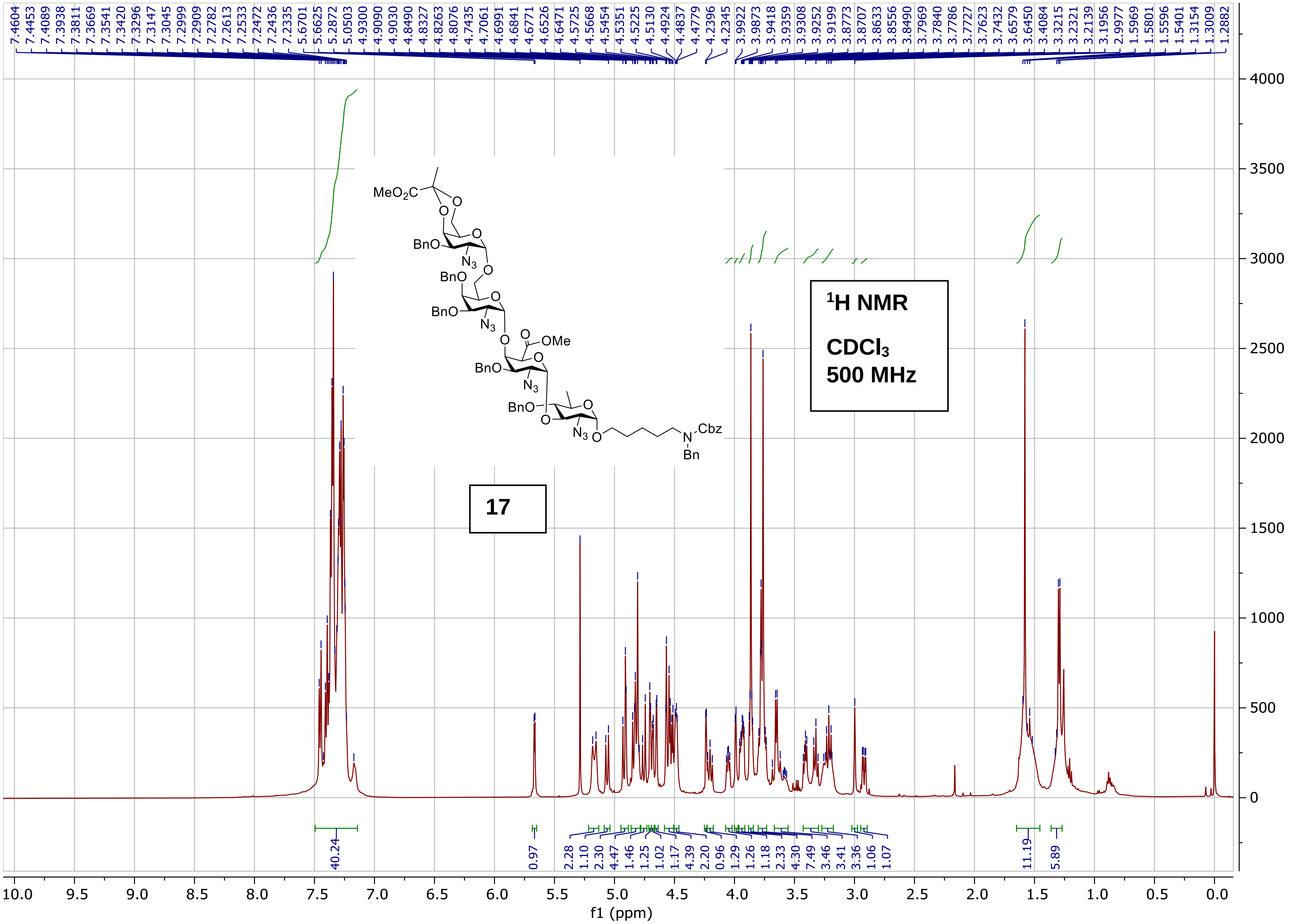

¹H NMR
CDCl₃
500 MHz
17
MeO₂C
BnO
N₃
OMe
Cbz
Bn
f1 (ppm)

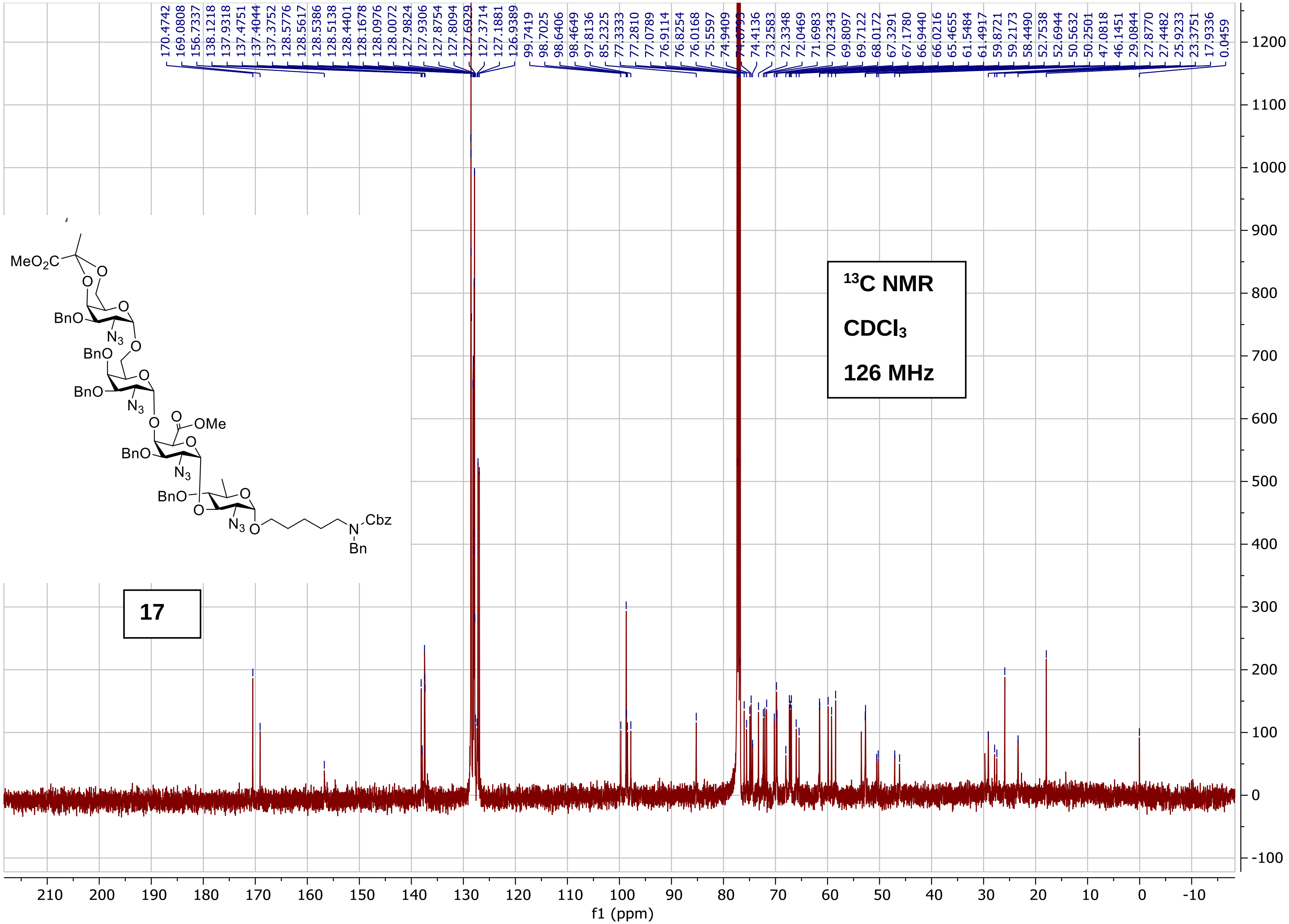
170.4742 169.0808 156.7337 138.1218 137.9318 137.4751 137.4044 137.3752 128.5776 128.5617 128.5386 128.5138 128.4401 128.1678 128.0976 128.0072 127.9824 127.9306 127.8754 127.8094 127.6929 127.3714 127.1881 126.9389 99.7419 98.7025 98.6406 98.4649 97.8136 85.2325 77.3333 77.2810 77.0789 76.9114 76.8254 76.0168 75.5597 74.9409 74.4136 73.2583 72.3348 72.0469 71.6983 70.2343 69.8097 69.7122 68.0172 67.3291 67.1780 66.9440 66.0216 65.4655 61.5484 61.4917 59.8721 59.2173 58.4490 52.7538 52.6944 50.5632 50.2501 47.0818 46.1451 29.0844 27.8770 27.4482 25.9233 23.3751 17.9336 0.0459
MeO2C
O
O
O
BnO
N3
O
BnO
O
BnO
N3
O
O
OMe
O
BnO
N3
BnO
O
O
N3
O
N
Cbz
Bn
17
13C NMR
CDCl3
126 MHz
f1 (ppm)

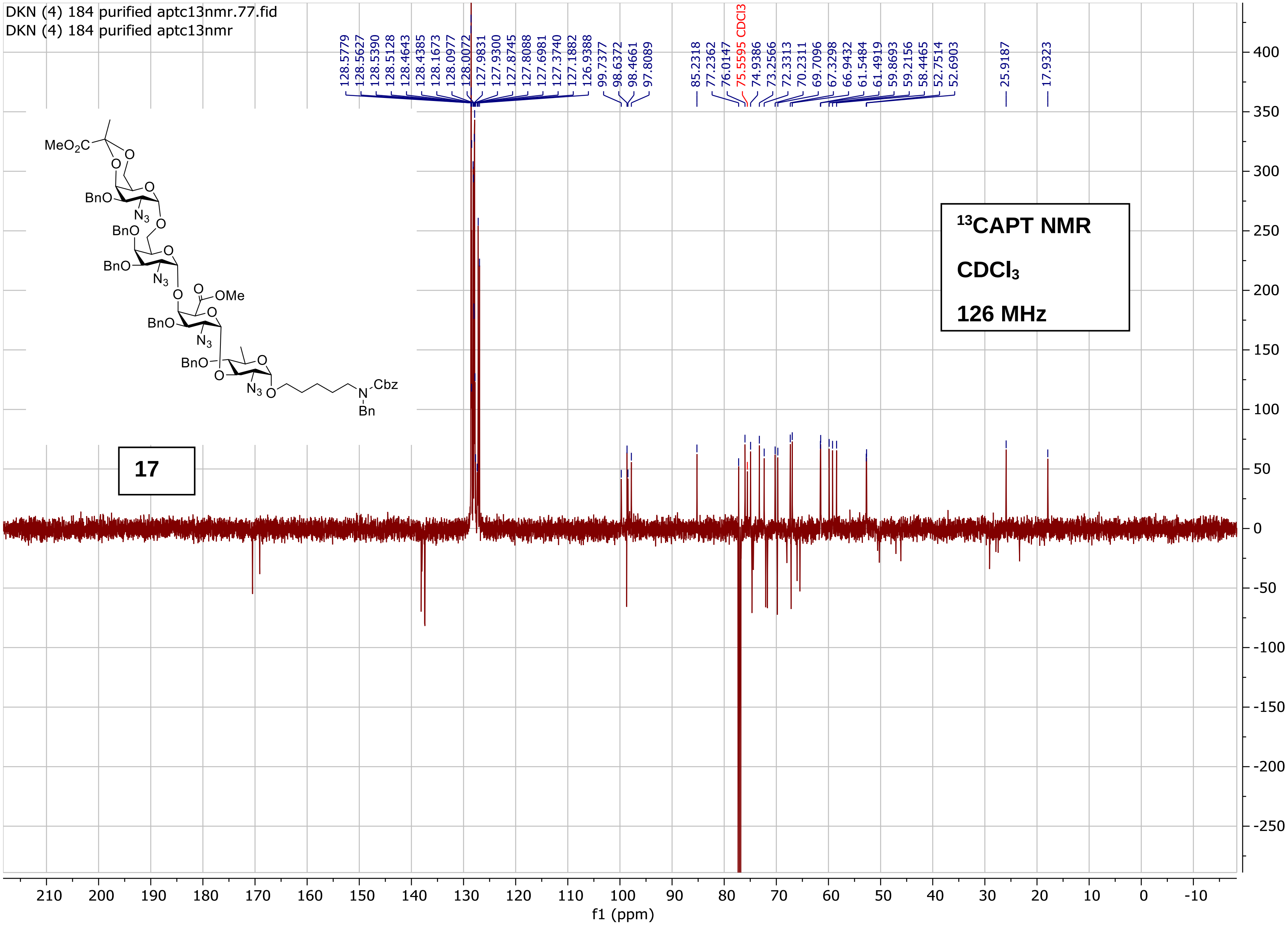
DKN (4) 184 purified aptc13nmr.77.fid
DKN (4) 184 purified aptc13nmr
128.5779
128.5627
128.5390
128.5128
128.4643
128.4385
128.1673
128.0977
128.0072
127.9831
127.9300
127.8745
127.8088
127.6981
127.3740
127.1882
126.9388
99.7377
98.6372
98.4661
97.8089
85.2318
77.2362
76.0147
75.5595 CDCl3
74.9386
73.2566
72.3313
70.2311
69.7096
67.3298
66.9432
61.5484
61.4919
59.8693
59.2156
58.4465
52.7514
52.6903
25.9187
17.9323
MeO2C
BnO
N3
17
13CAPT NMR
CDCl3
126 MHz
f1 (ppm)

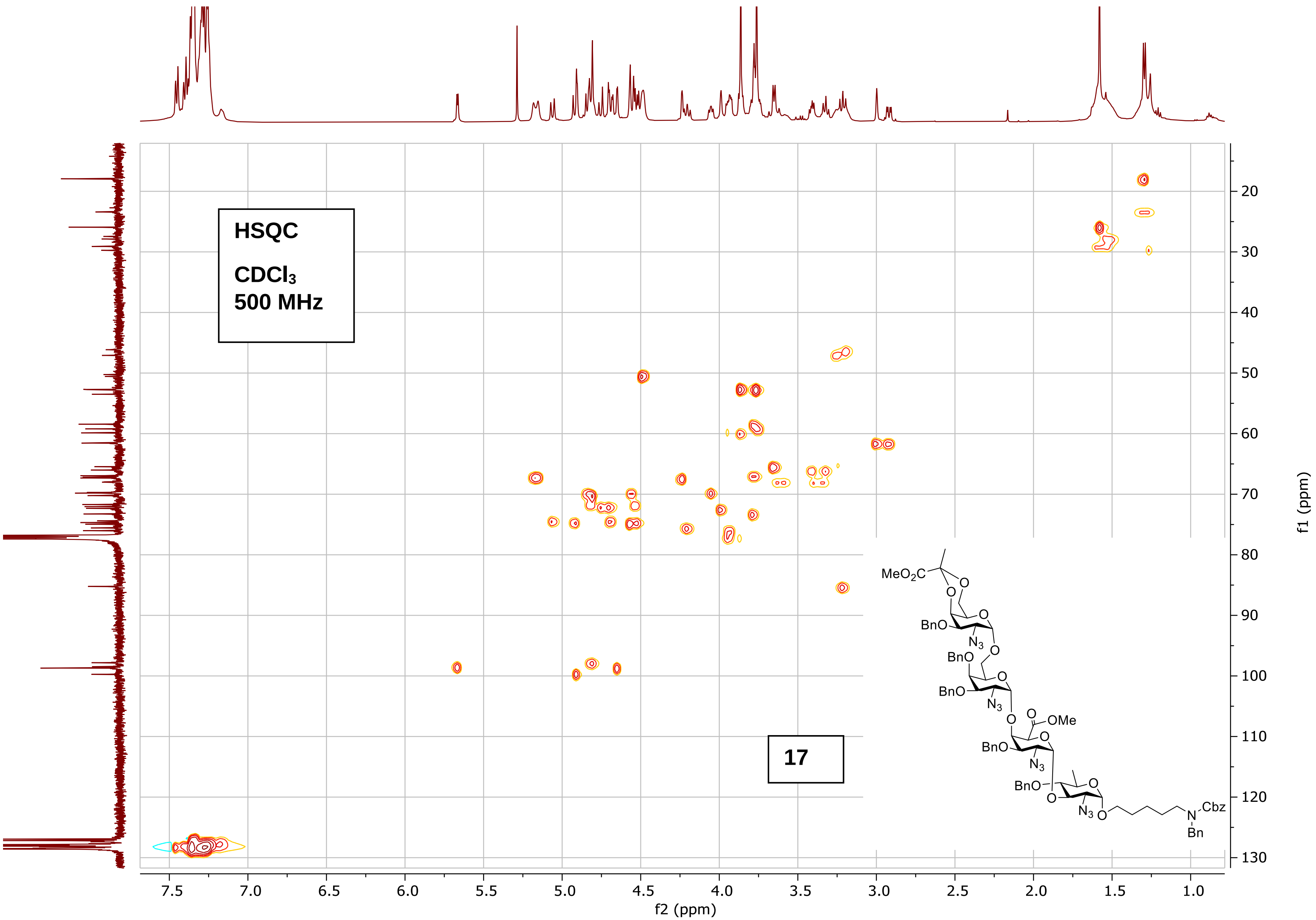
HSQC
CDCl3
500 MHz
17
MeO2C
BnO
N3
OMe
N
Cbz
Bn
f2 (ppm)
f1 (ppm)

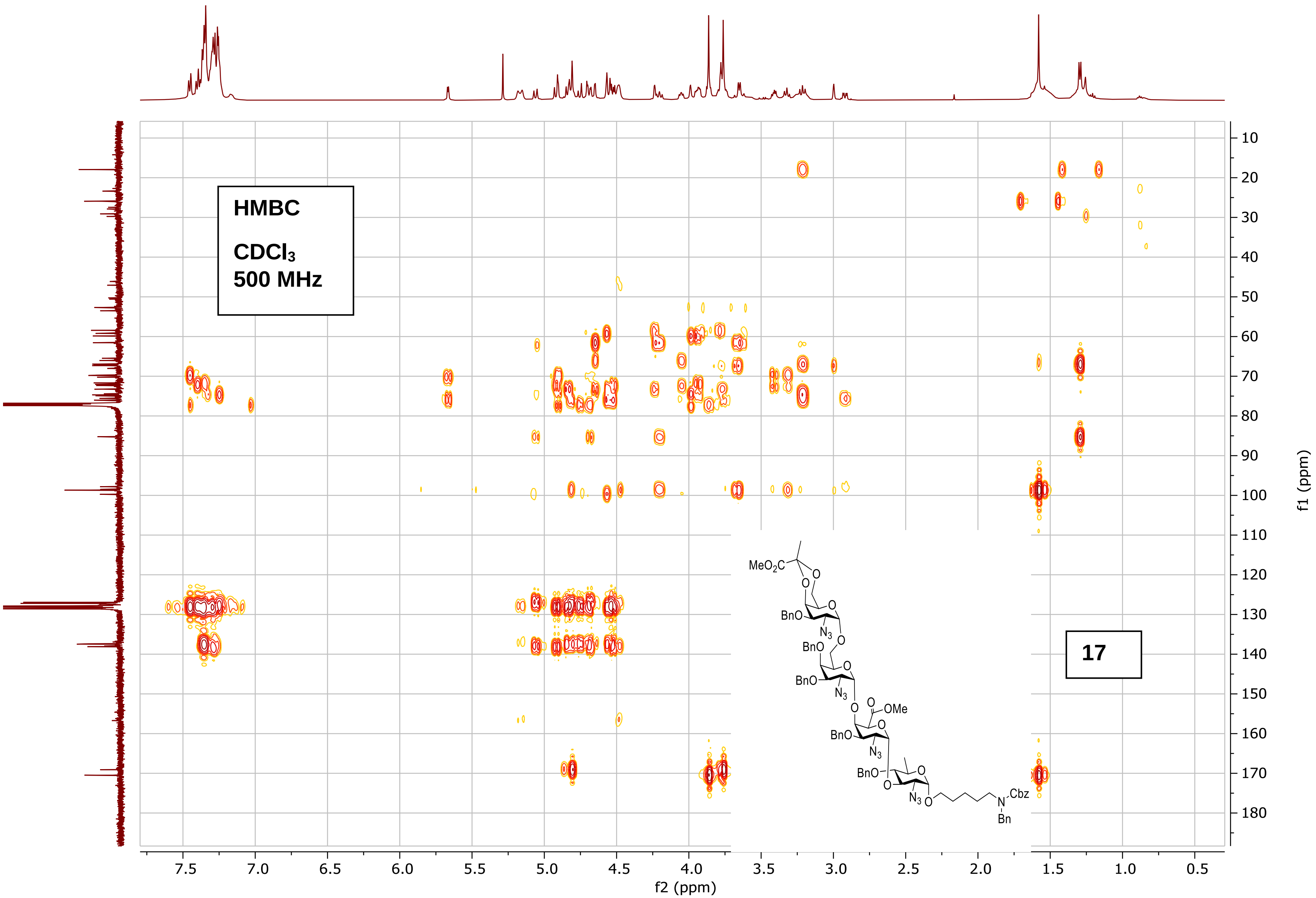
HMBC
CDCl3
500 MHz
17
MeO2C
BnO
N3
OMe
N
Cbz
Bn
f2 (ppm)
f1 (ppm)

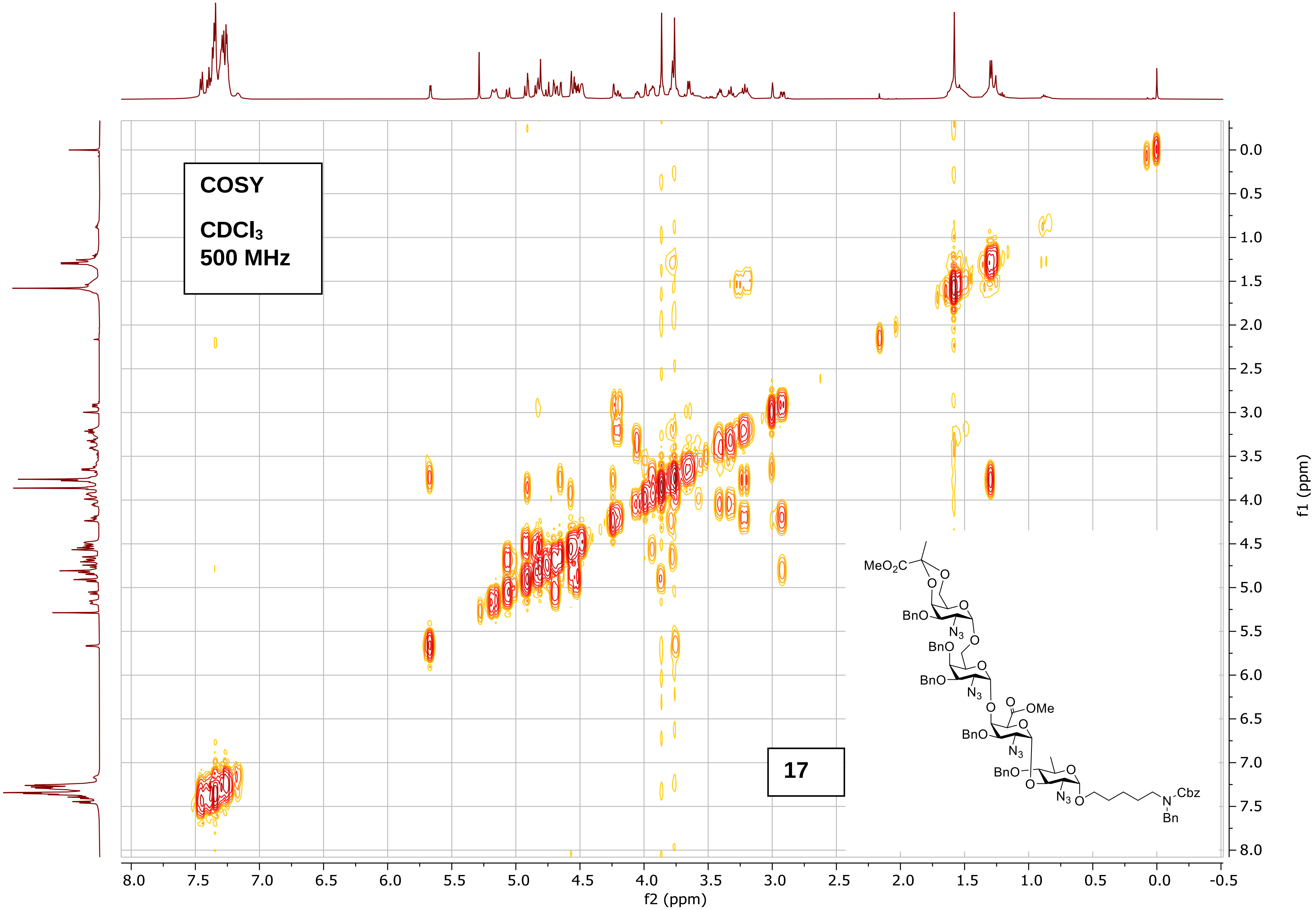
COSY
CDCl3
500 MHz
17
MeO2C
BnO
N3
OMe
Cbz
Bn
f2 (ppm)
f1 (ppm)

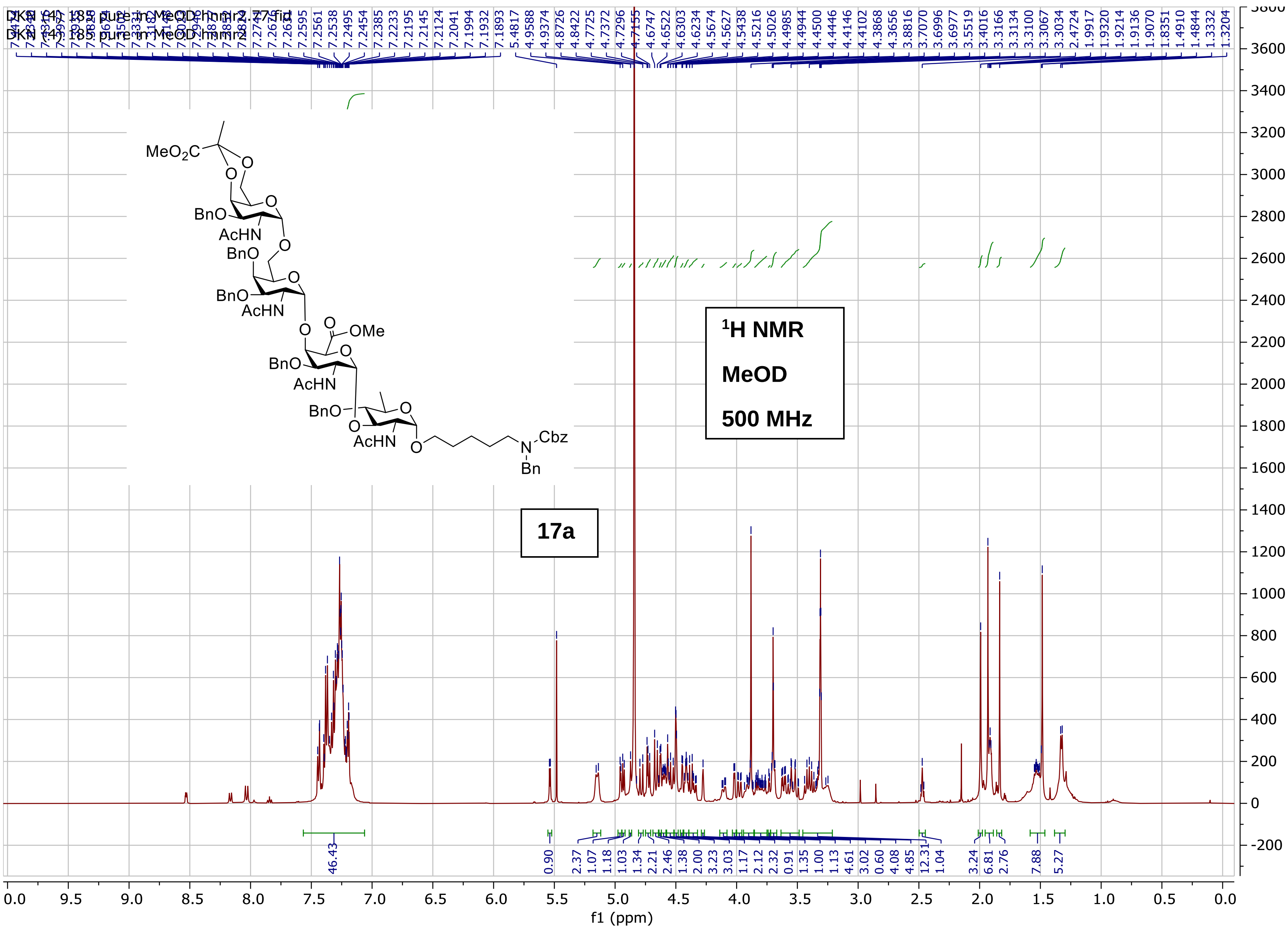

1H NMR
MeOD
500 MHz
17a
MeO2C
BnO
AcHN
OMe
Cbz
Bn
f1 (ppm)

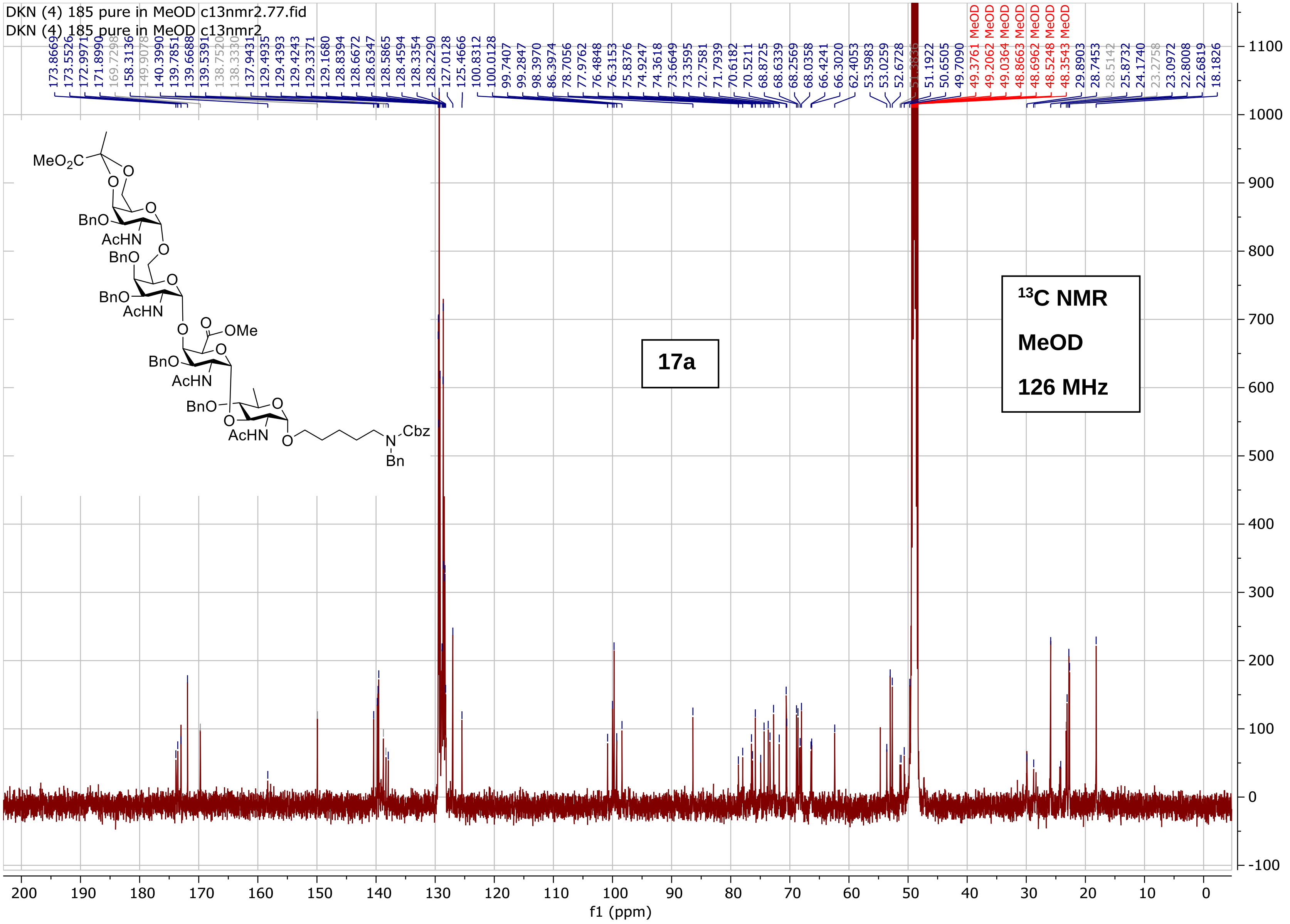
DKN (4) 185 pure in MeOD c13nmr2.77.fid
DKN (4) 185 pure in MeOD c13nmr2
173.8669
173.5526
172.9971
171.8990
169.7298
158.3136
149.9078
140.3990
139.7851
139.6688
139.5391
138.7520
138.3330
137.9431
129.4935
129.4393
129.4243
129.3371
129.1680
128.8394
128.6672
128.6347
128.5865
128.4594
128.3354
128.2290
127.0128
125.4666
100.8312
100.0128
99.7407
99.2847
98.3970
86.3974
78.7056
77.9762
76.4848
76.3153
75.8376
74.9247
74.3618
73.6649
73.3595
72.7581
71.7939
70.6182
70.5211
68.8725
68.6339
68.2569
68.0358
66.4241
66.3020
62.4053
53.5983
53.0259
52.6728
51.1922
50.6505
49.7090
49.3761 MeOD
49.2062 MeOD
49.0364 MeOD
48.8663 MeOD
48.6962 MeOD
48.5248 MeOD
48.3543 MeOD
29.8903
28.7453
28.5142
25.8732
24.1740
23.2758
23.0972
22.8008
22.6819
18.1826
MeO2C
O
O
O
BnO
AcHN
O
BnO
O
BnO
AcHN
O
O
OMe
O
BnO
AcHN
BnO
O
O
AcHN
O
N
Cbz
Bn
17a
13C NMR
MeOD
126 MHz
f1 (ppm)

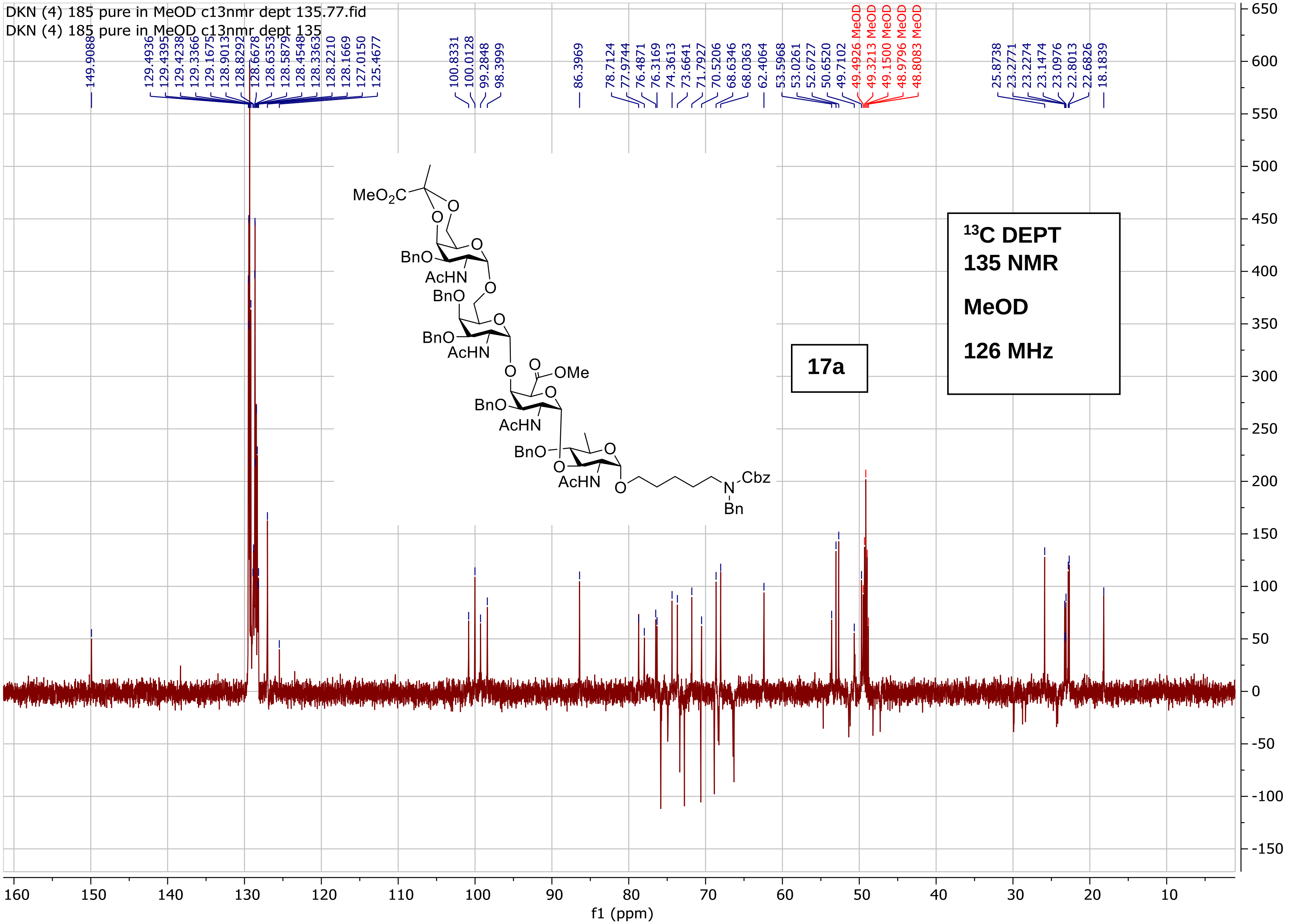

DKN (4) 185 pure in MeOD c13nmr dept 135.77.fid
DKN (4) 185 pure in MeOD c13nmr dept 135
149.9088
129.4936
129.4395
129.4238
129.3366
129.1675
128.9013
128.8292
128.6678
128.6353
128.5879
128.4548
128.3363
128.2210
128.1669
127.0150
125.4677
100.8331
100.0128
99.2848
98.3999
86.3969
78.7124
77.9744
76.4871
76.3169
74.3613
73.6641
71.7927
70.5206
68.6346
68.0363
62.4064
53.5968
53.0261
52.6727
50.6520
49.7102
49.4926 MeOD
49.3213 MeOD
49.1500 MeOD
48.9796 MeOD
48.8083 MeOD
25.8738
23.2771
23.2274
23.1474
23.0976
22.8013
22.6826
18.1839
MeO2C
BnO
AcHN
OMe
Cbz
Bn
17a
13C DEPT
135 NMR
MeOD
126 MHz
f1 (ppm)

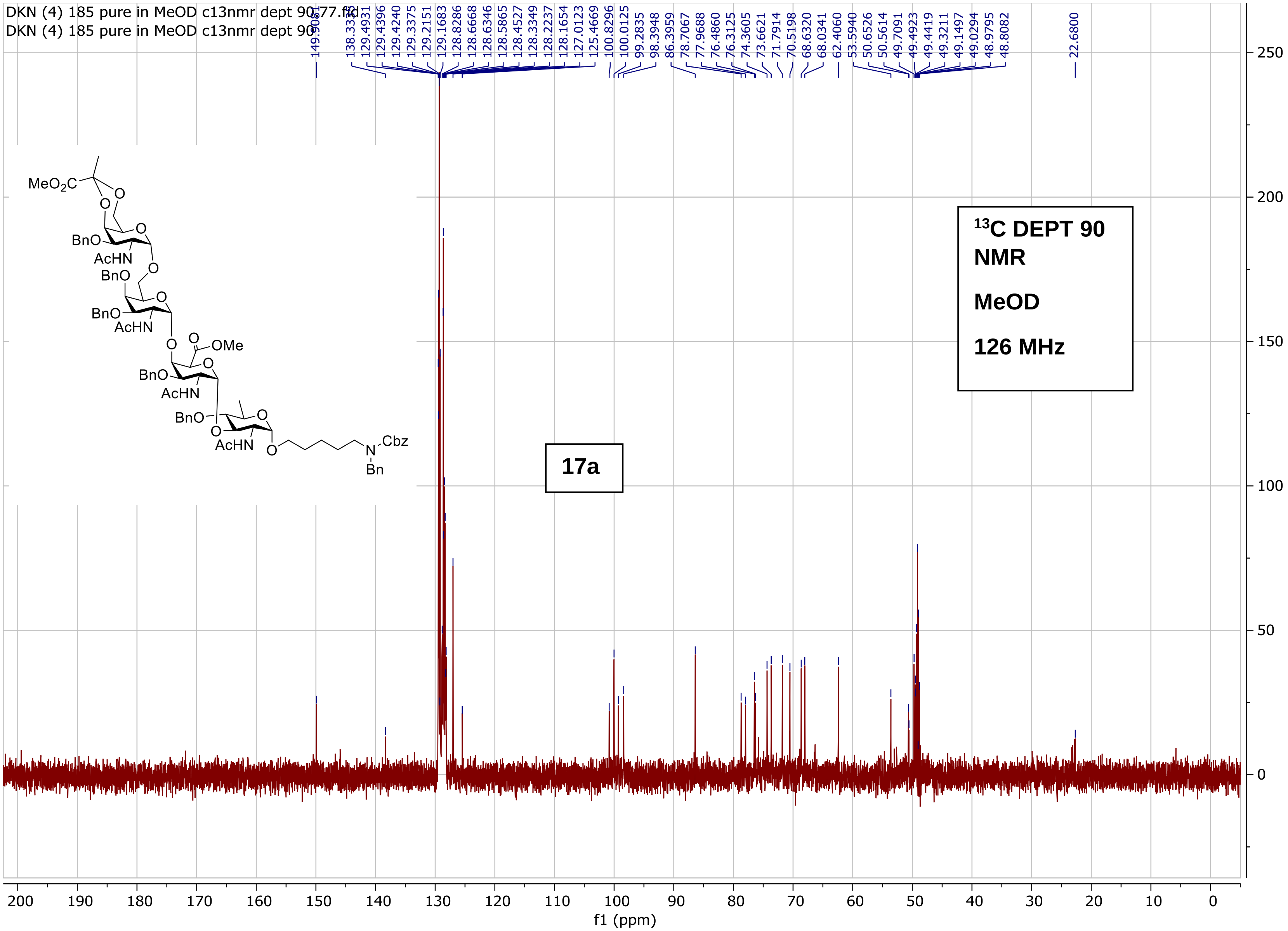
DKN (4) 185 pure in MeOD c13nmr dept 90.77.fid
DKN (4) 185 pure in MeOD c13nmr dept 90
149.9081
138.3332
129.4931
129.4396
129.4240
129.3375
129.2151
129.1683
128.8286
128.6668
128.6346
128.5865
128.4527
128.3349
128.2237
128.1654
127.0123
125.4669
100.8296
100.0125
99.2835
98.3948
86.3959
78.7067
77.9688
76.4860
76.3125
74.3605
73.6621
71.7914
70.5198
68.6320
68.0341
62.4060
53.5940
50.6526
50.5614
49.7091
49.4923
49.4419
49.3211
49.1497
49.0294
48.9795
48.8082
22.6800
MeO2C
O
O
O
BnO
AcHN
O
BnO
O
BnO
AcHN
O
O
OMe
O
BnO
AcHN
BnO
O
O
AcHN
O
N
Cbz
Bn
17a
13C DEPT 90
NMR
MeOD
126 MHz
f1 (ppm)

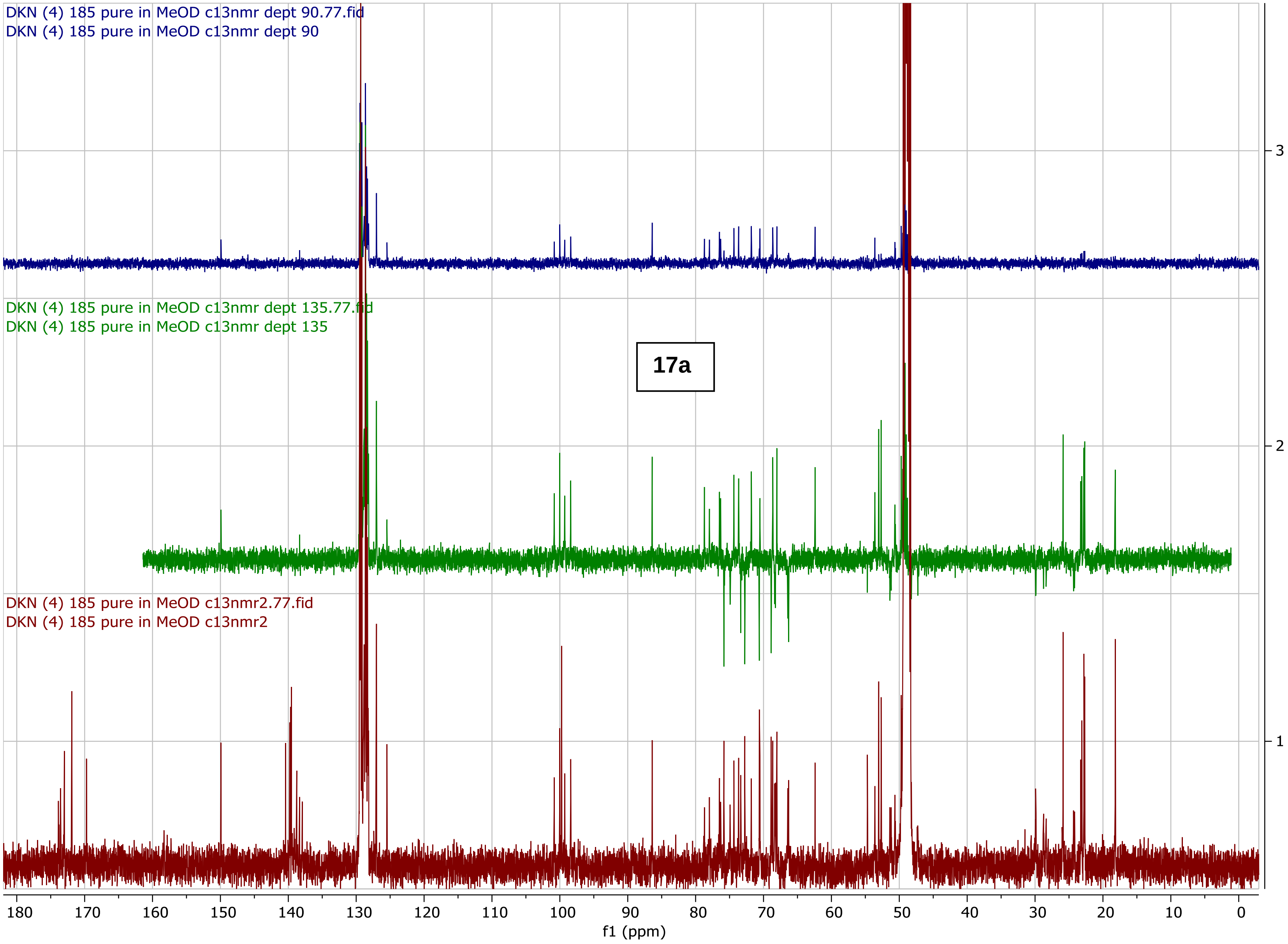
DKN (4) 185 pure in MeOD c13nmr dept 90.77.fid
DKN (4) 185 pure in MeOD c13nmr dept 90
DKN (4) 185 pure in MeOD c13nmr dept 135.77.fid
DKN (4) 185 pure in MeOD c13nmr dept 135
17a
DKN (4) 185 pure in MeOD c13nmr2.77.fid
DKN (4) 185 pure in MeOD c13nmr2
180 170 160 150 140 130 120 110 100 90 80 70 60 50 40 30 20 10 0
f1 (ppm)
3
2
1

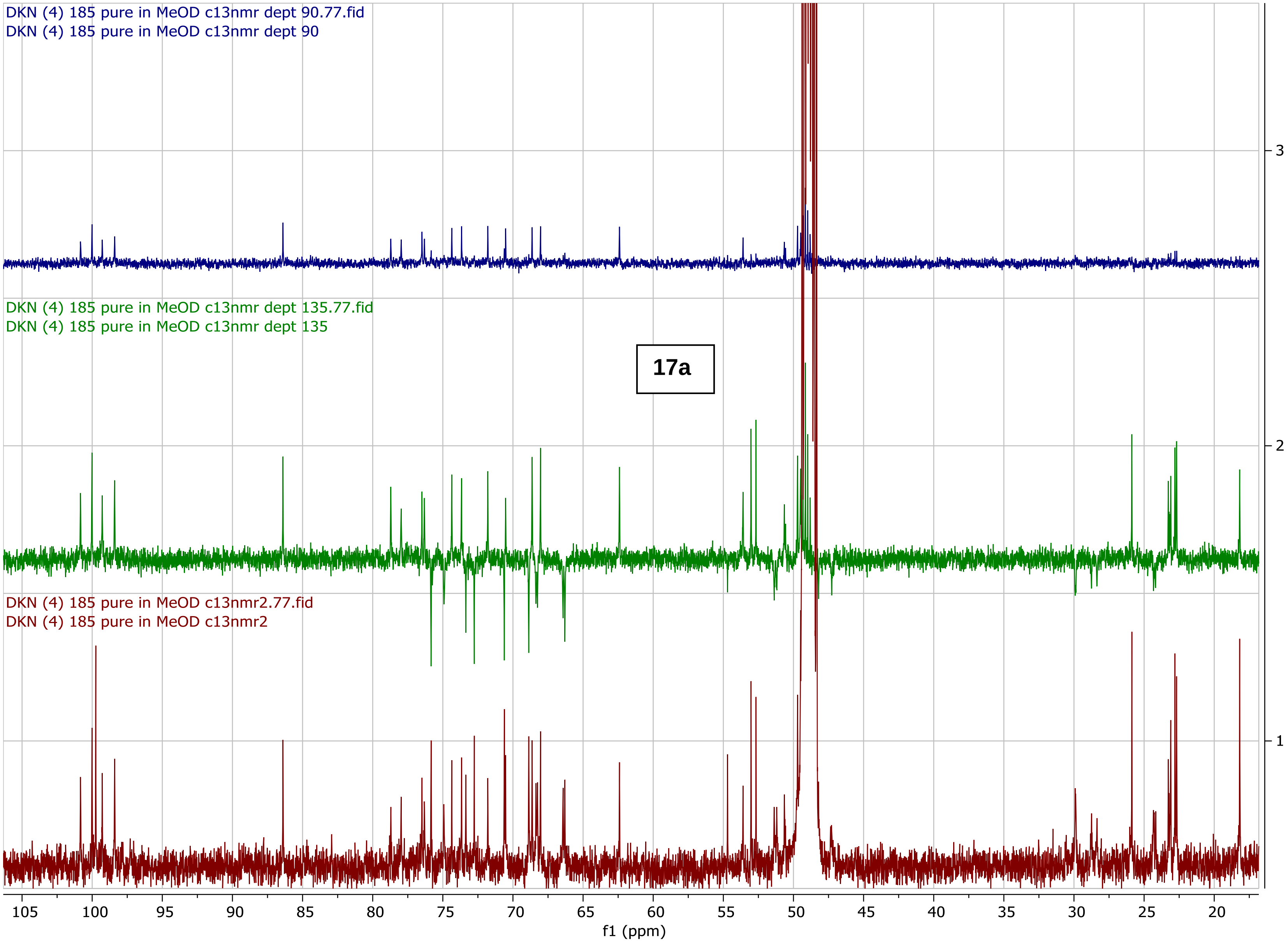
DKN (4) 185 pure in MeOD c13nmr dept 90.77.fid
DKN (4) 185 pure in MeOD c13nmr dept 90
DKN (4) 185 pure in MeOD c13nmr dept 135.77.fid
DKN (4) 185 pure in MeOD c13nmr dept 135
17a
DKN (4) 185 pure in MeOD c13nmr2.77.fid
DKN (4) 185 pure in MeOD c13nmr2
f1 (ppm)

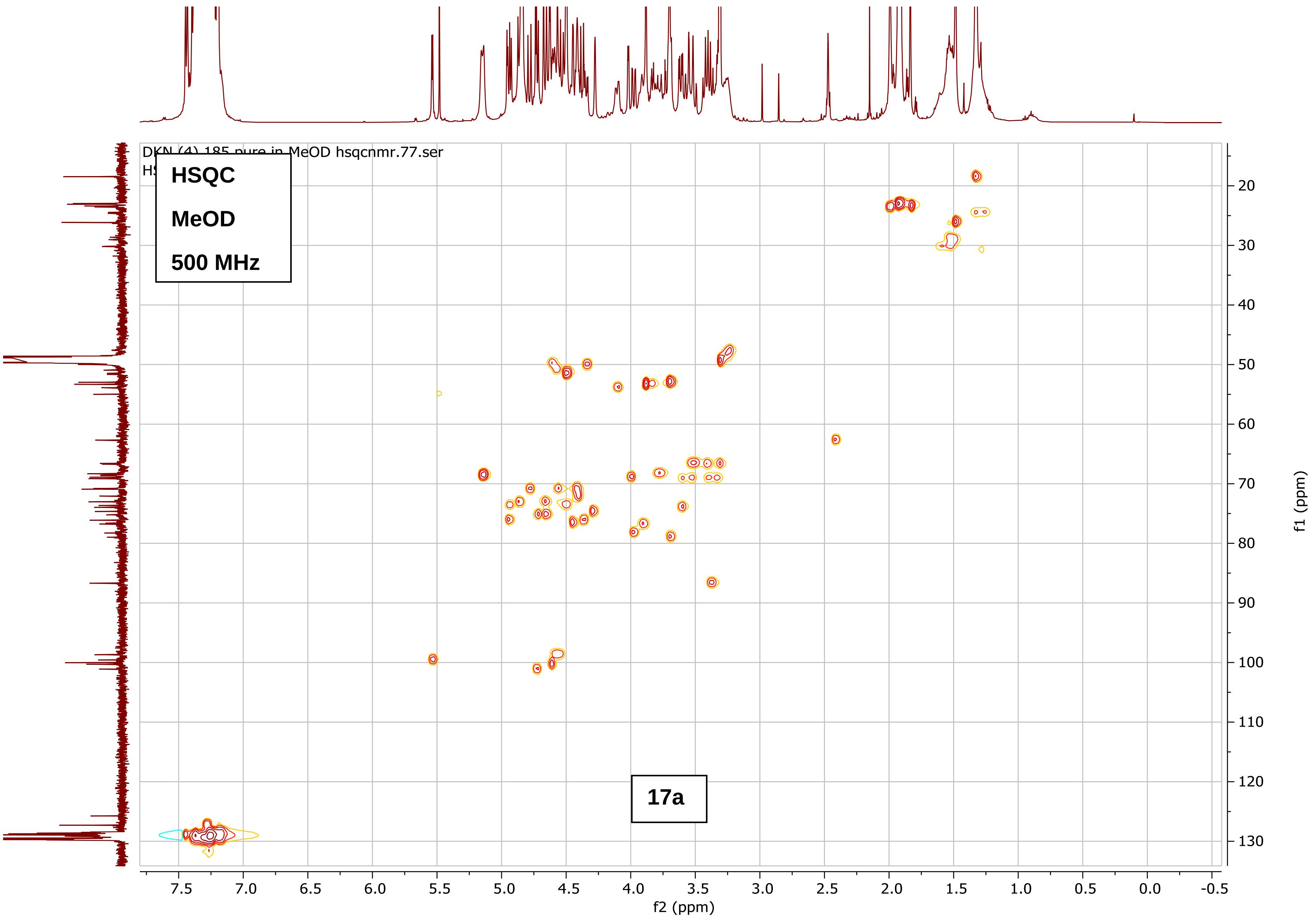
DKN (4) 185 pure in MeOD hsqcnmr.77.ser
HSQC
MeOD
500 MHz
17a
f2 (ppm)
f1 (ppm)

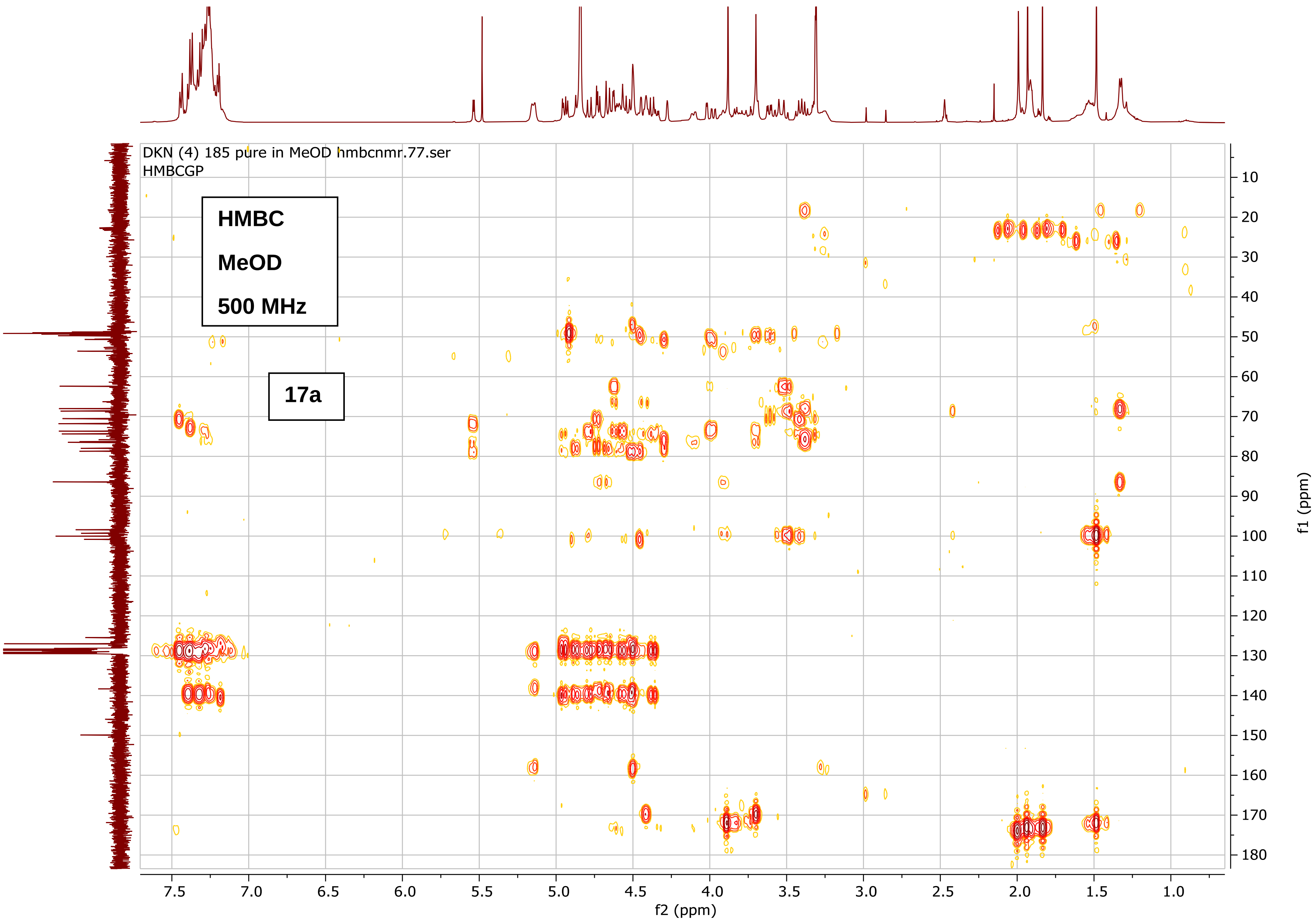

DKN (4) 185 pure in MeOD hmbcnmr.77.ser
HMBCGP
HMBC
MeOD
500 MHz
17a
f2 (ppm)
f1 (ppm)

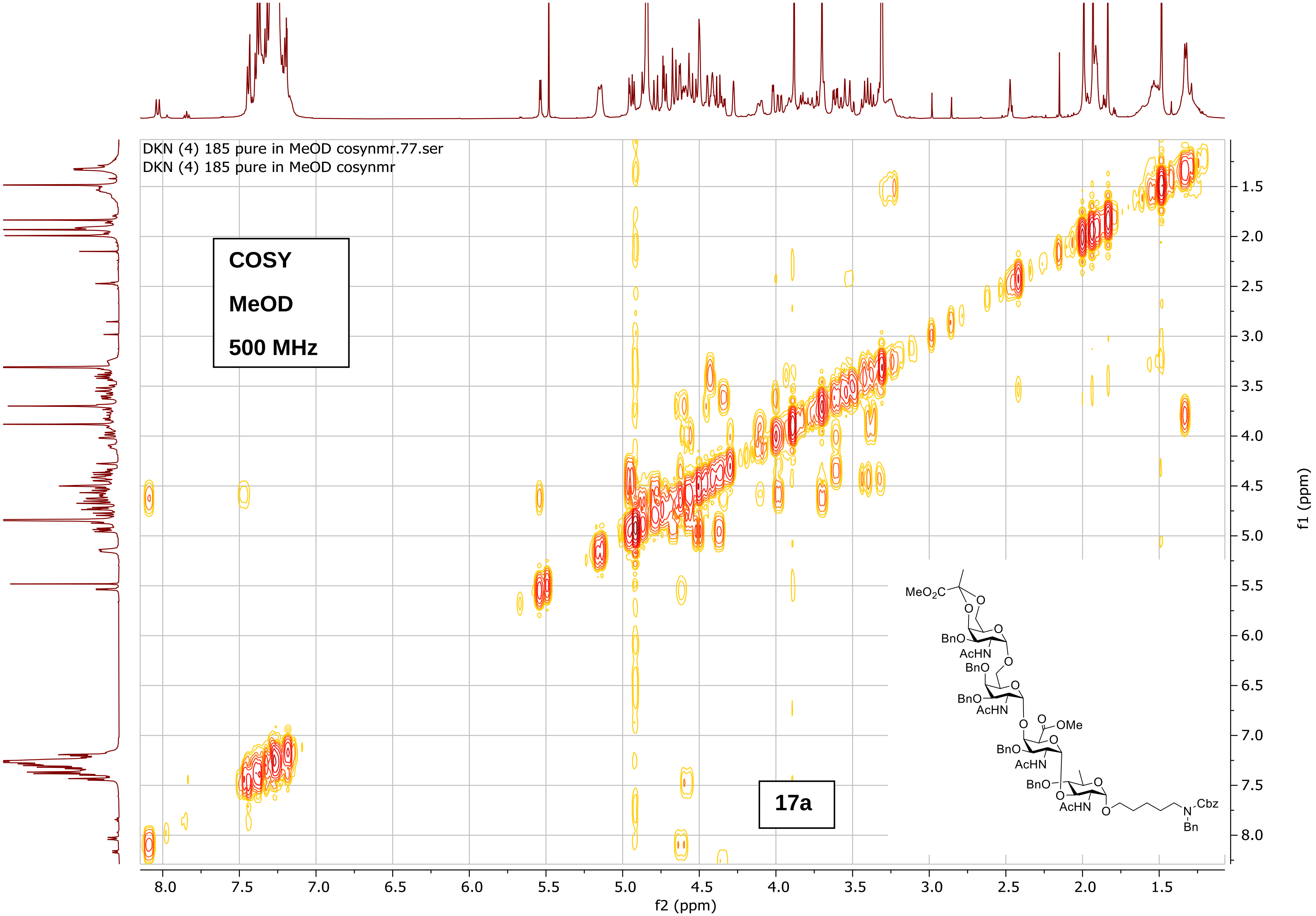
DKN (4) 185 pure in MeOD cosynmr.77.ser
DKN (4) 185 pure in MeOD cosynmr
COSY
MeOD
500 MHz
17a
f2 (ppm)
f1 (ppm)
MeO2C
BnO
AcHN
OMe
Cbz
Bn

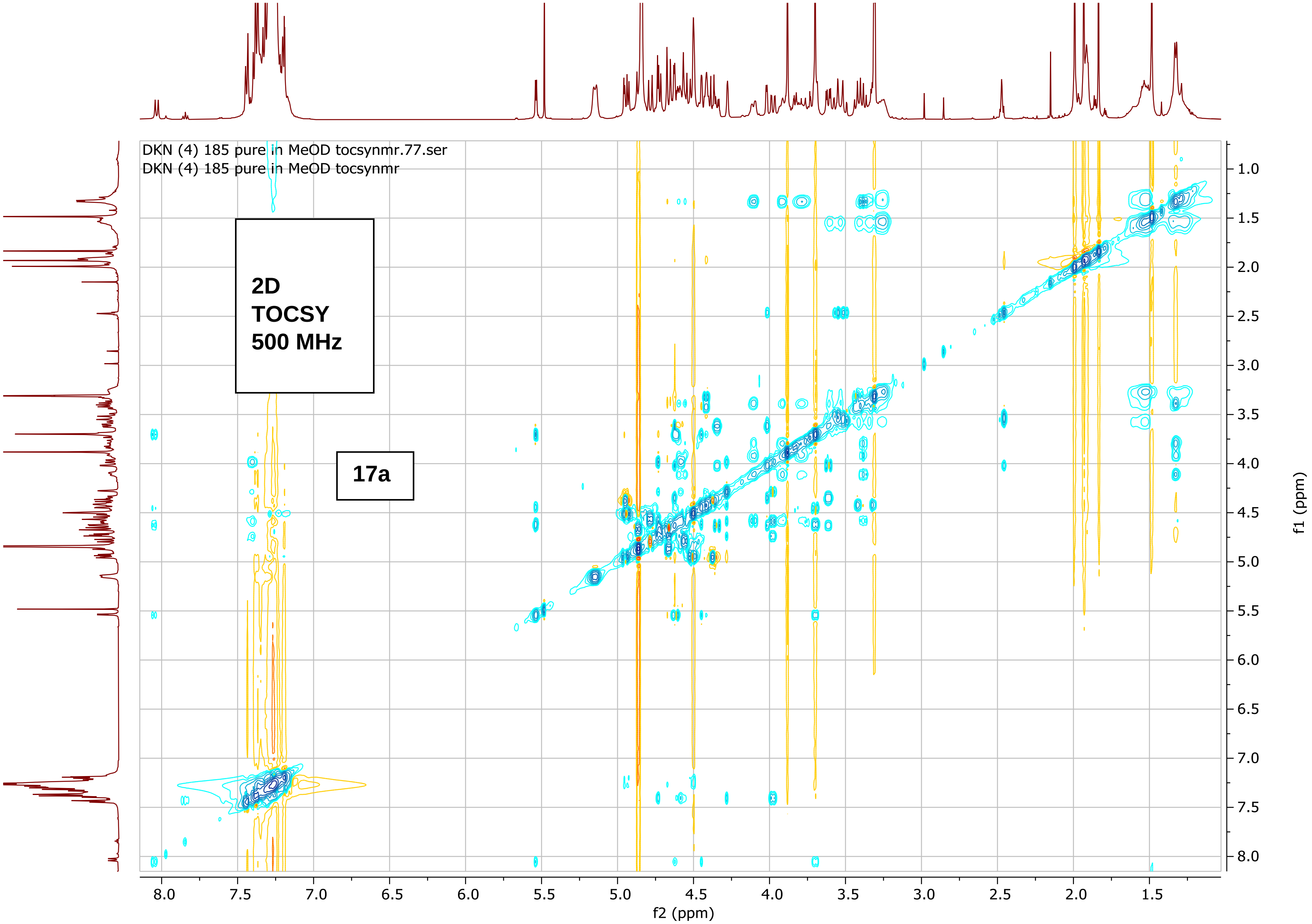
DKN (4) 185 pure in MeOD tocsynmr.77.ser
DKN (4) 185 pure in MeOD tocsynmr
2D TOCSY 500 MHz
17a
f2 (ppm)
f1 (ppm)
8.0 7.5 7.0 6.5 6.0 5.5 5.0 4.5 4.0 3.5 3.0 2.5 2.0 1.5
1.0 1.5 2.0 2.5 3.0 3.5 4.0 4.5 5.0 5.5 6.0 6.5 7.0 7.5 8.0

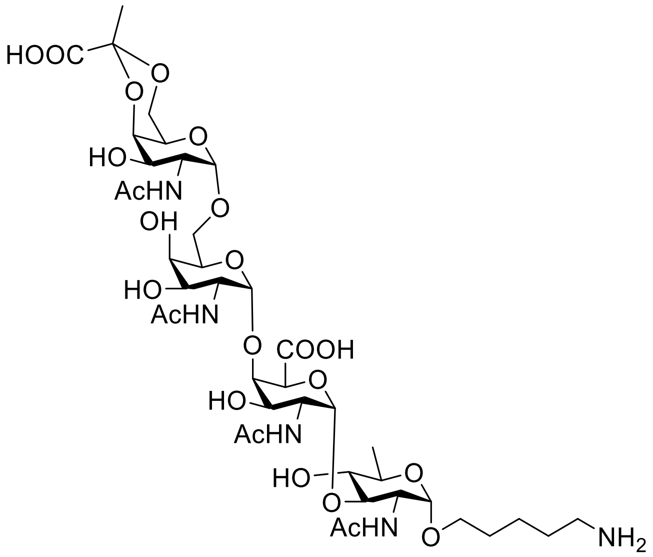

NMR done at 318K

Solvent: D_2O

NMR Instrument: Bruker AVANCE Neo 700 MHz spectrometer

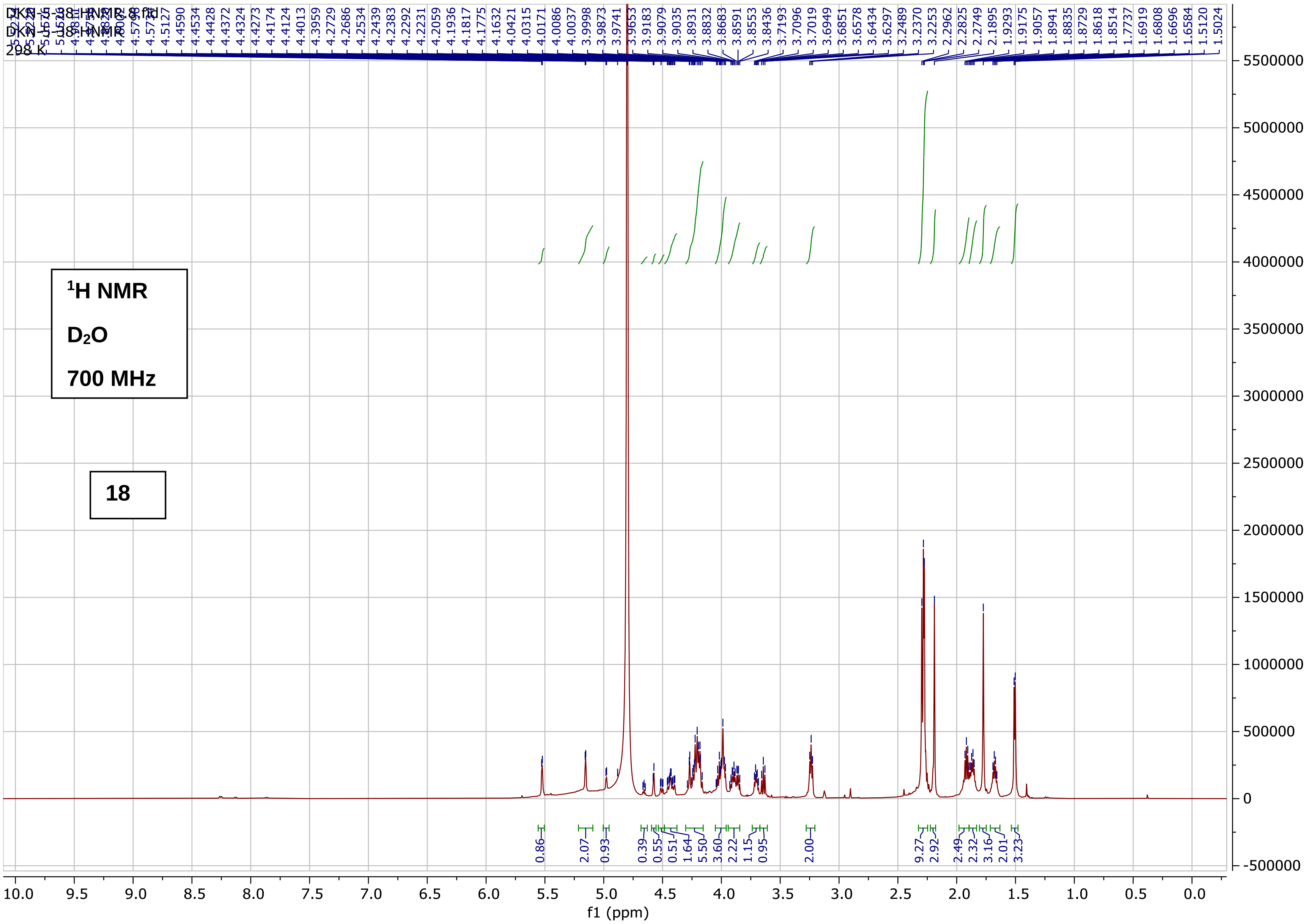

^{1}H NMR
D$_2$O
700 MHz
18
f1 (ppm)

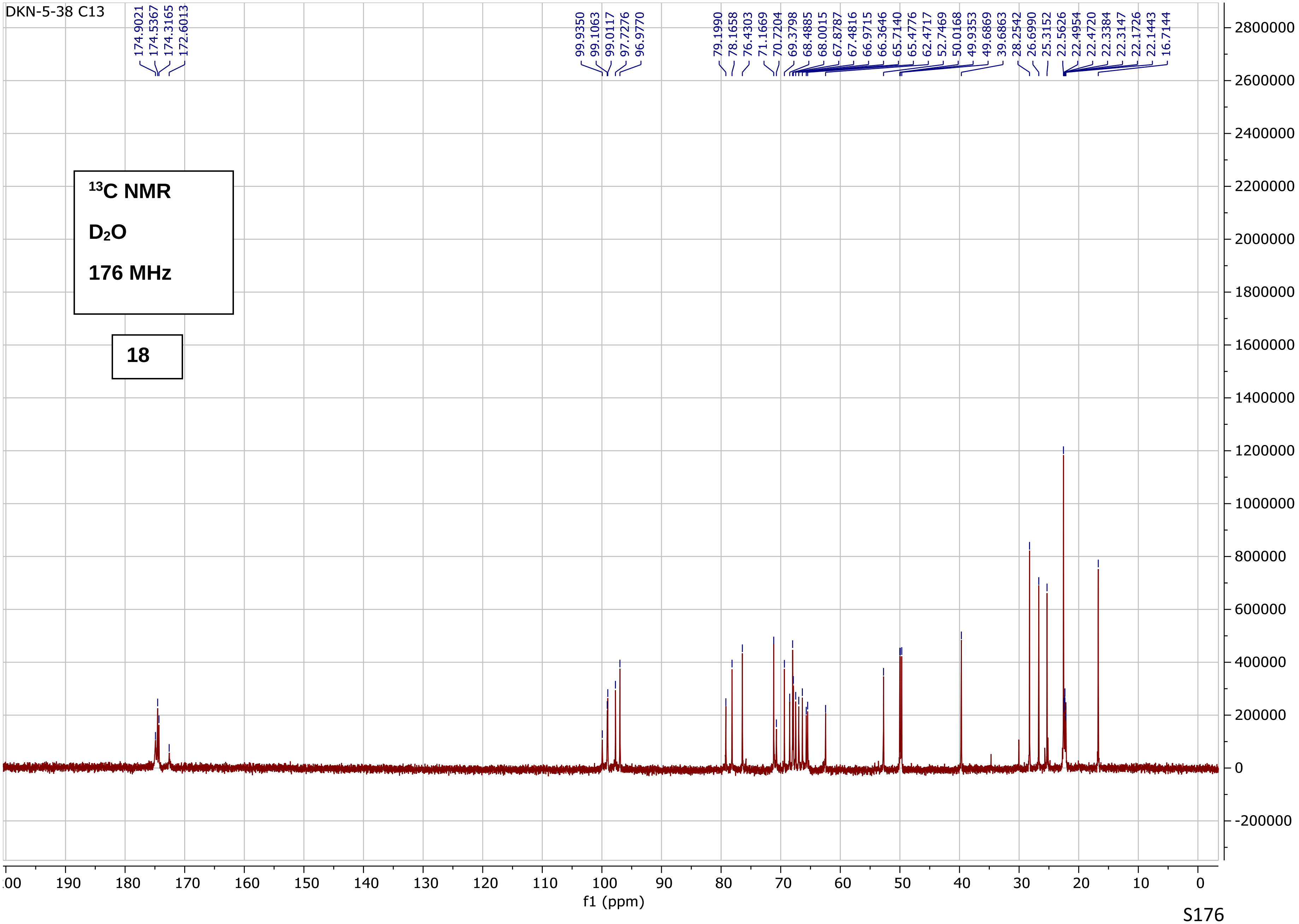
DKN-5-38 C13
174.9021
174.5367
174.3165
172.6013
99.9350
99.1063
99.0117
97.7276
96.9770
79.1990
78.1658
76.4303
71.1669
70.7204
69.3798
68.4885
68.0015
67.8787
67.4816
66.9715
66.3646
65.7140
65.4776
62.4717
52.7469
50.0168
49.9353
49.6869
39.6863
28.2542
26.6990
25.3152
22.5626
22.4954
22.4720
22.3384
22.3147
22.1726
22.1443
16.7144
^{13}C NMR
D_2O
176 MHz
18
f1 (ppm)

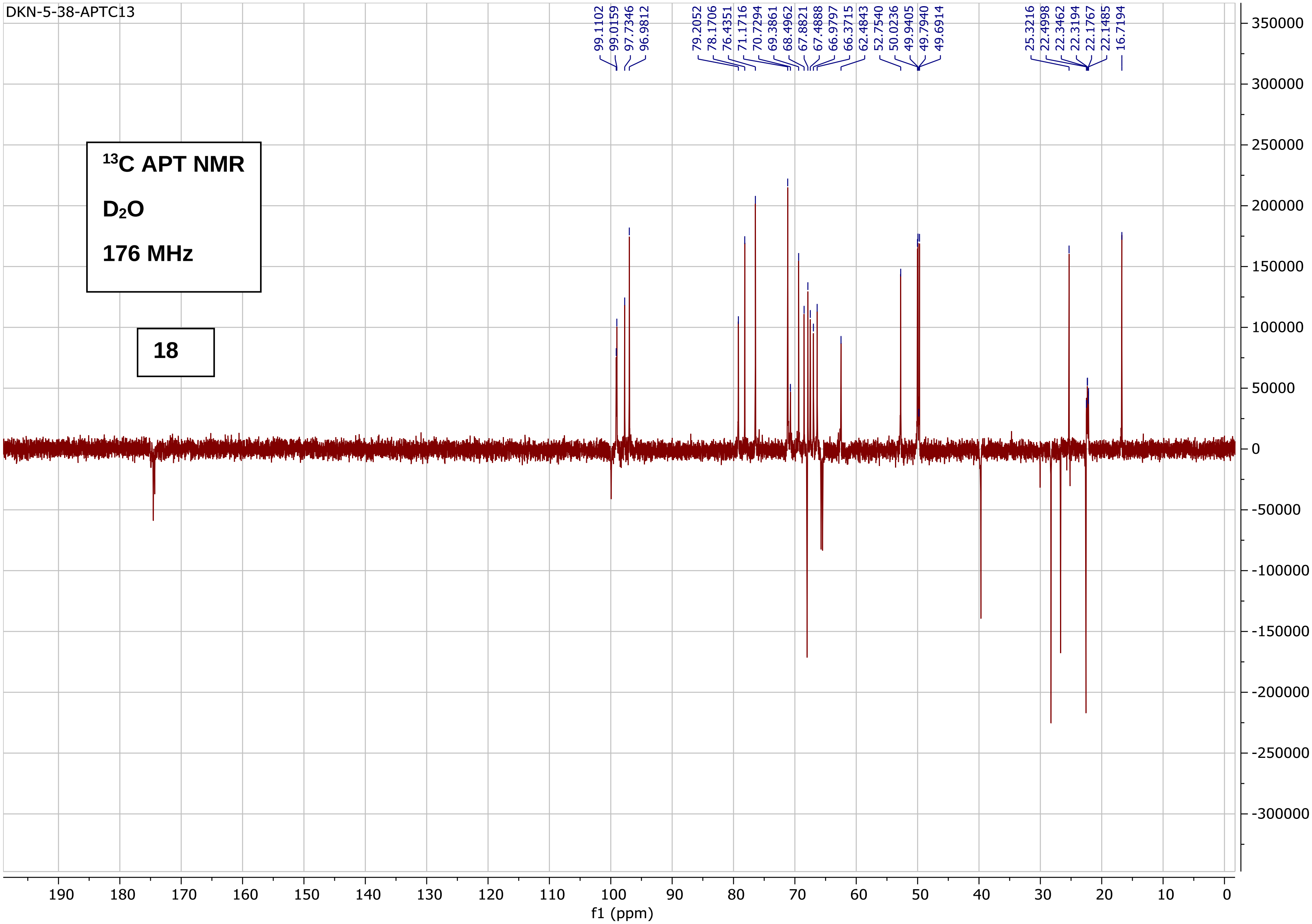

DKN-5-38-APTC13
^{13}C APT NMR
D$_2$O
176 MHz
18
99.1102
99.0159
97.7346
96.9812
79.2052
78.1706
76.4351
71.1716
70.7294
69.3861
68.4962
67.8821
67.4888
66.9797
66.3715
62.4843
52.7540
50.0236
49.9405
49.7940
49.6914
25.3216
22.4998
22.3462
22.3194
22.1767
22.1485
16.7194
f1 (ppm)

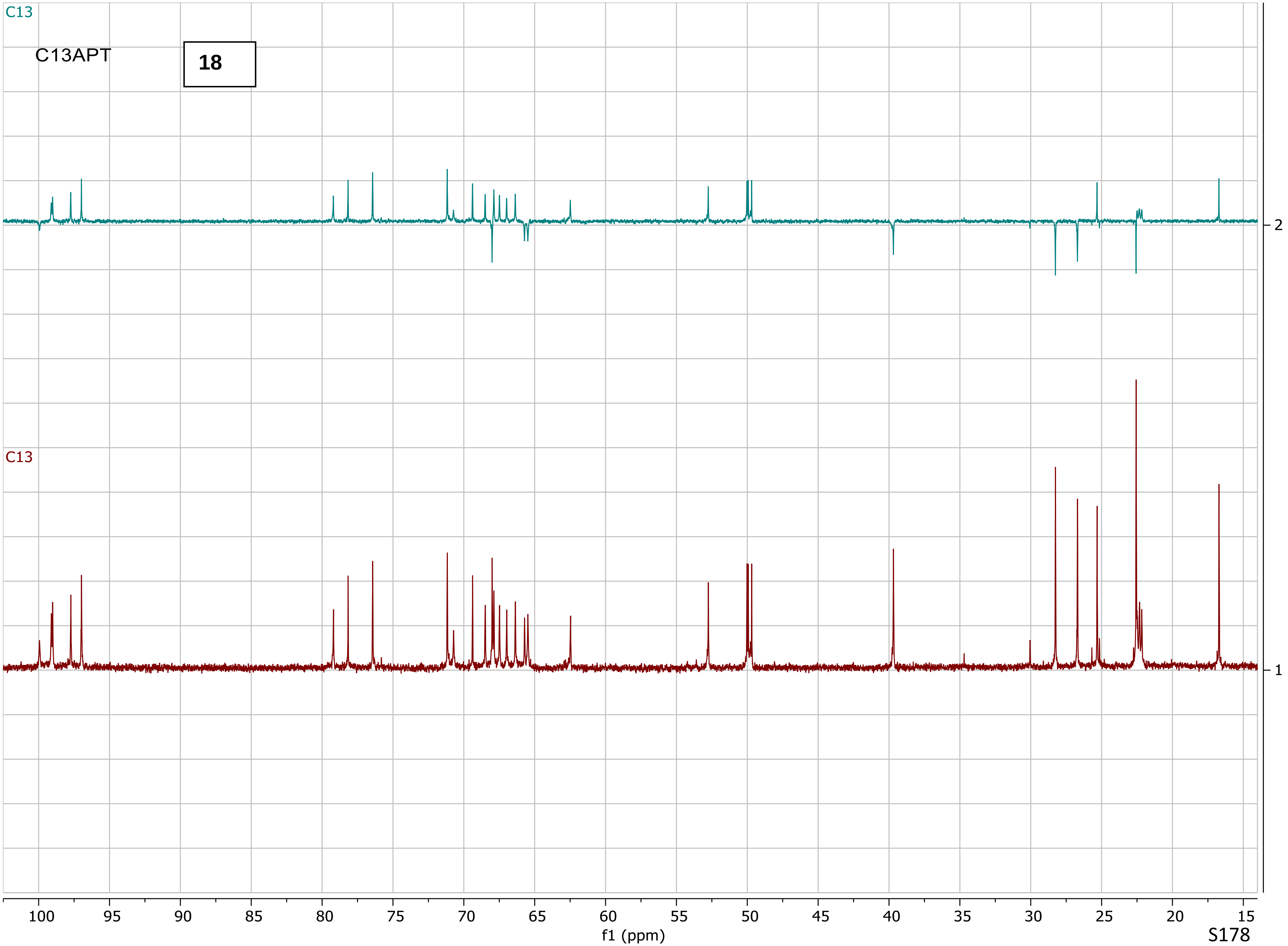
C13
C13APT
18
C13
100 95 90 85 80 75 70 65 60 55 50 45 40 35 30 25 20 15
f1 (ppm)
2
1

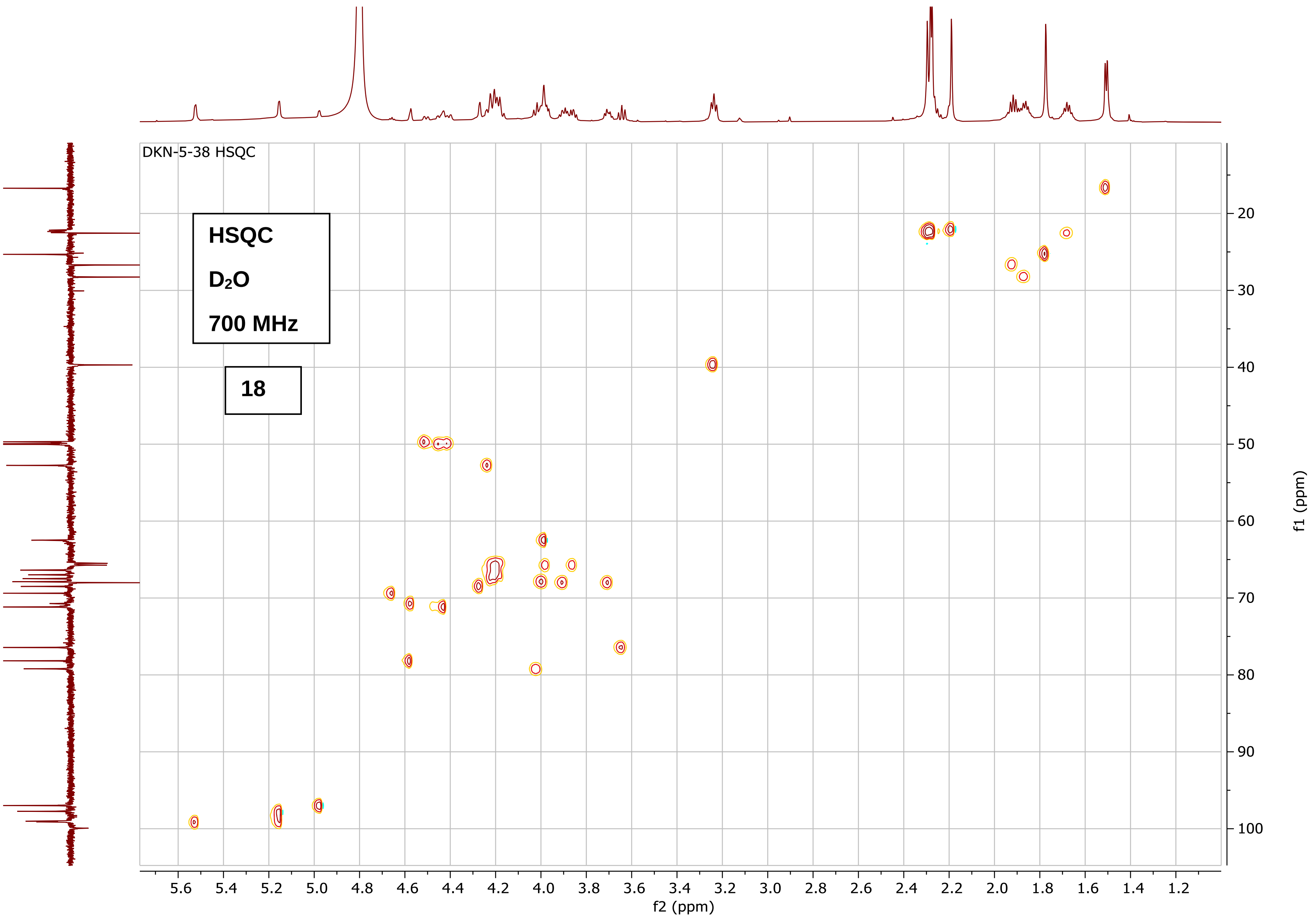
DKN-5-38 HSQC
HSQC
D_2O
700 MHz
18
f2 (ppm)
f1 (ppm)

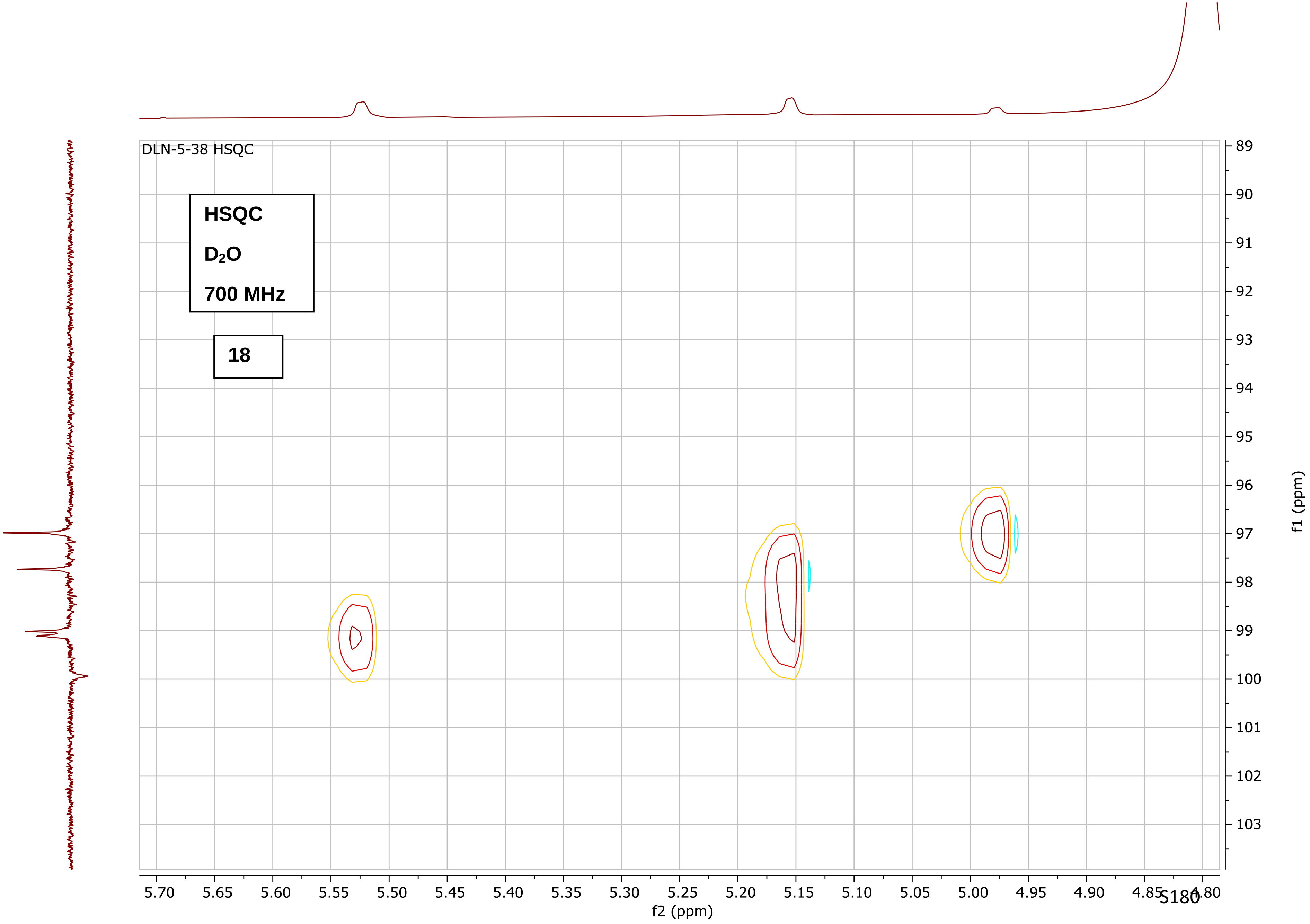
DLN-5-38 HSQC
HSQC
D2O
700 MHz
18
f2 (ppm)
f1 (ppm)
5.70 5.65 5.60 5.55 5.50 5.45 5.40 5.35 5.30 5.25 5.20 5.15 5.10 5.05 5.00 4.95 4.90 4.85 4.80
89 90 91 92 93 94 95 96 97 98 99 100 101 102 103

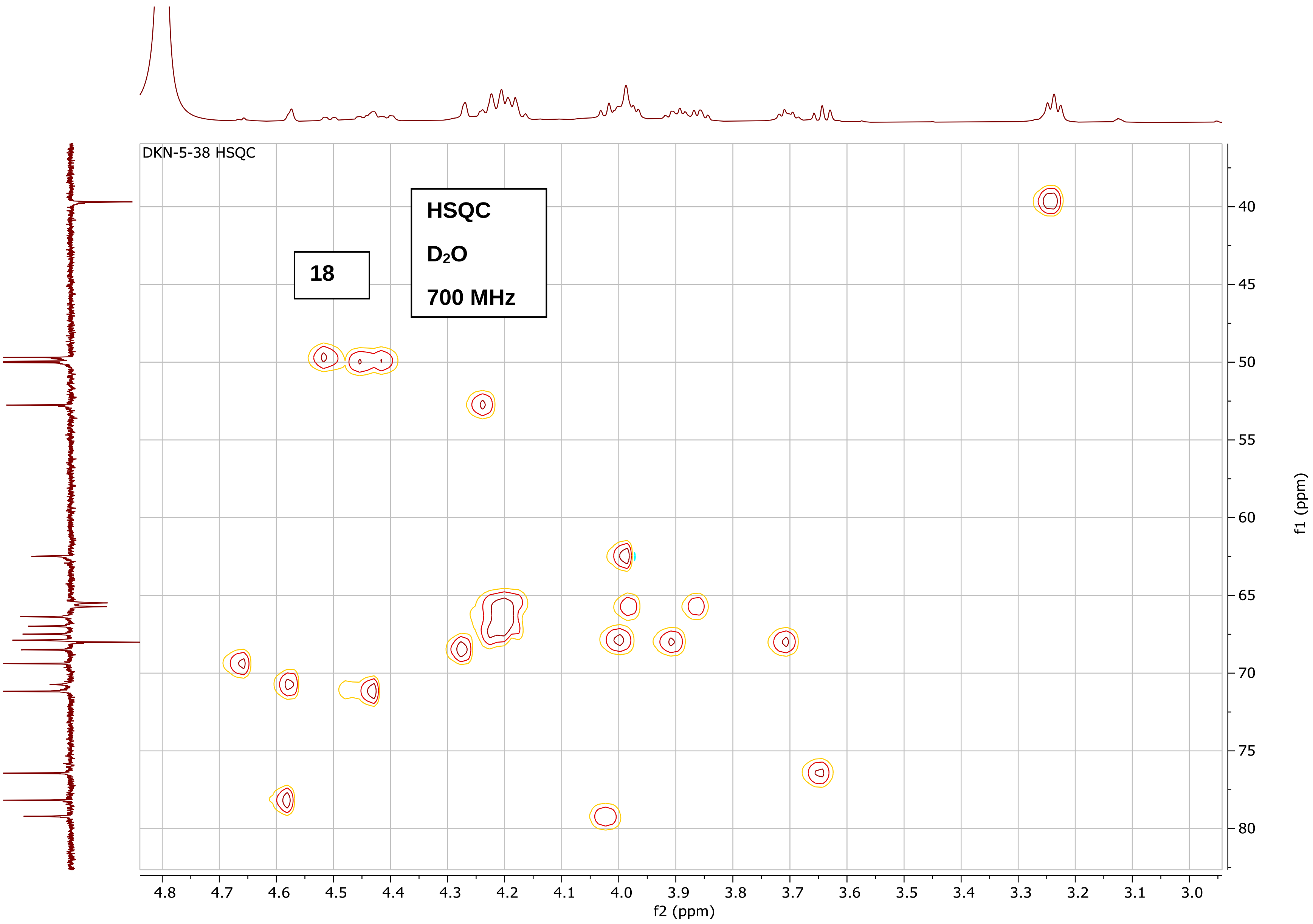
DKN-5-38 HSQC
HSQC
D2O
700 MHz
18
f2 (ppm)
f1 (ppm)
4.8 4.7 4.6 4.5 4.4 4.3 4.2 4.1 4.0 3.9 3.8 3.7 3.6 3.5 3.4 3.3 3.2 3.1 3.0
40 45 50 55 60 65 70 75 80

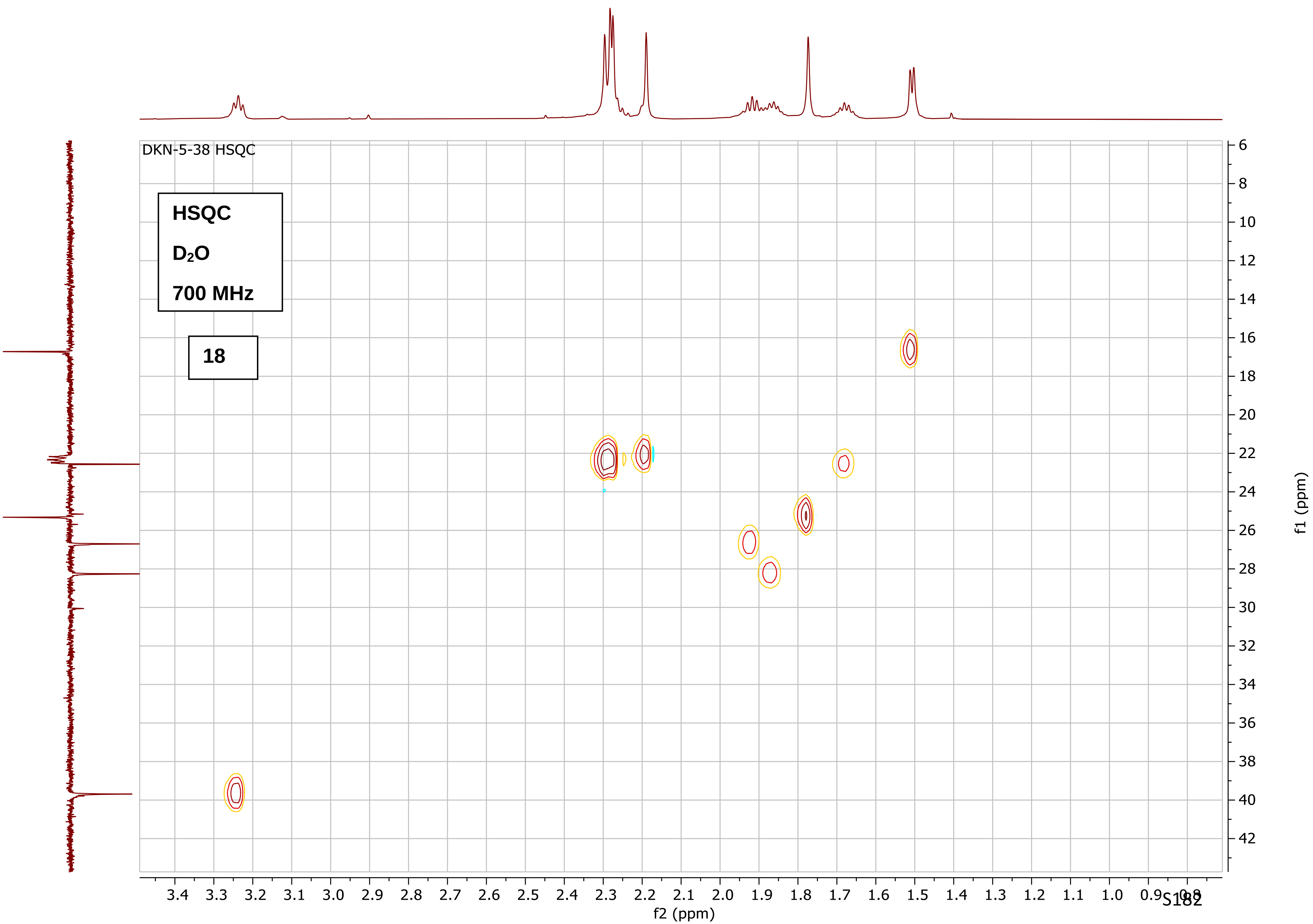

DKN-5-38 HSQC
HSQC
D_2O
700 MHz
18
f2 (ppm)
f1 (ppm)

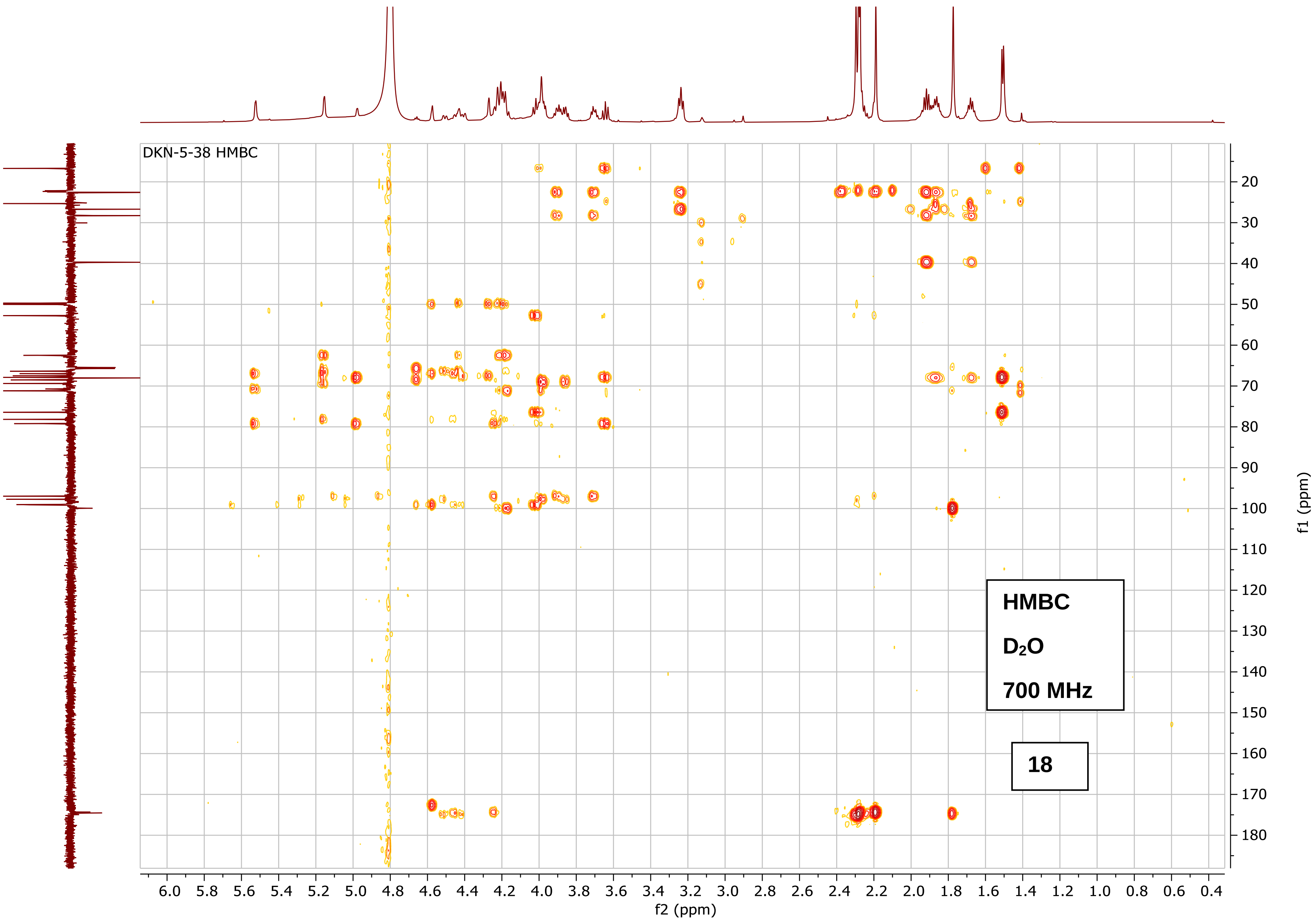
DKN-5-38 HMBC
HMBC
D2O
700 MHz
18
f2 (ppm)
f1 (ppm)

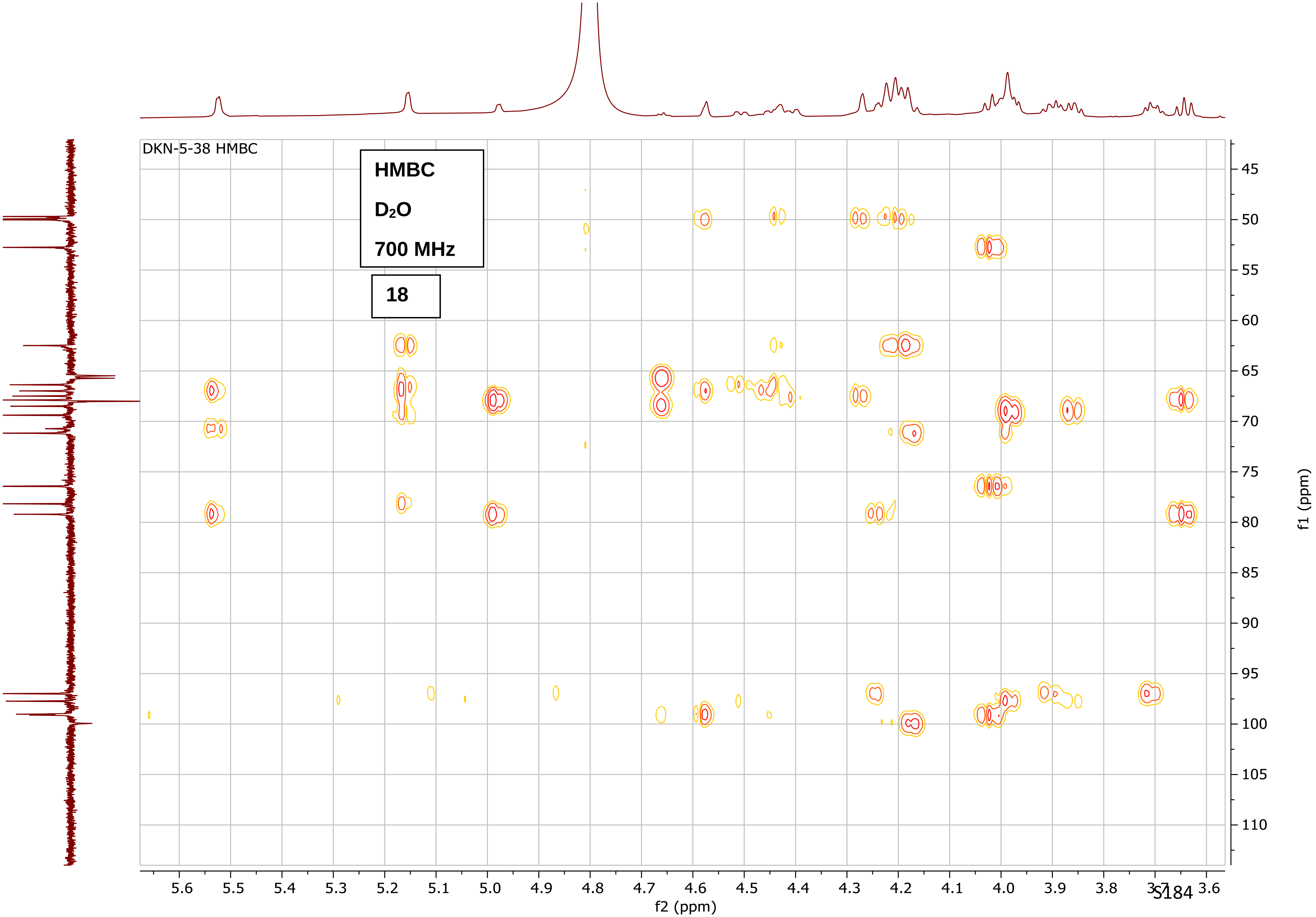

DKN-5-38 HMBC
HMBC
D2O
700 MHz
18
f2 (ppm)
f1 (ppm)

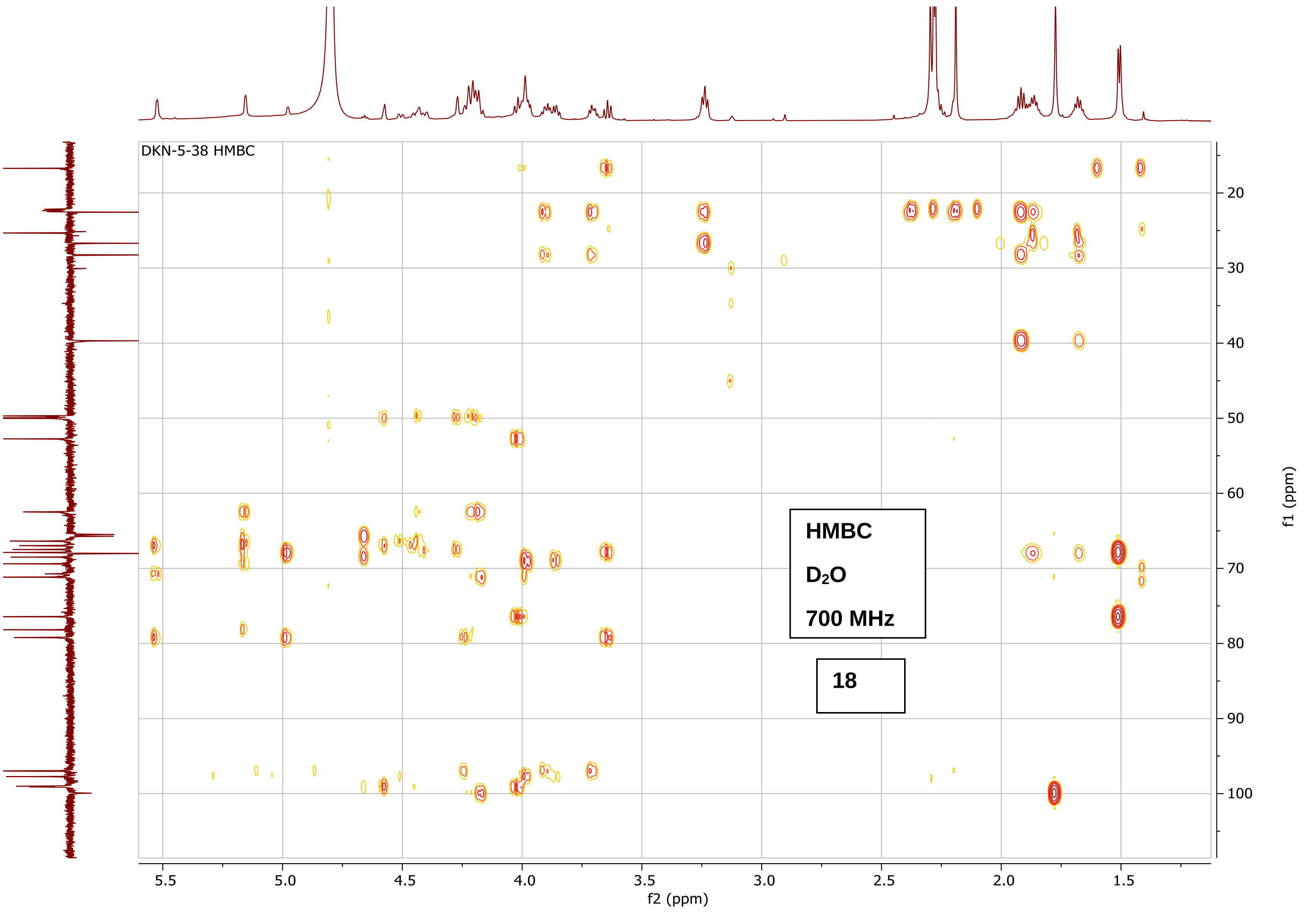
DKN-5-38 HMBC
HMBC
D2O
700 MHz
18
f2 (ppm)
f1 (ppm)
5.5
5.0
4.5
4.0
3.5
3.0
2.5
2.0
1.5
20
30
40
50
60
70
80
90
100

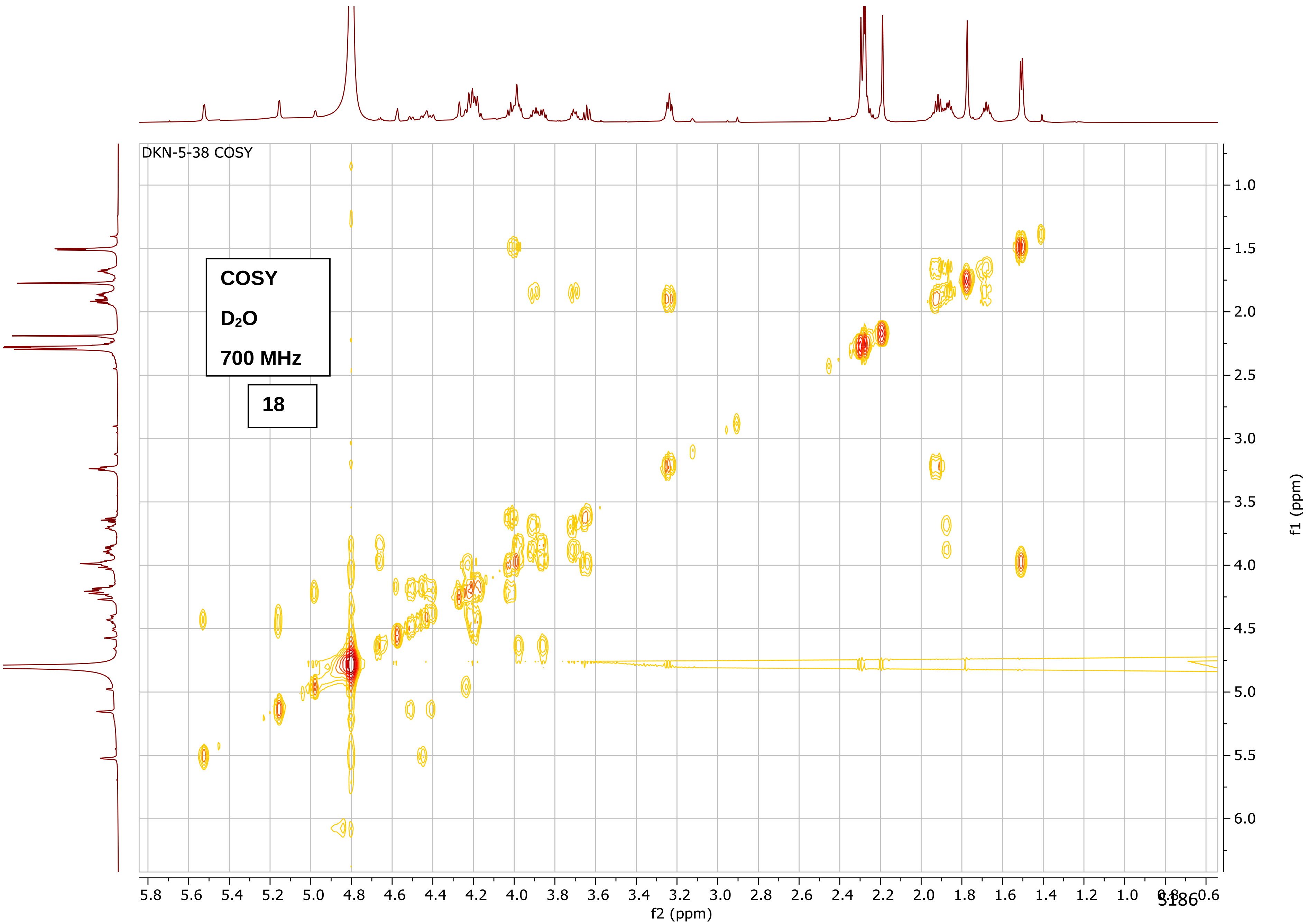
DKN-5-38 COSY
COSY
D_2O
700 MHz
18
f2 (ppm)
f1 (ppm)

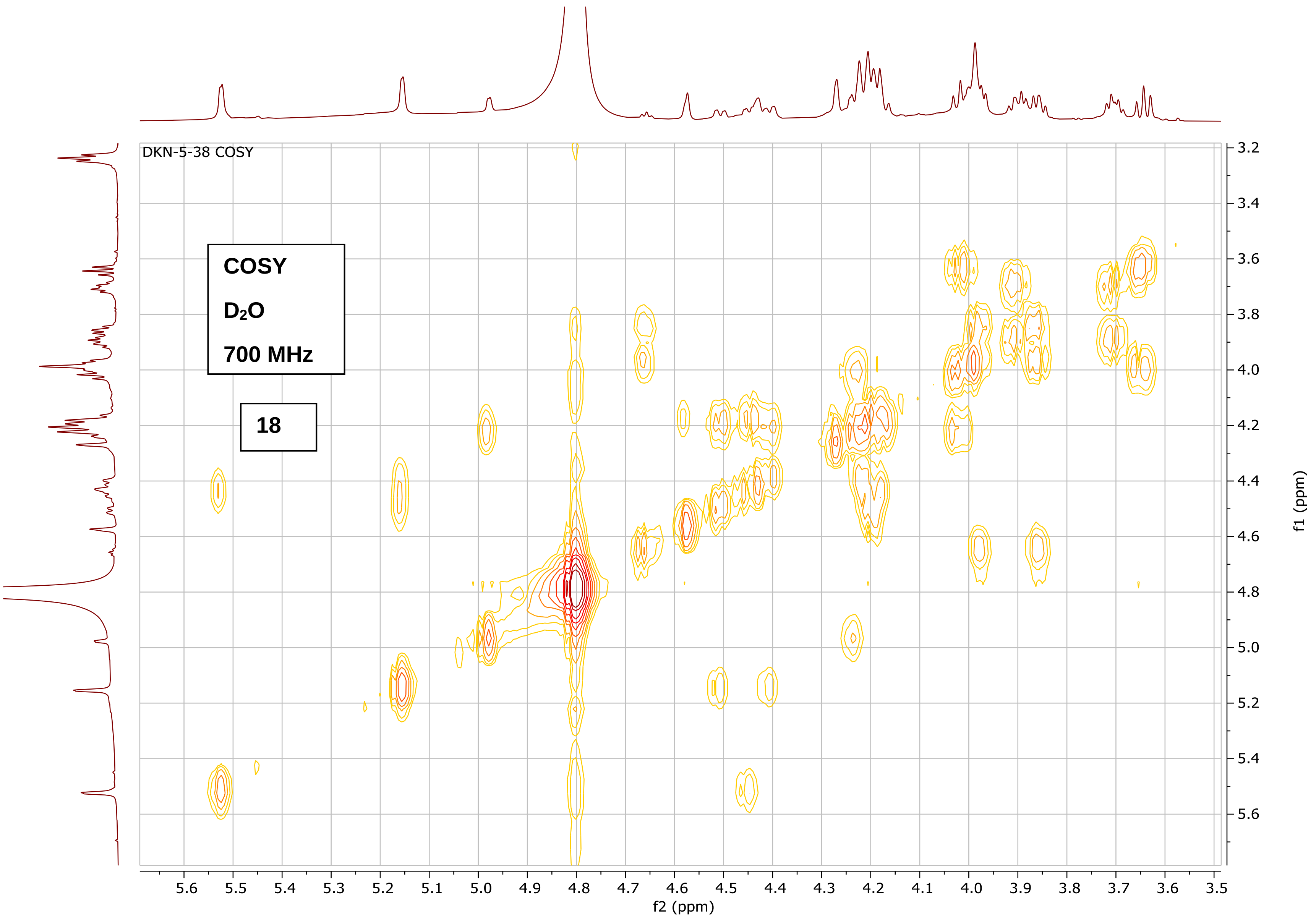

DKN-5-38 COSY
COSY
D2O
700 MHz
18
f2 (ppm)
f1 (ppm)
5.6
5.5
5.4
5.3
5.2
5.1
5.0
4.9
4.8
4.7
4.6
4.5
4.4
4.3
4.2
4.1
4.0
3.9
3.8
3.7
3.6
3.5
3.2
3.4

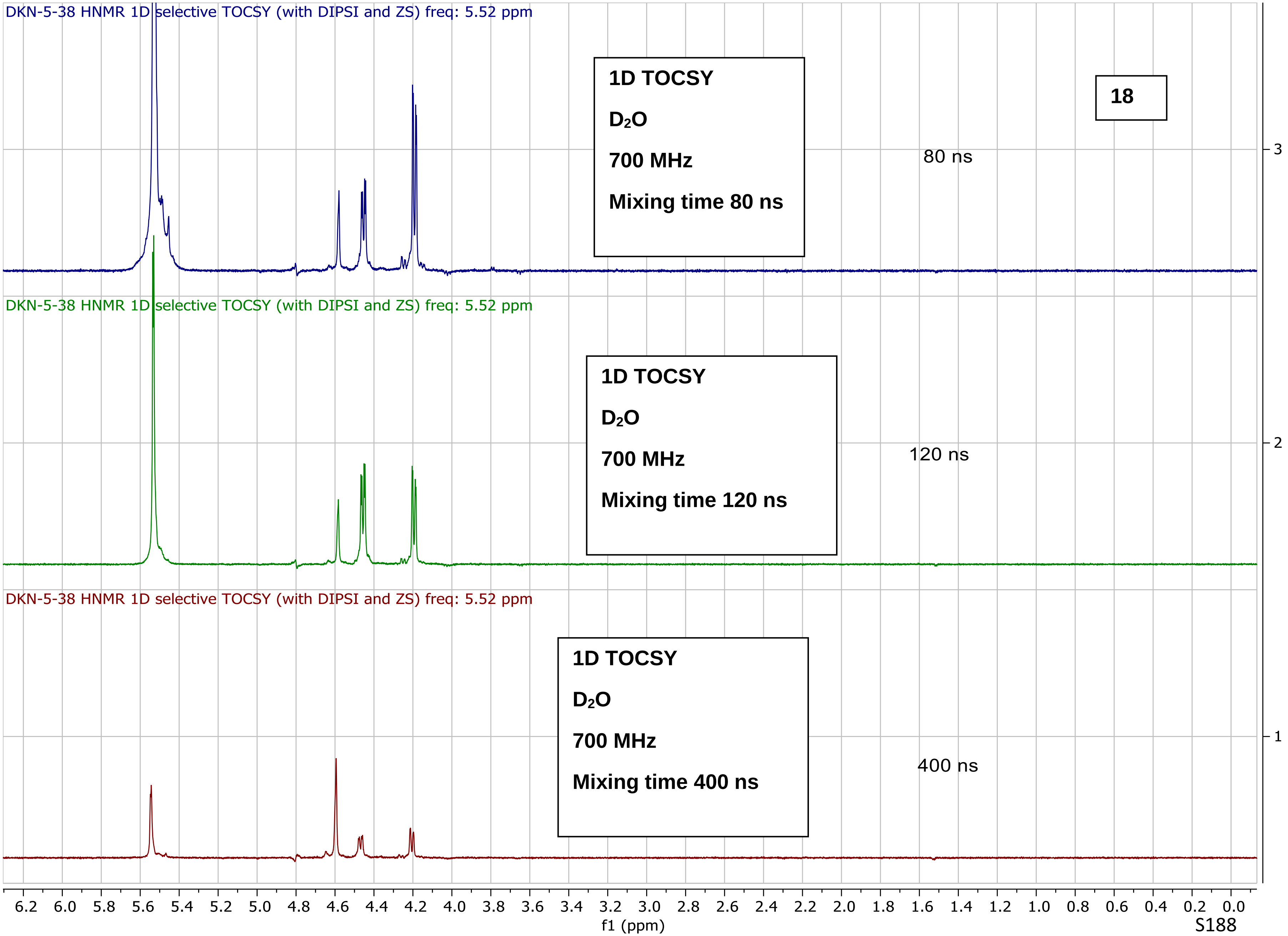
DKN-5-38 HNMR 1D selective TOCSY (with DIPSI and ZS) freq: 5.52 ppm
1D TOCSY
D2O
700 MHz
Mixing time 80 ns
18
80 ns
DKN-5-38 HNMR 1D selective TOCSY (with DIPSI and ZS) freq: 5.52 ppm
1D TOCSY
D2O
700 MHz
Mixing time 120 ns
120 ns
DKN-5-38 HNMR 1D selective TOCSY (with DIPSI and ZS) freq: 5.52 ppm
1D TOCSY
D2O
700 MHz
Mixing time 400 ns
400 ns
f1 (ppm)

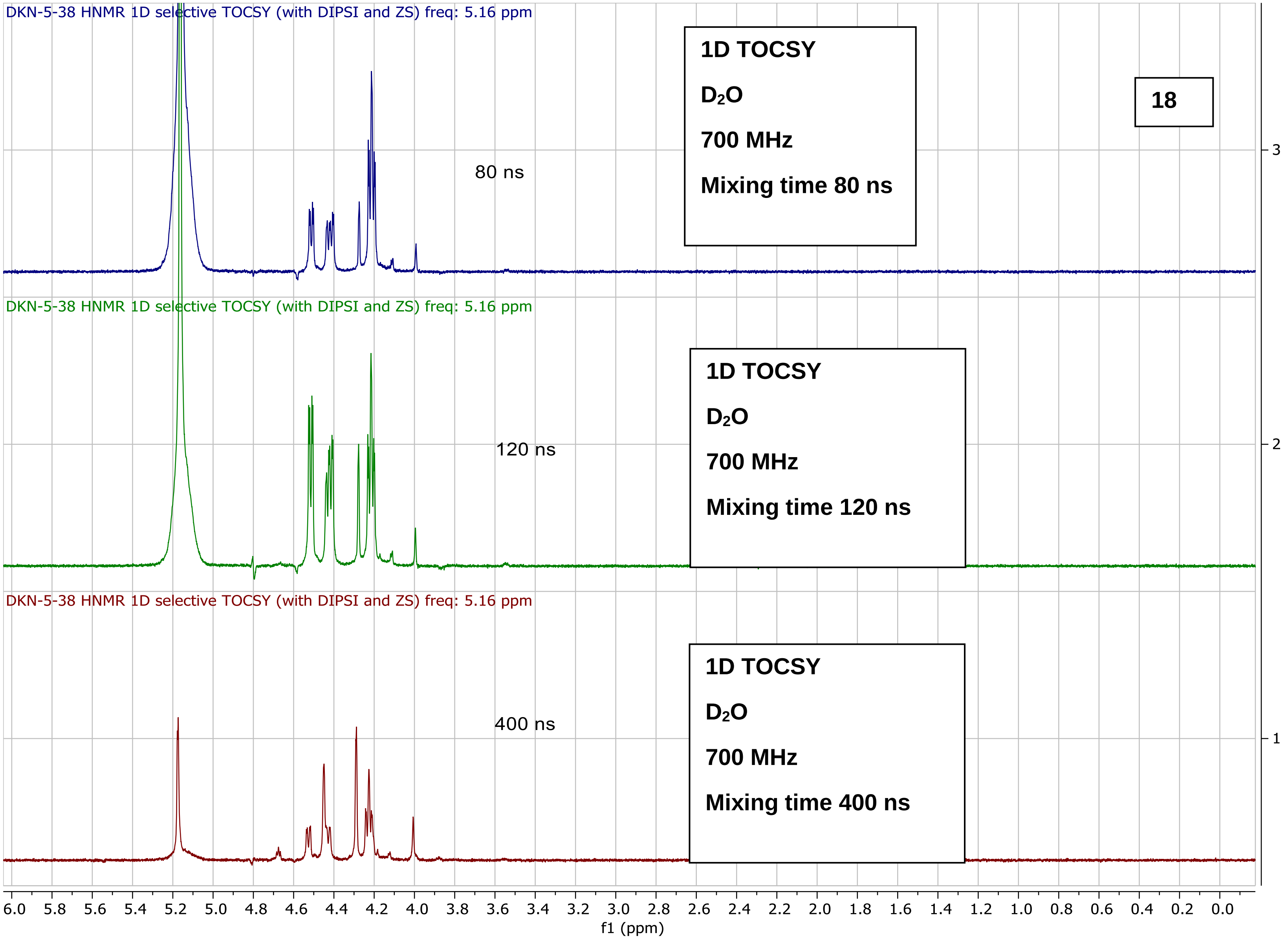

DKN-5-38 HNMR 1D selective TOCSY (with DIPSI and ZS) freq: 5.16 ppm
80 ns
1D TOCSY
D2O
700 MHz
Mixing time 80 ns
18
DKN-5-38 HNMR 1D selective TOCSY (with DIPSI and ZS) freq: 5.16 ppm
120 ns
1D TOCSY
D2O
700 MHz
Mixing time 120 ns
DKN-5-38 HNMR 1D selective TOCSY (with DIPSI and ZS) freq: 5.16 ppm
400 ns
1D TOCSY
D2O
700 MHz
Mixing time 400 ns
6.0 5.8 5.6 5.4 5.2 5.0 4.8 4.6 4.4 4.2 4.0 3.8 3.6 3.4 3.2 3.0 2.8 2.6 2.4 2.2 2.0 1.8 1.6 1.4 1.2 1.0 0.8 0.6 0.4 0.2 0.0
f1 (ppm)
3
2
1

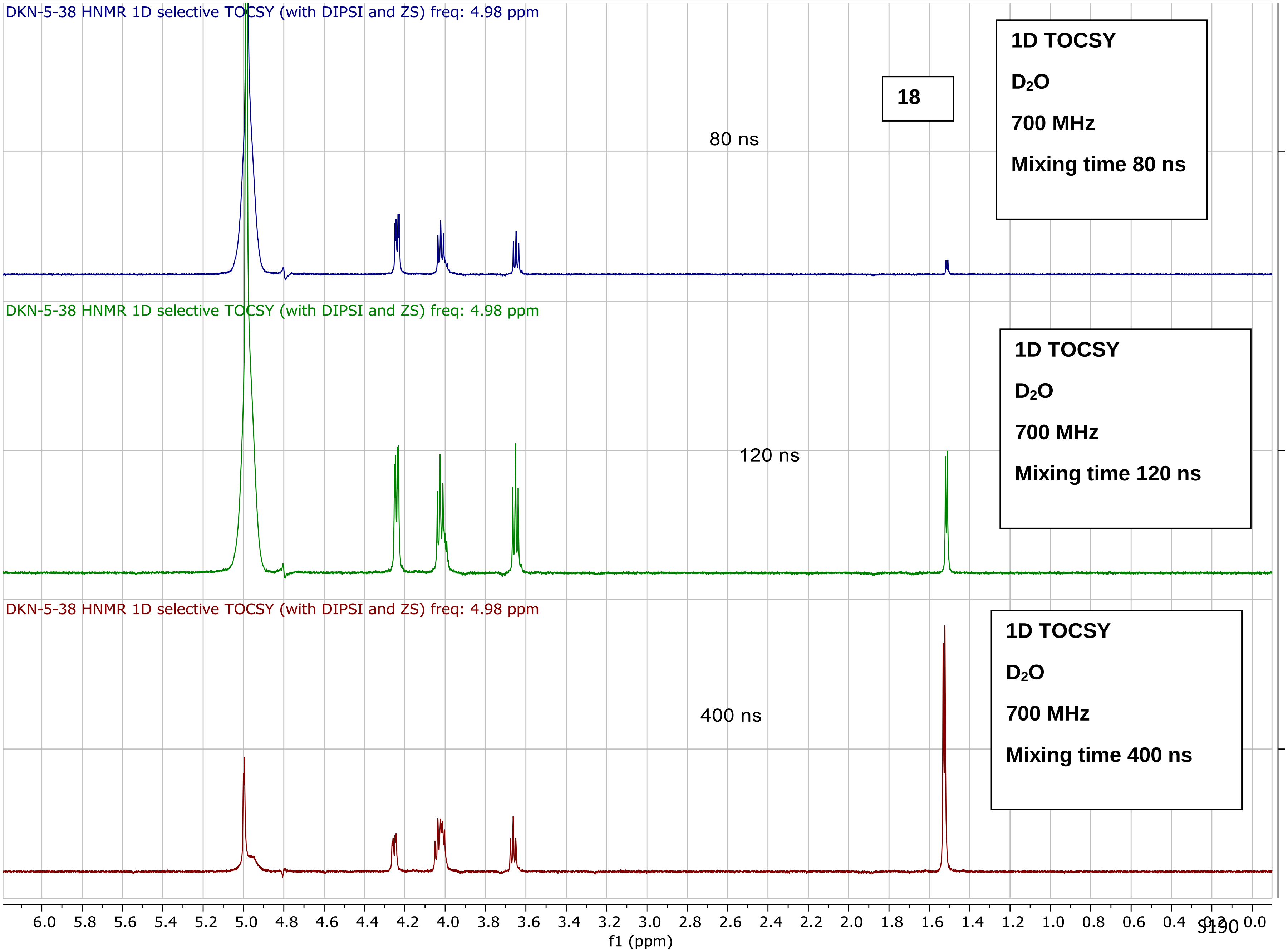
DKN-5-38 HNMR 1D selective TOCSY (with DIPSI and ZS) freq: 4.98 ppm
1D TOCSY
D2O
700 MHz
Mixing time 80 ns
18
80 ns
DKN-5-38 HNMR 1D selective TOCSY (with DIPSI and ZS) freq: 4.98 ppm
1D TOCSY
D2O
700 MHz
Mixing time 120 ns
120 ns
DKN-5-38 HNMR 1D selective TOCSY (with DIPSI and ZS) freq: 4.98 ppm
1D TOCSY
D2O
700 MHz
Mixing time 400 ns
400 ns
6.0 5.8 5.6 5.4 5.2 5.0 4.8 4.6 4.4 4.2 4.0 3.8 3.6 3.4 3.2 3.0 2.8 2.6 2.4 2.2 2.0 1.8 1.6 1.4 1.2 1.0 0.8 0.6 0.4 0.2 0.0
f1 (ppm)
3
2
1

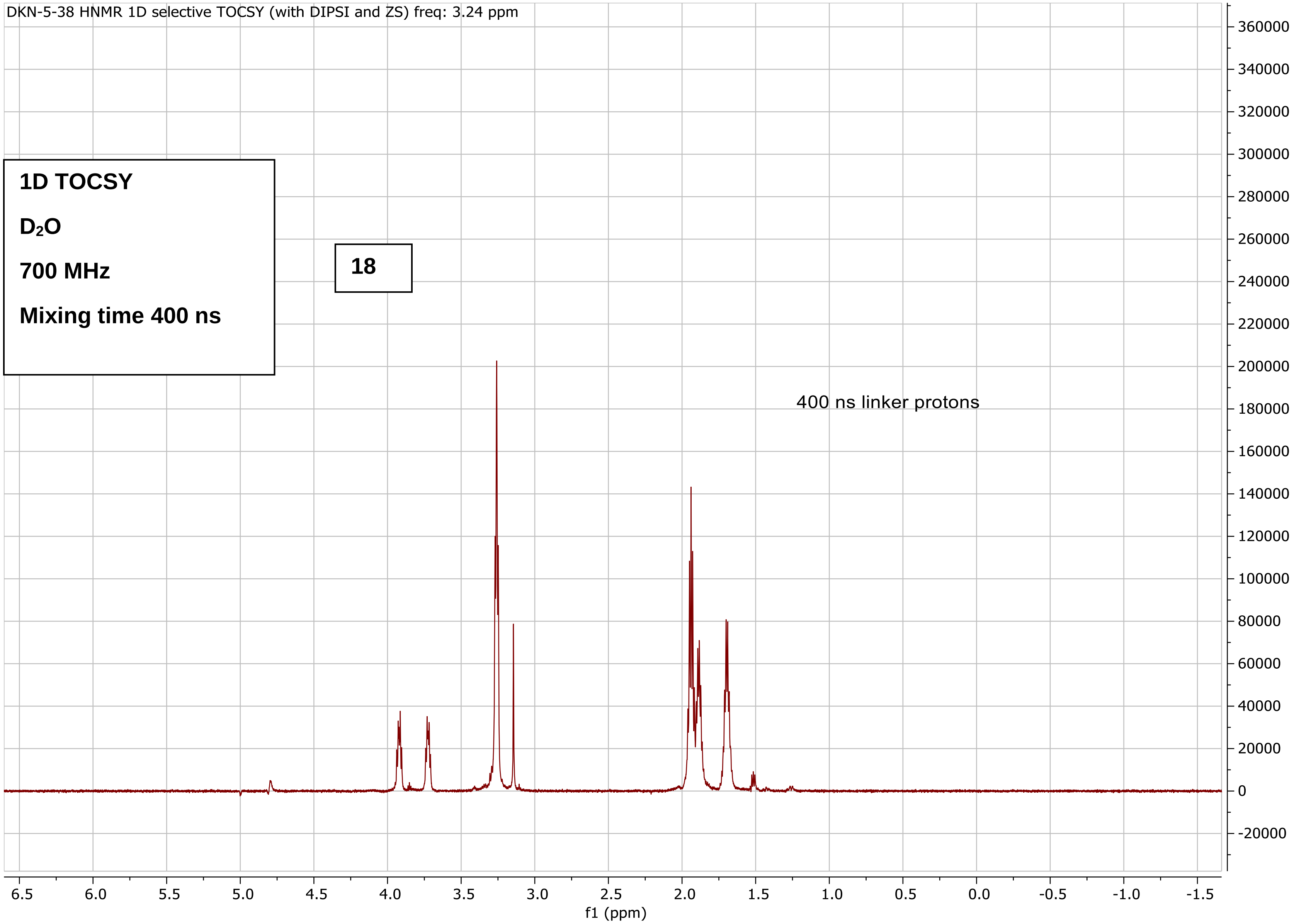
DKN-5-38 HNMR 1D selective TOCSY (with DIPSI and ZS) freq: 3.24 ppm
1D TOCSY
D2O
700 MHz
Mixing time 400 ns
18
400 ns linker protons
f1 (ppm)
